# A composite metric for assessing data on mortality and causes of death: the vital statistics performance index

**DOI:** 10.1186/1478-7954-12-14

**Published:** 2014-05-14

**Authors:** David E Phillips, Rafael Lozano, Mohsen Naghavi, Charles Atkinson, Diego Gonzalez-Medina, Lene Mikkelsen, Christopher JL Murray, Alan D Lopez

**Affiliations:** 1 Institute for Health Metrics and Evaluation, University of Washington, 2301 5th Ave. Suite 600, Seattle, WA 98121, USA; 2 National Institute of Public Health, Universidad No. 655 Colonia Santa María Ahuacatitlán, Cerrada Los Pinos y Caminera, Cuernavaca, MOR 62100, México; 3 LM Consulting, Independent Consultant, 4/78 Cairns St., Brisbane, QLD 4169, Australia; 4 School of Population and Global Health, University of Melbourne, 207 Bouverie St., Level 5, Melbourne, VIC 3010, Australia

**Keywords:** Mortality, Causes of death, Vital statistics, Civil registration, Vital registration, Data quality, Health information systems

## Abstract

**Background:**

Timely and reliable data on causes of death are fundamental for informed decision-making in the health sector as well as public health research. An in-depth understanding of the quality of data from vital statistics (VS) is therefore indispensable for health policymakers and researchers. We propose a summary index to objectively measure the performance of VS systems in generating reliable mortality data and apply it to the comprehensive cause of death database assembled for the Global Burden of Disease (GBD) 2013 Study.

**Methods:**

We created a *Vital Statistics Performance Index*, a composite of six dimensions of VS strength, each assessed by a separate empirical indicator. The six dimensions include: quality of cause of death reporting, quality of age and sex reporting, internal consistency, completeness of death reporting, level of cause-specific detail, and data availability/timeliness. A simulation procedure was developed to combine indicators into a single index. This index was computed for all country-years of VS in the GBD 2013 cause of death database, yielding annual estimates of overall VS system performance for 148 countries or territories.

**Results:**

The six dimensions impacted the accuracy of data to varying extents. VS performance declines more steeply with declining simulated completeness than for any other indicator. The amount of detail in the cause list reported has a concave relationship with overall data accuracy, but is an important driver of observed VS performance. Indicators of cause of death data quality and age/sex reporting have more linear relationships with simulated VS performance, but poor cause of death reporting influences observed VS performance more strongly. VS performance is steadily improving at an average rate of 2.10% per year among the 148 countries that have available data, but only 19.0% of global deaths post-2000 occurred in countries with well-performing VS systems.

**Conclusions:**

Objective and comparable information about the performance of VS systems and the utility of the data that they report will help to focus efforts to strengthen VS systems. Countries and the global health community alike need better intelligence about the accuracy of VS that are widely and often uncritically used in population health research and monitoring.

## Background

Vital statistics (VS) are tabulations of birth, marriage, divorce, and death certificates typically generated by civil registration systems. VS are usually based on legal requirements regarding the registration and certification of vital events. While the importance of civil registration for the identification of individuals is well recognized, VS are also critically important for informing public health policies and programs [1]. Although alternative sources of data exist, VS derived from national civil registration systems are the optimal source of mortality, fertility, and cause of death data because they are intended to cover the entire population, are available for subnational populations and, in principle, result from a medically certified cause of each death.

The importance of VS for public health extends beyond policy to research, program implementation, and evaluation. Good-quality VS greatly facilitate the reliable monitoring of progress with health development goals and in evaluating performance [2-10]. Yet there has been comparatively little focus on the monitoring and evaluation of VS strengthening activities themselves, and limited tools and techniques are available for doing so [5-8]. For the purposes of this analysis, a VS system is defined as any collection of agencies, institutions, and protocols established to ensure the certification, aggregation, and dissemination of VS.

But what do we mean by VS performance? In terms of its intended purpose, a well-performing VS system might be thought of as one which generates information that accurately reflects the current and past epidemiologic circumstances of its population. In other words, the performance of a VS system is the extent to which it can produce representative VS on the health, and particularly causes of death, in populations.

The few techniques that do exist for assessing performance and evaluating progress with VS systems can be broadly classified into three categories:

1. *Expert audit*: An independent consultant examines the features of a VS system according to a predetermined agenda, focusing on inputs, processes, and outputs. This is often done in person and by external experts.

2. *Self-assessment*: Key stakeholders in countries responsible for establishing and maintaining VS systems collectively perform a self-assessment in order to assess various aspects of inputs, processes, and/or outputs and identify system bottlenecks.

3. *Empirical output assessment*: VS themselves are evaluated according to a predetermined set of indicators.

All three approaches have also been used to examine and diagnose broader health systems, but most of the studies that focus specifically on VS systems are heavily based on expert audit and self-assessment; few use output assessment techniques [6,9-16]. To our knowledge, there have been only four attempts to evaluate VS via output assessment, three of which are closely related, with the fourth focusing primarily on births [17-20].

Output assessment offers many advantages over expert audit and self-assessment. Perhaps most important is that it is objective. No specific local knowledge or judgment is required to quantify problems within datasets, provided that those problems can be clearly defined. Expert audit and self-assessment, in contrast, rely on subjective observations made by the auditor(s) and depend on specialized knowledge of the specific system being evaluated. Second, empirical evaluation of data is highly reproducible, unlike expert evaluations or self-assessments which may produce differing assessments of the same system if repeated. Third, output assessment has the distinct advantage that it can be applied to evaluate VS retrospectively (to the extent that data are available). Finally, output assessment is largely costless when based on available data. On the contrary, expert audit and self-assessment must be conducted on a system by system basis, which may be both prohibitively expensive and time consuming. Objective methods for data quality evaluation can be applied with minimal cost for any number of countries simultaneously.

The traditional approach to evaluate VS is to examine completeness (percent of all births or deaths registered) or coverage (percent of the national population included in the VS system). While these are necessary, they are not sufficient to comprehensively describe VS performance. Other epidemiologic information, such as the decedent’s age, sex, and cause of death are critically important outputs of a VS system, while the quality and availability of VS data are critical to their usefulness.

Given the many different dimensions that might legitimately be viewed as contributing to the overall quality and usefulness of VS data, comparative analyses of system performance across space and time would be facilitated if they could be combined into a single indicator. Previous attempts to do so have relied on expert opinion to determine the relative importance of each dimension of VS data quality, introducing a degree of subjectivity and arbitrariness to what is otherwise an empirical approach [19]. The analytic framework presented in this paper identifies six dimensions for assessing the overall quality of VS data, and uses a simulation environment to empirically determine the relative weight of each dimension. Simulation results are applied to the database assembled for the upcoming Global Burden of Disease Study 2013 (GBD 2013) to estimate a summary metric of VS performance for each country-year from 1980 to 2012, defined here as the *Vital Statistics Performance Index* (VSPI).

## Methods

### Data

The input database for this analysis was the cause of death database developed for GBD 2013 by the Institute for Health Metrics and Evaluation at the University of Washington, which builds upon the previous GBD 2010 study [21,22]. Data were gathered from all known sources of VS, including the World Health Organization (WHO) Mortality Database, the United Nations Demographic Yearbook, individual publications from national ministries of health, and other sources [22]. Each country was categorized into one of seven mutually exclusive epidemiologic regions, which were previously defined for the GBD [23]. A more complete description of the underlying database for GBD can be found elsewhere [21,22].

Using a slightly less conservative definition of VS than the GBD, we have included in this analysis subnational VS where national VS were not available, such as in Ghana, and nationally aggregated hospital mortality records, such as in Bhutan. We have not included sample registration systems. All-cause mortality statistics were analyzed when cause of death statistics were unavailable. Overall, the input database encompassed 148 countries and 3,507 country-years from 1980 to 2012. The mean number of years of data available per country or territory was 24, with availability ranging from one year (for Bangladesh, the Federated States of Micronesia, Kenya, Myanmar, Mozambique, Malawi, Pakistan, and Tanzania) to all 33 years in this time period (from 14 countries). The greatest number of countries or territories in the database occurred for 2005 (122 countries), and the least (25 countries) occurred for 2012. The average annual number of countries or territories with available data was 109. Additional file 1: Table S1 shows the years for which data were available from each country or territory. For 2,903 country-years (82.8%), cause of death data in some form were available, while for the remaining 604 country-years (17.2%), only aggregated all-cause mortality data were available (see Additional file 1: Figure S1).

### Dimensions of vital statistics performance

Six general dimensions, or components, of VS performance were evaluated for all 3,507 country-years: 1) quality of cause of death reporting, 2) quality of age and sex reporting, 3) internal consistency, 4) completeness of death reporting, 5) level of cause-specific detail, and 6) public availability of VS data. We measured each dimension using a single empirical indicator (see Table 1). These indicators were selected using the following criteria: they could be empirically quantified, were comparable across data sources, and likely to be indicative of their corresponding dimension of VS performance.

**Table 1:** Vital statistics performance dimensions and indicators used to measure them

**VS performance dimension**	**Indicator used**	**Formula**
**Quality of cause of death reporting**	Garbage coding	$\frac{\mathrm{Deaths}\;\mathrm{Coded}\;\mathrm{to}\;\mathrm{Garbage}\;\mathrm{Type}\;1_{\mathrm{cy}}\;+\;\left(0.5\;*\;\mathrm{Deaths}\;\mathrm{Coded}\;\mathrm{to}\;\mathrm{Garbage}\;\mathrm{Type}\;2_{\mathrm{cy}}\right)}{\mathrm{All}\;\mathrm{Death}s_{\mathrm{cy}}}$
**Quality of age and sex reporting**	Age or sex unspecified	$\frac{\mathrm{Deaths}\;\mathrm{with}\;\mathrm{Either}\;\mathrm{Unspecified}\;\mathrm{Age}\;\mathrm{or}\;\mathrm{Se}x_{\mathrm{cy}}}{\mathrm{All}\;\mathrm{Death}s_{\mathrm{cy}}}$
**Internal consistency**	Medically impossible diagnoses	$\frac{\mathrm{Deaths}\;\mathrm{Considered}\;\mathrm{Medically}\;\mathrm{Impossible}\;\mathrm{Given}\;\mathrm{the}\;\mathrm{Age}\;\mathrm{or}\;\mathrm{Sex}\;\mathrm{of}\;\mathrm{Deceden}t_{\mathrm{cy}}}{\mathrm{All}\;\mathrm{Death}s_{\mathrm{cy}}}$
**Completeness of death reporting**	Completeness	$\frac{\mathrm{Deaths}\;\mathrm{Enumerated}\;\mathrm{by}\;\mathrm{VS}\;\mathrm{Syste}m_{\mathrm{cy}}}{\mathrm{Expected}\;\mathrm{Number}\;\mathrm{of}\;\mathrm{Death}s_{\mathrm{cy}}}$
**Level of cause-specific detail**	Length of cause list	$\frac{\mathrm{Number}\;\mathrm{of}\;\mathrm{Distinct}\;\mathrm{Causes}\;\mathrm{of}\;\mathrm{Death}\;\mathrm{Reported}\;\mathrm{by}\;\mathrm{VS}\;\mathrm{Dat}a_{\mathrm{cy}}}{\mathrm{Number}\;\mathrm{of}\;\mathrm{Distinct}\;\mathrm{Causes}\;\mathrm{of}\;\mathrm{Death}\;\mathrm{Reported}\;\mathrm{by}\;\mathrm{GBD}\;}$
**Availability of timely VS data**	NA	*Exponential smoothing on the combination of the previous indicators*

#### Quality of cause of death reporting

Misassignment of the underlying cause of death was selected as the indicator to describe the quality of cause of death reporting. Building on the work of Naghavi and colleagues [24], all data were systematically examined for records which were not coded to underlying causes of death, but rather coded to intermediate, immediate, unspecified, or otherwise inapplicable causes of death (collectively termed “garbage codes”). Closer examination reveals that most VS in fact contain a mixture of garbage codes, some being somewhat more meaningful than others. For example, deaths coded to the International Classification of Disease (ICD) code R99 – “other ill-defined and unspecified causes of mortality” – are effectively useless for public policy, whereas deaths coded to more specific, yet still vague codes such as C76 – “malignant neoplasm of other and ill-defined sites” – are more informative, despite being imprecise [25]. We classified garbage codes into two subcategories: those codes that do not contain any inherent information about the underlying cause of death (type 1), and those which do (type 2). There are several different categories of type 2 garbage codes; without any theoretical basis to weight each one separately, we arbitrarily assumed that type 2 garbage codes were, on average, 50% more informative than type 1. A full list of garbage codes can be found in the GBD supplementary material, and a table displaying this categorization for ICD-10 at the three-digit level can be found in Additional file 1: Table S2 [22].

For each country year (*cy*), we quantified the effective proportion of garbage codes as the sum of type 1 (i.e., uninformative garbage codes) and 50% of the amount of type 2 garbage codes, divided by the total number of deaths reported for that country-year:

$$\begin{array}{l} \mathrm{Adjusted}\;\mathrm{Garbage}\;{\mathrm{Proportion}}_{\mathrm{cy}}\\ \;=\frac{\mathrm{Garbage}\;\mathrm{Type}\;1_{\mathrm{cy}}+\;\left(0.5*\mathrm{Garbage}\;\mathrm{Type}\;2_{\mathrm{cy}}\right)}{\mathrm{All}\;\mathrm{Death}s_{\mathrm{cy}}} \end{array}$$

Where *Garbage Type* 1_*cy*_ and *Garbage Type* 2_*cy*_ are counts of deaths assigned to garbage codes in each category, and *All deaths*_*cy*_ is the total count of observed deaths for a given country and year, including garbage codes.

#### Quality of age and sex reporting

The indicator used for this component was the number of deaths with either unspecified age or sex. This was simply calculated as the fraction of all deaths reported with either an unspecified age or sex, or both.

#### Internal consistency

To measure this dimension, we use an indicator of medically impossible cause of death assignments for any given age or sex. Drawing on both the medical literature and expert opinion, we developed a conservative list of causes of death that we believe are impossible at certain ages or for one sex. Examples of such medically impossible combinations are males diagnosed with cervical cancer or pregnancy-related mortality at ages less than 10 years. Additional file 1: Table S3 lists the specific age-sex combinations of causes deemed to be impossible. We then computed the fraction of deaths that this represented for each country-year.

#### Completeness of death reporting

We assessed this dimension as the percent of all expected deaths in a country-year that were actually observed. To assess completeness, we used the findings reported by Murray and colleagues [26] for the GBD study. They used established demographic techniques, including Generalized Growth Balance, Synthetic Extinct Generations, and a combination of the two to estimate the proportion of adult deaths that were reported in a civil registration system in each country-year [26]. Completeness of death registration for ages 5 and under was estimated by comparing under-5 mortality estimates to individual VS point estimates of under-5 mortality [21]. We computed a weighted average of these two age groups to produce a single indicator of completeness, weighted according to the estimated numbers of deaths in each broad age group.

#### Level of cause-specific detail

The indicator used for this component was the number of distinct causes of death reported in the VS for each country-year, divided by a reference standard number of distinct causes of death. We chose the list of causes of death (192 unique causes) developed for GBD as the standard, given the substantial public health inputs that had already been made to develop this list for global disease and injury assessment. We then quantified this indicator by computing the proportion of the 192 GBD-standard causes of death that were available for each country-year [22]. Exceptions were made for data that were reported using the ICD-8 A–List prior to 1981 or ICD-9 Basic Tabulation List prior to 1998, since these tabulation schemes had been recommended by WHO as a reporting standard [27]. We chose the years 1981 and 1998 to define when, respectively, ICD-8 and ICD-9 became unacceptable as the reporting standard based on the timing of the reporting lists used by all other countries in our dataset, allowing a two-year “grace period” after the initial adoption of these ICD standards.

#### Timeliness and availability of data

The final dimension of VS performance is simply the availability of VS data. This is conceptually different to the previous five dimensions, but we argue for its inclusion on the basis that even data of perfect quality are useless for public policy debates unless they are available. While sporadic availability of VS can be informative, we believe that the potential value of VS data in any given year, notwithstanding the five quality dimensions discussed above, is more than simply availability of data for that year, and encompasses the information content from VS for the immediately preceding years, appropriately weighted to reflect proximity to the most recent year. Some delay, however, is understandable for countries that assiduously apply data checking procedures and incorporate coroner follow-up. We thus applied an exponential smoothing algorithm to the combined values of the other five indicators (see next section) in order to incorporate a measure of a system’s performance to yield sustained VS output. Less weight was given to years further in the past to emphasize the important role of current VS data to approximate current epidemiologic patterns (see Additional file 2).

The concept of “timeliness” of data is effectively captured through the use of smoothing, since missing data for recent years will be more heavily penalized in the calculation, given the weighting system used, than missing data for earlier years. Our approach of acknowledging the “momentum” inherent in prior data availability, formalized through smoothing, thus allows us to calculate a score for this dimension for all calendar years, irrespective of whether or not VS were available for a given year(s).

### Summary index of VS performance and data quality

The six dimensions described above may contribute differently to overall VS data quality and utility. For example, Table 2 demonstrates the variation across selected causes of death in the propensity to report deaths with unspecified age or sex. Certain causes, such as collective violence or malaria, are systematically reported with a higher percentage of unspecified age or sex (5.1% and 1.1%, respectively, based on all ICD-10-coded observations in our database). For completeness, garbage coding, and the attribution of impossible causes of death, various causes are systematically more affected than others as well. The consequence of these systematic patterns is that overall VS quality depends on both the level of each dimension (e.g., proportion of deaths with unspecified age or sex) as well as the cause of death composition of the population that generated the VS. The utility of VS data as a function of the level of detail in a cause list also depends on the composition of causes of death in a given population. For example, if the two leading causes of death are reported in aggregate, the VS will describe the epidemiologic profile of the population from which they were generated less accurately than if the two smallest causes of death were reported in aggregate. These observations suggest that a simple combination (such as an arithmetic mean) of the dimensions of VS performance may not be appropriate.

**Table 2:** Proportion of ICD-10 deaths with age or sex unspecified by cause (leading 15 causes only)

**Cause**	**Percent with unspecified age or sex**
Collective violence	5.1%
Assault by other means	2.1%
Other neonatal conditions	1.7%
Assault by firearm	1.6%
Legal intervention	1.3%
Yellow fever	1.2%
Exposure to forces of nature	1.2%
Assault by sharp object	1.1%
Malaria	1.1%
Neural tube defects	1.1%
Typhoid and paratyphoid fevers	1.0%
Neonatal encephalopathy (birth asphyxia and birth trauma)	0.9%
Pedestrian injury by road vehicle	0.9%
Syphilis	0.8%
Cholera	0.7%

### Simulation

To develop a summary index of VS system performance, we first designed a simulation procedure to estimate how accurately the data describe the cause of death patterns in a population, given the levels of each indicator. Each dimension (except availability/timeliness) was simulated at progressively worse states (while holding the four other dimensions constant), and the extent to which the simulated epidemiologic profile differed from the actual epidemiologic profile of a reference population was calculated, as described below.

The similarity between cause-specific mortality fractions (CSMFs) from a particular simulated case and the reference CSMFs was measured using a metric termed *CSMF Accuracy. CSMF Accuracy* measures the overall absolute difference between predicted and “true” CSMFs as a fraction of the theoretically largest possible absolute difference between them and is computed as follows:

$$\mathrm{CSMF}\;\mathrm{Accuracy}=1-\frac{\sum_{j=1}^{k}\mathrm{CSM}F_{J}^{\mathrm{true}}-\mathrm{CSM}F_{j}^{\mathrm{pred}}}{2\left[1-\mathrm{Minimum}\left(\mathrm{CSM}F_{j}^{\mathrm{true}}\right)\right]}$$

In terms of VS performance, *CSMF*^*pred*^ is the CSMF directly observed from a given VS dataset, and *CSMF*^*true*^ represents the hypothetically true CSMF for the same population for cause *j* out of a total number of *k* causes.

To define *CSMF*^*true*^ for our simulation, we utilized the CSMFs from GBD, estimated for seven broad epidemiologic regions, by year, sex and age [25]. Stochastic variation was introduced to the GBD CSMFs, which were modeled to only represent systematic epidemiological trends. This was done by comparing GBD CSMFs to observed VS CSMFs for all available ICD-10-coded country-years, and computing the square root of the mean squared error (RMSE) between the two sets of CSMFs, by cause, age, and sex. Normally distributed disturbances were generated with mean zero and standard deviation set to cause-age-sex-specific RMSE and added to the GBD estimates.

**Figure 1:**
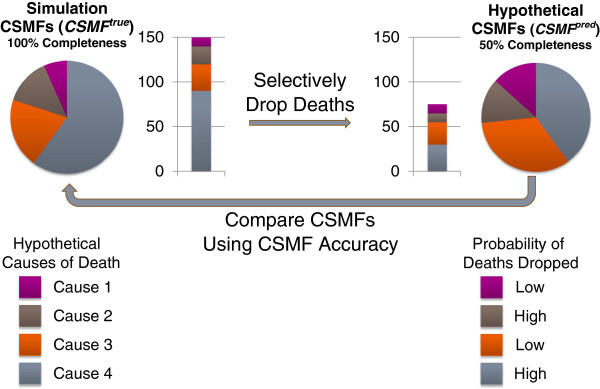
Simulation procedure for four hypothetical causes of death.

*CSMF*^*pred*^ in our simulation represents a hypothetical set of CSMFs which have been distorted due to a suboptimal level of one of the five (excluding availability/timeliness) dimensions of VS performance. These CSMFs were simulated by selectively removing deaths from the reference CSMFs *(CSMF*^*true*^*)*, and then recomputing CSMFs to define *CSMF*^*pred*^. Deaths were removed according to the empirical cause-specific probabilities derived from observed data for all VS country-years coded to ICD-10. For example, the proportion of each cause that was reported with either age or sex unspecified (Table 2) was used to selectively (with respect to the values in Table 2) remove deaths from the reference CSMFs when simulating suboptimal levels of age and sex reporting. Hypothetical CSMFs were simulated by manipulating one dimension at a time, holding the other four constant at their optimal level (either zero or one, depending on the dimension). Figure 1 provides a simplified diagram of this procedure, and Additional file 1: Table S5 demonstrates this procedure numerically.

**Figure 2:**
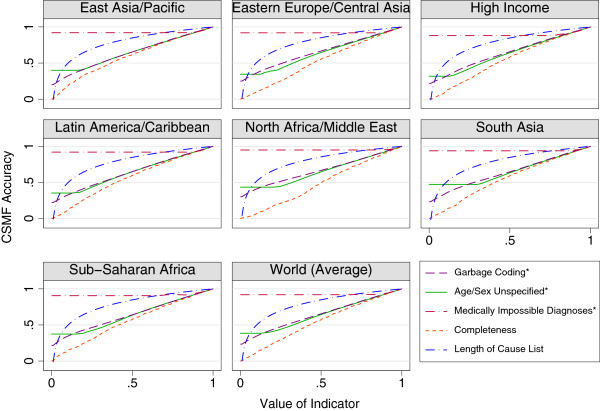
**Simulated CSMF accuracy associated with each indicator by region.** *Subtracted from one so that higher values are preferable to lower, as with other indicators.

Hypothetical sets of CSMFs were computed at progressively worse levels of a given dimension, from zero (e.g., no unspecified ages or sexes) to one (e.g., all deaths reported without age or sex) in increments of 0.01. At each simulated level, *CSMF Accuracy* was computed based on *CSMF*^*pred*^ and *CSMF*^*true*^. This procedure was repeated for each dimension separately. Due to the instability of CSMFs based on small death counts, *CSMF Accuracy* was observed to actually increase as a consequence of a worse level of a given dimension at extreme values. In such cases, the minimum *CSMF Accuracy* from higher levels of the same dimension was imposed to restrict *CSMF Accuracy* estimates from paradoxically increasing. Figure 2 displays the *CSMF Accuracy* associated with varying levels of each indicator for each of the seven GBD regions as well as the global mean of all regions.

### Computation of the vital statistics performance index

Simulation results were applied to the input database (described earlier) by mapping region-specific *CSMF Accuracy* estimates obtained through simulation to observed levels of each dimension of VS performance (see Additional file 1: Table S4). For example, a garbage-coding level of 20% (i.e., 80% of deaths coded to nongarbage) produced CSMFs that were 87% accurate in simulation. From this we conclude that any observed VS country-year which had 20% of deaths coded to garbage codes was in fact 87% accurate in measuring the actual epidemiological pattern of causes of death in that population. In other words, there is not a straight one-to-one mapping between data accuracy (i.e., correct specification of the CSMFs in a population) and data quality (extent of garbage coding in that population). Continuing this example, the indicator for impossible cause of death assignments was found to produce a simulated *CSMF Accuracy* of 92% when 20% of deaths were impossibly coded. Thus if a given country in a particular year had 20% of deaths assigned to garbage codes and 20% of deaths coded to impossible causes of death, then these values would map to 87% accuracy as a consequence of poor cause of death reporting, and 92% accuracy based on observed internal consistency of the data. This mapping was performed for all levels of all five indicators with special exceptions made for observations with no cause of death reporting (see discussion). This produced transformed, or accuracy-weighted, values of the observed indicators of VS performance for all country-years with available VS. Additional file 1: Table S4 provides the transformed values for each indicator, at each level of simulation, from 1% to 100% globally, although the actual simulation was performed by epidemiologic region. These values provide evidence of the differential impact of different levels of performance in each of these dimensions on the overall accuracy and utility of VS data.

Using the transformed (weighted) values for each indicator, we created a composite index from the five cause-specific VS performance dimensions as the product of their associated *CSMF Accuracy* values, as follows:

$$\begin{array}{l} \;\mathrm{VS}\;\mathrm{Performance}\;\mathrm{Inde}x_{\mathrm{cy}}=\\ \prod \left[\begin{array}{c} \left(\mathrm{Transformed}\;\mathrm{Adjusted}\;\mathrm{non}-\mathrm{Garbage}\;\mathrm{Proportio}n_{\mathrm{cy}}\right)\\ \left(\mathrm{Transformed}\;\mathrm{Proportion}\;\mathrm{of}\;\mathrm{Deaths}\;\mathrm{with}\;\mathrm{Known}\;\mathrm{Age}\;\mathrm{or}\;\mathrm{Se}x_{\mathrm{cy}}\right)\\ \left(\mathrm{Transformed}\;\mathrm{Proportion}\;\mathrm{of}\;\mathrm{Deaths}\;\mathrm{which}\;\mathrm{are}\;\mathrm{not}\;\mathrm{Medically}\;\mathrm{Impossibl}e_{\mathrm{cy}}\right)\\ \left(\mathrm{Transformed}\;\mathrm{Completenes}s_{\mathrm{cy}}\right)\\ \left(\mathrm{Transformed}\;\mathrm{Cause}\;\mathrm{List}\;\mathrm{Lengt}h_{\mathrm{cy}}\right) \end{array}\;\right] \end{array}$$

In other words, if the hypothetical country from the above example had 100% completeness, no deaths with unspecified age or sex (100% known age or sex) and 100% of the reference cause list available, its VSPI in that year would be the product of 0.87 and 0.92, or 0.80 (these being the values of *CSMF Accuracy* associated with poor cause of death reporting and poor internal consistency, respectively, in the above example). In other words, as a result of suboptimal cause of death reporting, this country’s VS are only 87% representative of what might be considered perfectly representative data. Because this example country also has suboptimal internal consistency, the VS are even less representative, reducing their accuracy from 87% to 80% (0.87 × 0.92).

The final dimension of VS performance is the availability of timely VS data. Because of the unique, multiyear implications of this component, as described earlier, it would be inappropriate to simply include it as a sixth indicator in the formula above since the representativeness and usefulness of VS is dependent on the existence of data, not only for the current year, but for previous years as well. We thus applied an exponential smoothing algorithm to values of the VSPI, counting all years without data as zero. To continue with the previous example (where the hypothetical country had a VSPI of 0.80 for a given year) suppose that the VSPI for all previous years had a value of 0.70. In this case, the present year’s final VSPI would be between 0.80 and 0.70 as a consequence of previous years with VS of poorer accuracy. For the exact specification of the smoothing algorithm, refer to the Additional file 2. In effect, this application makes the current value of the index most dependent on the quality of the present year’s VS, but it also requires that a VS system has consistently produced data in the recent past in order for it to be considered as well-performing.

## Results

### Simulation results

The results from the simulation define the relative importance of the five dimensions for determining the representativeness of VS in a single year. Figure 2 illustrates the relationship between each indicator and *CSMF Accuracy*, based on simulation, and Additional file 1: Table S4 shows the exact values of *CSMF Accuracy* for each indicator. *CSMF Accuracy* declined more steeply as a consequence of declining completeness than for any other indicator, meaning that if all indicators were equal, the level of completeness would exert the largest impact on the final VSPI. The level of cause specific detail was found to have a highly concave relationship with *CSMF Accuracy*, displaying only modest declines in accuracy at higher values, with declines gradually becoming more severe as the indicator approached zero. The indicators representing quality of cause of death reporting and age and sex reporting (garbage coding and unspecified age or sex, respectively) displayed similar *CSMF Accuracy* trends to one another. Both indicators implied a generally linear decrease in *CSMF Accuracy* as the indicator increased, with the important distinction of a different minimum, or point above which accuracy does not decrease any further. This was at a lower point for the indicator of unspecified age or sex than for garbage coding. Interestingly, these two indicators both had nonzero intercepts with the y-axis, indicating that even when both were at their worst possible levels, simulated VS still yielded some information content about the cause of death structure in the population. Finally, medically impossible diagnoses, the indicator describing internal consistency of the data, also reached a point beyond which *CSMF Accuracy* did not decline. This threshold was reached at a much lower level of this indicator than the unspecified age or sex indicator, however, owing to the limited number of causes which could potentially be impossibly coded based on age and sex.

### Vital statistics performance index

**Figure 3:**
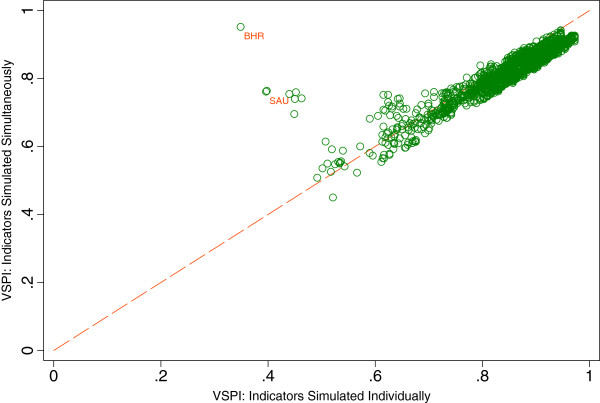
**VS performance index.** (a) Most recent year with data available (post-2005) (b) 2012.

The estimated VSPI for each country for the most recent year with data available (post 2005) is illustrated for broad categories in Figure 3a, which captures the data availability component of the index, but not timeliness of data, and in Figure 3b, which does. Specific values of the VSPI for countries, ranked according to the value of the index for their latest available data year, are given in Table 3. The mean VSPI since 2005 was 0.61, with a standard deviation of 0.31 (N = 133). Developed countries generally had higher VSPI values than developing countries, although a number of interesting departures from this generalization can be observed. For example Cuba, Costa Rica, Mexico, and Venezuela all demonstrate well-performing VS systems, whereas Switzerland, due to data being reported in recent years using a tabulation cause list rather than for detailed ICD codes, has low VS performance relative to its neighbors.

**Table 3:** VS Performance Index by country/territory, most recent year available (post-2005)

**Country**	**Year**	**VS performance index**
Hungary	2012	95.7
Finland	2011	95.6
Estonia	2012	94.6
New Zealand	2009	94.4
Lithuania	2010	93.8
Moldova	2012	93.4
Australia	2011	92.1
Malta	2011	91.5
United Kingdom	2012	91.5
Austria	2011	91.3
Venezuela	2009	91.3
Iceland	2009	91.2
Slovakia	2010	91.2
Canada	2009	91.1
Cuba	2010	91.1
Latvia	2012	90.9
United States	2010	90.9
Chile	2009	90.8
Czech Republic	2012	90.5
Slovenia	2010	90.5
Costa Rica	2011	90.2
Germany	2012	90.0
Sweden	2010	89.4
Croatia	2012	89.3
Trinidad and Tobago	2008	89.3
Ireland	2010	88.9
Serbia	2012	88.9
Israel	2011	88.8
Mexico	2012	88.5
Netherlands	2011	88.5
Spain	2011	88.3
Bahamas	2008	87.8
Denmark	2011	87.8
Norway	2012	87.6
Japan	2011	87.0
Romania	2011	87.0
Mauritius	2011	86.9
Belize	2009	86.8
France	2010	86.7
South Korea	2011	86.6
Luxembourg	2011	86.6
Kyrgyzstan	2010	86.3
Kuwait	2011	85.9
Bermuda	2008	85.8
Puerto Rico	2010	85.2
Belgium	2010	84.9
Poland	2011	84.6
Saint Vincent and the Grenadines	2010	84.3
Antigua and Barbuda	2009	83.0
Dominica	2010	82.9
Bulgaria	2012	82.8
Panama	2009	82.7
Brazil	2010	82.6
Colombia	2008	82.5
Cyprus	2011	82.0
Hong Kong Special Administrative Region of China	2011	82.0
Grenada	2010	81.9
Barbados	2008	81.1
Macedonia, FYR	2010	80.9
Portugal	2011	80.4
Argentina	2010	80.0
Italy	2010	79.1
Nicaragua	2011	78.3
Guatemala	2009	75.6
Malaysia	2008	75.5
El Salvador	2009	74.3
Taiwan	2012	73.2
Egypt	2011	73.0
South Africa	2009	72.6
Russia	2012	71.6
Guyana	2009	70.6
Singapore	2011	70.5†
Greece	2011	69.4
Suriname	2009	69.1
Jamaica	2006	68.8
Paraguay	2010	65.7
Saint Lucia	2008	64.0
Ecuador	2010	63.4
Qatar	2011	63.1
Uruguay	2009	61.7
Bahrain	2009	61.5
Peru	2010	61.0
Thailand	2007	57.2
Iran	2010	55.7
Jordan	2010	54.1
Armenia	2012	53.1
Uzbekistan	2005	53.0
Maldives	2011	52.5
Switzerland	2010	51.6
Georgia	2010	51.4
Turkey	2012	50.6
Dominican Republic	2010	50.2
Ukraine	2011	49.8
Seychelles	2009	47.6
Occupied Palestinian Territory	2009	47.5
Saudi Arabia	2012	45.7
Kazakhstan	2010	44.4
Brunei Darussalam	2011	40.2
Philippines	2008	40.2
Belarus	2009	38.3
Oman	2010	38.1
Sri Lanka	2006	35.6
China	2012	34.2
Montenegro	2009	31.8
Syria	2007	30.2†
Tajikistan	2005	30.0
Fiji	2011	29.6
Azerbaijan	2007	29.3
Algeria	2007	27.6
Bosnia and Herzegovina	2011	23.2
Bolivia	2006	21.7†
Kiribati	2005	18.3*
Mongolia	2010	15.1†
Morocco	2011	12.1
Iraq	2008	11.9
Botswana	2008	8.9†
Andorra	2010	8.4*
Albania	2010	7.7†
Turkmenistan	2006	7.6*
Macao Special Administrative Region of China	2010	7.4*
Bhutan	2009	6.1
India	2008	4.3
Zimbabwe	2007	4.1
Marshall Islands	2006	3.0*
Libya	2008	2.1
Myanmar	2005	1.8*
Ghana	2007	1.4
Gabon	2006	1.3†
Malawi	2007	0.4†
Pakistan	2009	0.1†
Kenya	2005	0.0†
Nigeria	2007	0.0†
Tanzania	2010	0.0†

*All-causes only.

†Index computed without garbage.

The following countries had no data available in 2005–2012: Afghanistan, Angola, Bangladesh, Benin, Burkina Faso, Burundi, Cambodia, Cameroon, Cape Verde, Central African Republic, Chad, Comoros, Congo, Congo, the Democratic Republic of the, Cote d’Ivoire, Djibouti, Equatorial Guinea, Eritrea, Ethiopia, Gambia, Guinea, Guinea-Bissau, Haiti, Honduras, Indonesia, Laos, Lebanon, Lesotho, Liberia, Madagascar, Mali, Mauritania, Micronesia, Federated States of, Mozambique, Namibia, Nepal, Niger, North Korea, Papua New Guinea, Rwanda, Samoa, Sao Tome and Principe, Senegal, Sierra Leone, Solomon Islands, Somalia, Sudan, Swaziland, Timor-Leste, Togo, Tonga, Tunisia, Uganda, United Arab Emirates, Vanuatu, Vietnam, Yemen, Zambia.

Among high-income countries, Finland, New Zealand, Australia, and the United Kingdom had the highest-performing VS systems, with index values of 0.957, 0.944, 0.921, and 0.915, respectively. Six other countries from the high-income GBD region achieved VSPIs greater than 0.90 in their most recent available year, although most countries (27) did not have VS available for 2012. The high-income region scored the highest average VSPI of 0.814 (standard deviation 0.179, N = 33). Considerable heterogeneity is apparent within the Eastern Europe/Central Asia region, with Hungary, Moldova, Lithuania, and Estonia achieving values above or equal to 0.93 since 2010, meaning that their VS were at least 93% representative of the epidemiological situation of the country. Four additional countries in this region had indices greater than 0.90, yet the region overall had an average VSPI of 0.637 (standard deviation 0.301, N = 29), and 19 countries did not have VS available in 2012. Similar variability in VSPIs was found in the East Asia/Pacific region, which ranged from 0.820 in Hong Kong to 0.018 in Myanmar, with an average of 0.393 for the region (standard deviation 0.275, N = 13). The second-highest average index for a region was in Latin America and the Caribbean, which averaged 0.765 (standard deviation 0.148, N = 29) and ranged from 0.217 in Bolivia to 0.913 in Venezuela, indicating VS in Bolivia are only 21.7% representative of the likely Bolivian cause of death composition, compared with 91.3% representative in Venezuela. North Africa and the Middle East had an average index of 0.439 (standard deviation 0.237, N = 15), and sub-Saharan Africa averaged 0.203 (standard deviation 0.327, N = 11) with a notably high index (0.869) in Mauritius. However, this average was based on very few reporting countries with only 22.9% of all countries in sub-Saharan Africa providing data after 2005, as compared to 70% for the world, and 100% for the high-income and Eastern Europe/Central Asia regions. Finally, the South Asia region had the fewest number of countries (3) with available data in this time period, all of which had a very low VSPI: 0.061 in Bhutan, 0.043 in India, and 0.001 in Pakistan, based on data publicly available.A major factor affecting the utility of vital registration data is how timely they are. Arguably, the value of vital statistics in informing public policy debates will be greater the more current they are. Figure 3b shows VSPI scores for countries in 2012, the most recent year for which mortality statistics can be expected to be available (allowing an 18 month processing period). A comparison with Figure 3a is illuminating. Countries such as Hungary, the United Kingdom, Moldova and Estonia are likely to derive greatest benefit from their vital statistics because they are up-to-date. Conversely, several countries, including Canada, New Zealand, Venezuela and Chile, which scored highly when only data availability and quality are taken into account (Figure 3a), show much worse performance when comparing index scores in 2012. Indeed, CRVS performance in these four countries drops from the top category when timeliness is not taken into account, to the second bottom category when it is (Figure 3b). In this sense, Figure 3b shows the importance of recent data for providing information about the current epidemiological conditions in a country. In this sense, Figure 3b shows the importance of recent data for providing information about the current epidemiological conditions in a country. Reporting delays in most countries around the world greatly reduce the current VS performance index.

### Contributions of performance dimensions

As expected, the five simulated indicators contributed differentially to observed VSPI values worldwide. Table 4 shows the amount of variation (measured as the marginal sum of squares and correlation coefficients) in the VSPI that can be explained by each component. Considering both the weight obtained through simulation and the variation in the actual data, the indicators of completeness and cause-specific detail were the strongest drivers of observed VSPI values, each explaining a substantial amount of variation (marginal sums of squares: 30.49 and 36.73; correlation coefficients: 0.71 and 0.62, respectively). The garbage coding indicator also had a strong impact on observed VSPI values, with a marginal sum of squares of 12.02 and correlation with the overall VSPI of 0.26, indicating that quality of cause of death reporting was also a very important dimension for VS quality. The indicator measuring impossible diagnoses explained more variance in the VSPI (18.58) but only did so for a few observations and had a relatively low (0.11) correlation with VSPI scores. Finally, the unspecified age or sex indicator explained the least amount of variation, with a very low marginal sum of squares (1.56), indicating that most countries’ VS system were performing similarly on this dimension.

**Table 4:** ANOVA results and correlation between VS performance index and indicators

	**Correlation with VSPI**	**Marginal sum of squares**
**Completeness**	0.71	30.49
**Cause list length**	0.62	36.73
**Garbage**	0.26	12.02
**Age/sex unspecified**	0.25	1.56
**Impossible diagnoses**	0.11	18.58

### Progress in strengthening vital statistics

**Figure 4:**
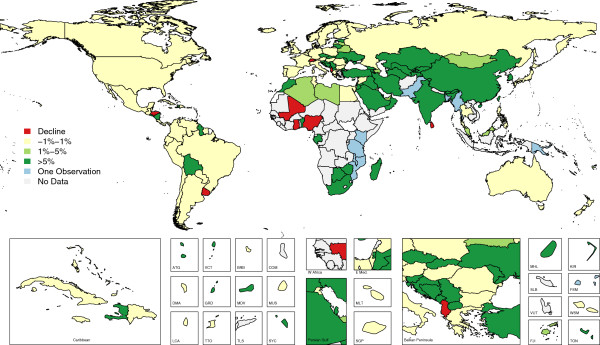
Average annualized rate of change in VS Performance Index from earliest to latest available observation (1980–2012).

Given the current global focus on strengthening VS systems, it is of interest to examine how VS performance in countries has changed over time. From the first year with available data (from each country included in analysis) to 2012, VS performance has modestly increased at an average annual rate of 0.36% per year, excluding abnormally high rates of change due to near-zero starting or ending values. Narrowing this time frame to include only years in which data are available (to avoid penalizing a country for a reporting delay), this rate of change increases to 2.10% per year, indicating that many countries have not reported data for more recent years, despite the policy value in doing so. A number of countries (27) were able to achieve improvements in VS performance greater than 5% per year (over the years for which data were available), as shown in Figure 4. Eight countries improved at modest rates between 1% and 5%, while 62, mostly high-income, countries demonstrated essentially stagnant VS performance (between −1% and 1% per year). In five countries, the VSPI actually declined at rates between 1% and 3% per year. Additional file 1: Figure S1 displays the entire time series for each country, including the five dimensions.

At the global level, a more realistic appraisal of progress with VS systems would need to include countries that haven’t reported VS data and also to take into account the fraction of global mortality that each country represented in each year. When we do this, we find that only 19.0% of the world’s deaths between 2000 and 2012 occurred in countries that have well-performing VS systems (VSPI values greater than or equal to 0.8). This is barely an improvement from the same percentage in the 1990s, 18.9%, as well as the 1980s, 18.1%. Conversely, 67.4% of global mortality occurred in countries with poorly performing (lower than 0.5) VS systems post-2000, an improvement from 69.8% in the previous decade, and 72.7% in the 1980s. Thus while we might conclude that there has been improvement in CRVS systems over the past 30 years, it has been disappointingly slow, with the result that approximately two-thirds of global mortality currently is either poorly measured or entirely missed by VS systems in countries.

## Discussion

As with any analytic framework, a number of assumptions and operational choices were required to be made in order to create the VSPI in a manner which balances parsimony and interpretability with conceptual and methodological rigor. It is important to note, however, that the exact purpose of the simulation procedure developed here was to reduce the number of assumptions that otherwise would have been required, particularly about weighting indicators, in order to specify the relative importance of the various domains. We discuss below some of the more important implications of these choices.

### Potential methodological limitations in computing the index

**Figure 5:**
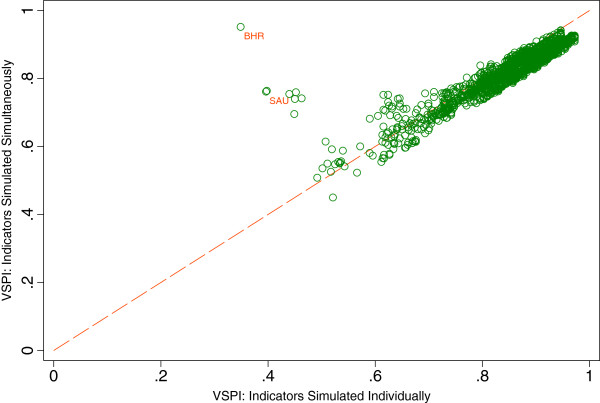
**Multiplicative model choice compared to alternative.** Note: Bahrain and Saudi Arabia stand out because CSMF Accuracy on the x−axis has been restricted to decrease or remain constant as simulated indicators decrease; this is not possible for the y−axis.

One methodological choice was the reliance on a multiplicative model for combining indicators. A possible alternative option would have been to simulate the accuracy associated with differing levels of all indicators simultaneously in order to avoid such a model choice altogether. This was found to be prohibitively difficult to implement due to the fact that the cause list length indicator was the only dimension simulated with a hierarchically organized cause list. Barring country-years with suboptimal cause list lengths, we tested this alternative, “simultaneous-simulation” model, and found very similar results to the “multiplicative model” (Figure 5). Additionally, Figure 5 demonstrates that simulating each indicator individually has an advantage over simulating all indicators simultaneously, namely that *CSMF Accuracy* can be constrained to continually decline with worse levels of each indicator, as mentioned earlier. Two countries in this figure, Saudi Arabia and Bahrain, would have unrealistically high VSPI values if a “simultaneous simulation” model were to be adopted, due to the chance possibility that *CSMF Accuracy* could increase when based on a smaller number of deaths.

There may also be dependency between indicators such as between garbage codes and cause list length: VS reported with a short cause list will be likely to have a small garbage proportion as a result. Gabon, for example, has a garbage percentage of exactly zero from 2001 to 2006. On closer inspection, it is clear that this is simply because only a small handful of aggregated causes of death were reported in these years. Because of the high degree of weight put on the garbage indicator relative to the cause list length indicator, Gabon would rank among the best countries in its region for VS performance, a conclusion that many would find surprising. We decided that the most elegant solution to this problem would be to simply compute the VSPI without the garbage indicator for cases where this was required, allowing an additional one-fourth weight for each other indicator. This exception affected approximately 3.5% (123) of all country-years, which were selected on the basis of the following criteria: a) garbage percentage for a given country-year ranked above the 90^th^ percentile of all garbage percentages in the database and b) the cause list presented in the country-year was shorter than the reference standard cause list.

Approximately 17% of all country-years available for analysis reported data without causes of death, yet three of the six components pertain to causes of death. The solution posed in the previous paragraph, namely simply estimating the index without the missing indicators, does not apply in these cases, however; VS without cause of death information are likely to be of less value than those with poorly-coded causes of death. Hence, some value of CSMF accuracy that equates to zero cause of death reporting is needed. Theoretically, the minimum CSMF accuracy associated with the cause of death quality dimension represents this value because it reflects the accuracy of VS if 100% of all deaths were coded to garbage. We therefore used this minimum CSMF accuracy value in place of the cause-specific indicators of garbage and cause list length for all country-years that reported all causes in aggregate.

### Availability and timeliness

Although the GBD database involved a substantial data collection effort, countries may have produced VS for various years which were either not made publicly available or were otherwise not included. Our analysis cannot distinguish between reasons for missing VS in the GBD database. As such, the VSPI might underestimate the actual amount of data that a country has available and hence underestimate the performance of their VS system. This dimension may also lead to a potentially misleading interpretation about the quality of recent VS data in a country which accomplished a rapid improvement in a short period of time. We believe that data availability and timeliness are critical to the utility of VS for public policy because to truly understand the policy implications of the current epidemiologic description of a population, a consistent time series is necessary. Hence we included availability as one of the six components of the index by use of exponential smoothing. By doing so, the VSPI provides a more comprehensive view of the performance of a VS system rather than assessing VS data at a single point in time. Indeed, given the impact of this dimension on the overall VSPI, improving VS performance in some countries may be less a matter of strengthening data collection procedures and more a matter of ensuring that data are routinely compiled and made available in a timely fashion.

### Alternative indicators

Another consideration in our analysis is the choice of which indicators to use to describe performance. Another potential indicator of quality of cause of death reporting is the extent of misclassification, whereby certain causes of death are incorrectly and differentially diagnosed as other nongarbage codes. This phenomenon has been documented, for example, in South Africa, where HIV/AIDS mortality was drastically underreported and AIDS-related conditions consequently overreported [28]. Although studies such as this have been conducted to examine misclassification, none (to our knowledge) have examined an exhaustive list of causes of death, or at least none have done so in a manner which was generalizable to the national level, let alone across countries [29-35]. Without quantification of the direction and magnitude of misclassification amongst all causes of death, incorporating this as an indicator was beyond the scope of this analysis. Also, misclassification and garbage coding both serve to describe the more general concept of cause of death reporting. These indicators have been observed to be highly correlated; hence garbage coding may be a sufficient indicator of overall cause of death reporting accuracy alone [30]. Furthermore, including multiple separate indicators of a single dimension would skew the final index to favor that component, due to its redundant appearance in the final formula.

A similar argument may be made for including an indicator of age misclassification, as well as age group aggregation (i.e., the use of 10-year, 15-year, or larger age groups in tabulation rather than the standard five-year age groups). Although aggregation is a readily quantifiable indicator, the counterarguments presented above against the inclusion of misclassification are equally valid in both cases. Unspecified age and sex reporting should act as a suitable summary indicator of general quality of age reporting and redundant indicators should be avoided.

VS coverage has been used as a complement to completeness in other analyses [19]. The choice to use completeness alone was made given the methodologically rigorous series of completeness estimates produced for GBD, and the lack of comparable information on coverage [21]. In general, and as limited sensitivity analyses have shown, the nuances of computing indicators to represent dimensions of VS performance are seemingly less consequential than the inclusion of exclusion of the dimensions themselves, as well as the development of a meaningful combination of these components.

### Other potential methodological limitations

There are a number of other potential limitations to this analysis, including the arbitrary adjustment of garbage codes, the relevance of *CSMF Accuracy*, the smoothing component over time, the noise (stochasticity) generation process in defining the simulation dataset, the representativeness of simulation proportions, and other nuances specific to the simulation for each indicator. We discuss these more technical issues further in the Additional file 2.

## Conclusions

There are clearly many choices and assumptions that must be made to construct a summary index of VS performance. The index we have proposed in this paper measures the performance of the output of death registration systems, gives insight into the relative contributions of five dimensions to overall performance of a VS system, and does so in an objective fashion. As the limitations of this study imply, summary indicators such as this do have a tendency to obscure nuances within VS performance, and practical application of the VSPI must entail examination of its subcomponents. This approach is not intended to entirely replace local expert opinion about VS system performance, but instead complement it, especially for monitoring change.

The findings from this study show that, all else being equal, completeness of a VS system is the most important aspect affecting performance, with similarly large contributions arising from the use of abbreviated cause of death lists to report data and widespread use of garbage codes to assign the underlying cause of death. Given the dominance of these dimensions, national strategies to rapidly strengthen VS systems would do well to focus on them.

Among the countries which have measurable VS systems, VS performance appears to be improving steadily. While this is encouraging, global efforts to improve VS systems have been less impressive; one-third of the world’s countries still do not report mortality data, because their systems are too rudimentary to compile data, only cover some urban areas, or particularly because the majority of people die outside health facilities and are not registered. Accounting for the size of the populations not served by VS systems, it is apparent that a great deal of room for improvement remains globally. The regular application of the VSPI to a global database will show where improvement is most needed and where encouraging progress has taken place. Such evidence is crucial for guiding global investments in strengthening vital statistics in countries.

As the emphasis on empirically based priority setting, monitoring global public health, and the evaluation of health programs continues to grow, so will the demand for reliable, timely, and comprehensive health information, particularly VS. Summary metrics such as the *Vital Statistics Performance Index* should greatly assist countries and the global public health community to understand in a comparable fashion how well these critical data systems are performing and help focus strategies designed to make them more fit for purpose.

## Abbreviations

CoD: Causes of death; VS: Vital statistics; GBD: Global burden of disease; ICD: International Classification of Disease; CSMF: Cause-specific mortality fraction; RMSE: Root mean squared error.

## Competing interests

The authors declare that they have no competing interests.

## Authors’ contributions

DP, MN, and ADL conceptualized the study. DP wrote the first draft. DP and DG-M helped develop the database and designed and implemented the analysis. CA helped develop the database. ADL, CJLM, RL, and MN oversaw the development of the database, helped design the analysis, and provided expert opinion. LM contributed to drafts and provided expert advice on civil registration and vital statistics. DP accepts full responsibility for the work and the conduct of the study, had access to the data, and controlled the decision to publish. All authors reviewed the draft and results. All authors read and approved the final manuscript.

## Supplementary Material

Additional file 1: Table S1 Country-years with data available. **Table S2.** Categorization of Garbage Codes. **Table S3.** Combinations of Age or Sex with Cause of Death Deemed Impossible. **Table S4.** Proportion of ICD-10 Deaths Coded to Any Garbage Code by Cause (Leading 15 CSMFs Only). **Figure S1.** VS Performance Index and Indicators by Country and Time Period. **Figure S2.** Effect of Smoothing. Click here for file

Additional file 2: Text 1 Methods – Simulation. **Text 2.** Methods – Smoothing. **Text 3.** Discussion – Timeliness and Availability. **Text 4.** Discussion – Potential Methodological Limitations. Click here for file

## References

[B1] Dow U Birth Registration: The “First” Right. The Progress of Nations 1998 New York, USA: UNICEF 5 11

[B2] AbouZahr C Boerma T Health information systems: the foundations of public health Bull World Health Organ 2005 83 8 578 583 16184276 PMC2626318

[B3] PARIS21 Secretariat A Guide to Designing a National Strategy for the Development of Statistics (NSDS) 2004 Paris, France: PARIS21

[B4] World Health Organization Sixtieth World Health Assembly. WHA60.27 Strengthening of Health Information Systems 2007 Geneva, Switzerland: WHO

[B5] Mikkelsen L Lopez AD Improving the Quality and Use of Birth, Death and Cause-of-Death Information: Guidance for a Standards-Based Review of Country Practices [Internet] 2010 Health Information Systems Knowledge Hub, School of Population Health, University of Queensland and World Health Organization [cited 2013 Aug 27]. Available from: http://www.uq.edu.au/hishub/docs/WP01/WP_01.pdf

[B6] World Health Organization, University of Queensland Health Information Systems Knowledge Hub Rapid Assessment of National Civil Registration and Vital Statistics Systems 2010 Geneva, Switzerland: WHO

[B7] Mikkelsen L Strategic Planning to Strengthen Civil Registration and Vital Statistics Systems: Guidance for Using Findings from a Comprehensive Assessment [Internet] 2012 Health Information Systems Knowledge Hub, School of Population Health, University of Queensland [cited 2013 Aug 27]. Available from: http://www.uq.edu.au/hishub/docs/WP23/HISHUB-WP%2023-02%20OCT.pdf

[B8] Lopez AD Mikkelsen L Rampatige R Upham S AbouZahr C Gamage S de Savigny D Schmider A Strengthening Civil Registration and Vital Statistics for Births, Deaths and Causes of Death: Resource Kit [Internet] 2013 Health Metrics Network and World Health Organization [cited 2013 Aug 27]. Available from: http://www.uq.edu.au/hishub/docs/Resource%20Kit/CRVS_ResourceKIt_active_content.pdf

[B9] International Monetary Fund The General Data Dissemination System: Guide for Participants and Users 2007 Washington D.C., USA: International Monetary Fund

[B10] PARIS21 Task Team on, Statistical Capacity Building Indicators The Framework for Determining Statistical Capacity Building Indicators 2002 Washington D.C., USA: International Monetary Fund

[B11] Health Metrics Network Strengthening Country Health Information Systems: Assessment and Monitoring Tool. Version 1.75 2006 Geneva, Switzerland: WHO

[B12] Aqil A Lippeveld T Hozumi D PRISM framework: a paradigm shift for designing, strengthening and evaluating routine health information systems Health Policy Plan 2009 24 3 217 228 10.1093/heapol/czp010 19304786 PMC2670976

[B13] World Health Organization Data Quality Assessment (DQA) Tool Version 1.0 [Internet] 2013 WHO [cited 2013 Jul 8]. Available from: http://www.who.int/entity/healthinfo/DQA_Tool.zip

[B14] World Health Organization The Immunization Data Quality Self-Assessment (DQS) Tool 2005 Geneva, Switzerland: WHO Department of Immunization, Vaccines and Biologicals

[B15] Jordan K Percheret M Croft P Quality of morbidity coding in general practice computerized medical records: a systematic review Fam Pract 2004 21 4 396 412 10.1093/fampra/cmh409 15249528

[B16] Brouwer H Bindels P Weert H Data quality improvement in general practice Fam Pract 2006 23 5 529 536 10.1093/fampra/cml040 16868006

[B17] Gourbin C Masuy-Stroobant G Registration of vital data: are live births and stillbirths comparable all over Europe? Bull World Health Organ 1995 73 4 449 460 7554016 PMC2486783

[B18] Mahapatra P Rao C Cause of death reporting systems in India: a performance analysis Natl Med J India 2001 14 3 154 162 11467144

[B19] Mathers CD Ma Fat D Inoue M Rao C Lopez AD Counting the dead and what they died from: an assessment of the global status of cause of death data Bull World Health Organ 2005 83 3 171 177 15798840 PMC2624200

[B20] Mahapatra P Shibuya K Lopez AD Coullare F Notzon FC Rao C Szreter S Civil registration systems and vital statistics: successes and missed opportunities Lancet 2007 370 9599 1653 1663 10.1016/S0140-6736(07)61308-7 18029006

[B21] Wang H Dwyer-Lindgren L Lofgren KT Rajaratnam JK Marcus JR Levin-Rector A Levitz CE Lopez AD Murray CJL Age-specific and sex-specific mortality in 187 countries, 1970–2010: a systematic analysis for the Global Burden of Disease Study 2010 Lancet 2012 380 9859 2071 2094 10.1016/S0140-6736(12)61719-X 23245603

[B22] Lozano R Naghavi M Foreman K Lim S Shibuya K Aboyans V Abraham J Adair T Aggarwal R Ahn SY AlMazroa MA Alvarado M Anderson HR Anderson LM Andrews KG Atkinson C Baddour LM Barker-Collo S Bartels DH Bell ML Benjamin EJ Bennett D Bhalla K Bikbov B Bin Abdulhak A Birbeck G Blyth F Bolliger I Boufous S Bucello C Global and regional mortality from 235 causes of death for 20 age groups in 1990 and 2010: a systematic analysis for the Global Burden of Disease Study 2010 Lancet 2012 380 9859 2095 2128 10.1016/S0140-6736(12)61728-0 23245604 PMC10790329

[B23] Murray CJ Ezzati M Flaxman AD Lim S Lozano R Michaud C Naghavi M Salomon JA Shibuya K Vos T Wikler D Lopez AD GBD 2010: design, definitions, and metrics Lancet 2012 380 9859 2063 2066 10.1016/S0140-6736(12)61899-6 23245602

[B24] Naghavi M Makela S Foreman K O’Brien J Pourmalek F Lozano R Algorithms for enhancing public health utility of national causes-of-death data Popul Health Metr 2010 8 9 doi:10.1186/1478-7954-8-9 10.1186/1478-7954-8-9 PMC2873308 20459720

[B25] World Health Organization ICD 10: International Statistical Classification of Diseases and Related Health Problems Volume 1. 10th Revision 1992 Geneva, Switzerland: World Health Organization

[B26] Murray CJL Rajaratnam JK Marcus J Laakso T Lopez AD What can we conclude from death registration? Improved methods for evaluating completeness PLoS Med 2010 7 4 e1000262 10.1371/journal.pmed.1000262 20405002 PMC2854130

[B27] World Health Organization International Classification of Diseases (ICD) [Internet] 2013 Geneva, Switzerland: World Health Organization [cited 2013 Sep 11]. Available from: http://www.who.int/classifications/icd/en/

[B28] Groenewald P Nannan N Bourne D Laubscher R Bradshaw D Identifying deaths from AIDS in South Africa AIDS 2005 19 2 Available from: http://journals.lww.com/aidsonline/Fulltext/2005/01280/Identifying_deaths_from_AIDS_in_South_Africa.12.aspx 10.1097/00002030-200501280-00012 15668545

[B29] Al-Samarrai T Madsen A Zimmerman R Maduro G Li W Greene C Impact of a Hospital-Level Intervention to reduce heart disease over reporting on leading causes of death Prev Chronic Dis 2013 10 E77 23680506 10.5888/pcd10.120210 PMC3667027

[B30] Rao C Yang G Hu J Ma J Xia W Lopez AD Validation of cause-of-death statistics in urban China Int J Epidemiol 2007 36 3 642 651 10.1093/ije/dym003 17329316

[B31] Kircher T Anderson RE Cause of Death. Proper completion of the death certificate J Am Med Assoc 1987 258 3 349 352 10.1001/jama.1987.03400030065033 3599328

[B32] Feuer EJ Merrill RM Hankey BF Cancer surveillance series: interpreting trends in prostate cancer—part II: cause of death misclassification and the recent rise and fall in prostate cancer mortality J Natl Cancer Inst 1999 91 12 1025 1032 10.1093/jnci/91.12.1025 10379965

[B33] Lloyd-Jones DM Martin DO Larson MG Levy D Accuracy of death certificates for coding coronary heart disease as the cause of death Ann Intern Med 1998 129 12 1020 1026 10.7326/0003-4819-129-12-199812150-00005 9867756

[B34] Jensen HH Godtfredsen NS Lange P Vestbo J Potential misclassification of causes of death from COPD Eur Respir J 2006 28 4 781 785 16807258 10.1183/09031936.06.00152205

[B35] Lahti R Penttilä A The validity of death certificates: routine validation of death certification and its effects on mortality statistics Forensic Sci Int 2001 115 1–2 15 32 11056267 10.1016/s0379-0738(00)00300-5

