# Supplementary material for: A composite metric for assessing data on mortality and causes of death: the vital statistics performance index
Source: Popul Health Metr. 2014 May 14;12:14. doi: 10.1186/1478-7954-12-14 (PMC4060759; doi:10.1186/1478-7954-12-14)
Supplement: Additional file 1: Table S1 — Country-years with data available. Table S2. Categorization of Garbage Codes. Table S3. Combinations of Age or Sex with Cause of Death Deemed Impossible. Table S4. Proportion of ICD-10 Deaths Coded to Any Garbage Code by Cause (Leading 15 CSMFs Only). Figure S1. VS Performance Index and Indicators by Country and Time Period. Figure S2. Effect of Smoothing. [file 1478-7954-12-14-S1.pdf]

Appendix Table 1. Country-years with data available.

Country	Years
Afghanistan	None Available
Albania	1987-2010
Algeria	1980-1982, 1985-1987, 1990, 1992, 1997-1998, 2000, 2003, 2005-2011
Andorra	1992, 1994, 2003-2005, 2007-2010
Angola	None Available
Antigua and Barbuda	1980, 1983, 1985-2009
Argentina	1980-2011
Armenia	1981-2012
Australia	1980-2011
Austria	1980-2011
Azerbaijan	1981-2010
Bahamas	1980-2008
Bahrain	1980-1982, 1984-1993, 1995-2009, 2012
Bangladesh	1996
Barbados	1980-1995, 2000-2008
Belarus	1980-2011
Belgium	1980-2010
Belize	1980-2009
Benin	1996-2000
Bermuda	1980-2009, 2011
Bhutan	2000-2009
Bolivia	1988, 2000-2003, 2006
Bosnia and Herzegovina	1981, 1985-1991, 1998-2011
Botswana	2005-2008
Brazil	1980-2011
Brunei Darussalam	1981-1992, 1995-2011
Bulgaria	1980-2012
Burkina Faso	None Available
Burundi	None Available
Cambodia	None Available
Cameroon	None Available
Canada	1980-2011
Cape Verde	1980, 1983-1985, 1990-1991, 1995-2000
Central African Republic	None Available
Chad	None Available
Chile	1980-2011
China	1991-2002, 2004-2012
Colombia	1980-2009, 2011
Comoros	None Available
Congo	None Available
Congo, the Democratic Republic of the	None Available
Costa Rica	1980-2011
Cote d'Ivoire	None Available
Croatia	1985-2012
Cuba	1980-2010
Cyprus	1980-2011
Czech Republic	1980-2012
Denmark	1980-2011
Djibouti	None Available
Dominica	1980-2010
Dominican Republic	1980-1992, 1994-2001, 2003-2010
Ecuador	1980-2010
Egypt	1980-1981, 1983-2011
El Salvador	1980-2009, 2011
Equatorial Guinea	None Available
Eritrea	None Available
Estonia	1980-2012
Ethiopia	None Available

Fiji	1980-1987, 1991, 1996-2004, 2009-2011
Finland	1980-2011
France	1980-2011
Gabon	2001-2006
Gambia	None Available
Georgia	1981-2010, 2012
Germany	1980-2012
Ghana	2000-2007
Greece	1980-2011
Grenada	1980, 1984-1986, 1988-2010
Guatemala	1980-1981, 1983-2009, 2011
Guinea	None Available
Guinea-Bissau	None Available
Guyana	1980, 1984-1985, 1988-1999, 2001-2009
Haiti	1980-1981, 1987, 1992, 1999, 2002-2004
Honduras	1980-1983, 1987-1990, 1994
Hong Kong Special Administrative Region of China	1980-2011
Hungary	1980-2012
Iceland	1980-2011
India	1980-1983, 1986-2006, 2008-2010
Indonesia	None Available
Iran	1980-1988, 1990-1993, 1995-2010
Iraq	1987-1989, 2008
Ireland	1980-2010
Israel	1980-2012
Italy	1980-2010
Jamaica	1980-1991, 1996, 1999-2006
Japan	1980-2011
Jordan	1980, 1986, 1991, 1999, 2004-2006, 2008-2010
Kazakhstan	1981-1982, 1984-2010
Kenya	2005
Kiribati	1990-2002, 2005
Kuwait	1980-1989, 1991-2011
Kyrgyzstan	1981-2011
Laos	None Available
Latvia	1980-2012
Lebanon	None Available
Lesotho	None Available
Liberia	None Available
Libya	1981, 1989, 1995-1996, 2000, 2002, 2006-2008
Lithuania	1980-2012
Luxembourg	1980-2011
Macao Special Administrative Region of China	1981-1982, 1984, 1986-2010
Macedonia, FYR	1982, 1989-2011
Madagascar	1984-1995
Malawi	2007
Malaysia	1980-1982, 1984-1986, 1990-2009
Maldives	1980-2011
Mali	1981, 1984-1994
Malta	1980-2011
Marshall Islands	1986-1997, 2005-2006
Mauritania	None Available
Mauritius	1980-2011
Mexico	1980-2012
Micronesia, Federated States of	2003
Moldova	1981-1982, 1984-2012
Mongolia	1980, 1984-1985, 1987-1990, 1994-2010
Montenegro	1992, 1995-2009, 2011
Morocco	1982, 1988-1991, 1993, 1995-1997, 1999, 2001, 2005, 2007-2008, 2011
Mozambique	2003
Myanmar	2005

Namibia	None Available
Nepal	None Available
Netherlands	1980-2011
New Zealand	1980-2011
Nicaragua	1984, 1987-1994, 1996-2011
Niger	None Available
Nigeria	2004-2007
North Korea	None Available
Norway	1980-2012
Occupied Palestinian Territory	1997-2009
Oman	2001, 2003-2007, 2009-2010
Pakistan	2009
Panama	1980-2010
Papua New Guinea	1980, 1985-1990
Paraguay	1980-1992, 1994-2010
Peru	1980-2010
Philippines	1980-2005, 2008-2009
Poland	1980-2011
Portugal	1980-2011
Puerto Rico	1980-2003, 2005-2010
Qatar	1981-1997, 1999-2011
Romania	1980-2011
Russia	1980-2012
Rwanda	None Available
Saint Lucia	1980-2006, 2008
Saint Vincent and the Grenadines	1980, 1982-1988, 1990, 1992, 1995-2010
Samoa	1980-1981
Sao Tome and Principe	1980, 1984-1987, 1991
Saudi Arabia	1998-2012
Senegal	None Available
Serbia	1995-2012
Seychelles	1980-1996, 1998-2012
Sierra Leone	None Available
Singapore	1980-2011
Slovakia	1980-2011
Slovenia	1982-2011
Solomon Islands	None Available
Somalia	None Available
South Africa	1980-1982, 1985, 1990, 1993-2012
South Korea	1980-2011
Spain	1980-2011
Sri Lanka	1980-1989, 1991-2007
Sudan	None Available
Suriname	1980-1982, 1984-2009
Swaziland	None Available
Sweden	1980-2011
Switzerland	1980-2011
Syria	1980, 1982-1985, 1987, 2005-2007
Taiwan	1980-2012
Tajikistan	1981-1982, 1984-2005, 2007-2008, 2011
Tanzania	2010
Thailand	1980-2000, 2002-2009
Timor-Leste	None Available
Togo	None Available
Tonga	1981-1983, 1990, 2001, 2003-2004
Trinidad and Tobago	1980-2008
Tunisia	1980, 1984, 1987-1989, 1993-1995, 1998-1999
Turkey	1980-1985, 1987-2012
Turkmenistan	1981-1982, 1984-2006
Uganda	None Available
Ukraine	1980-2012

United Arab Emirates	1985, 1990, 1995, 2000-2004
United Kingdom	1980-2012
United States	1980-2011
Uruguay	1980-2004, 2007-2009
Uzbekistan	1981-1982, 1984-2006
Vanuatu	None Available
Venezuela	1980-2011
Vietnam	None Available
Yemen	None Available
Zambia	None Available
Zimbabwe	1987, 1989-1990, 1995-2007

Appendix Table 2. Categorization of Garbage Codes.

	Type 1<br>No Inherent Information about the<br>Underlying Cause of Death	Type 2<br>Informative about the Underlying Cause of Death<br>but Sub-Optimal
**ICD10 Codes (3-digit)**	**A59, A71, A74*, B07, B08, B09, B30, B35, B36, B85, B87, B88, E50, E64*, F09, F17, F30, F31, F32, F33, F34, F35, F36, F37, F38, F39, F40, F41, F42, F43, F44, F45, F46, F47, F48, F49, F51, F52, F53, F54, F55, F56, F57, F58, F59, F60, F61, F62, F63, F64, F65, F66, F67, F68, F69, F70, F71, F72, F73, F74, F75, F76, F77, F78, F79, F80, F81, F82, F83, F84, F85, F86, F87, F88, F89, F90, F91, F92, F93, F94, F95, F96, F97, F98, F99, G15, G16, G17, G18, G19, G27, G28, G29, G32, G33, G34, G38, G39, G42, G43, G44, G47*, G48, G49, G50, G51, G52, G53, G54, G55, G56, G57, G58, G59, G60, G62, G63, G64, G65, G66, G67, G68, G69, G74, G75, G76, G77, G78, G79, G84, G85, G86, G87, G88, G89, H00, H01, H02, H03, H04, H05*, H06, H07, H08, H09, H10, H11, H12, H13, H14, H15, H16, H17, H18, H19, H20, H21, H22, H23, H24, H25, H27, H28, H29, H30, H31, H32, H33, H34, H35, H36, H37, H38, H39, H40, H41, H42, H43, H44, H45, H46, H47, H48, H49, H50, H51, H52, H53, H54, H55, H56, H57, H58, H59, H60, H61, H62, H65, H66, H67, H68, H69, H71, H72, H73, H74, H75, H76, H77, H78, H79, H80, H81, H82, H83, H84, H85, H86, H87, H88, H89, H90, H91, H92, H93, H94, H95, H96, H97, H98, H99, K00, K01, K02, K03, K04, K05, K06, K07, K08, K09, K10, K11, K12, K13, K14, K15, K16, K17, K18, K19, K30, K31*, L20, L21, L22, L23, L24, L25, L26, L27, L28, L29, L30, L40, L41, L42, L43, L44, L45, L49, L50, L52, L53, L54, L55, L56, L57, L58, L59, L60, L62, L63, L64, L65, L66, L67, L68, L70, L71, L72, L73, L74, L75, L76, L77, L78, L79, L80, L81, L82, L83, L84, L85, L86, L87, L90, L91, L92, L94, L95, L98*, L99, M04, M10, M11, M13, M14, M15, M16, M17, M18, M22, M23, M24, M25, M26, M27, M28, M29, M37, M38, M39, M43*, M44, M45, M46, M47, M48, M49*, M50, M51, M52, M53, M54, M55, M56, M57, M58, M59, M60, M61, M62, M63, M64, M65*, M66, M67, M68, M69, M70, M71*, M72, M73*, M74, M75, M76, M77, M78, M79, M80, M81, M82, M83, M84, M85, M89*, M90, M91, M92, M93, M94, M95, M96, M97, M98, M99, N09, N24, N32*, N33, N35, N37, N38, N40, N42, N43, N46, N47, N48, N52, N53, N54, N55, N56, N57, N58, N59, N60, N61, N62, N63, N64, N66, N67, N68, N69, N78, N79, N85, N86, N88, N89, N90, N91, N95, N97, Q08, Q09, Q10*, Q19, Q29, Q36, Q46, Q47, Q48, Q49, Q88, Q94, R07, R08, R09, R12, R14, R15, R19*, R20, R21, R22, R23, R24, R25, R26, R27, R28, R29, R30, R32, R33, R34, R35, R36, R37, R38, R39, R41, R42, R43, R44, R45, R46, R47, R48, R49, R51, R52, R53, R55, R57, R58, R59, R60, R61, R62, R63, R64, R65, R66, R67, R68, R69, R70, R71, R72, R74, R75, R76, R77, R78*, R79, R80, R81, R82, R83, R84, R85, R86, R87, R88, R89, R90, R91, R92, R93, R94, R95, R96, R97, R98, R99, U04, Z00, Z01, Z02, Z03, Z04, Z05, Z06, Z07, Z08, Z09, Z10, Z11, Z12, Z13, Z14, Z15, Z16, Z17, Z18, Z19, Z20, Z21, Z22, Z23, Z24, Z25, Z26, Z27, Z28, Z29, Z30, Z31, Z32, Z33, Z34, Z35, Z36, Z37, Z38, Z39, Z40, Z41, Z42, Z43, Z44, Z45, Z46, Z47, Z48, Z49, Z50, Z51, Z52, Z53, Z54, Z55, Z56, Z57, Z58, Z59, Z60, Z61, Z62, Z63, Z64, Z65, Z66, Z67, Z68, Z69, Z70, Z71, Z72, Z73, Z74, Z75, Z76, Z77, Z78, Z79, Z80, Z81, Z82, Z83, Z84, Z85, Z86, Z87, Z88, Z89, Z90, Z91, Z92, Z93, Z94, Z95, Z96, Z97, Z98, Z99**	**A14, A29, A40, A41, A45, A47, A48*, A49*, A61, A62, A64, A72, A73, A76, A97, A99, B11, B12, B13, B14, B17*, B19*, B28, B31, B32, B34, B55*, B61, B62, B64, B82, B83*, B84, B89, B93, B94*, B95*, B96, B97, B98, B99, C14, C26, C27, C28, C29, C35, C36, C39, C42, C46, C55, C57*, C59, C63*, C68*, C75*, C76, C77, C78, C79, C80, C87, C98, C99, D00*, D01*, D02*, D07*, D08, D09*, D10*, D13*, D14*, D17, D18, D19, D20, D21, D26*, D28*, D29*, D30*, D36*, D37*, D38*, D39*, D40*, D41*, D44*, D48*, D49*, D54, D59*, D75*, D79, D84*, D85, D87, D88, D89*, D90, D91, D92, D93, D94, D95, D96, D97, D98, D99, E07*, E08, E17, E18, E19, E34*, E35, E37, E38, E39, E47, E48, E49, E62, E69, E90, E91, E92, E93, E94, E95, E96, E97, E98, E99, F06*, F07*, F08, F50*, G00*, G01, G02, G03*, G09, G91, G93*, G94, G96, G98, G99, H26, I00, I03, I04, I14, I16, I17, I18, I19, I29, I32, I43, I50, I51*, I52, I53, I54, I55, I56, I57, I58, I59, I62*, I64, I67*, I68, I69*, I79, I90, I92, I93, I94, I98*, I99, ID5, J07, J08, J15*, J17, J18, J19, J22, J23, J24, J25, J26, J27, J28, J29, J48, J49, J50, J51, J52, J53, J54, J55, J56, J57, J58, J59, J64, J71, J72, J73, J74, J75, J76, J77, J78, J79, J81, J83, J85, J87, J88, J89, J90, J93, J97, J98, J99, K23, K24, K32, K33, K34, K39, K47, K48, K49, K53, K54, K63*, K69, K75*, K78, K79, K84, K87, K88, K89, K92*, K93*, K96, K97, K98, K99, L06, L07, L09, L15, L16, L17, L18, L19, L31, L32, L33, L34, L35, L36, L37, L38, L39, L46, L47, L48, L61, L69, L96, M12*, M19, M20, M21, M87*, N39*, N84*, O08, O17, O18, O19, O27, O37, O38, O39, O49, O50, O51, O52, O53, O54, O55, O56, O57, O58, O59, O78, O79, O93, O94, O95, P06, P16, P17, P18, P30, P31, P32, P33, P34, P40, P41, P42, P43, P44, P45, P46, P47, P48, P49, P62, P63, P64, P65, P66, P67, P68, P69, P73, P79, P82, P85, P86, P87, P88, P89, P96*, P97, P98, P99, Q89*, Q99*, R54, S00, S01, S02, S03, S04, S05, S06, S07, S08, S09, S10, S11, S12, S13, S14, S15, S16, S17, S18, S19, S20, S21, S22, S23, S24, S25, S26, S27, S28, S29, S30, S31, S32, S33, S34, S35, S36, S37, S38, S39, S40, S41, S42, S43, S44, S45, S46, S47, S48, S49, S50, S51, S52, S53, S54, S55, S56, S57, S58, S59, S60, S61, S62, S63, S64, S65, S66, S67, S68, S69, S70, S71, S72, S73, S74, S75, S76, S77, S78, S79, S80, S81, S82, S83, S84, S85, S86, S87, S88, S89, S90, S91, S92, S93, S94, S95, S96, S97, S98, S99, T00, T01, T02, T03, T04, T05, T06, T07, T08, T09, T10, T11, T12, T13, T14, T15, T16, T17, T18, T19, T20, T21, T22, T23, T24, T25, T26, T27, T28, T29, T30, T31, T32, T33, T34, T35, T36, T37, T38, T39, T40, T41, T42, T43, T44, T45, T46, T47, T48, T49, T50, T51, T52, T53, T54, T55, T56, T57, T58, T59, T60, T61, T62, T63, T64, T65, T66, T67, T68, T69, T70, T71, T73, T74, T75, T76, T78, T79, T80, T81, T82, T83, T84, T85, T86, T87, T88, T90, T91, T92, T93, T94, T95, T96, T97, T98, V87*, V88*, V89, V90*, V99, W47, W48, W63, W71, W72, W82, W95, W96, W98, X07, X41, X42, X44, X55, X56, X59, Y09, Y10, Y11, Y12, Y13, Y14, Y15, Y16, Y17, Y18, Y19, Y20, Y21, Y22, Y23, Y24, Y25, Y26, Y27, Y28, Y29, Y30, Y31, Y32, Y33, Y34, Y85, Y86, Y87*, Y89*, Y90, Y91, Y92, Y93, Y94, Y95, Y96, Y97, Y98, Y99**

*** At least one corresponding 4-digit code not classified garbage**
Causes classified as garbage codes at the 4-digit level, but not at the corresponding 3-digit level not displayed

Appendix Table 3. Combinations of Age or Sex with Cause of Death Deemed Impossible.

GBD Code	Cause	Impossible Before Age	Impossible After Age	Impossible Amongst
A.1.2.1	HIV-TB	Post Neonatal	74	None
A.1.2.2	HIV (other)	Post Neonatal	74	None
A.2.1.7	Amoebiasis	Post Neonatal	None	None
A.2.11	Measles	Post Neonatal	59	None
A.2.2	Typhoid fevers	Post Neonatal	None	None
A.2.8	Diptheria	1	59	None
A.2.9	Whooping cough	Post Neonatal	59	None
A.3.11	Dengue	Post Neonatal	None	None
A.3.13	Rabies	Post Neonatal	None	None
A.3.14	Intestinal nematode	Post Neonatal	None	None
A.3.14.1	Ascariasis	Post Neonatal	None	None
A.3.2	Chagas	Post Neonatal	None	None
A.3.3	Leishmaniasis	Post Neonatal	None	None
A.3.4	Trypanosomiasis	1	None	None
A.3.5	Schistosomiasis	Post Neonatal	None	None
A.3.6	Cysticercosis	5	None	None
A.3.7	Echinococcosis	1	None	None
A.4.1	Maternal hemorrhage	15	49	Males
A.4.2	Maternal sepsis	15	49	Males
A.4.3	Maternal hypertension	15	64	Males
A.4.4	Obstructed labor	15	None	Males
A.4.5	Abortion	15	49	Males
A.4.6	Other maternal disorders	15	49	Males
A.5.3	Neonatal sepsis	None	Late Neonatal	None
A.6.1	Protein-energy malnutrition	Late Neonatal	None	None
A.6.2	Iodine deficiency	1	None	None
A.6.5	Other nutritional deficiencies	Post Neonatal	None	None
A.7.1.1	Syphilis	10	None	None
A.7.1.2	Chlamydia	10	None	None
A.7.1.3	Gonnorhea	10	None	None
A.7.1.5	Other STDs	10	None	None
A.7.2.3	Acute hepatitis C	Post Neonatal	None	None
A.7.2.4	Acute hepatitis E	Post Neonatal	None	None
B.1.1	Esophageal cancer	15	None	None
B.1.10	Colorectal cancer	15	None	None
B.1.11	Mouth cancer	15	None	None
B.1.12	Nasopharynx cancer	5	None	None
B.1.13	Other pharynx cancer	15	None	None
B.1.14	Gallbladder cancer	15	None	None
B.1.15	Pancreatic cancer	15	None	None
B.1.16	Melanoma	15	None	None
B.1.17	Non-melanoma skin cancer	15	None	None
B.1.18	Ovarian cancer	15	None	Males
B.1.19	Testicular cancer	15	None	Females
B.1.2	Stomach cancer	15	None	None
B.1.20	Kidney cancers	1	None	None
B.1.21	Bladder cancer	15	None	None
B.1.22	Brain cancer	1	None	None
B.1.23	Thyroid cancer	10	None	None
B.1.25	Non-Hodgkin lymphoma	1	None	None
B.1.26	Myeloma	15	None	None
B.1.27	Leukemia	1	None	None
B.1.3	Liver cancer	5	None	None
B.1.3.1	Liver cancer hepatitis B	5	None	None
B.1.3.2	Liver cancer hepatitis C	5	None	None
B.1.3.3	Liver cancer alcohol	15	None	None
B.1.3.4	Liver cancer other	5	None	None
B.1.4	Larynx cancer	15	None	None
B.1.5	Lung cancer	15	None	None
B.1.6	Breast cancer	15	None	Males
B.1.7	Cervical cancer	15	None	Males
B.1.8	Uterine cancer	15	None	Males
B.1.9	Prostate cancer	15	None	Females
B.10.2.12	Decubitus ulcer	1	None	None
B.2.1	Rheumatic heart disease	1	None	None
B.2.10	Other cardio & circulatory	1	None	None
B.2.2	Ischemic heart disease	1	None	None
B.2.3.1	Ischemic stroke	1	None	None
B.2.3.2	Hemorrhagic stroke	1	None	None
B.2.4	Hypertensive heart disease	1	None	None
B.2.5	Cardiomyopathy	1	None	None
B.2.6	Atrial fibrillation	1	None	None
B.2.8	Peripheral vascular disease	1	None	None
B.3.1	COPD	1	None	None
B.3.2	Pneumoconiosis	1	None	None
B.3.3	Asthma	1	None	None
B.3.4	Interstitial lung diseases	1	None	None
B.3.5	Other respiratory diseases	1	None	None
B.4.3	Cirrhosis alcohol	15	None	None
B.5.1	Peptic ulcer	1	None	None
B.5.10	Other digestive diseases	1	None	None
B.5.2	Gastrititis & duodenitis	1	None	None
B.5.3	Appendicitis	1	None	None
B.5.5	Inguinal & femoral hernia	1	None	None
B.5.6	Inflammatory bowel disease	1	None	None
B.5.7	Vascular intestinal disorders	1	None	None
B.5.8	Gall bladder diseases	1	None	None
B.5.9	Pancreatitis	1	None	None

B.6.1	Alzheimer's disease	40	None	None
B.6.2	Parkinson's disease	30	None	None
B.6.4	Multiple sclerosis	5	None	None
B.6.7	Other neurological disorders	1	None	None
B.7.1	Schizophrenia	10	None	None
B.7.11	Other mental & behavioral	1	None	None
B.7.3.1	Opioid use	15	None	None
B.7.3.2	Cocaine use	15	None	None
B.7.3.3	Amphetamine use	10	None	None
B.7.3.5	Other drug use	10	None	None
B.7.7	Eating disorders	5	None	None
B.8.4.2	Urolithiasis	1	None	None
B.8.5.1	Fibroids	15	None	Males
B.8.5.4	Endometriosis	15	54	Males
B.8.5.5	Genital prolapse	15	None	Males
B.8.5.7	Other gynecological disorders	15	59	Males
B.9.1	Rheumatoid arthritis	5	None	None
B.9.5	Other musculoskeletal	10	None	None
C.1.1.2	Bicycle road injury	1	None	None
C.3.1	Self-harm	10	None	None

Appendix Table 4. CSMF Accuracy Transformations from Simulation.

	CSMF Accuracy (%)				
**Value of Indicator (%)**	**Garbage Coding***	**Age/Sex Unspecified***	**Medically Impossible Diagnoses***	**Completeness**	**Length of Cause List**
100	100	100	100	100	100
99	99.3	99.3	99.1	99.2	99.8
98	98.7	98.5	98.3	98.4	99.6
97	98	97.8	97.5	97.6	99.3
96	97.3	97.1	96.8	97.2	99.1
95	96.7	96.4	96.2	96.8	98.9
94	96	95.7	95.6	96	98.7
93	95.4	95	95.1	94.8	98.4
92	94.7	94.3	94.6	93.6	98.2
91	94	93.6	94.2	92.8	97.9
90	93.4	92.9	93.8	92.5	97.7
89	92.7	92.2	93.4	92.1	97.5
88	92	91.5	93.2	91.3	97.2
87	91.3	90.8	93	90.1	97
86	90.7	90.2	92.8	88.9	96.7
85	90	89.5	92.7	88.1	96.5
84	89.3	88.8	92.5	87.7	96.2
83	88.6	88.1	92.4	87.3	95.9
82	87.9	87.4	92.3	86.5	95.7
81	87.2	86.7	92.3	85.3	95.4
80	86.6	86	92.3	84.1	95.1
79	85.9	85.4	92.2	83.3	94.9
78	85.2	84.7	92.3	82.8	94.6
77	84.5	84	92.2	82.4	94.3
76	83.8	83.3	92.2	81.6	94
75	83.1	82.6	92.1	80.4	93.7
74	82.4	81.9	92.1	79.1	93.4
73	81.7	81.2	92.1	78.3	93.1
72	81	80.5	92.1	77.4	92.8
71	80.3	79.8	92.1	77	92.5
70	79.6	79.1	92	76.6	92.2
69	78.9	78.4	92	75.7	91.9
68	78.2	77.7	92	74.4	91.6
67	77.4	77	92	73.1	91.3
66	76.7	76.3	92	72.2	90.9
65	76	75.6	92	71.8	90.6
64	75.3	74.9	92	71.3	90.3
63	74.6	74.2	91.9	70.4	89.9
62	73.9	73.4	91.9	69	89.6
61	73.2	72.7	91.9	67.7	89.2
60	72.5	72	91.9	66.7	88.9
59	71.8	71.2	91.9	66.3	88.5
58	71.1	70.5	91.9	65.8	88.1
57	70.4	69.8	91.9	64.8	87.8
56	69.7	69	91.9	63.4	87.4
55	69	68.2	91.9	61.9	87
54	68.3	67.5	91.9	60.9	86.6
53	67.6	66.7	91.9	60.4	86.2
52	66.9	65.9	91.9	59.9	85.8
51	66.2	65.2	91.9	59	85.3
50	65.5	64.4	91.9	57.4	84.9
49	64.8	63.6	91.9	55.9	84.5
48	64	62.8	91.9	55.4	84
47	63.3	62	91.9	54.8	83.6
46	62.6	61.2	91.9	53.2	83.1
45	61.9	60.4	91.9	52.4	82.6
44	61.2	59.6	91.9	51.6	82.1
43	60.5	58.8	91.9	50	81.6
42	59.8	57.9	91.9	49.1	81.1
41	59.1	57.1	91.9	48.3	80.6
40	58.4	56.3	91.9	46.6	80
39	57.7	55.4	91.9	45.8	79.5
38	57	54.6	91.9	45	78.9
37	56.3	53.7	91.9	43.3	78.3
36	55.6	52.8	91.9	41.7	77.7
35	54.9	52	91.9	40.9	77.1
34	54.2	51.1	91.9	40	76.5
33	53.5	50.2	91.9	38.4	75.8
32	52.8	49.4	91.9	37.5	75.2
31	52.1	48.5	91.9	36.7	74.5
30	51.3	47.7	91.9	35	73.8
29	50.6	47	91.9	34.1	73
28	49.9	46.2	91.9	33.2	72.3
27	49.1	45.4	91.9	31.5	71.5
26	48.4	44.8	91.9	30.6	70.7

25	47.6	44	91.9	29.8	69.8
24	46.9	43.3	91.9	28.6	68.9
23	46.1	42.7	91.9	27.5	68
22	45.3	42.1	91.9	26.7	67
21	44.5	41.5	91.9	25.8	66
20	43.7	41	91.9	24.1	64.9
19	42.9	40.5	91.9	22.9	63.8
18	42.1	40.1	91.9	21.7	62.6
17	41.3	39.7	91.9	19.8	61.4
16	40.4	39.3	91.9	18.9	60.1
15	39.5	39.2	91.9	17.9	58.7
14	38.6	39	91.9	16	57.2
13	37.6	38.6	91.9	15.4	55.6
12	36.6	38.5	91.9	14.8	53.8
11	35.6	38.5	91.9	13.7	51.9
10	34.6	38.5	91.9	11.9	49.8
9	33.5	38.5	91.9	10	47.5
8	32.4	38.5	91.9	9.1	45
7	31.2	38.5	91.9	8.3	42.1
6	30	38.5	91.9	6.9	38.7
5	28.9	38.5	91.9	5.5	34.7
4	27.7	38.5	91.9	4.4	29.9
3	26.3	38.5	91.9	3.3	23.6
2	24.9	38.5	91.9	1.7	14.8
1	23.5	38.5	91.9	0.8	14.8
0	23.5	38.5	91.9	0	0

* Subtracted from one so that higher values are preferable to lower, as with other indicators

Accuracy values are global average. Region-specific values were used in final computation.

Appendix Table 5. Example Simulation Procedure.

Description: This table depicts a simplified demonstration of the simulation procedure for six hypothetical causes of death and for indicator representing unspecified age or sex.

Column A:	This column displays the names of the six hypothetical causes of death in this demonstration. There are two infections diseases (A1 and A2) two non-communicable diseases (B1 and B2) and two injuries (C1 and C2)
Column B:	This columns displays the proportion (in this hypothetical scenario) of each cause which was reported with either an unknown age or unknown sex.<br>In the actual simulation, these proportions were computed by dividing the number of unspecified deaths by the total number of deaths, one cause at a time, amongst all ICD10 data.
Column C:	This column is simply a normalized, or rescaled, transformation of column B. each proportion in column B was divided by the total of column B to normalize the proportions such that they sum to 1.<br>This is done for operational purposes which will become clear at later stages. (See formulas below)
Column D:	This column displays the (hypothetical) death counts from the simulation dataset, which was described in the methods section of the text. Briefly, the simulation dataset is the region-level GBD 2010 estimates with added noise.<br>These death counts have been arbitrarily generated to sum to 50 total deaths and do not reflect anything used in the actual simulation.
Column E:	This column is simply the cause-specific mortality fractions (CSMFs) associated with the death counts from column D. Like column C, these were computed by dividing each row in column D by the total of column D. (See formulas below)
Column F:	This column displays the resulting death counts from the simulation at a level of 5% simulated loss (described in the methods section of the text).<br>In other words, in this iteration of the simulation, 5% (2.5 deaths) of the total number of deaths are lost due to unspecified ages or sexes.<br>The 2.5 lost deaths were not evenly distributed amongst the six causes. Instead, column C was multiplied by 2.5 to distribute the lost deaths according to the proportions in column B, and these products were subtracted from column D. (See formulas below)
Column G:	This column is simply the cause-specific mortality fractions (CSMFs) associated with the death counts from column F. Like column E, these were computed by dividing each row in column F by the total of column F. (See formulas below)
Column H:	This column is identical to column F, with the exception that it represents another iteration of the simulation at a level of 15% simulated loss.
Column I:	This column is identical to column G, with the exception that it represents another iteration of the simulation at a level of 15% simulated loss.
Column J:	This column is identical to column F, with the exception that it represents another iteration of the simulation at a level of 25% simulated loss.
Column K:	This column is identical to column G, with the exception that it represents another iteration of the simulation at a level of 25% simulated loss.
Column L:	This column displays the CSMF accuracy associated with the iteration of the simulation depicted by column G. It is the CSMF accuracy, or degree of concordance between the CSMFs in column G and the CSMFs in column E.<br>CSMF accuracy is described in the methods section of the text, and the exact formula is listed below. Note that although %5 of all deaths are lost due to unspecified ages or sexes, only 3.3% accuracy is lost.
Column M:	This column displays the CSMF accuracy associated with the iteration of the simulation depicted by column I. It is the CSMF accuracy, or degree of concordance between the CSMFs in column I and the CSMFs in column E.<br>CSMF accuracy is described in the methods section of the text, and the exact formula is listed below. Note that although %15 of all deaths are lost due to unspecified ages or sexes, only 11.1% accuracy is lost.
Column N:	This column displays the CSMF accuracy associated with the iteration of the simulation depicted by column K. It is the CSMF accuracy, or degree of concordance between the CSMFs in column K and the CSMFs in column E.<br>CSMF accuracy is described in the methods section of the text, and the exact formula is listed below. Note that although %25 of all deaths are lost due to unspecified ages or sexes, only 21% accuracy is lost.<br>Also note that as the simulated loss increases (over columns L, M and N), the hypothetical CSMFs (columns G, I and K) become increasingly more distorted, resulting in more CSMF accuracy loss as a fraction of simulated loss.

A	B	C	D	E	F	G	H	I	J	K
**Cause**	**Average Age/Sex Unknown Proportion (Computed from data)**	**Average Age/Sex Unknown Proportion Rescaled**	**Simulation Deaths**	**Simulation CSMF**	**Hypothetical Deaths (Simulated Loss = 5%)**	**Hypothetical CSMF (Simulated Loss = 5%)**	**Hypothetical Deaths (Simulated Loss = 15%)**	**Hypothetical CSMF (Simulated Loss = 15%)**	**Hypothetical Deaths (Simulated Loss = 25%)**	**Hypothetical CSMF (Simulated Loss = 25%)**
A1	0.005	0.040	5	0.100	4.901	0.103	4.702	0.111	4.504	0.120
A2	0.05	0.397	8	0.160	7.008	0.148	5.024	0.118	3.040	0.081
B1	0.002	0.016	12	0.240	11.960	0.252	11.881	0.280	11.802	0.315
B2	0.004	0.032	17	0.340	16.921	0.356	16.762	0.394	16.603	0.443
C1	0.015	0.119	3	0.060	2.702	0.057	2.107	0.050	1.512	0.040
C2	0.05	0.397	5	0.100	4.008	0.084	2.024	0.048	0.040	0.001

Column	Formula
A	N/A
B	deaths unknown / total deaths (from all ICD10 data aggregated)
C	B/SUM(B:B)
D	N/A
E	D/SUM(D:D)
F	D-(C*(0.05*SUM(D:D)))
G	F/SUM(F:F)
H	D-(C*(0.15*SUM(D:D)))
I	H/SUM(H:H)
J	D-(C*(0.25*SUM(D:D)))
K	J/SUM(J:J)
L	1-((SUM(ABS(E3-G3),ABS(E4-G4),ABS(E5-G5),ABS(E6-G6),ABS(E7-G7),ABS(E8-G8)))/(2*(1-MIN(E3:E8))))
M	1-((SUM(ABS(E3-I3),ABS(E4-I4),ABS(E5-I5),ABS(E6-I6),ABS(E7-I7),ABS(E8-I8)))/(2*(1-MIN(E3:E8))))
N	1-((SUM(ABS(E3-K3),ABS(E4-K4),ABS(E5-K5),ABS(E6-K6),ABS(E7-K7),ABS(E8-K8)))/(2*(1-MIN(E3:E8))))

L	M	N
**CSMF Accuracy (Simulated Loss = 5%)**	**CSMF Accuracy (Simulated Loss = 15%)**	**CSMF Accuracy (Simulated Loss = 25%)**
96.7%	88.9%	79.0%

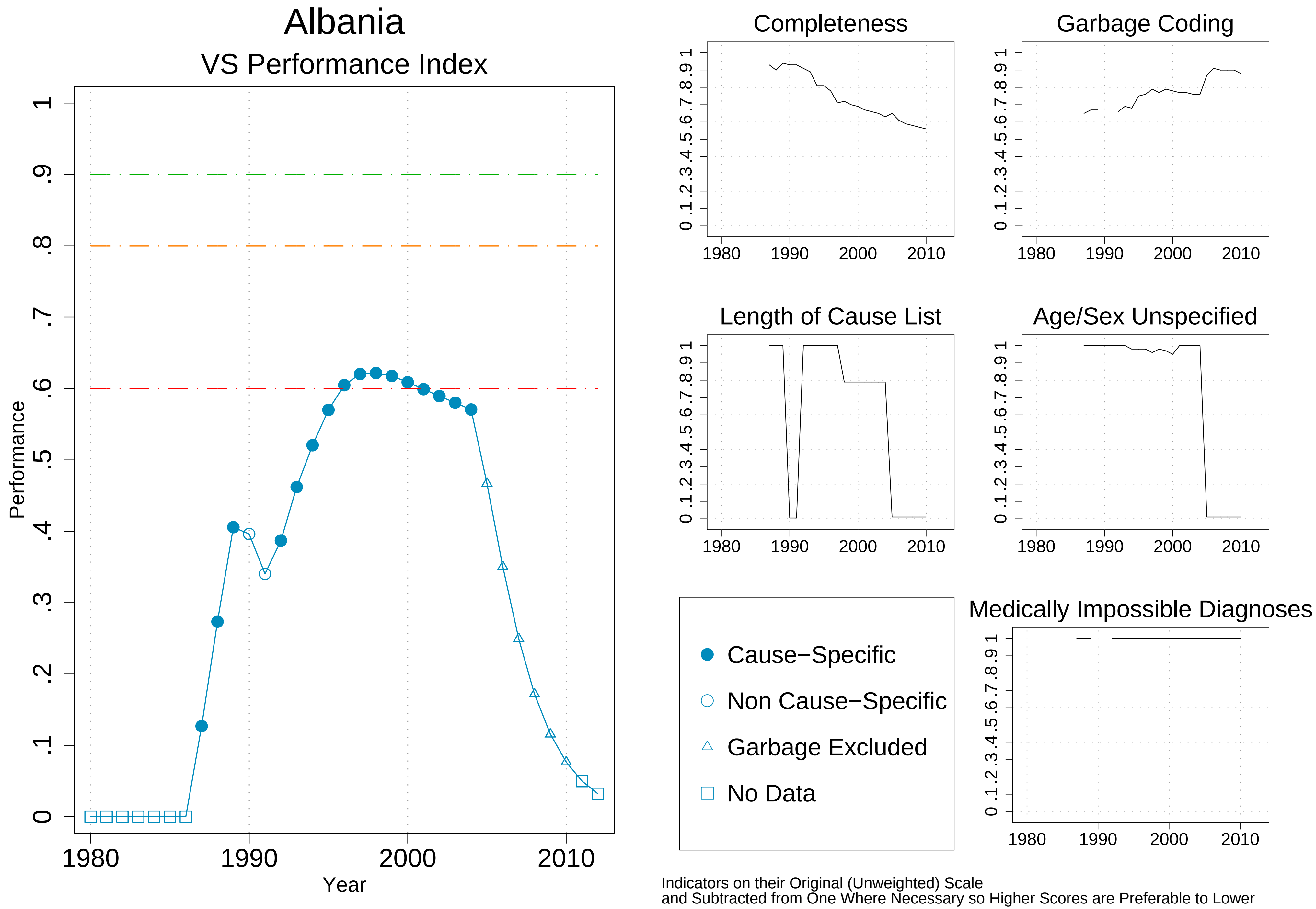

Indicators on their Original (Unweighted) Scale
and Subtracted from One Where Necessary so Higher Scores are Preferable to Lower

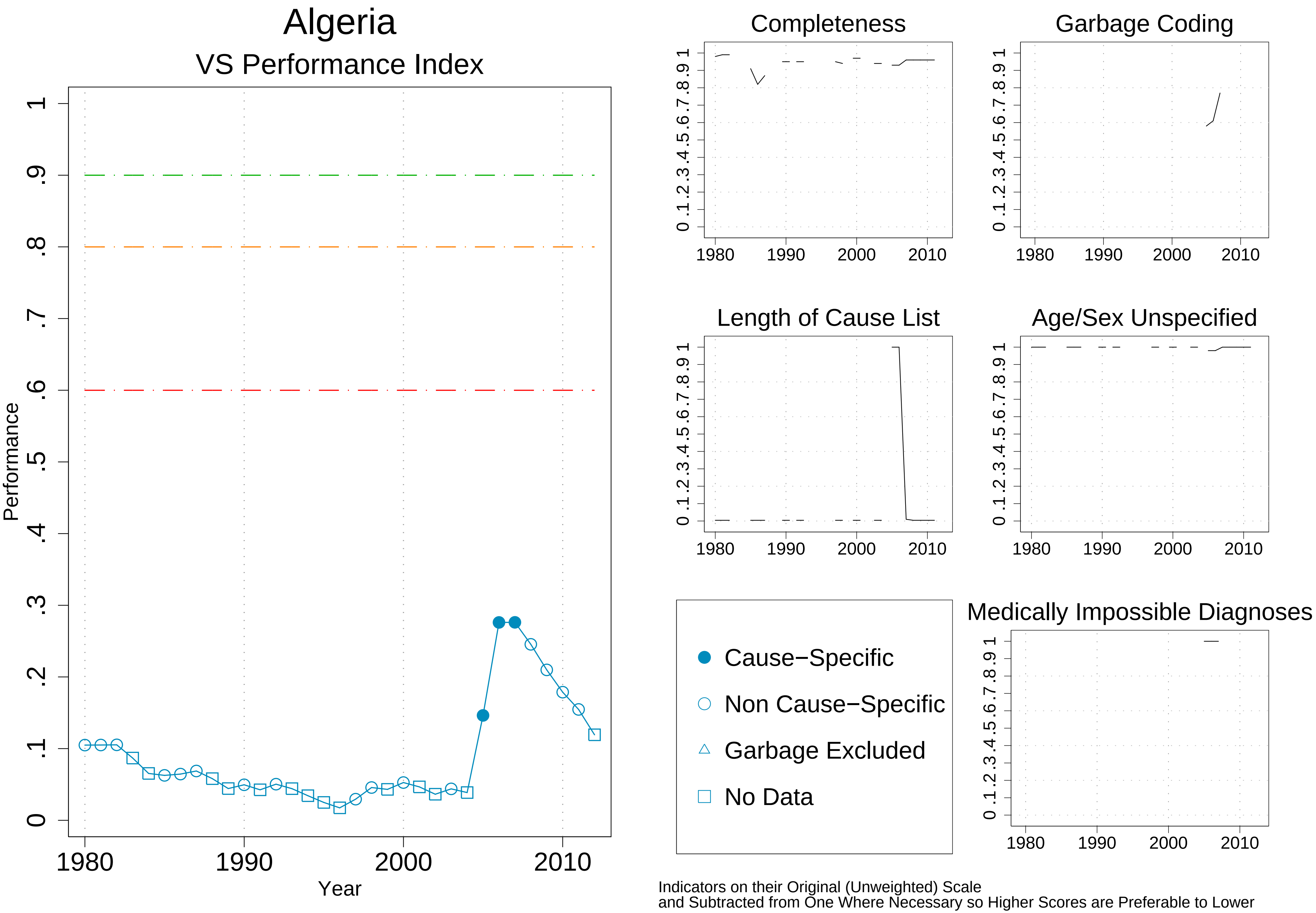

Indicators on their Original (Unweighted) Scale
and Subtracted from One Where Necessary so Higher Scores are Preferable to Lower

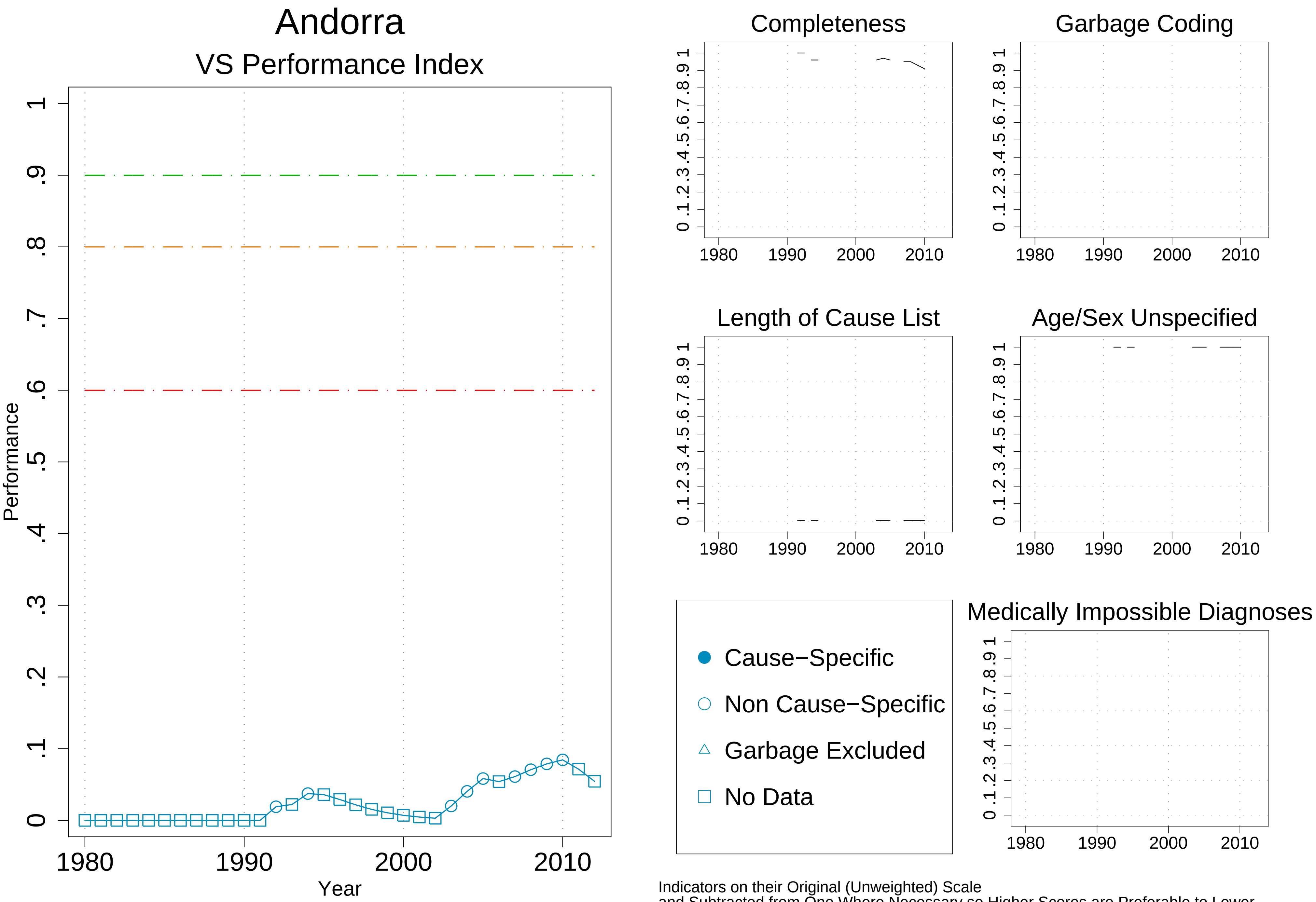

Indicators on their Original (Unweighted) Scale
and Subtracted from One Where Necessary so Higher Scores are Preferable to Lower

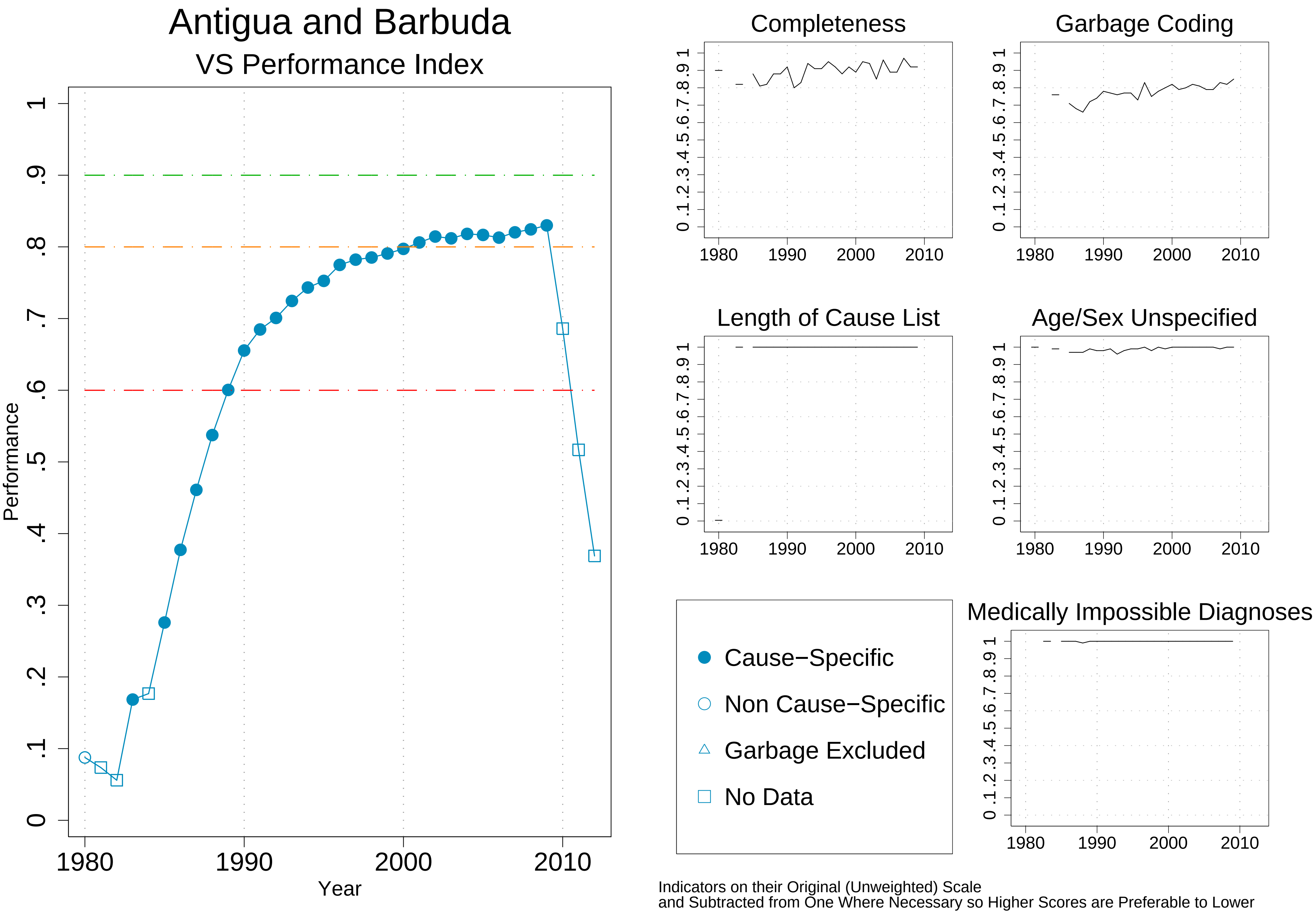

Indicators on their Original (Unweighted) Scale
and Subtracted from One Where Necessary so Higher Scores are Preferable to Lower

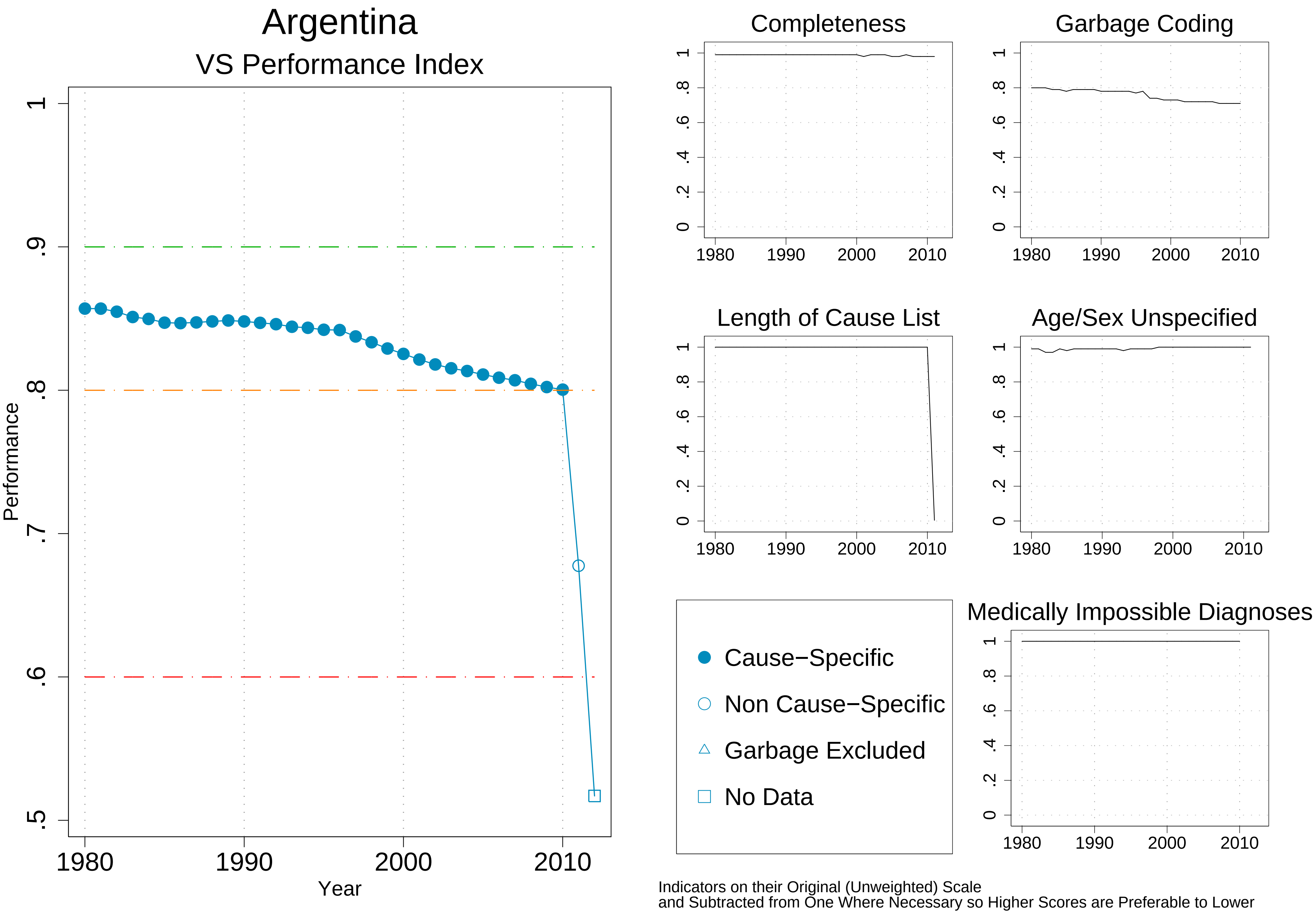

Indicators on their Original (Unweighted) Scale
and Subtracted from One Where Necessary so Higher Scores are Preferable to Lower

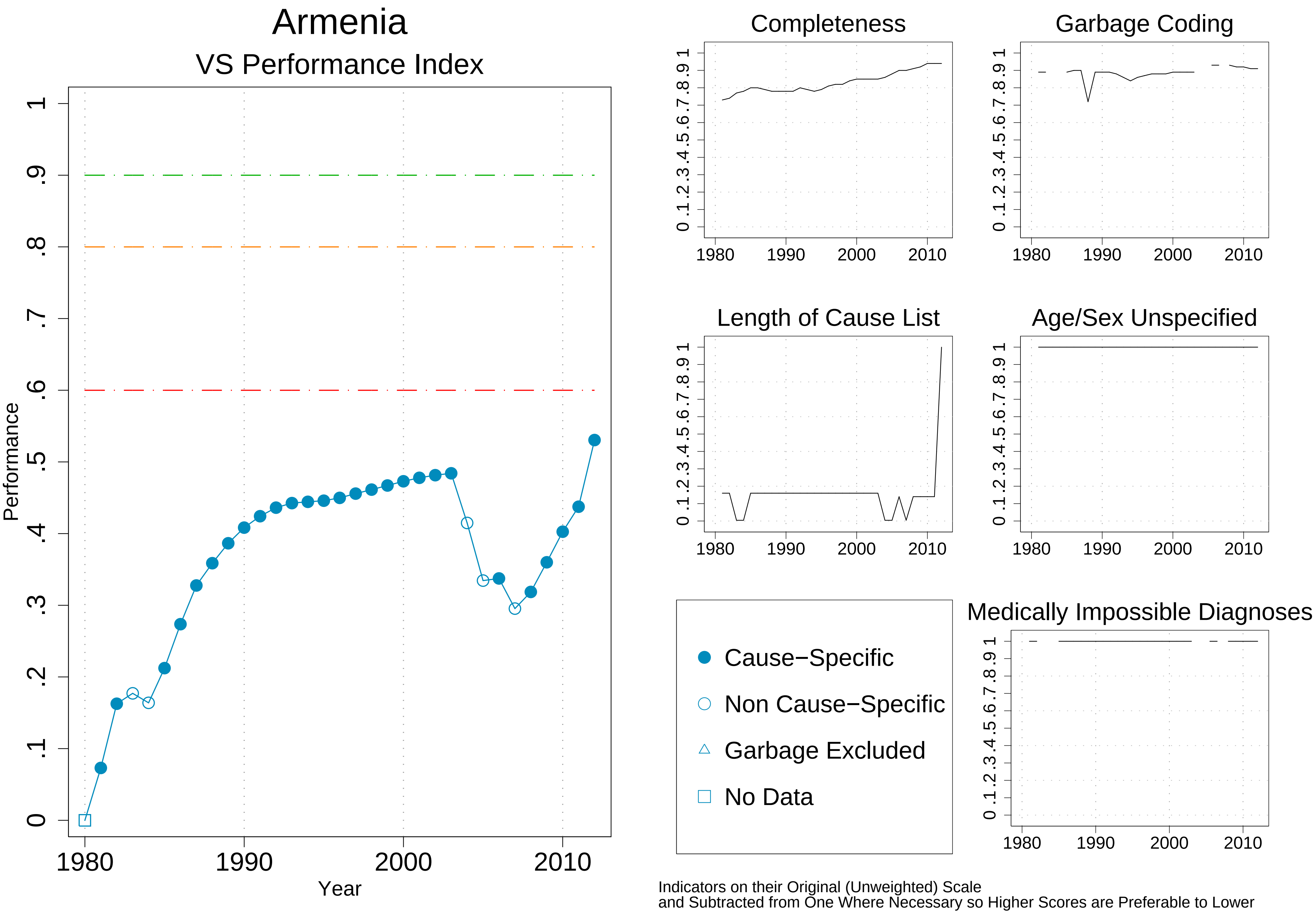

Indicators on their Original (Unweighted) Scale
and Subtracted from One Where Necessary so Higher Scores are Preferable to Lower

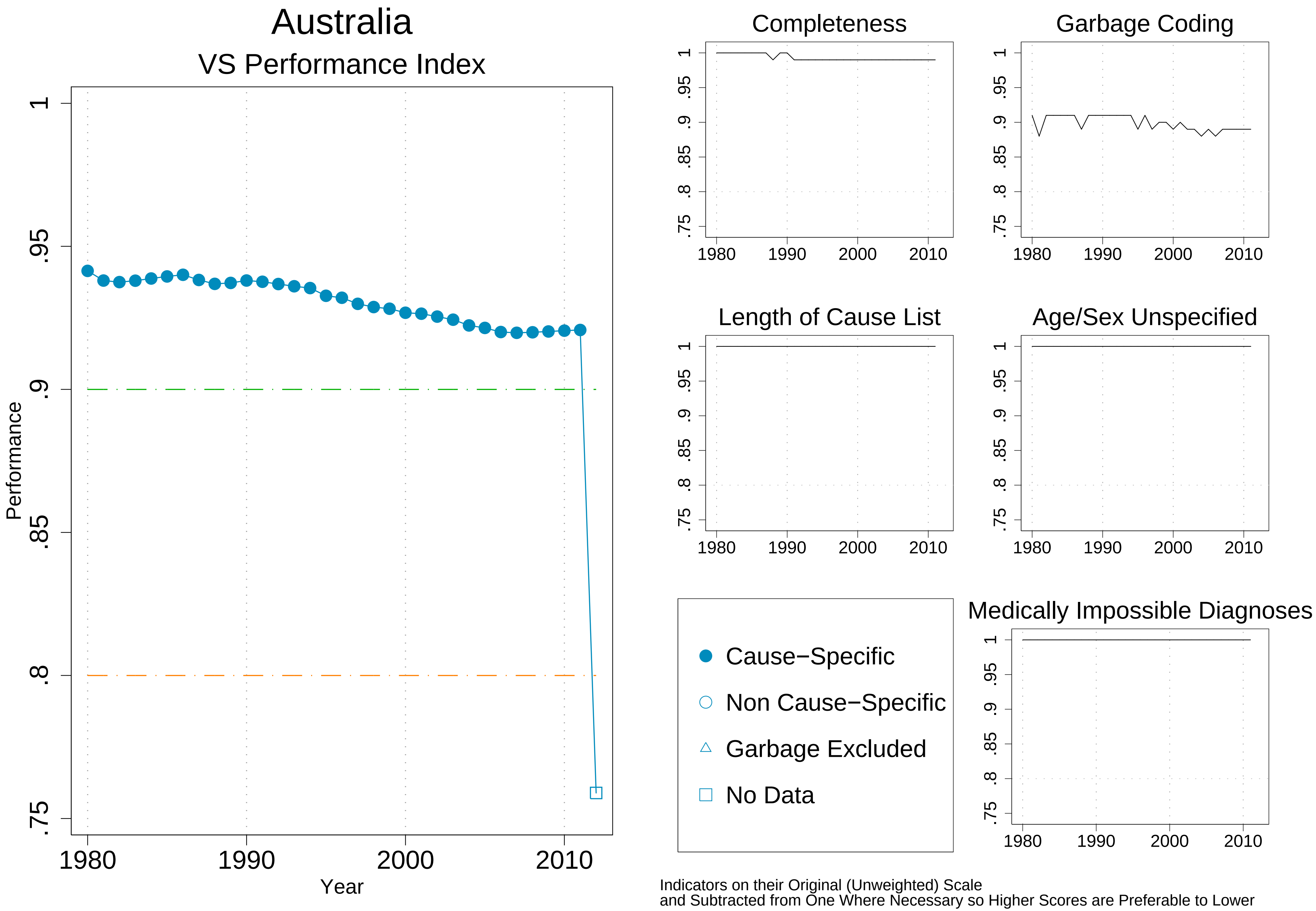
Australia
VS Performance Index
Performance
Year
1980
1990
2000
2010
.75
.8
.85
.9
.95
1
Completeness
Garbage Coding
Length of Cause List
Age/Sex Unspecified
Medically Impossible Diagnoses
Cause−Specific
Non Cause−Specific
Garbage Excluded
No Data
Indicators on their Original (Unweighted) Scale
and Subtracted from One Where Necessary so Higher Scores are Preferable to Lower

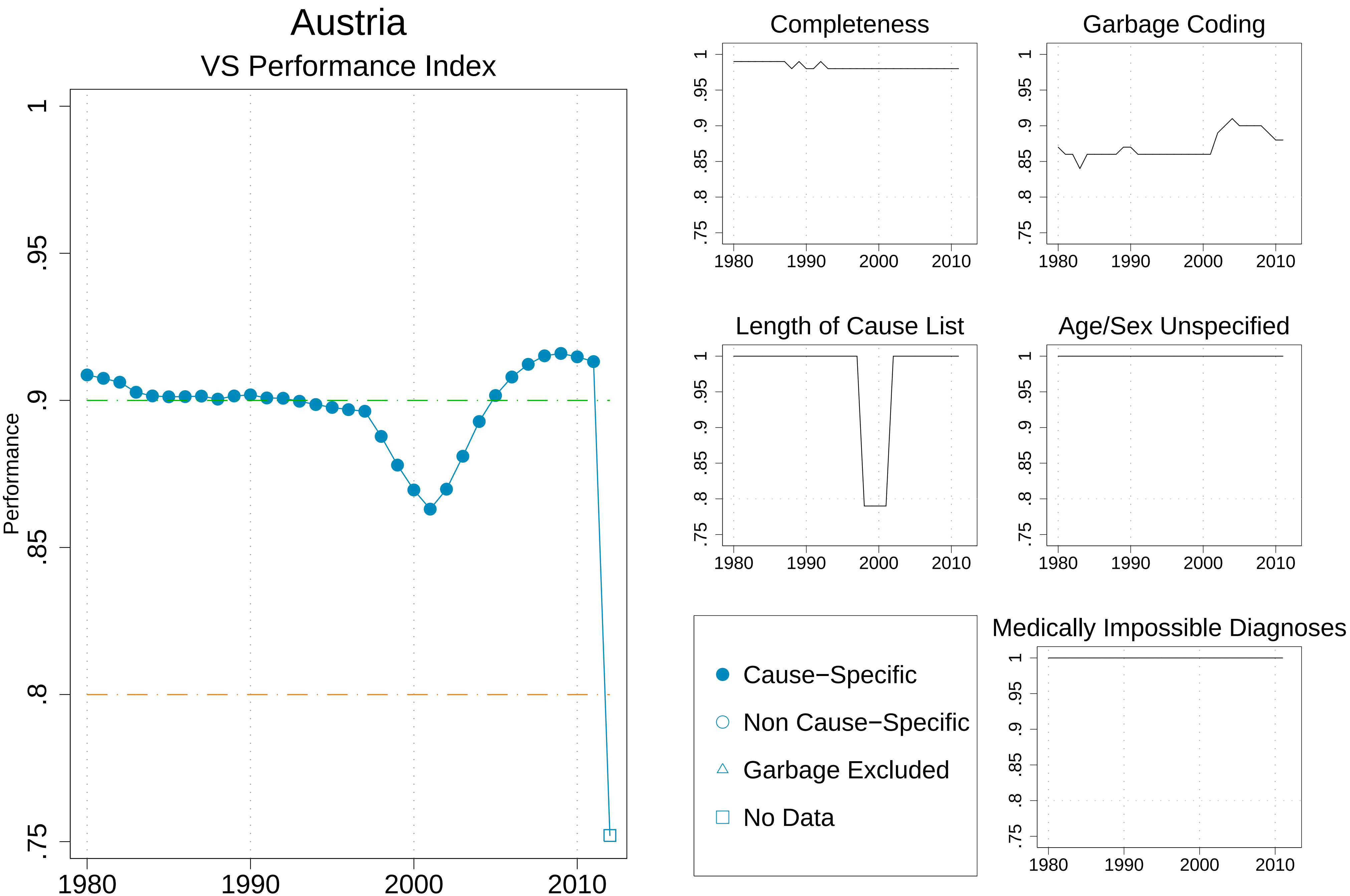

Indicators on their Original (Unweighted) Scale
and Subtracted from One Where Necessary so Higher Scores are Preferable to Lower

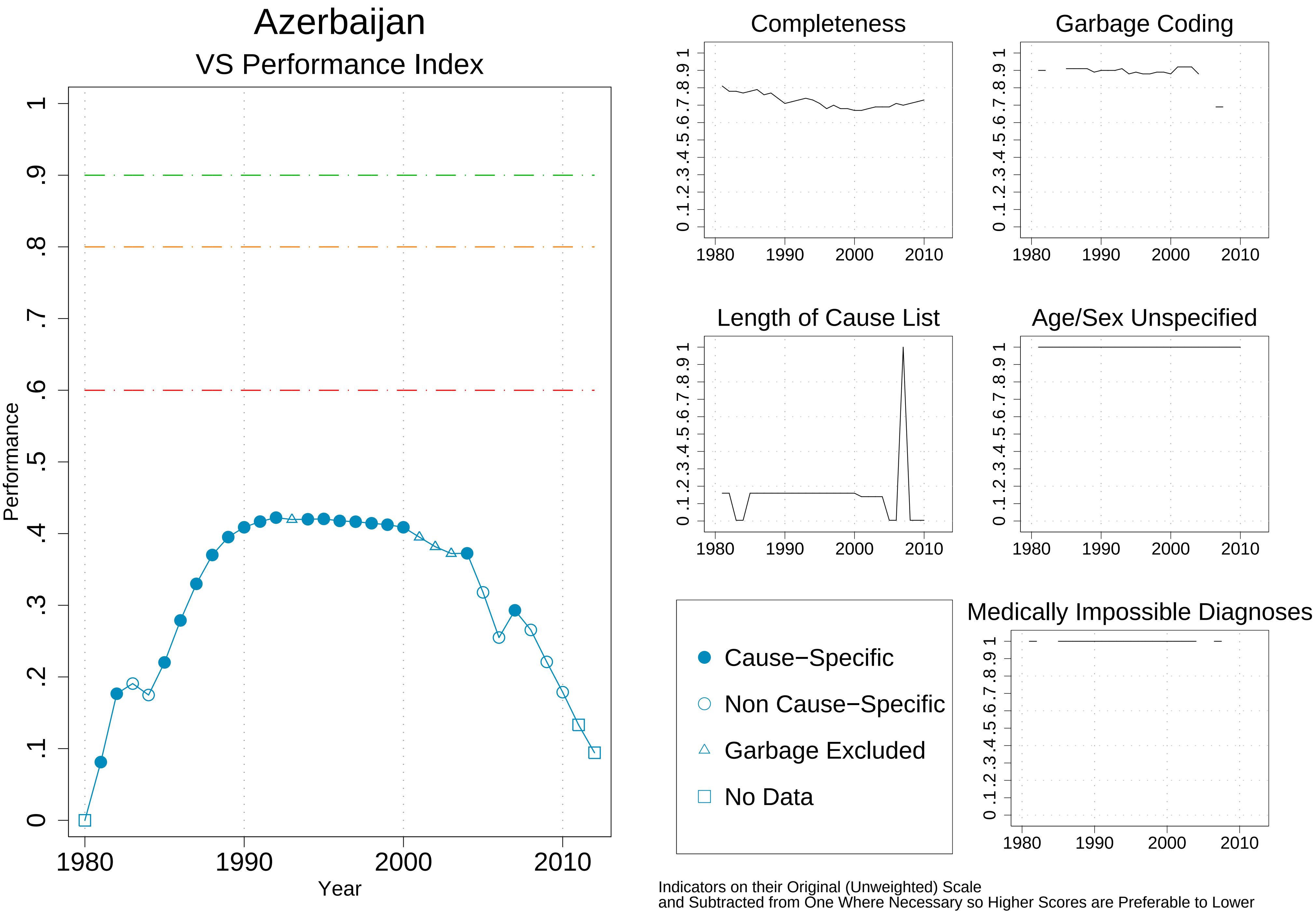

Azerbaijan
VS Performance Index
Performance
Year
Completeness
Garbage Coding
Length of Cause List
Age/Sex Unspecified
Medically Impossible Diagnoses
Cause−Specific
Non Cause−Specific
Garbage Excluded
No Data
Indicators on their Original (Unweighted) Scale
and Subtracted from One Where Necessary so Higher Scores are Preferable to Lower

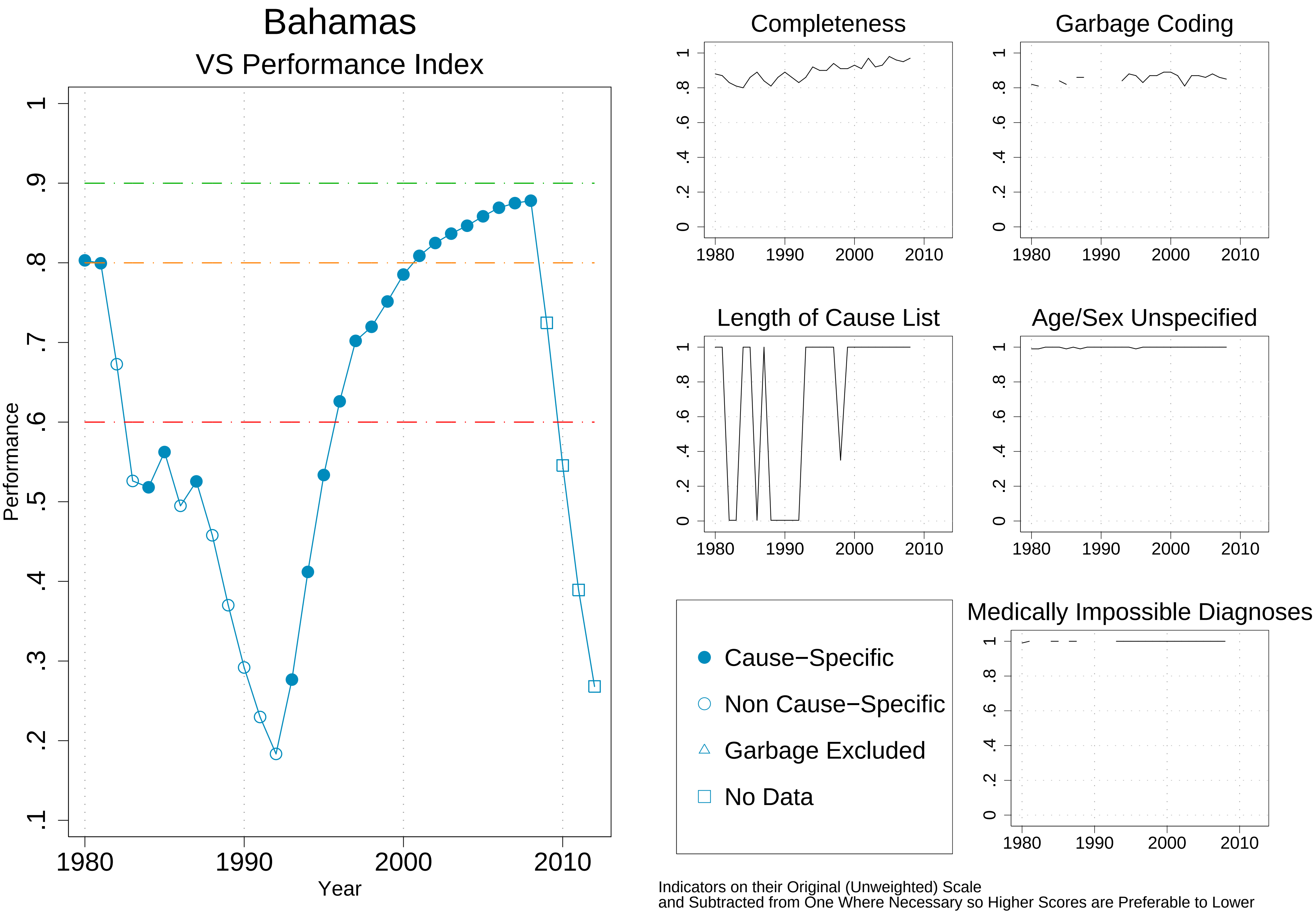

Indicators on their Original (Unweighted) Scale
and Subtracted from One Where Necessary so Higher Scores are Preferable to Lower

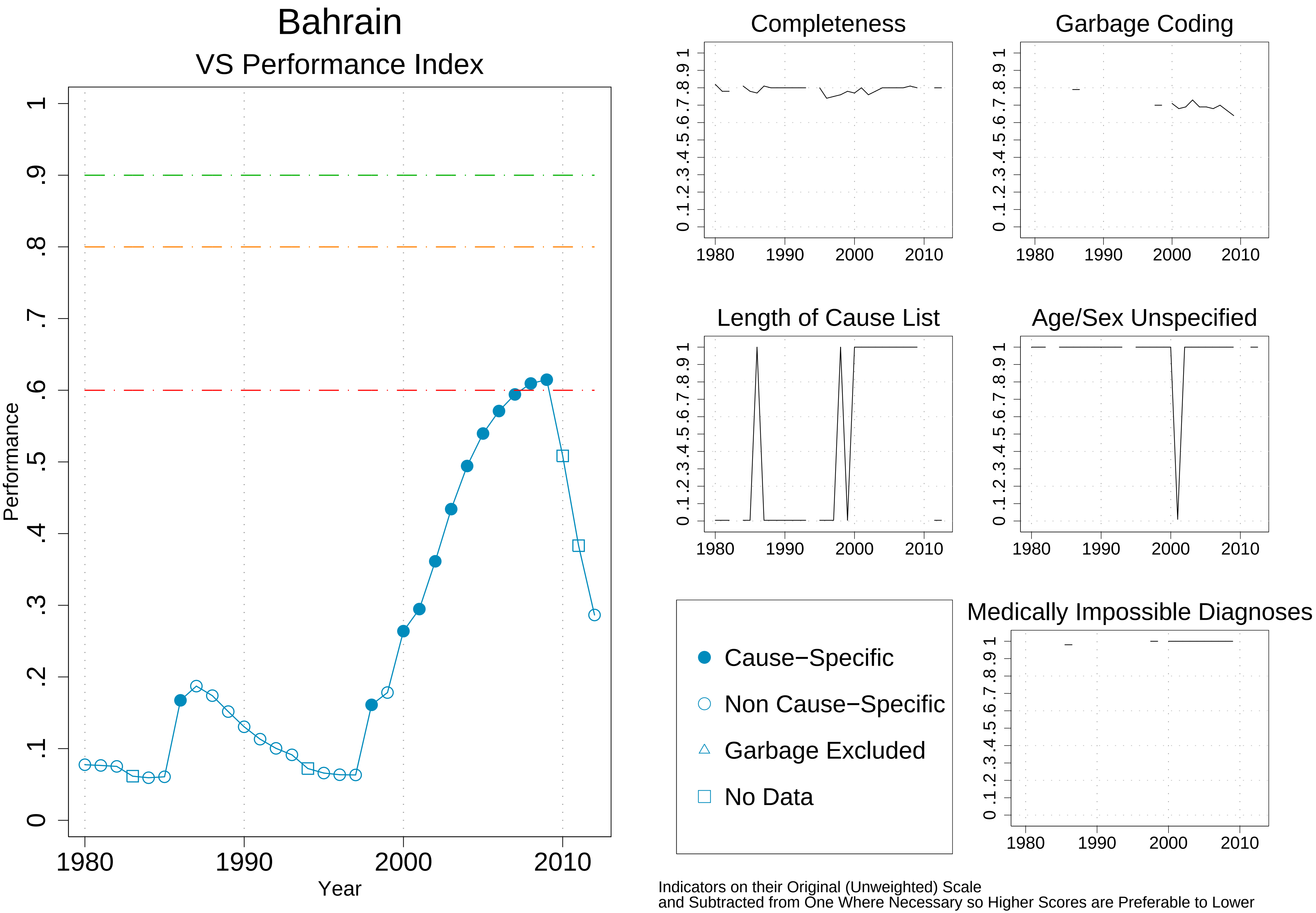

Bahrain
VS Performance Index
Performance
Year
Completeness
Garbage Coding
Length of Cause List
Age/Sex Unspecified
Medically Impossible Diagnoses
Cause−Specific
Non Cause−Specific
Garbage Excluded
No Data
Indicators on their Original (Unweighted) Scale
and Subtracted from One Where Necessary so Higher Scores are Preferable to Lower

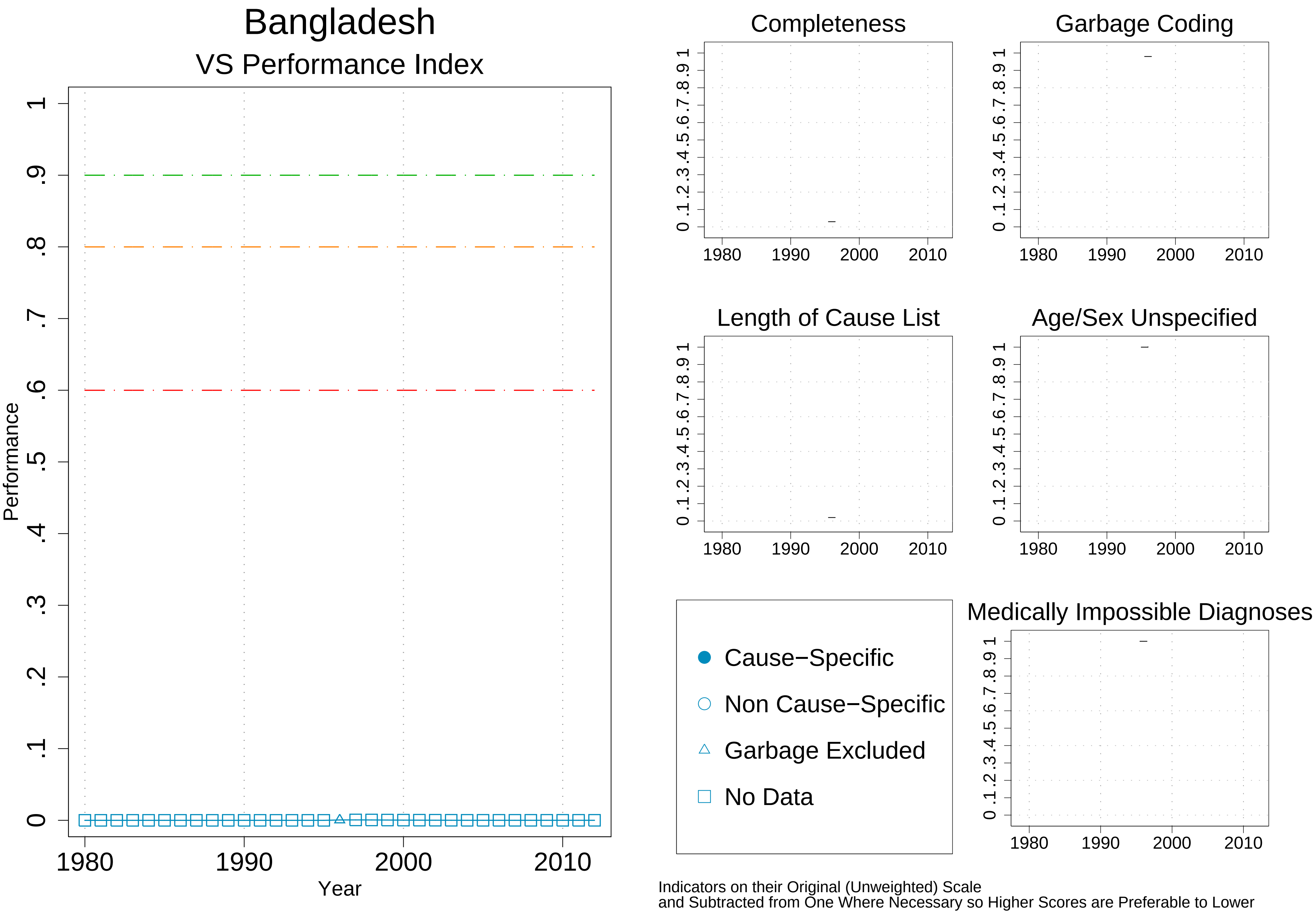

Indicators on their Original (Unweighted) Scale
and Subtracted from One Where Necessary so Higher Scores are Preferable to Lower

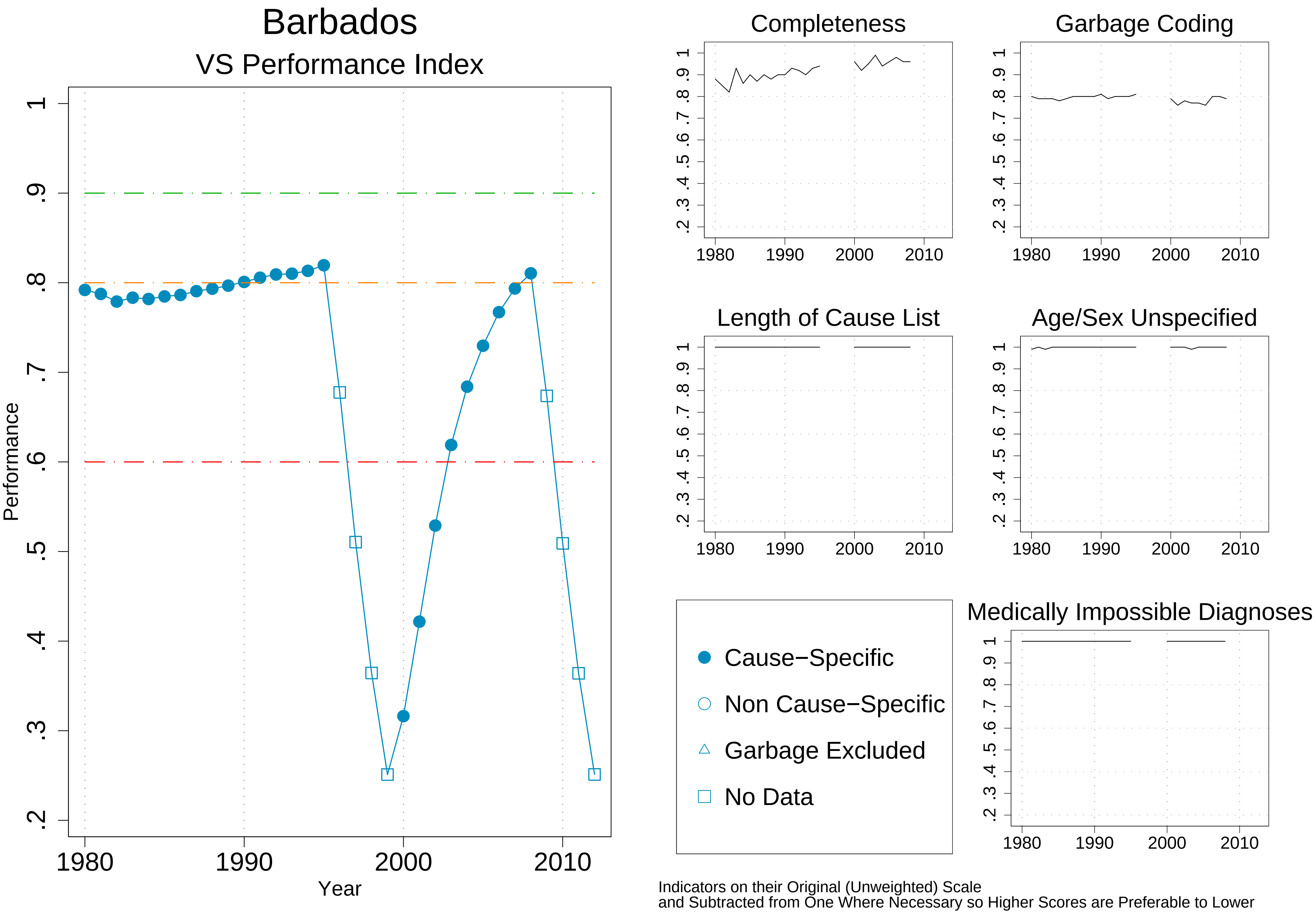

Indicators on their Original (Unweighted) Scale
and Subtracted from One Where Necessary so Higher Scores are Preferable to Lower

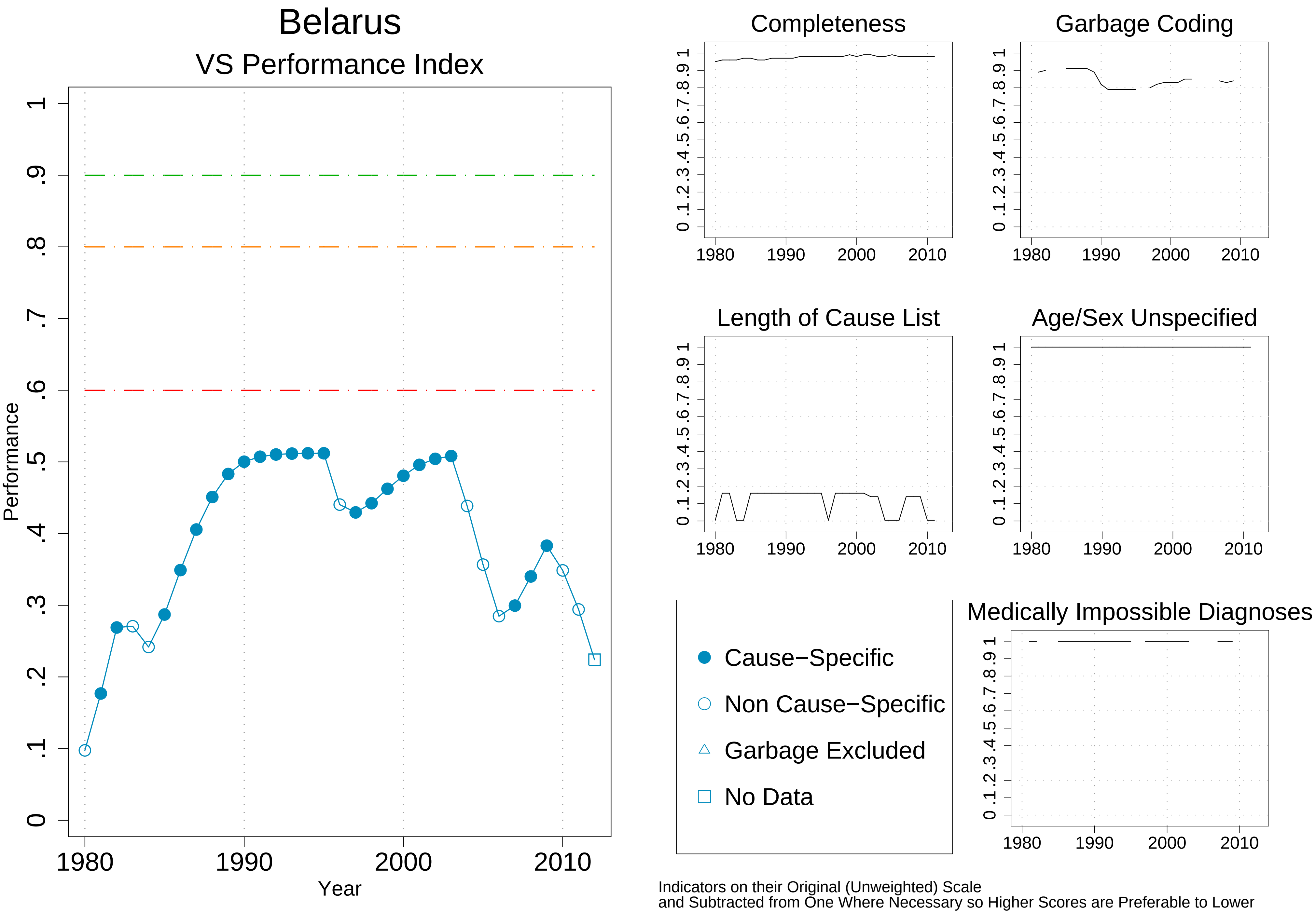

Indicators on their Original (Unweighted) Scale
and Subtracted from One Where Necessary so Higher Scores are Preferable to Lower

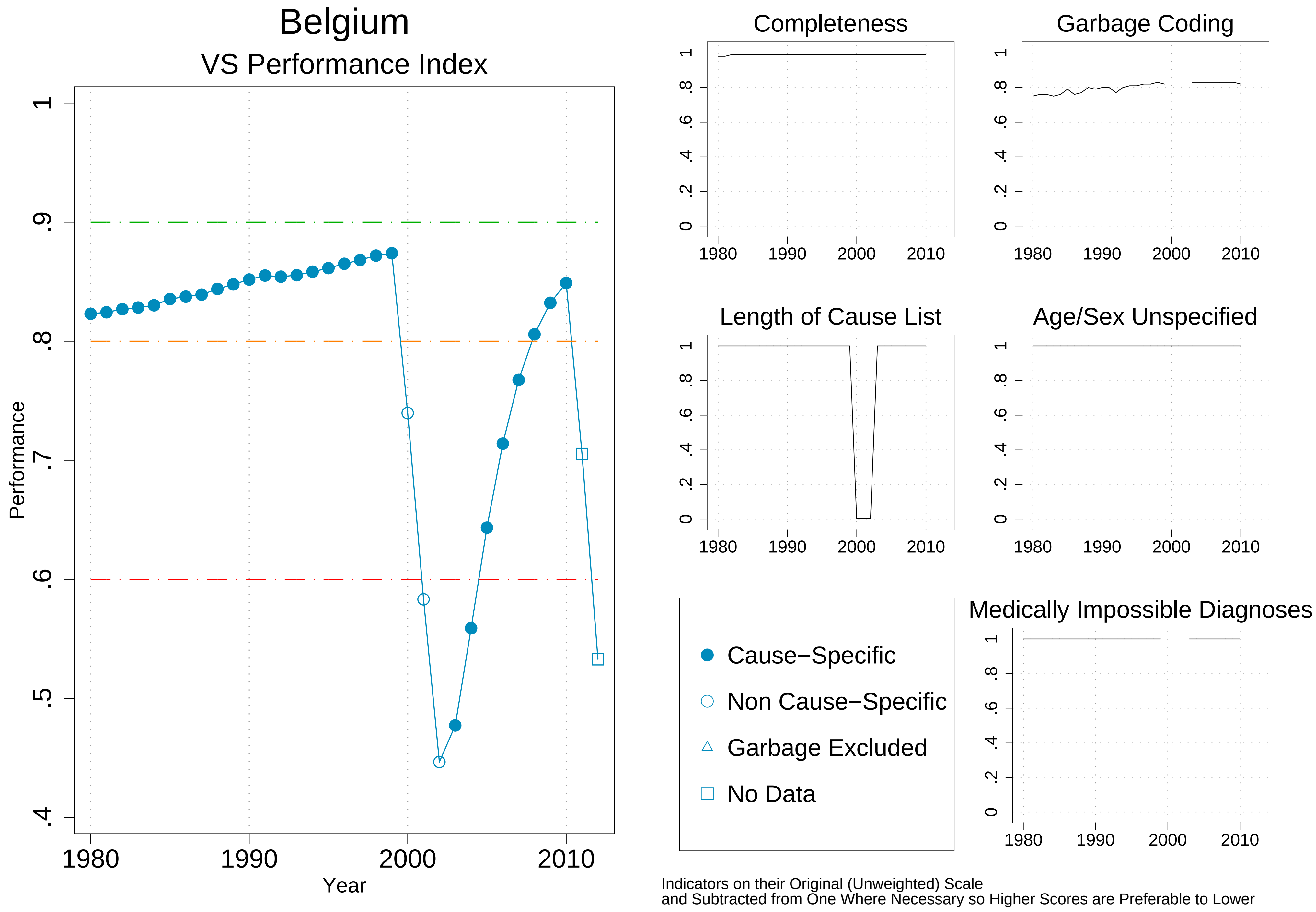
Belgium
VS Performance Index
Performance
Year
Completeness
Garbage Coding
Length of Cause List
Age/Sex Unspecified
Medically Impossible Diagnoses
Cause−Specific
Non Cause−Specific
Garbage Excluded
No Data
Indicators on their Original (Unweighted) Scale
and Subtracted from One Where Necessary so Higher Scores are Preferable to Lower

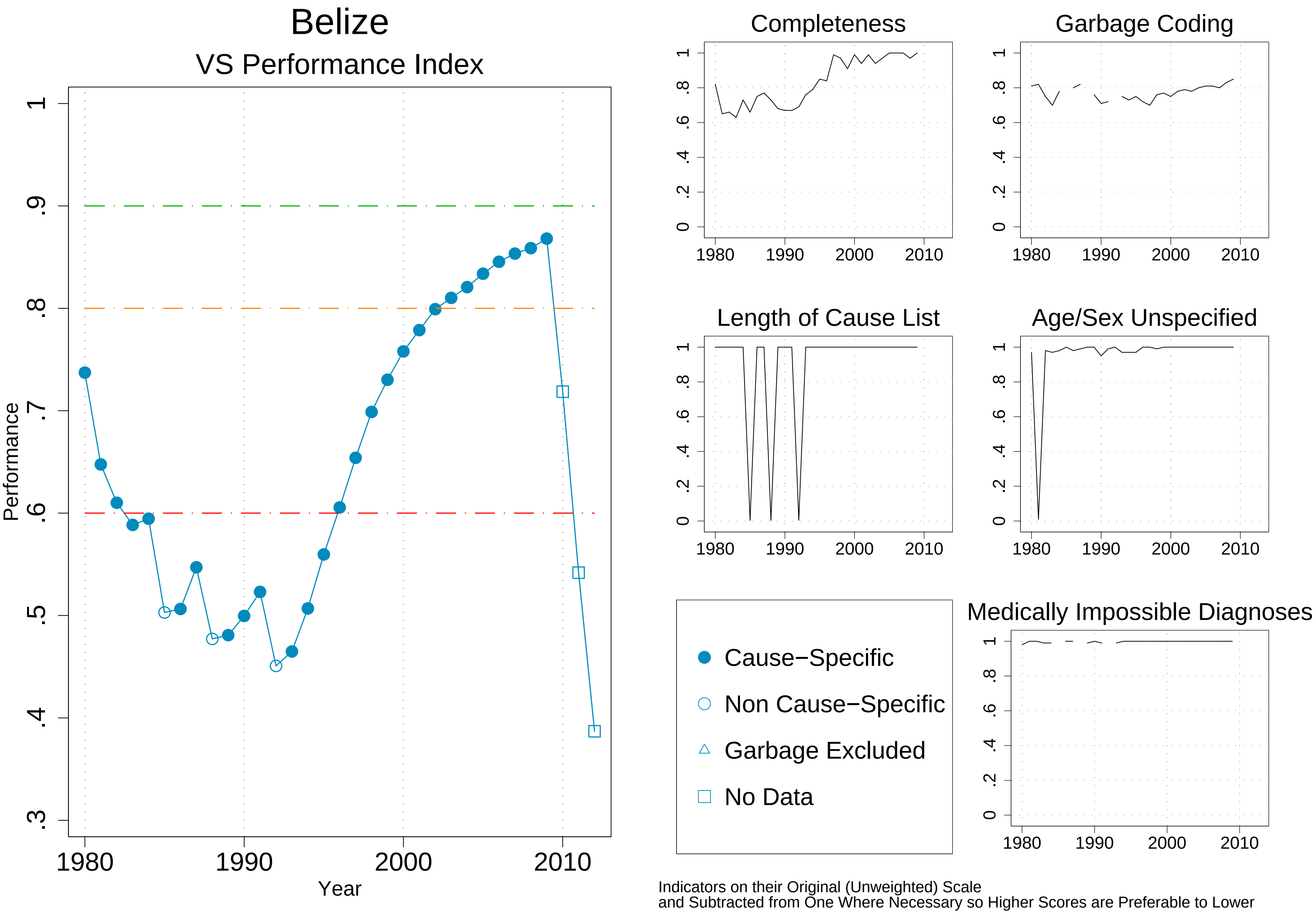

Belize
VS Performance Index
Performance
Year
Completeness
Garbage Coding
Length of Cause List
Age/Sex Unspecified
Medically Impossible Diagnoses
Cause−Specific
Non Cause−Specific
Garbage Excluded
No Data
Indicators on their Original (Unweighted) Scale
and Subtracted from One Where Necessary so Higher Scores are Preferable to Lower

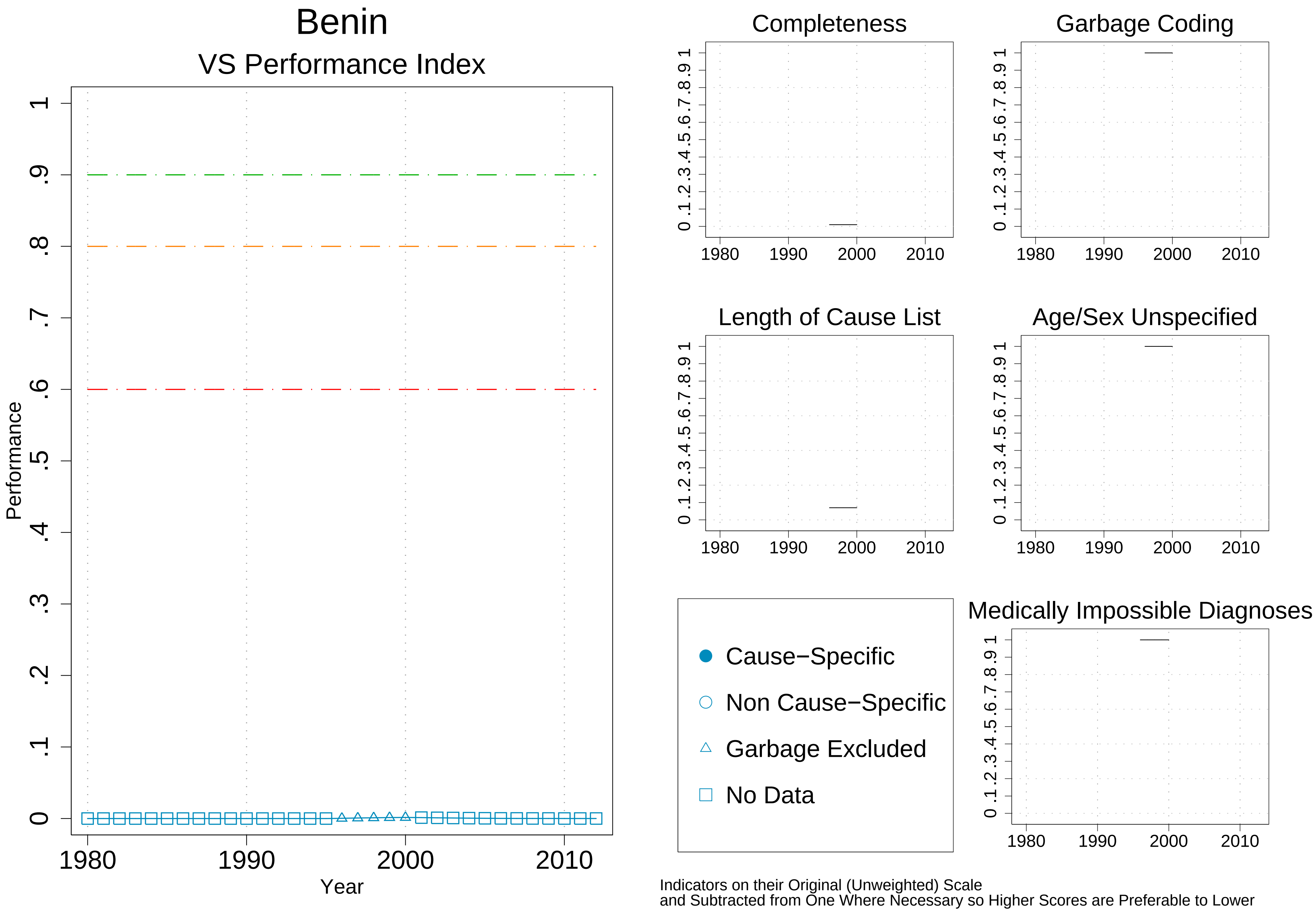

Indicators on their Original (Unweighted) Scale
and Subtracted from One Where Necessary so Higher Scores are Preferable to Lower

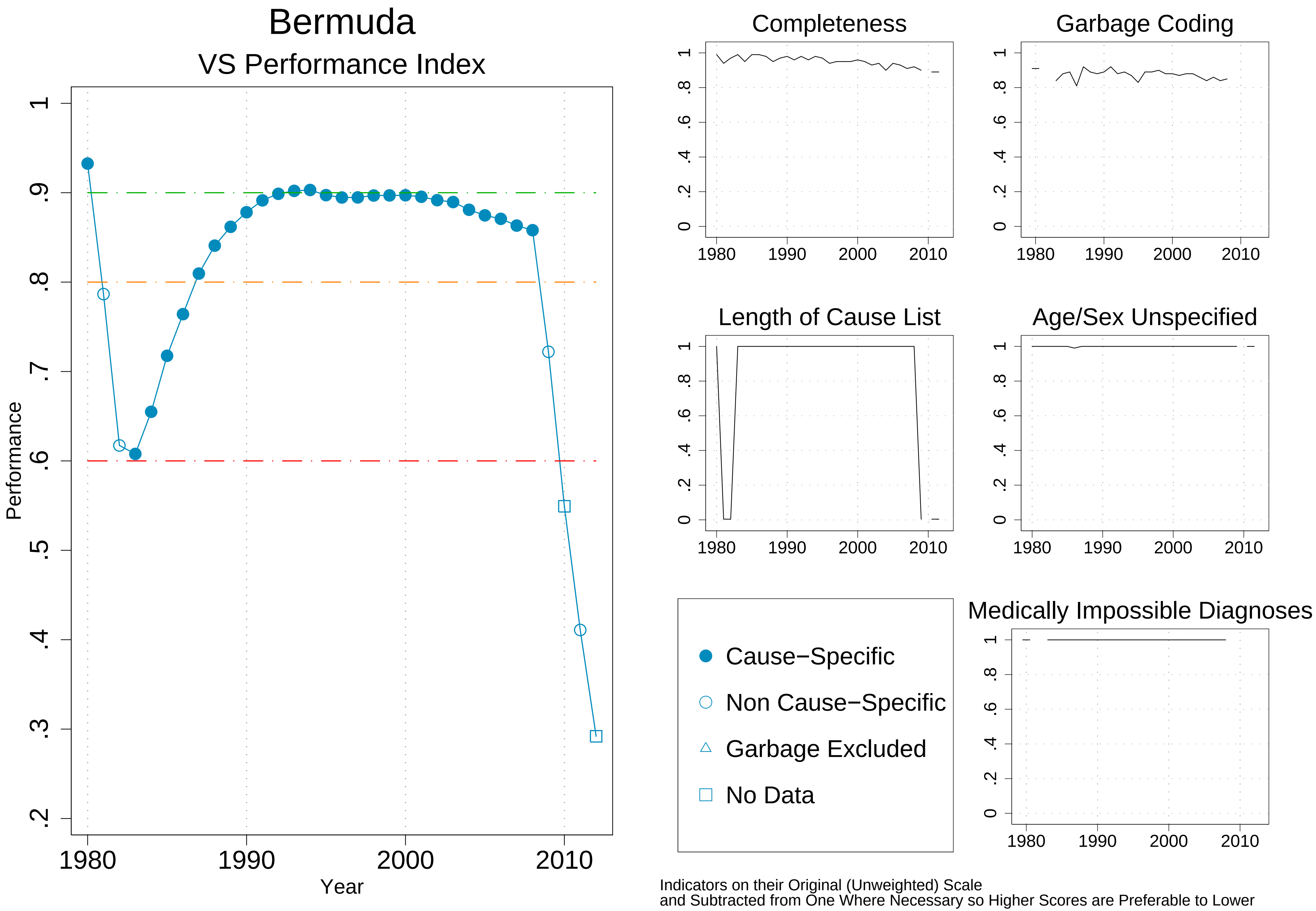

Indicators on their Original (Unweighted) Scale
and Subtracted from One Where Necessary so Higher Scores are Preferable to Lower

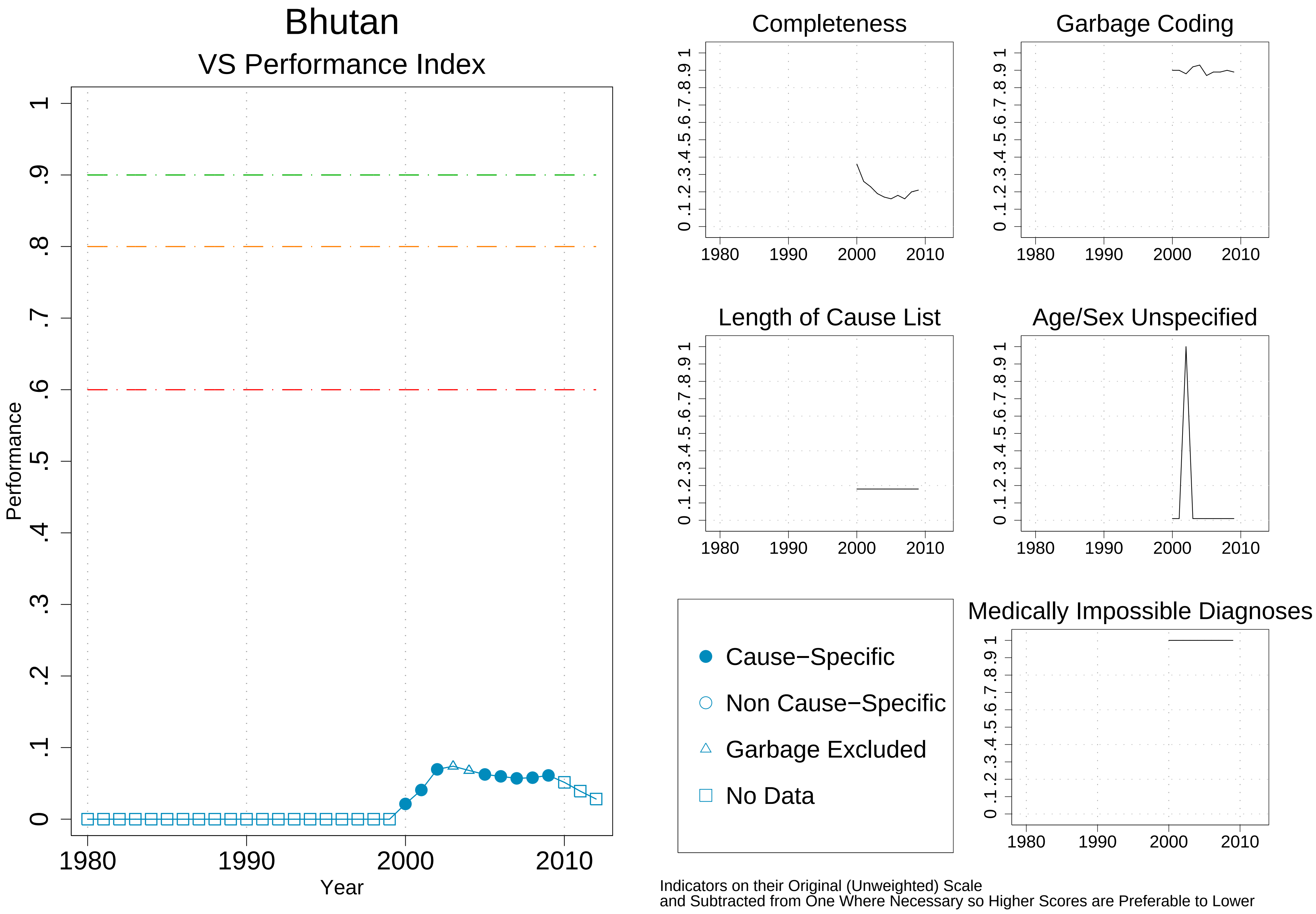
Bhutan
VS Performance Index
Performance
Year
Completeness
Garbage Coding
Length of Cause List
Age/Sex Unspecified
Medically Impossible Diagnoses
Cause−Specific
Non Cause−Specific
Garbage Excluded
No Data
Indicators on their Original (Unweighted) Scale
and Subtracted from One Where Necessary so Higher Scores are Preferable to Lower

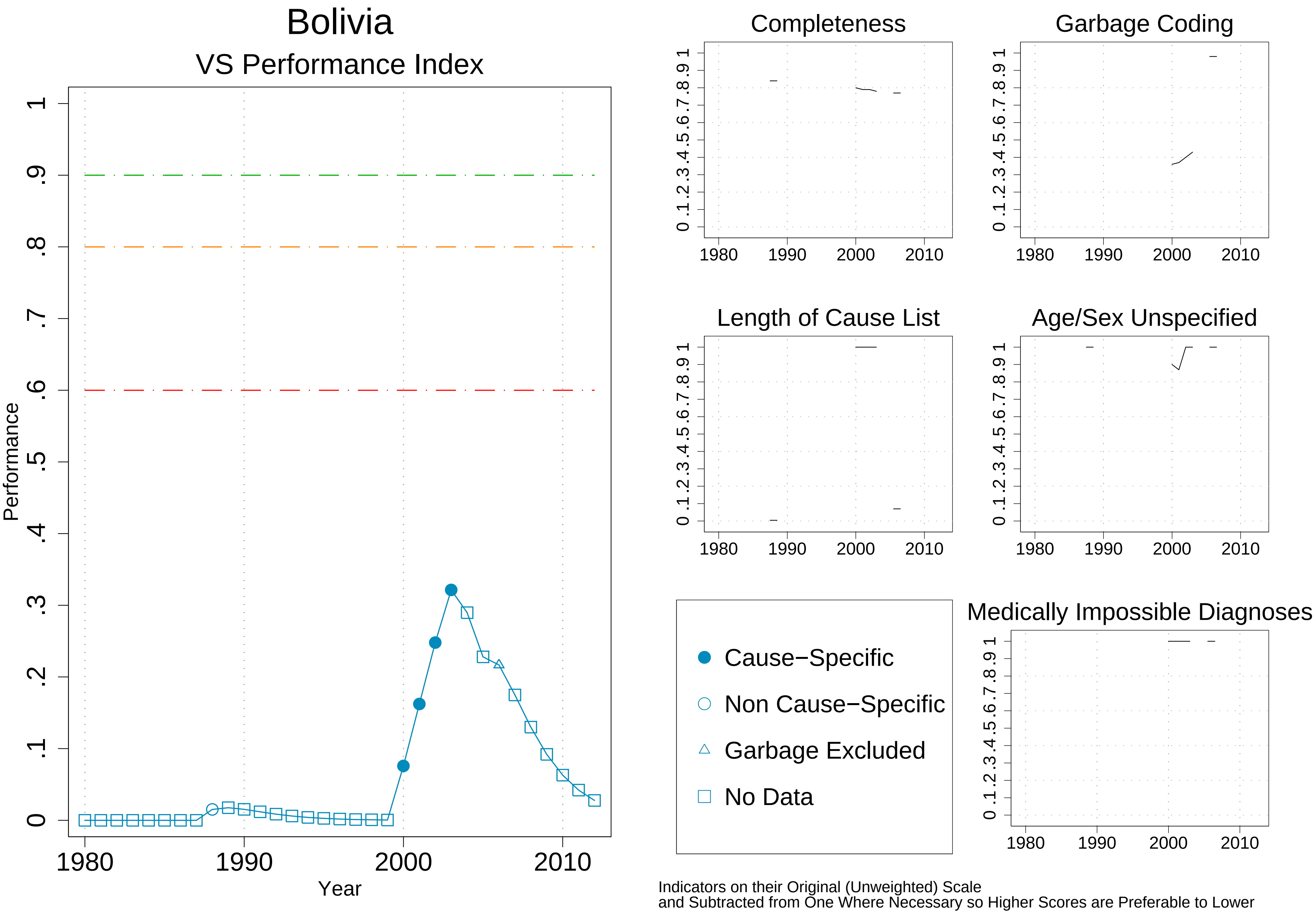

Indicators on their Original (Unweighted) Scale
and Subtracted from One Where Necessary so Higher Scores are Preferable to Lower

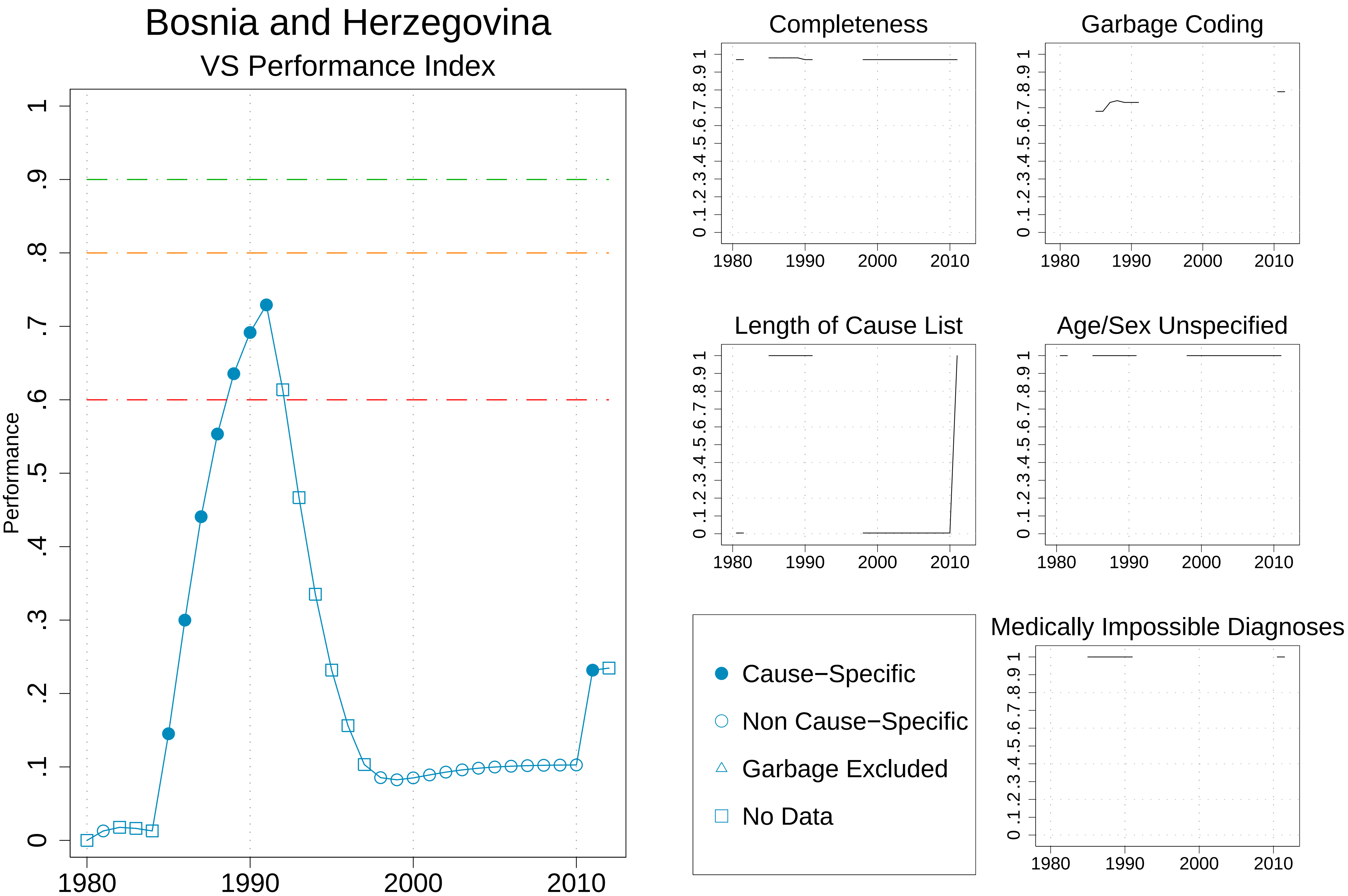

Indicators on their Original (Unweighted) Scale
and Subtracted from One Where Necessary so Higher Scores are Preferable to Lower

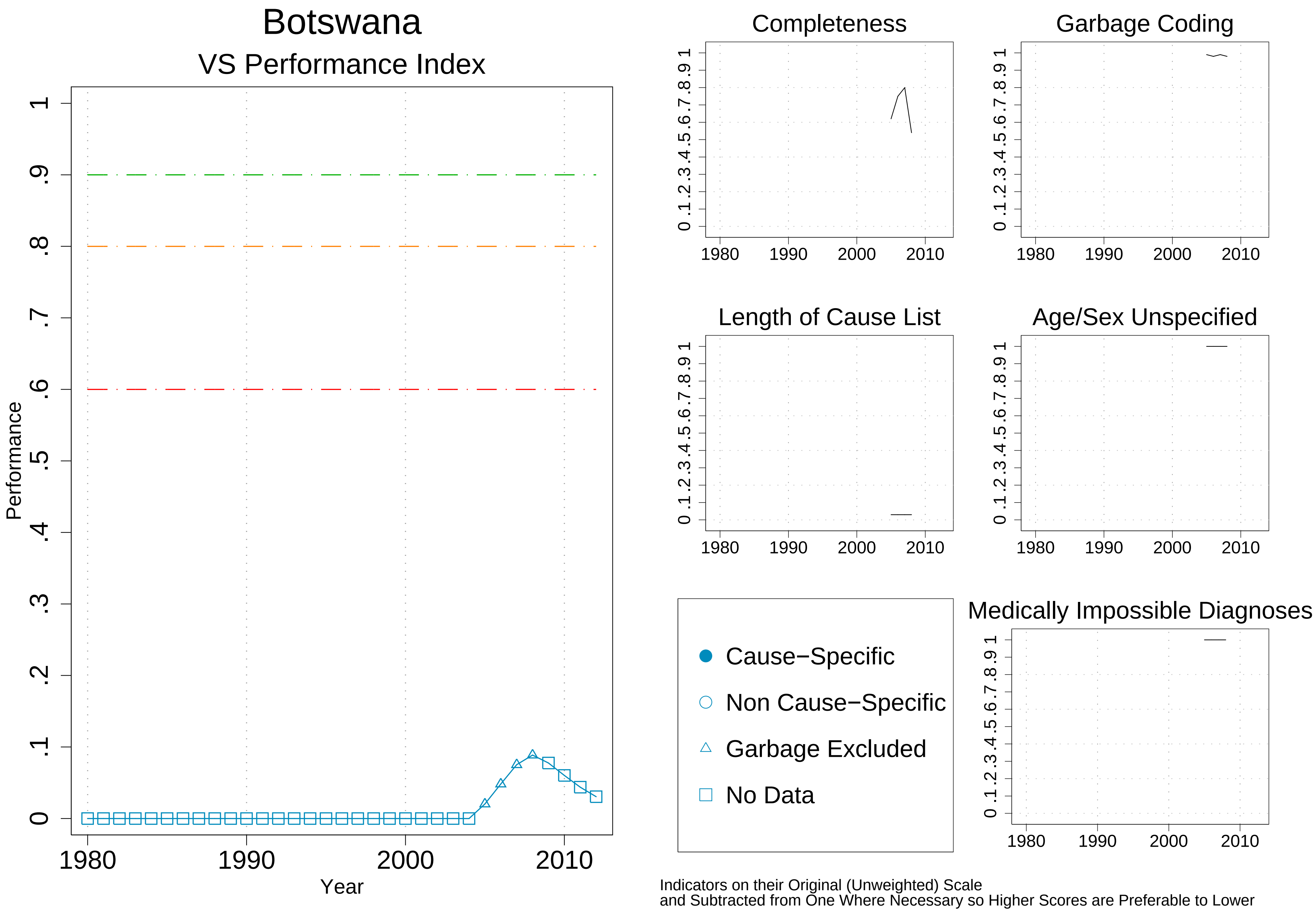

Indicators on their Original (Unweighted) Scale
and Subtracted from One Where Necessary so Higher Scores are Preferable to Lower

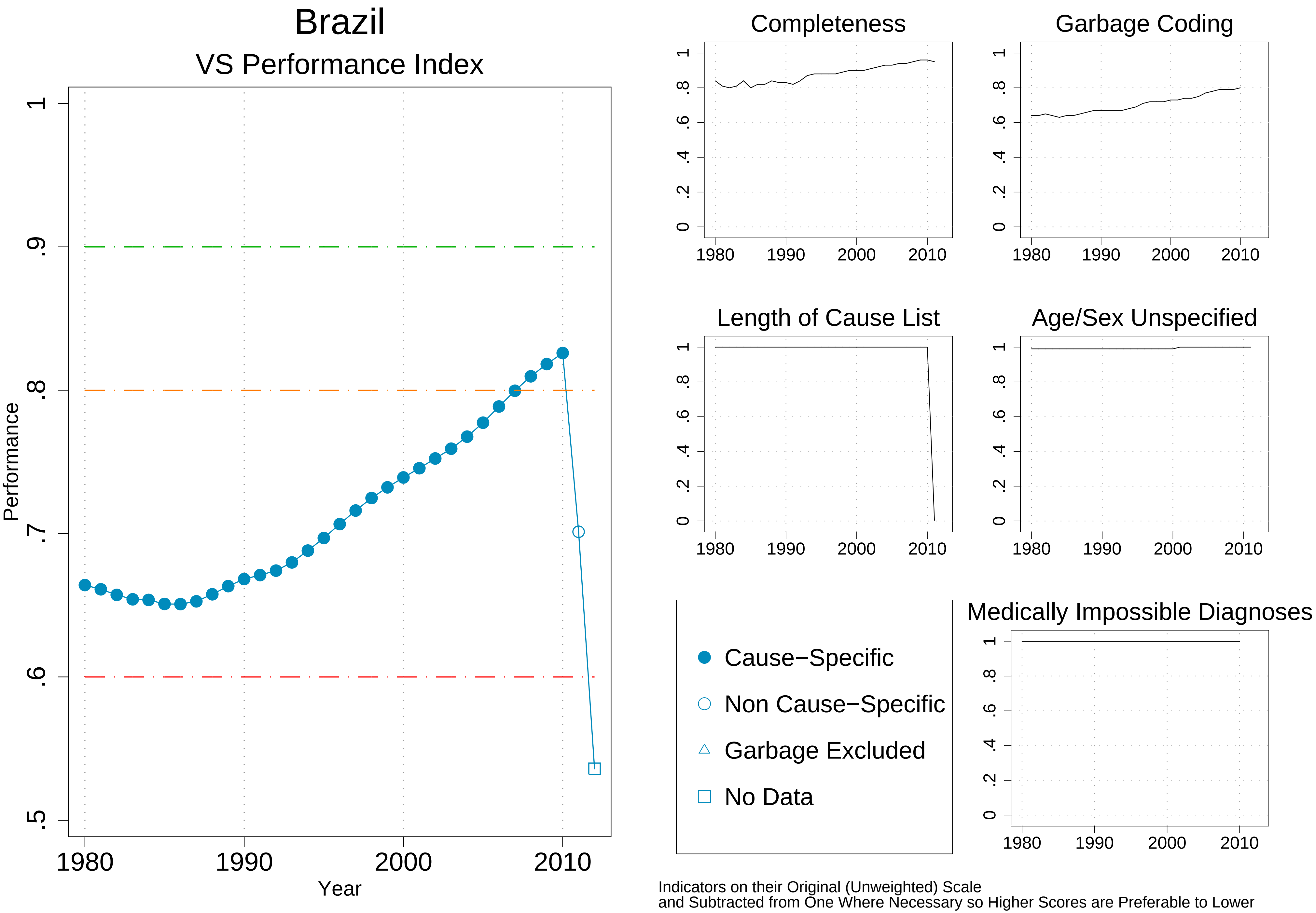
Brazil
VS Performance Index
Performance
Year
.5
.6
.7
.8
.9
1
1980
1990
2000
2010
Completeness
Garbage Coding
Length of Cause List
Age/Sex Unspecified
Medically Impossible Diagnoses
0
.2
.4
.6
.8
1
Cause−Specific
Non Cause−Specific
Garbage Excluded
No Data
Indicators on their Original (Unweighted) Scale
and Subtracted from One Where Necessary so Higher Scores are Preferable to Lower

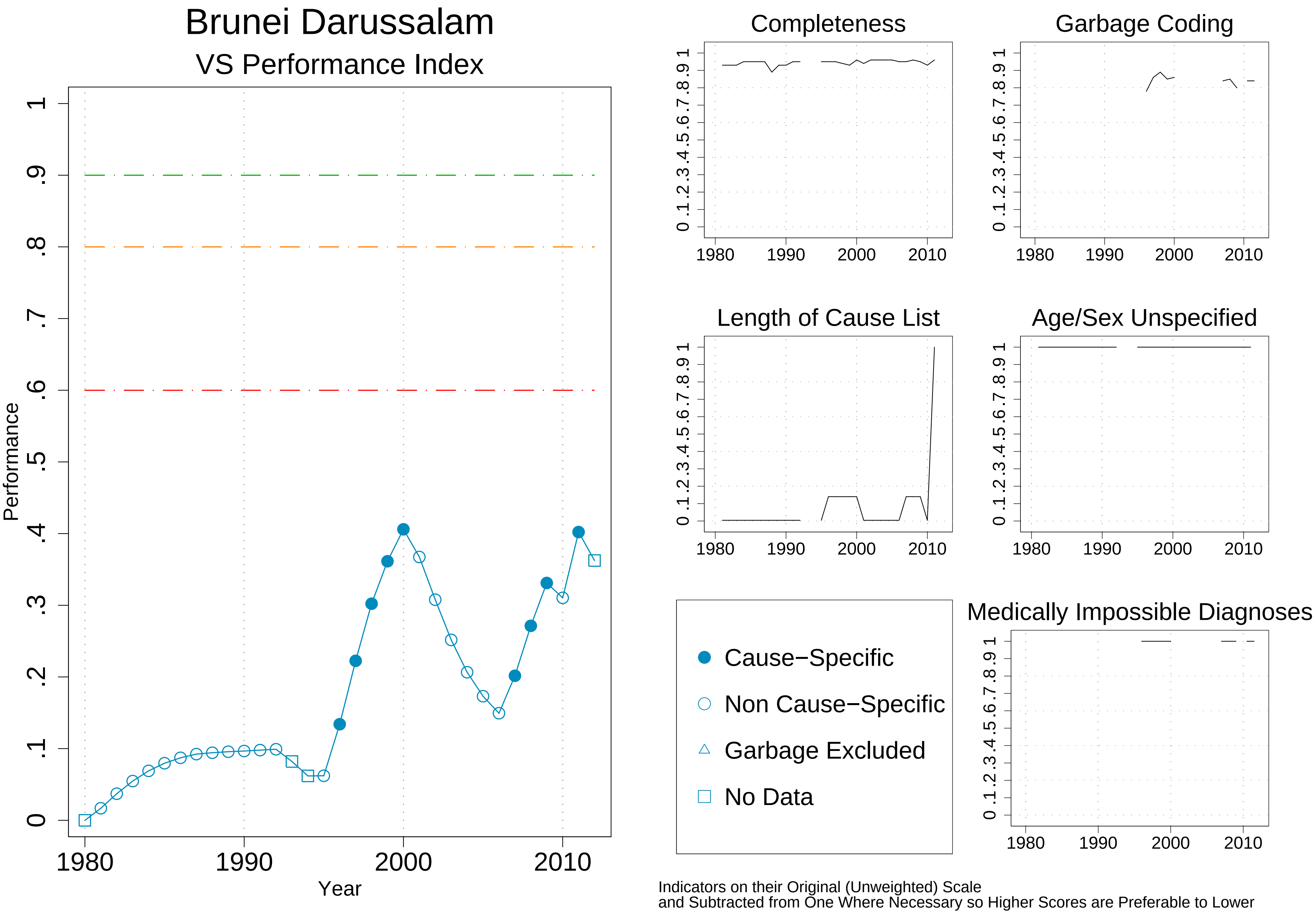

Indicators on their Original (Unweighted) Scale
and Subtracted from One Where Necessary so Higher Scores are Preferable to Lower

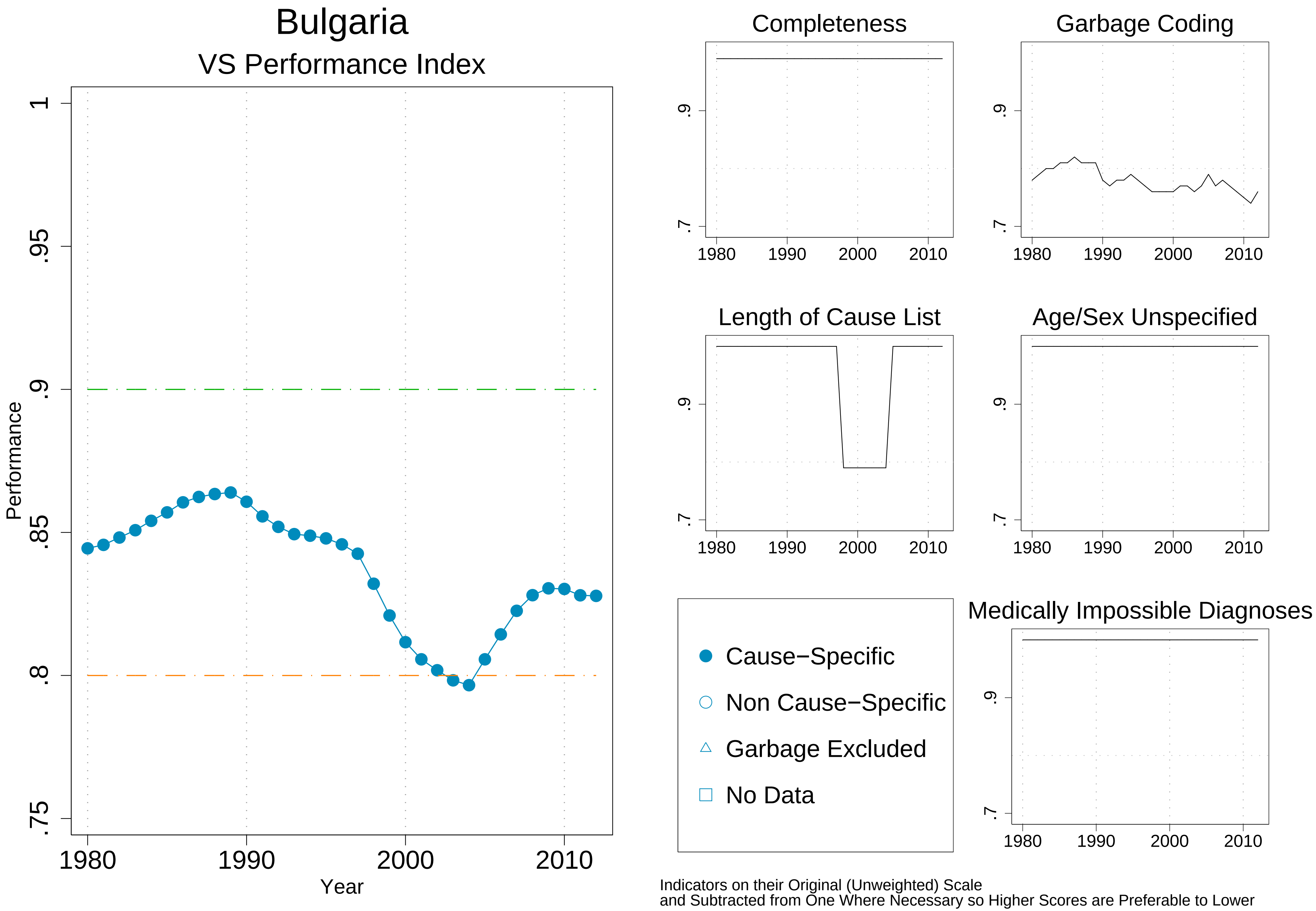

Indicators on their Original (Unweighted) Scale
and Subtracted from One Where Necessary so Higher Scores are Preferable to Lower

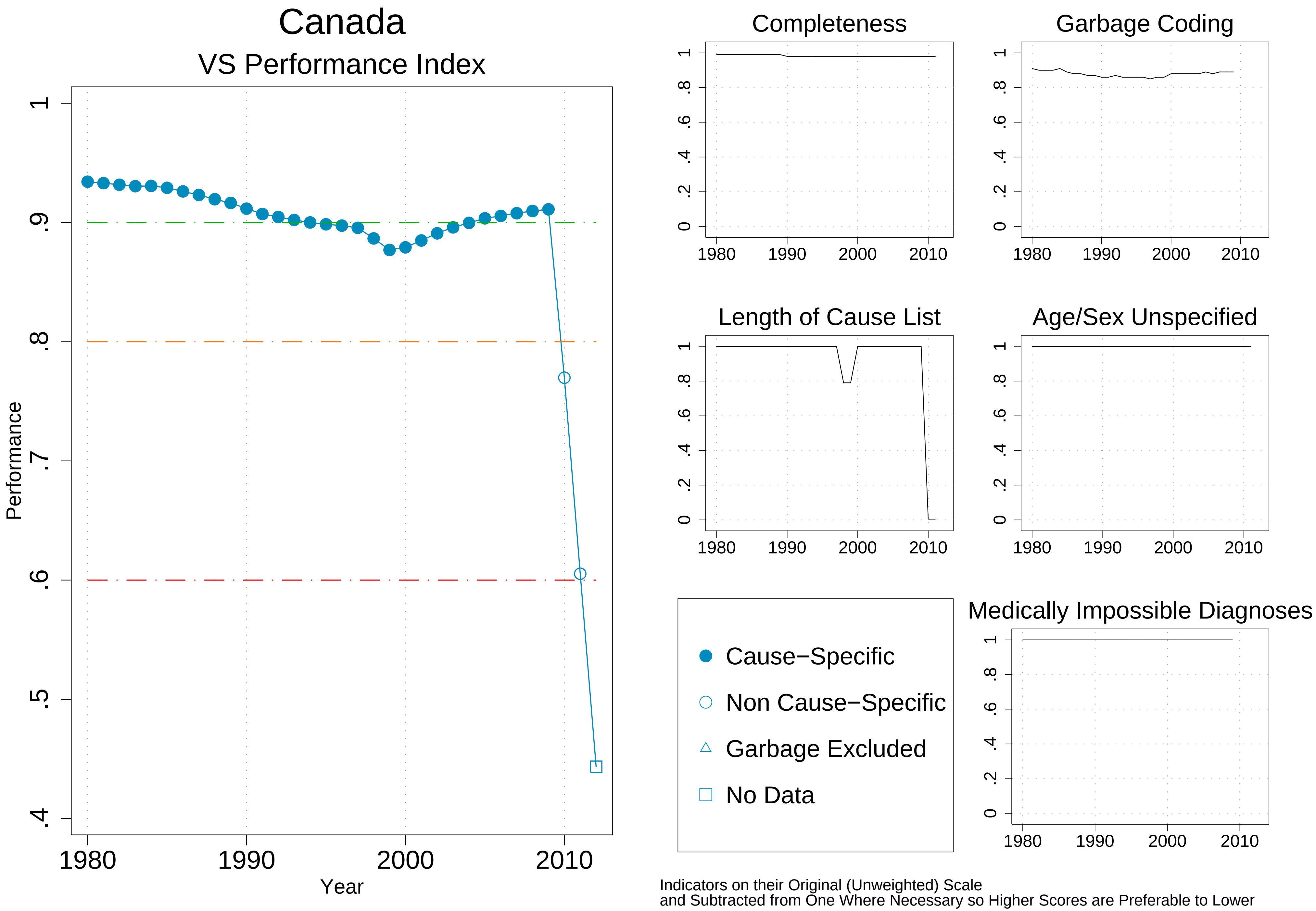

Indicators on their Original (Unweighted) Scale
and Subtracted from One Where Necessary so Higher Scores are Preferable to Lower

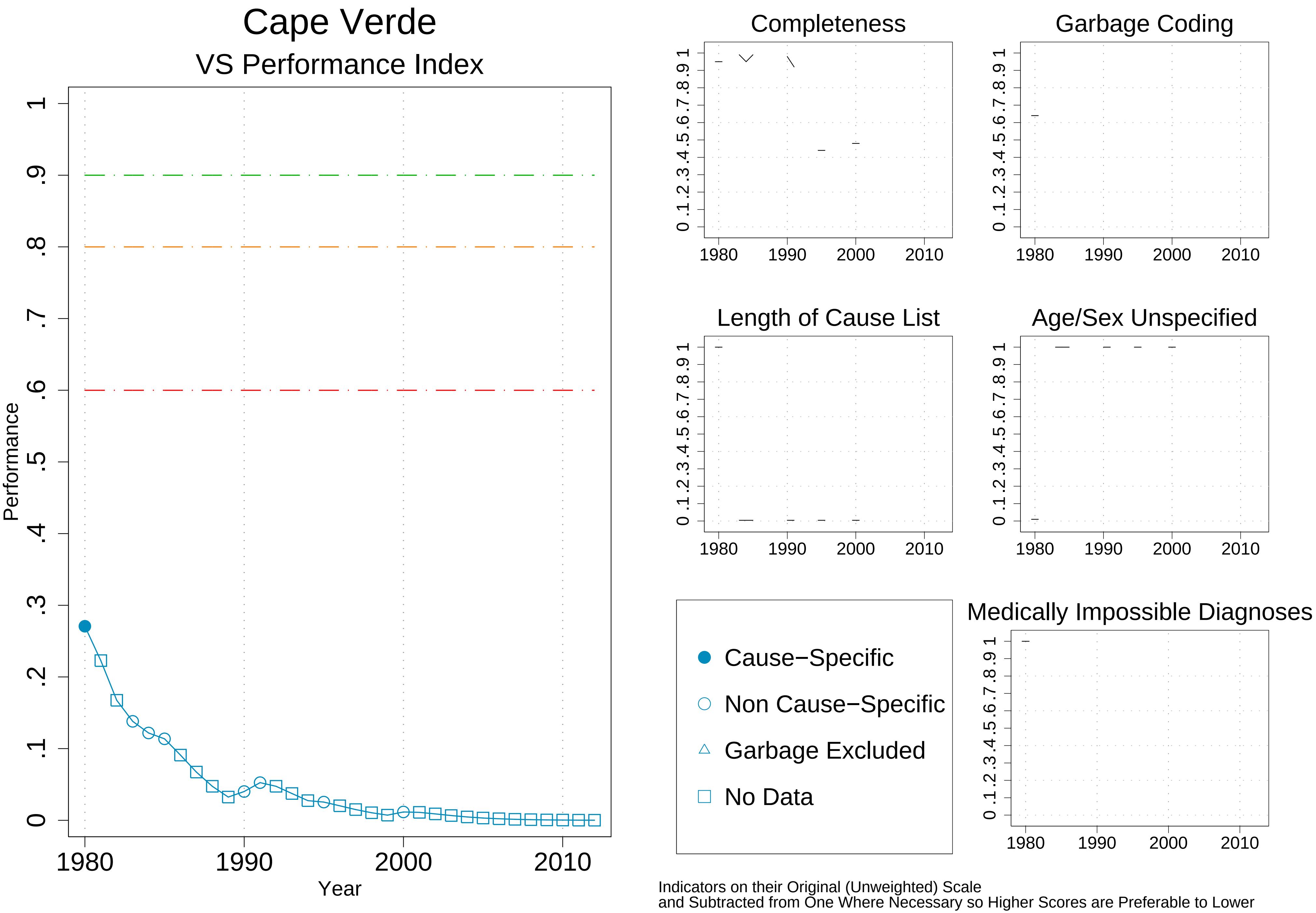

Indicators on their Original (Unweighted) Scale
and Subtracted from One Where Necessary so Higher Scores are Preferable to Lower

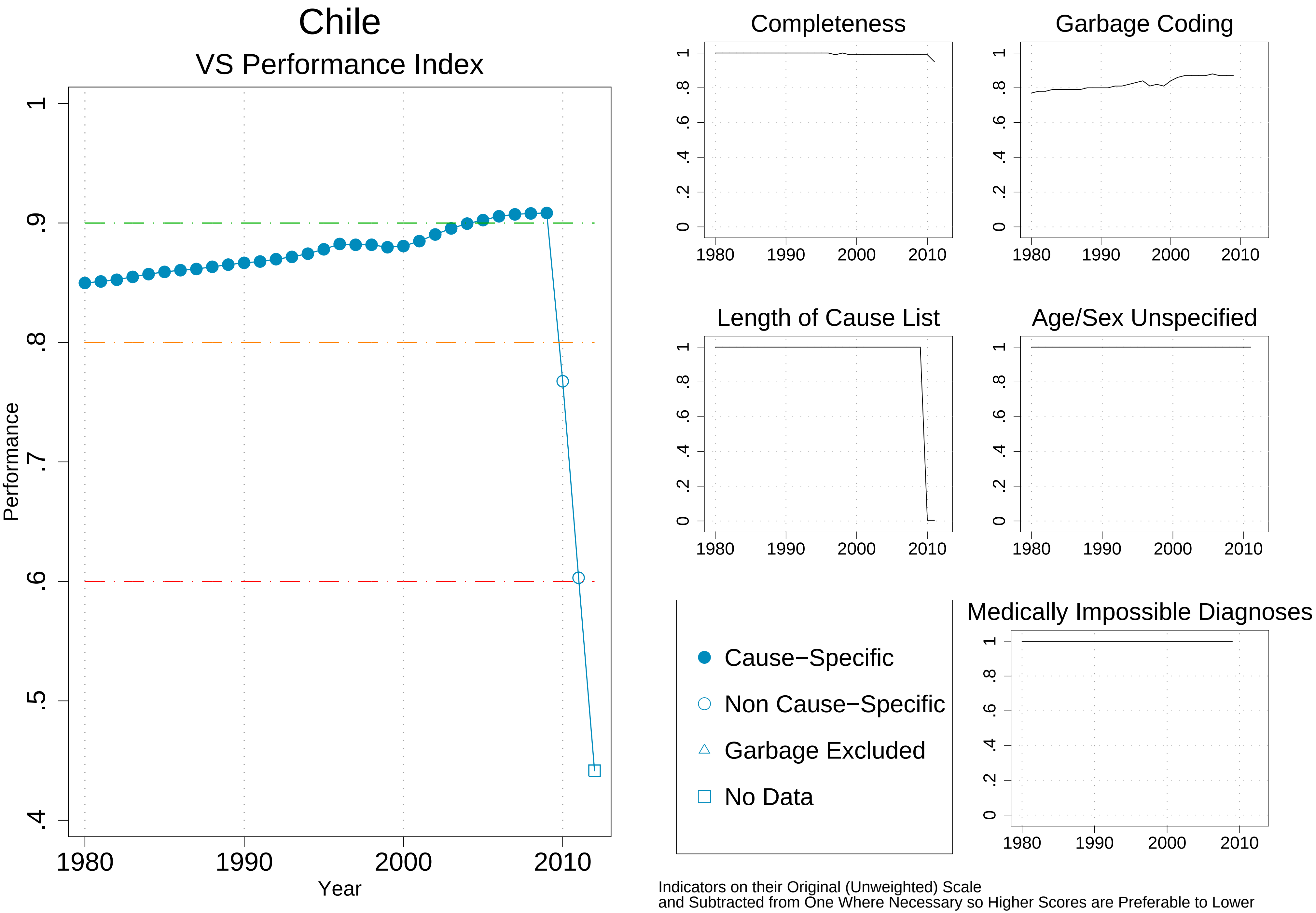

Indicators on their Original (Unweighted) Scale
and Subtracted from One Where Necessary so Higher Scores are Preferable to Lower

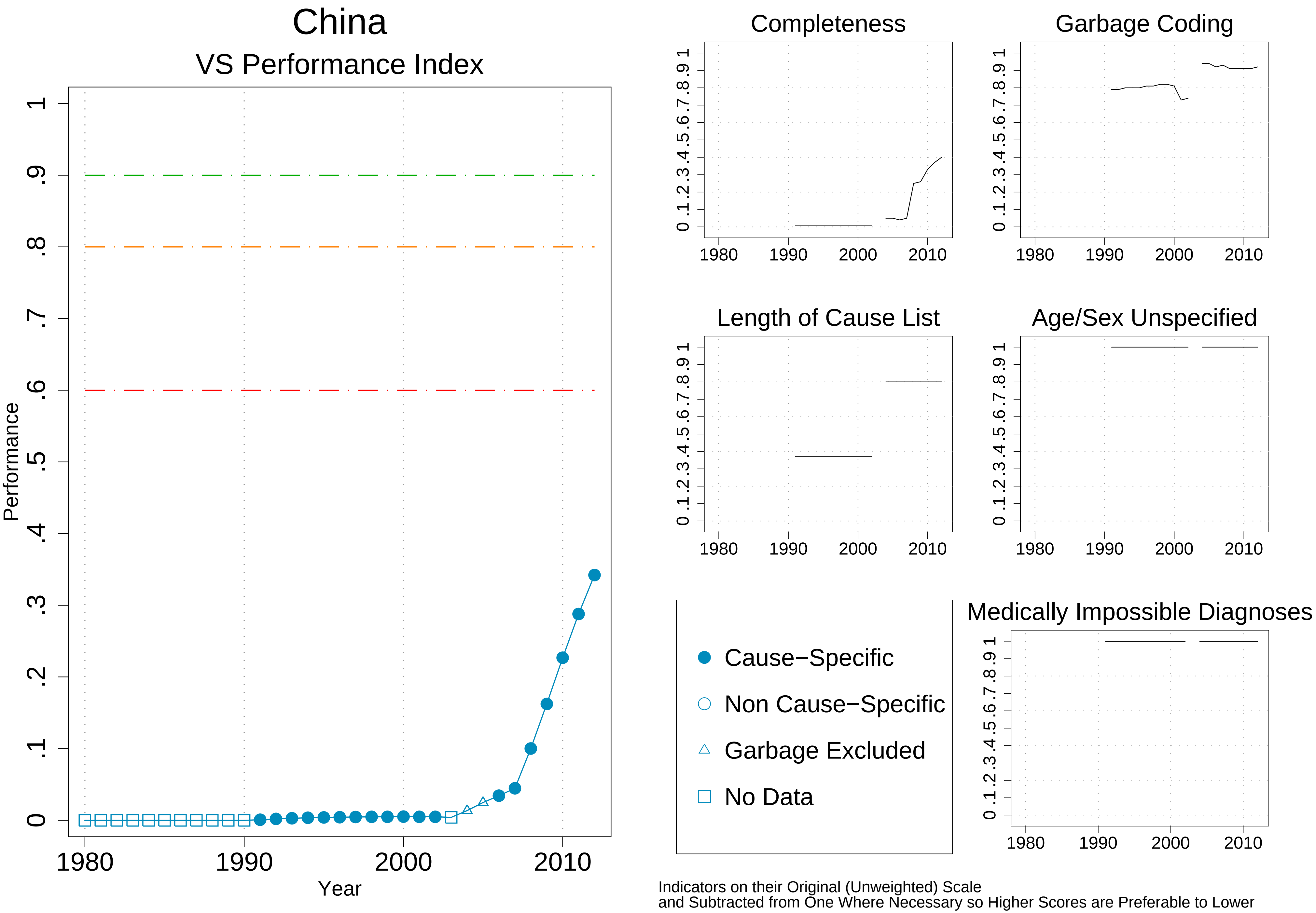

Indicators on their Original (Unweighted) Scale
and Subtracted from One Where Necessary so Higher Scores are Preferable to Lower

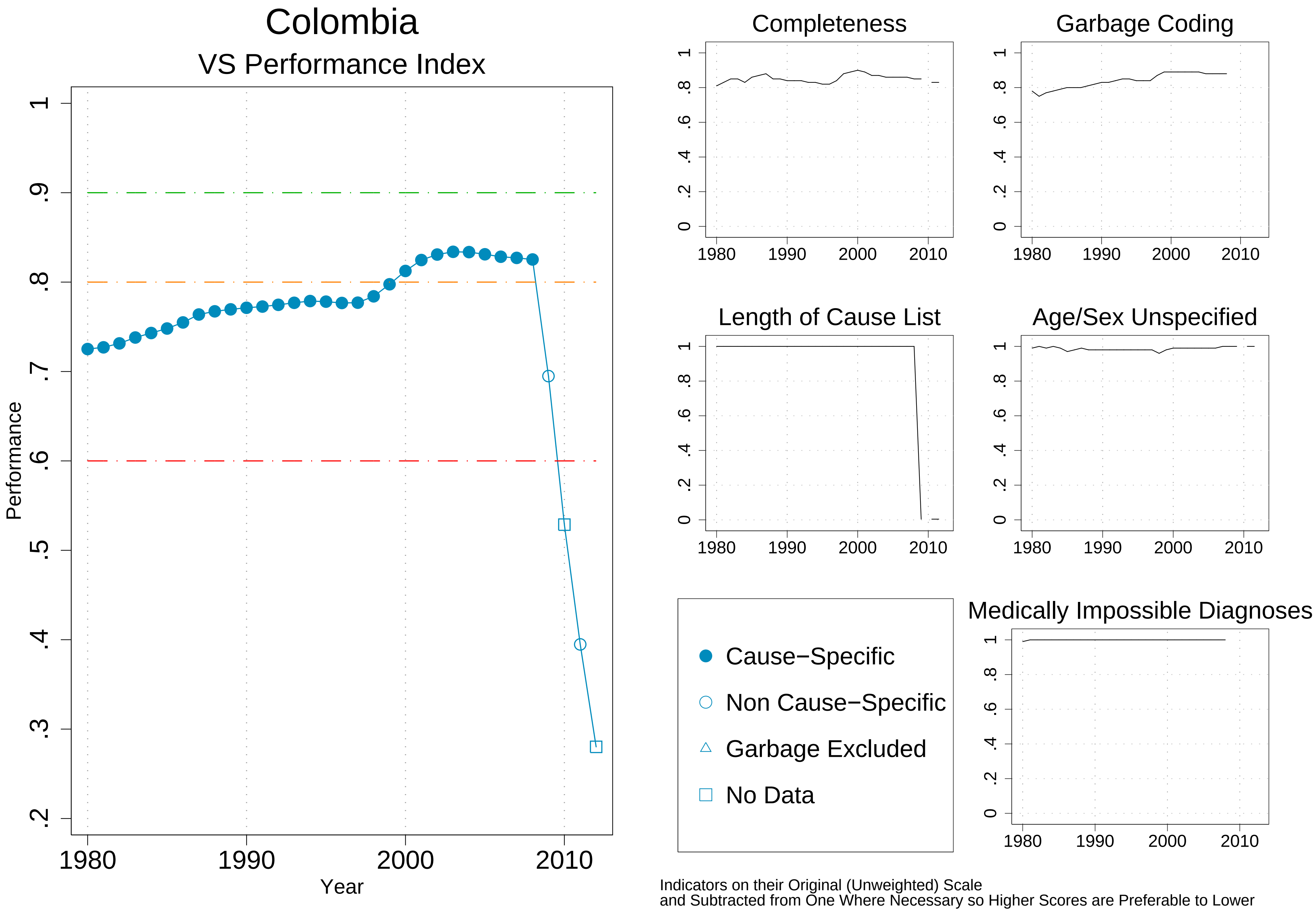

Indicators on their Original (Unweighted) Scale
and Subtracted from One Where Necessary so Higher Scores are Preferable to Lower

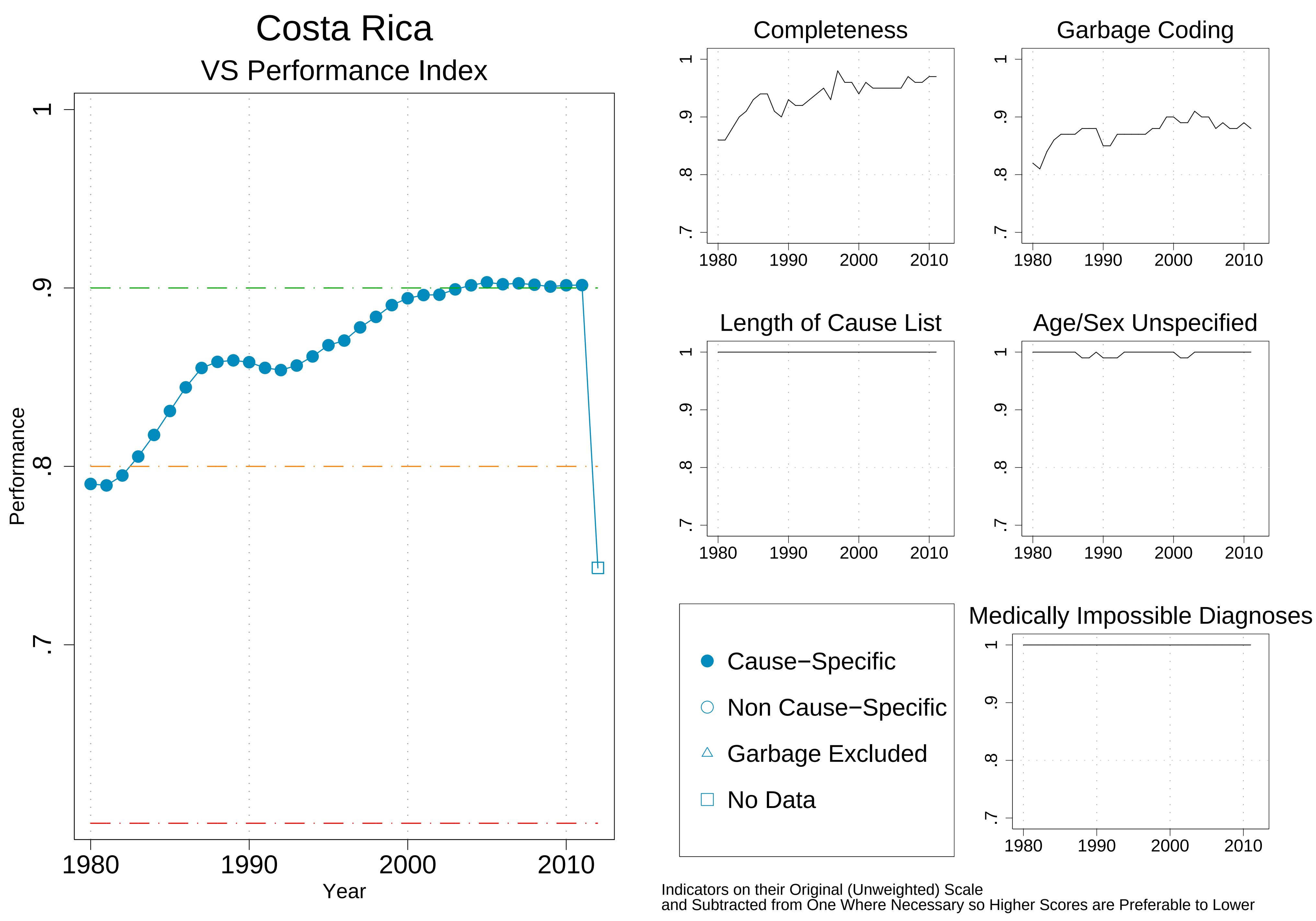

Costa Rica
VS Performance Index
Performance
Year
Completeness
Garbage Coding
Length of Cause List
Age/Sex Unspecified
Medically Impossible Diagnoses
Cause−Specific
Non Cause−Specific
Garbage Excluded
No Data
Indicators on their Original (Unweighted) Scale
and Subtracted from One Where Necessary so Higher Scores are Preferable to Lower

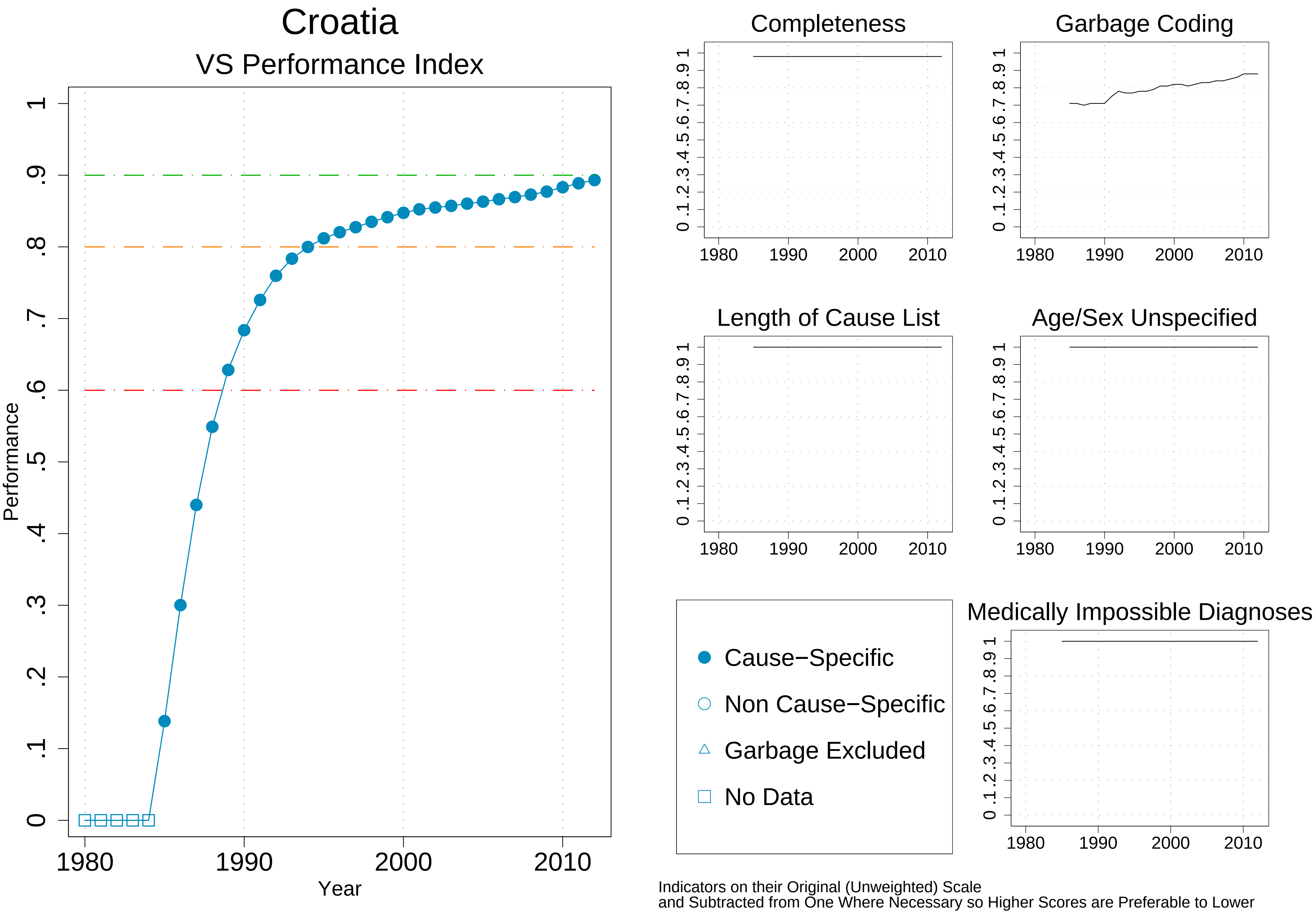

Indicators on their Original (Unweighted) Scale
and Subtracted from One Where Necessary so Higher Scores are Preferable to Lower

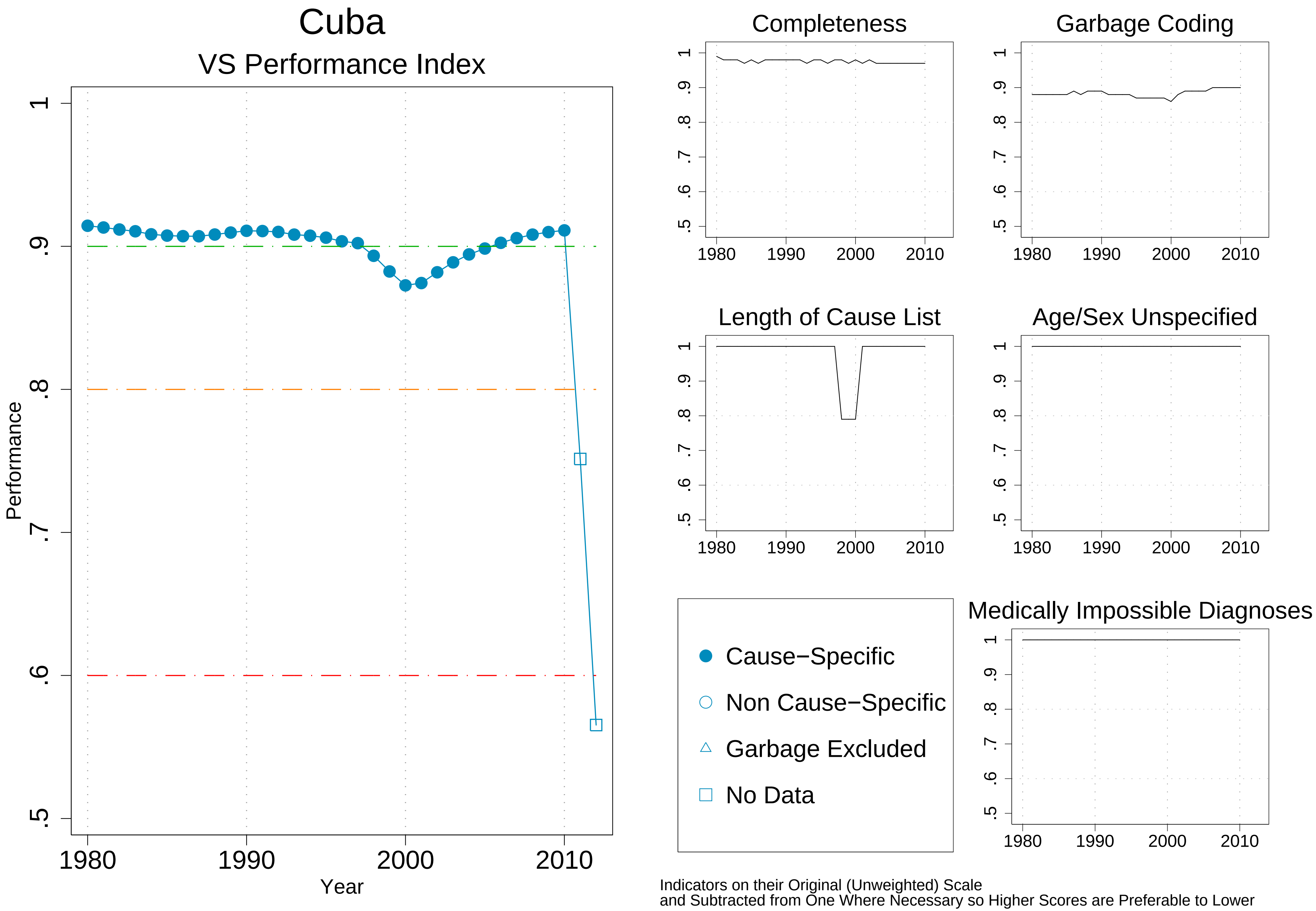

Indicators on their Original (Unweighted) Scale
and Subtracted from One Where Necessary so Higher Scores are Preferable to Lower

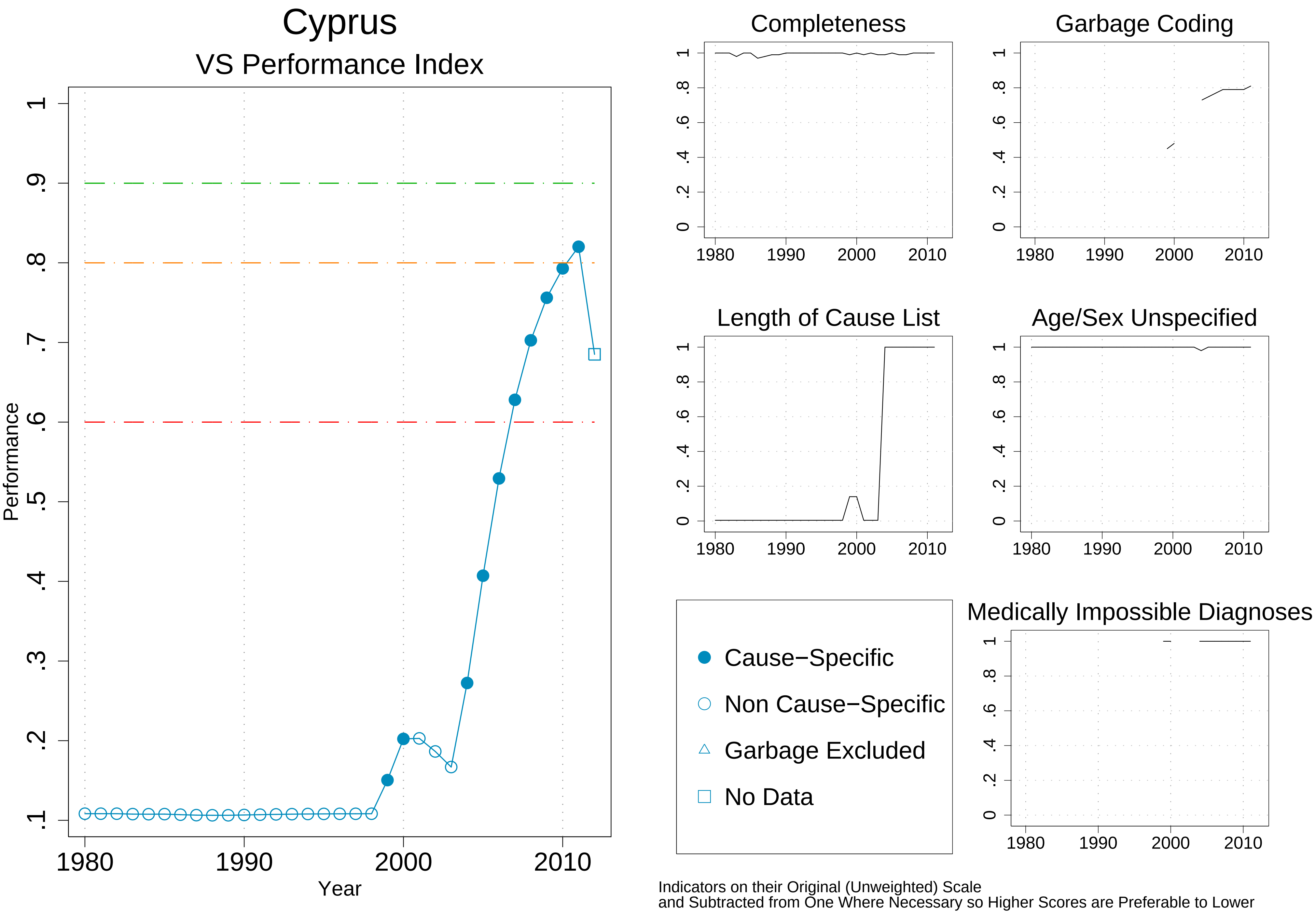

Indicators on their Original (Unweighted) Scale
and Subtracted from One Where Necessary so Higher Scores are Preferable to Lower

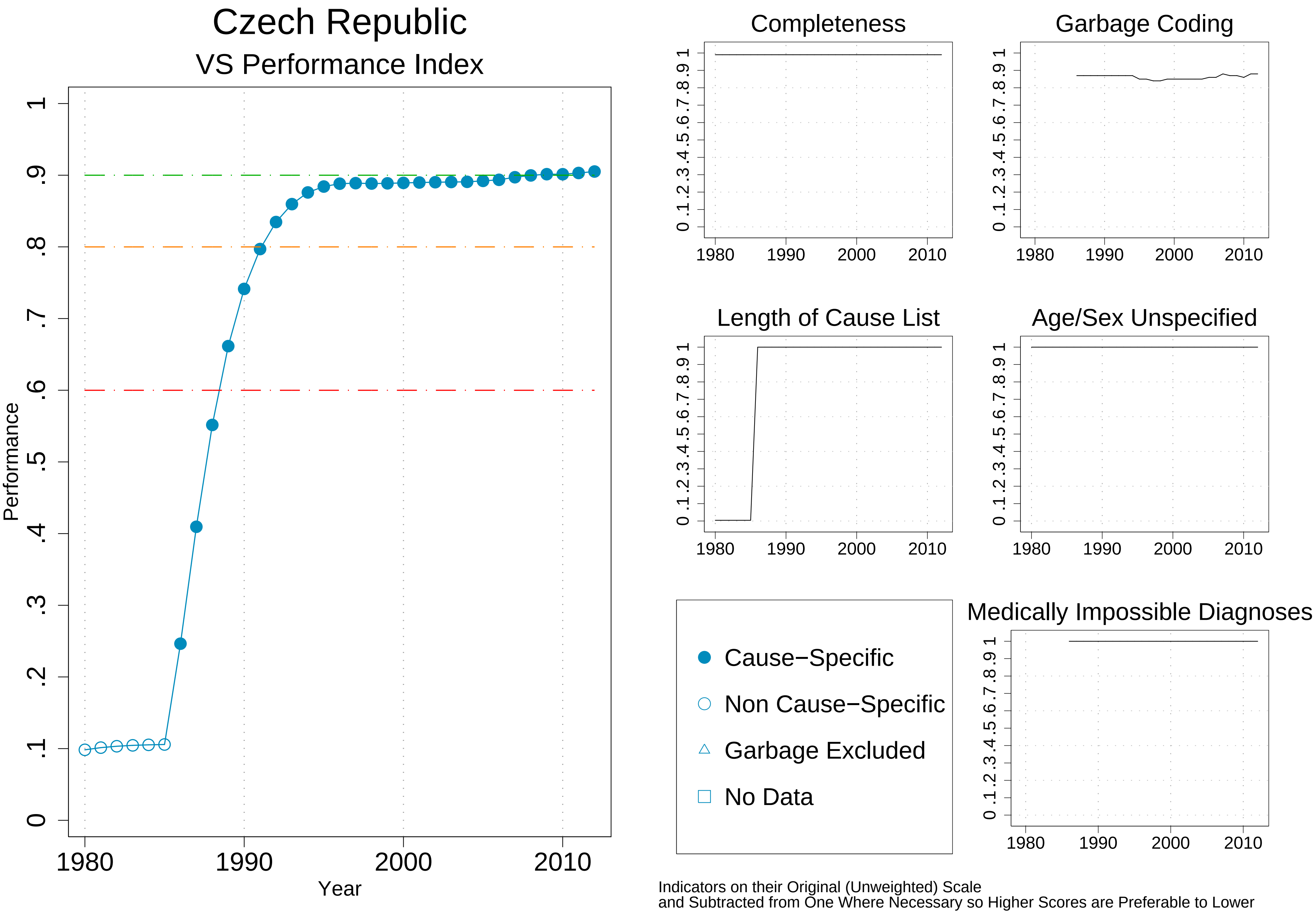

Indicators on their Original (Unweighted) Scale
and Subtracted from One Where Necessary so Higher Scores are Preferable to Lower

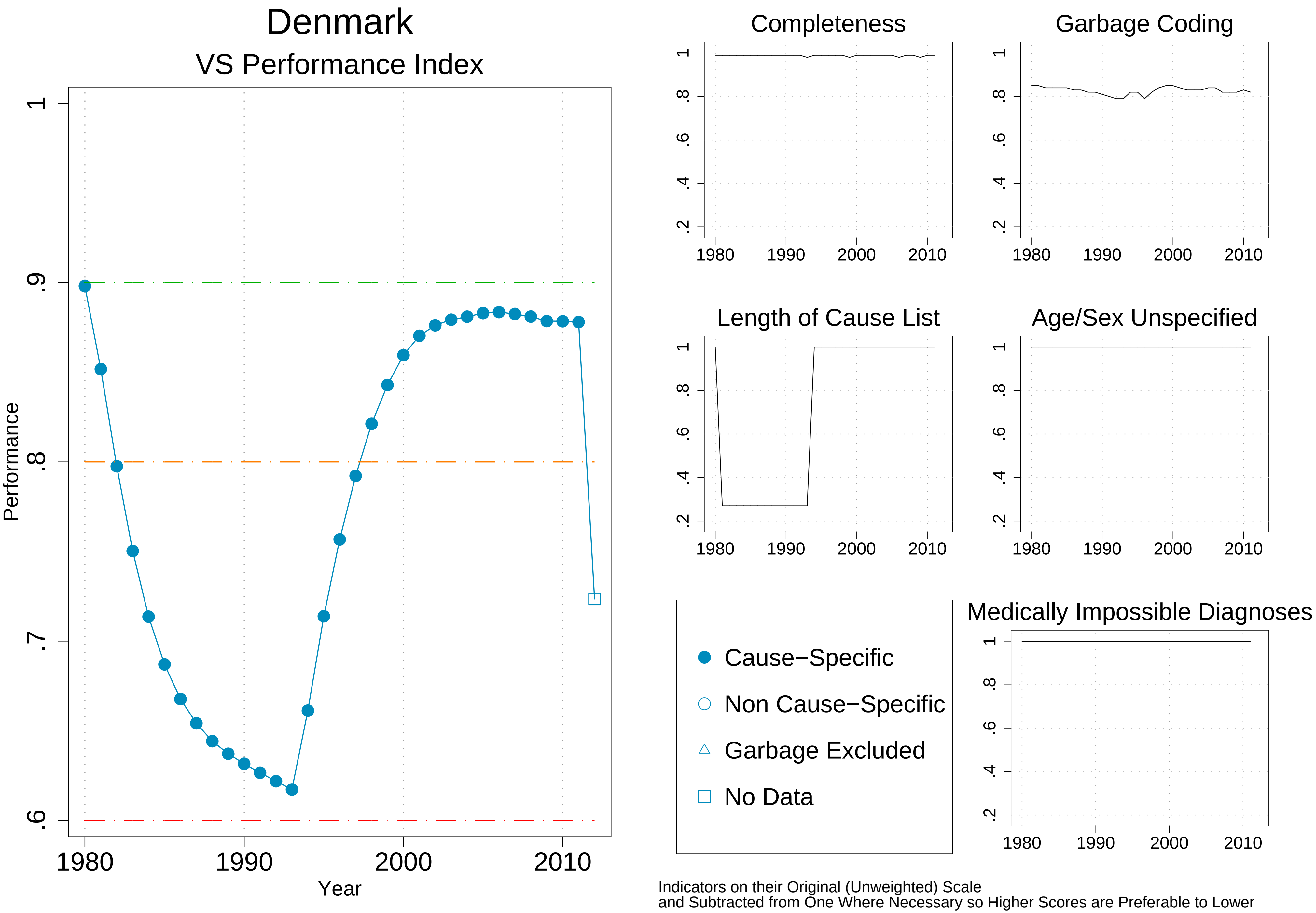

Indicators on their Original (Unweighted) Scale
and Subtracted from One Where Necessary so Higher Scores are Preferable to Lower

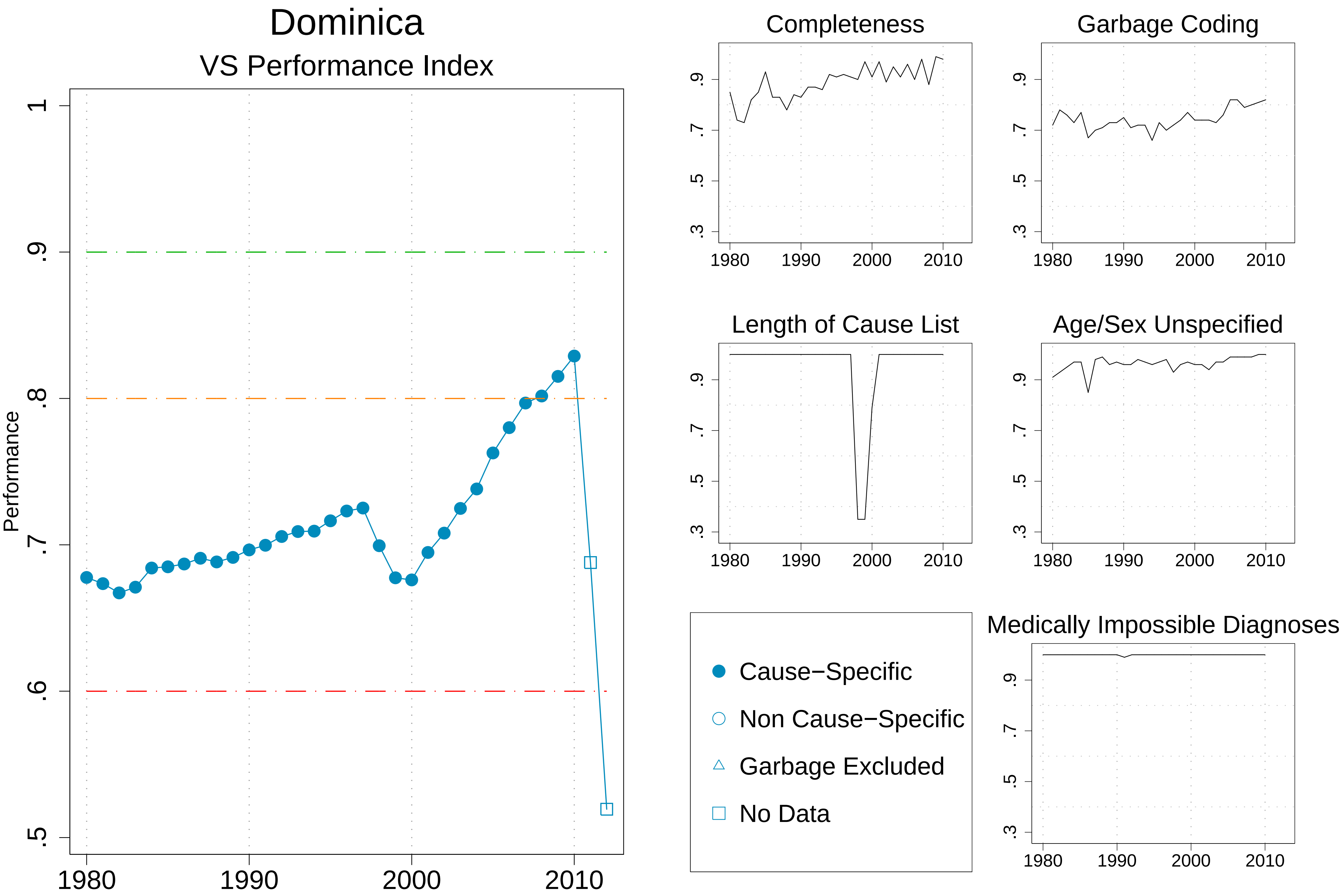

Indicators on their Original (Unweighted) Scale
and Subtracted from One Where Necessary so Higher Scores are Preferable to Lower

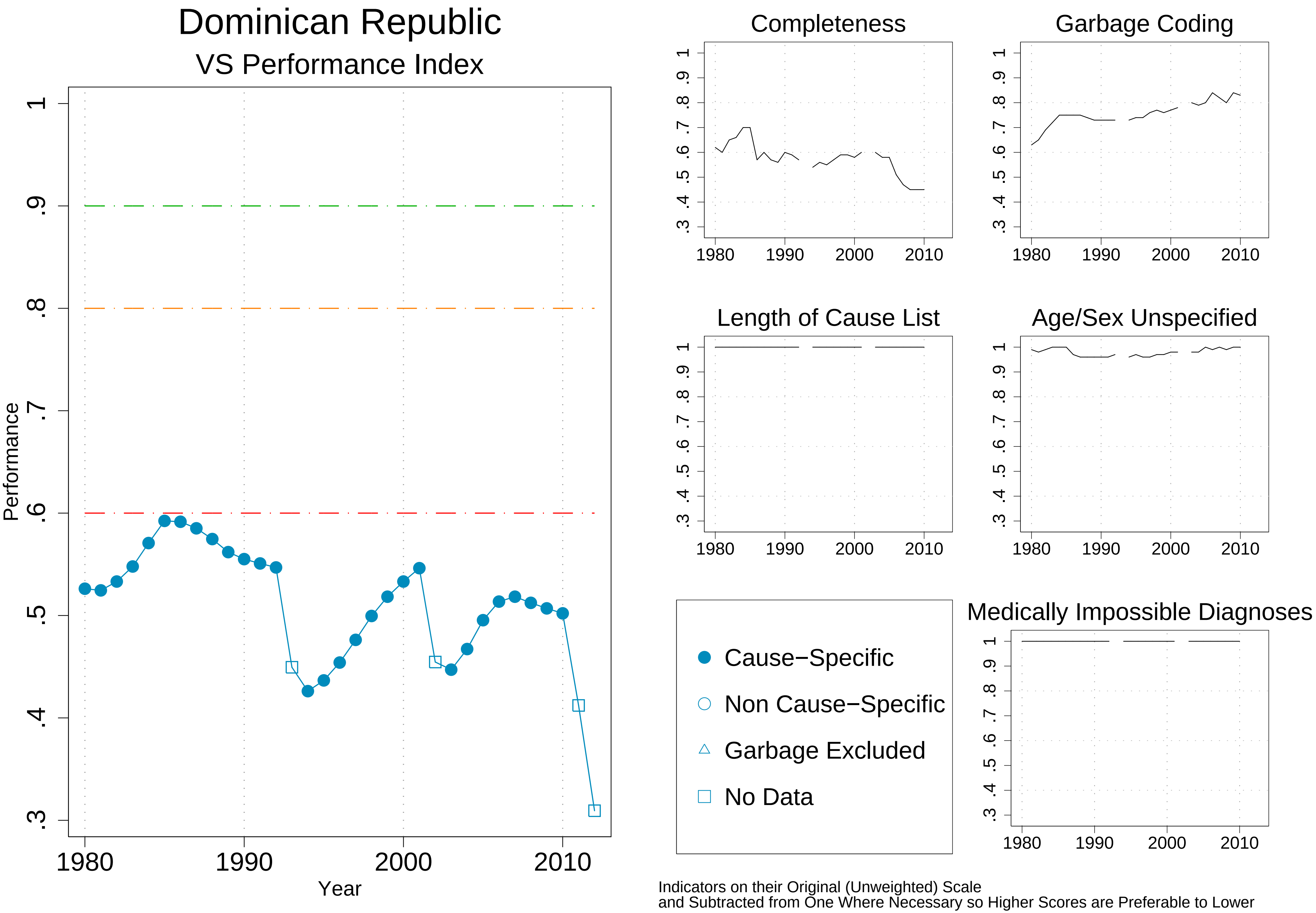

Indicators on their Original (Unweighted) Scale
and Subtracted from One Where Necessary so Higher Scores are Preferable to Lower

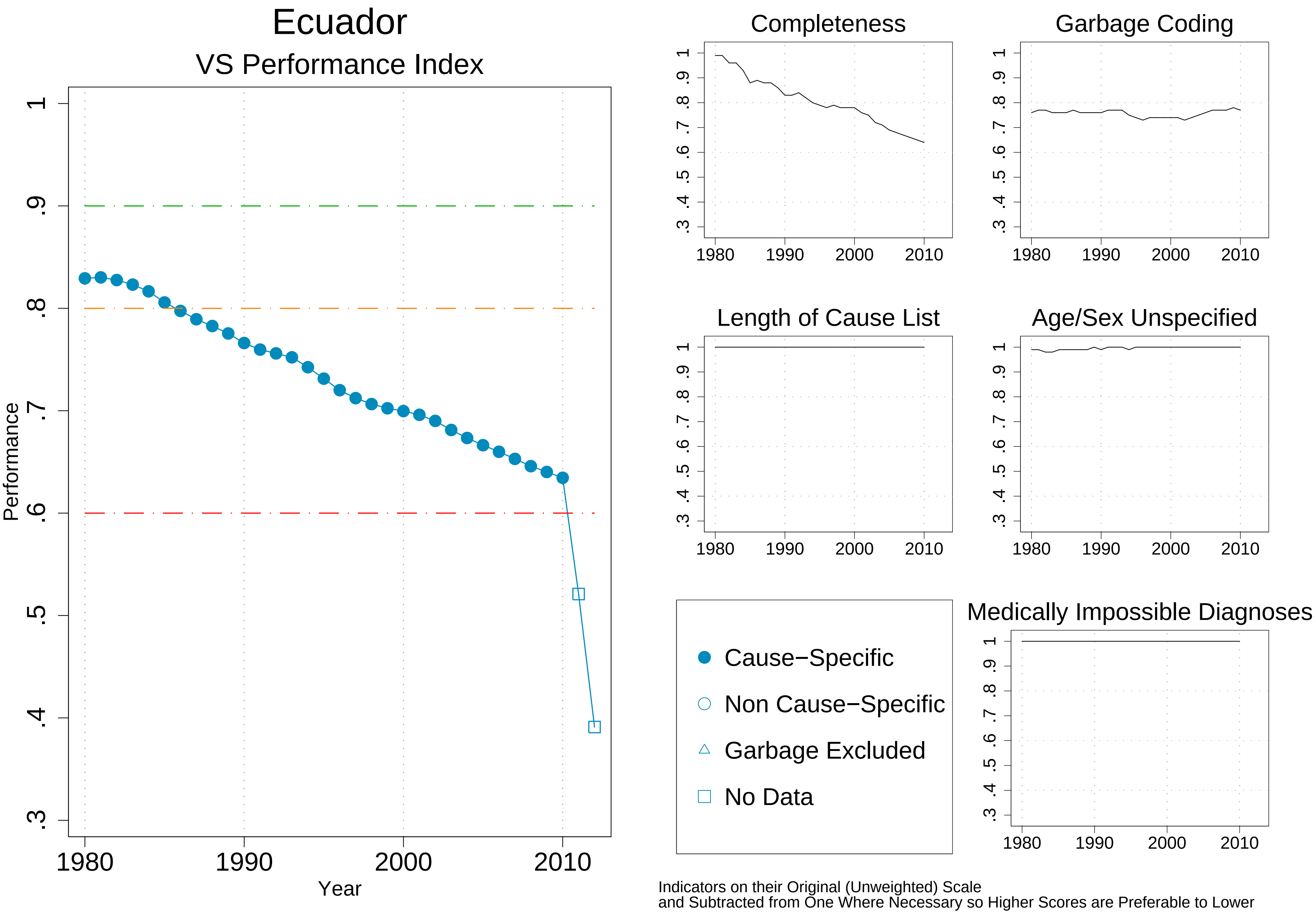

Indicators on their Original (Unweighted) Scale
and Subtracted from One Where Necessary so Higher Scores are Preferable to Lower

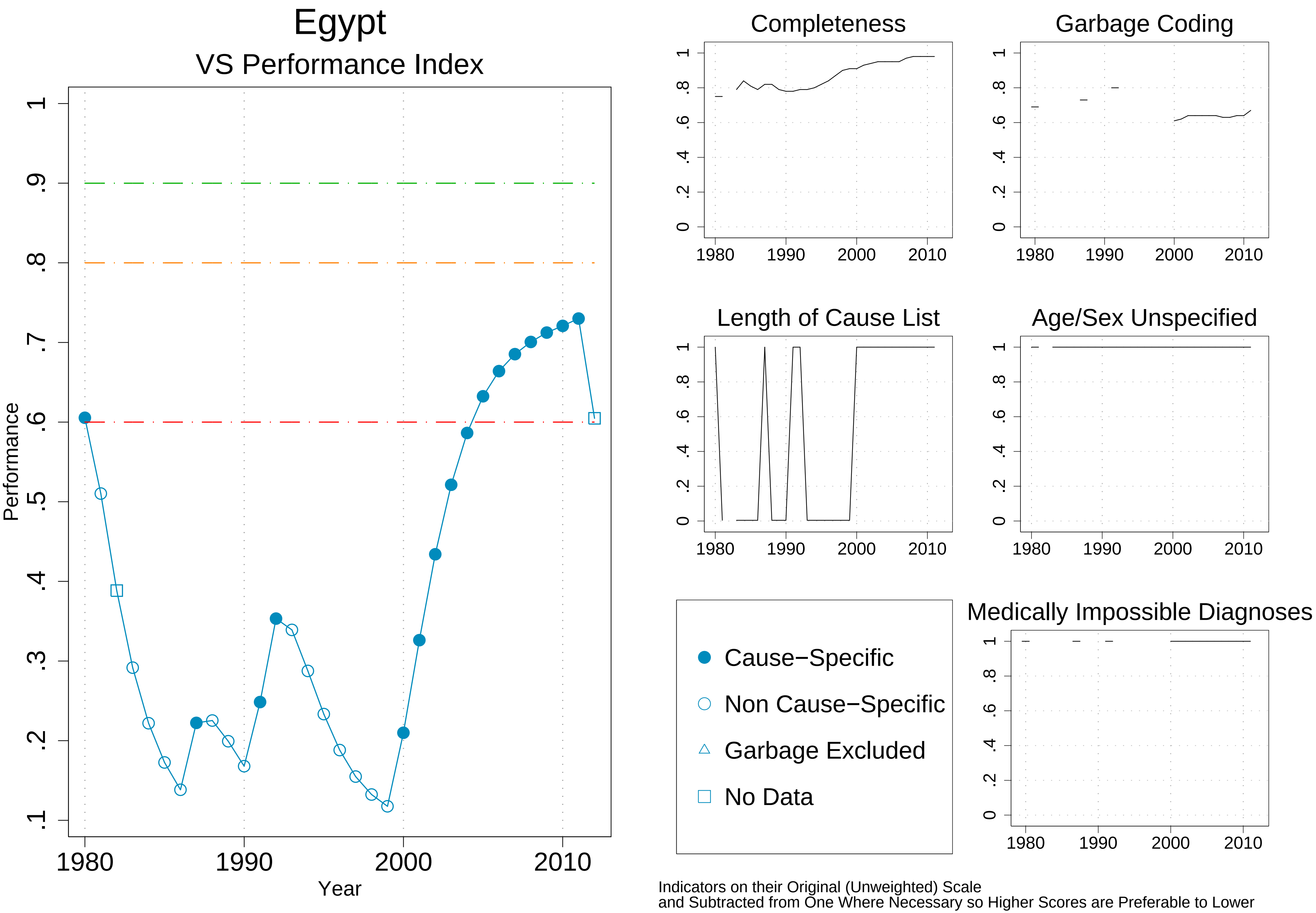

Indicators on their Original (Unweighted) Scale
and Subtracted from One Where Necessary so Higher Scores are Preferable to Lower

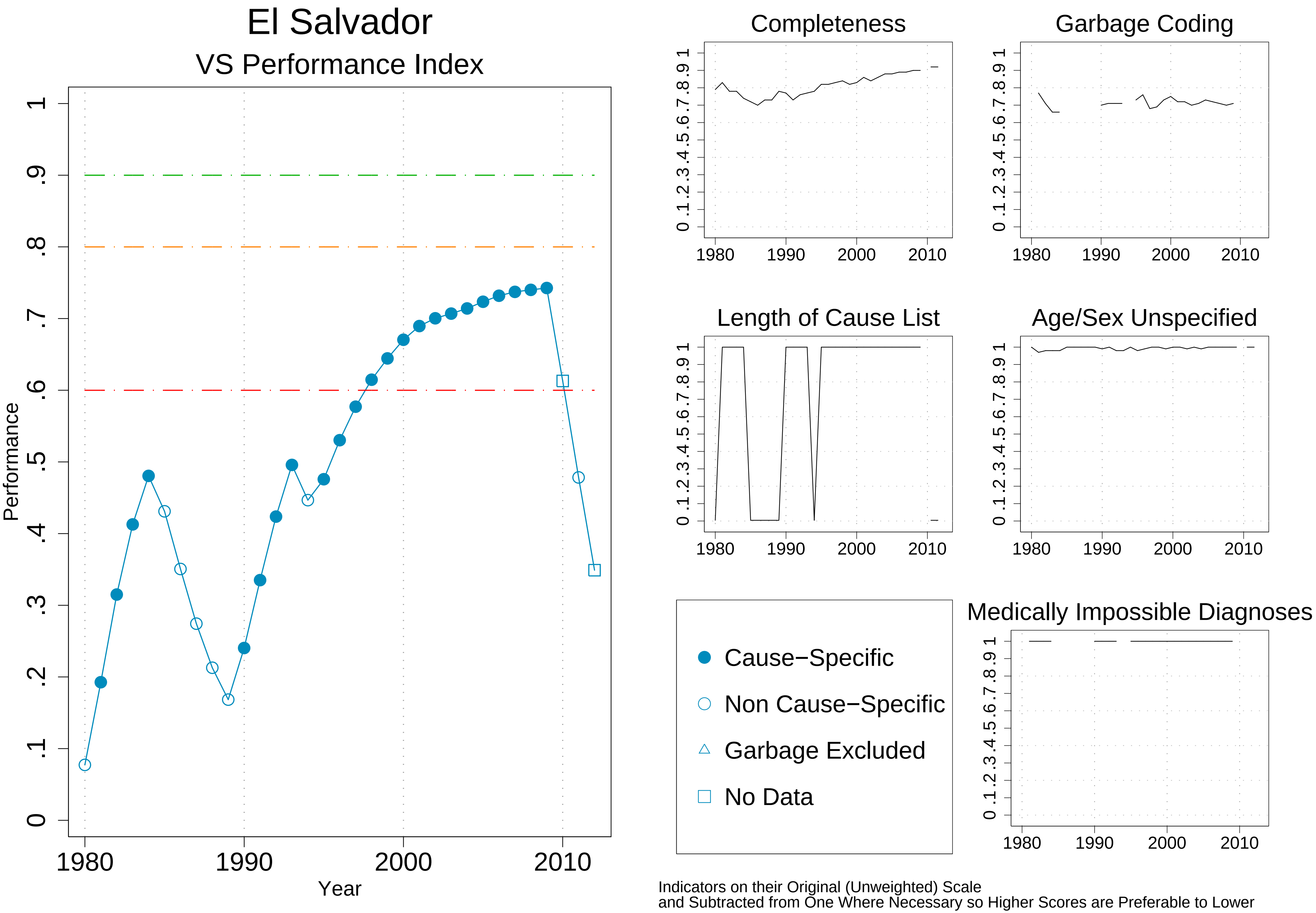

Indicators on their Original (Unweighted) Scale
and Subtracted from One Where Necessary so Higher Scores are Preferable to Lower

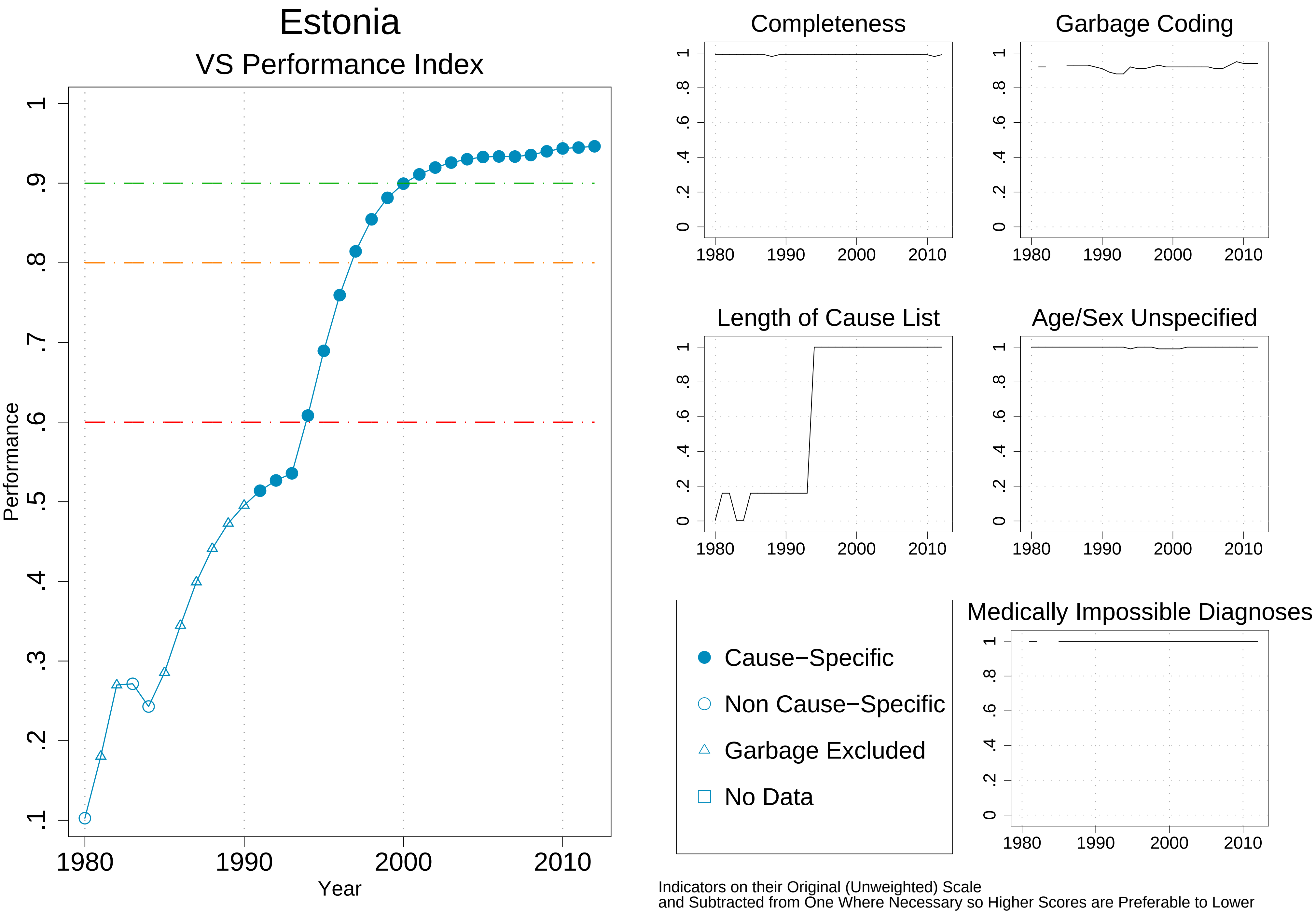

Indicators on their Original (Unweighted) Scale
and Subtracted from One Where Necessary so Higher Scores are Preferable to Lower

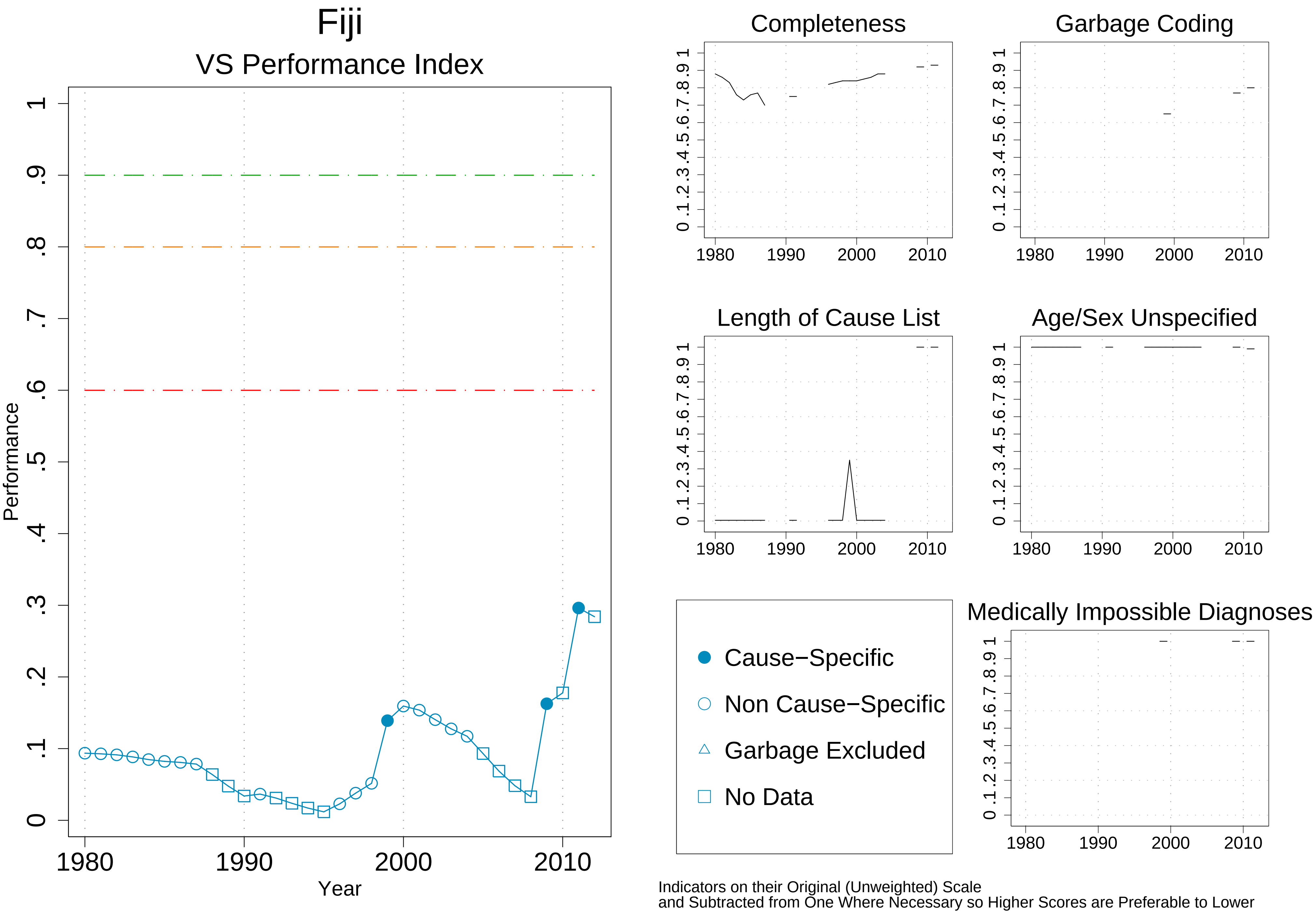

Indicators on their Original (Unweighted) Scale
and Subtracted from One Where Necessary so Higher Scores are Preferable to Lower

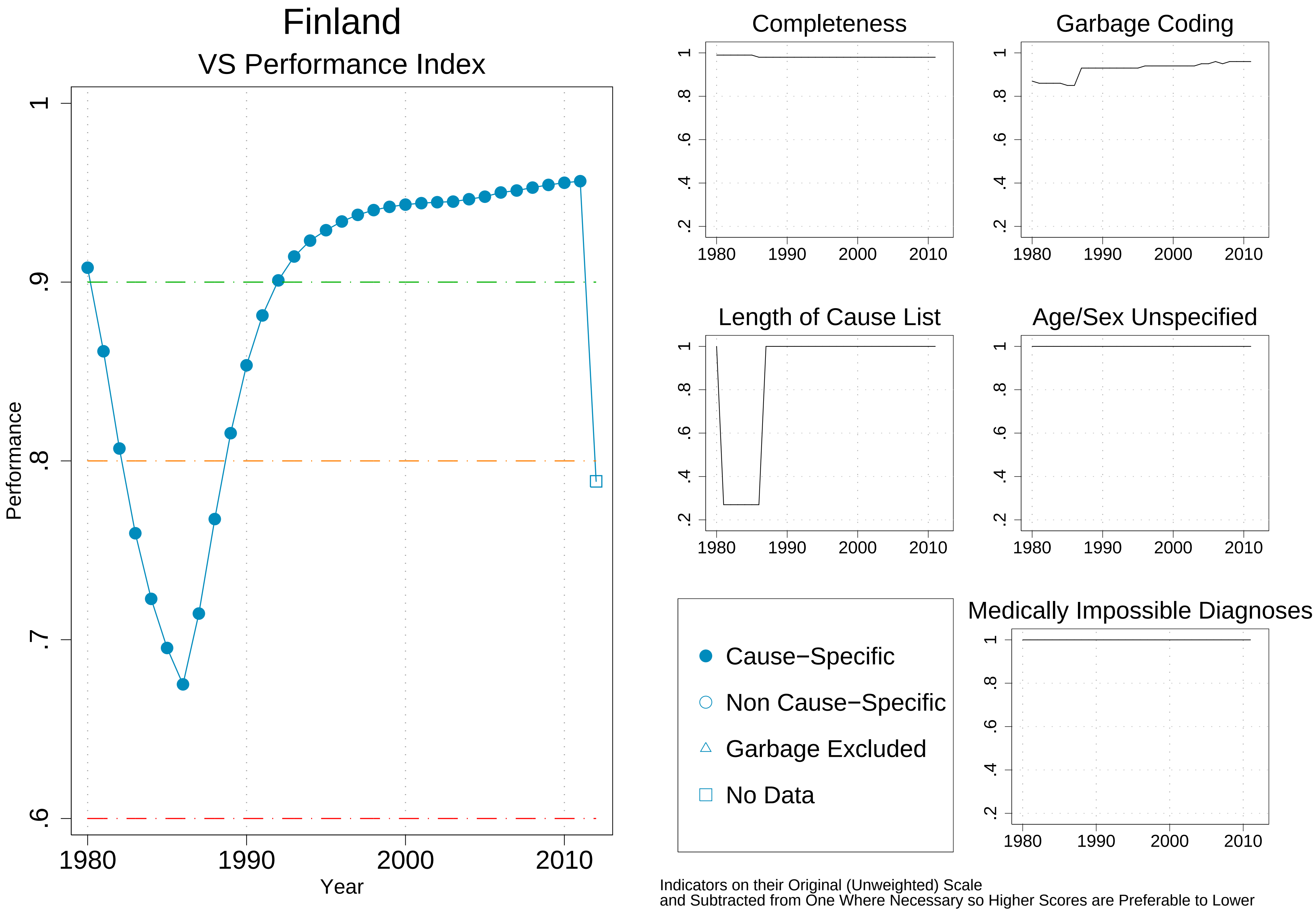

Finland
VS Performance Index
Performance
Year
1980
1990
2000
2010
.6
.7
.8
.9
1
Completeness
Garbage Coding
Length of Cause List
Age/Sex Unspecified
Medically Impossible Diagnoses
Cause−Specific
Non Cause−Specific
Garbage Excluded
No Data
Indicators on their Original (Unweighted) Scale
and Subtracted from One Where Necessary so Higher Scores are Preferable to Lower

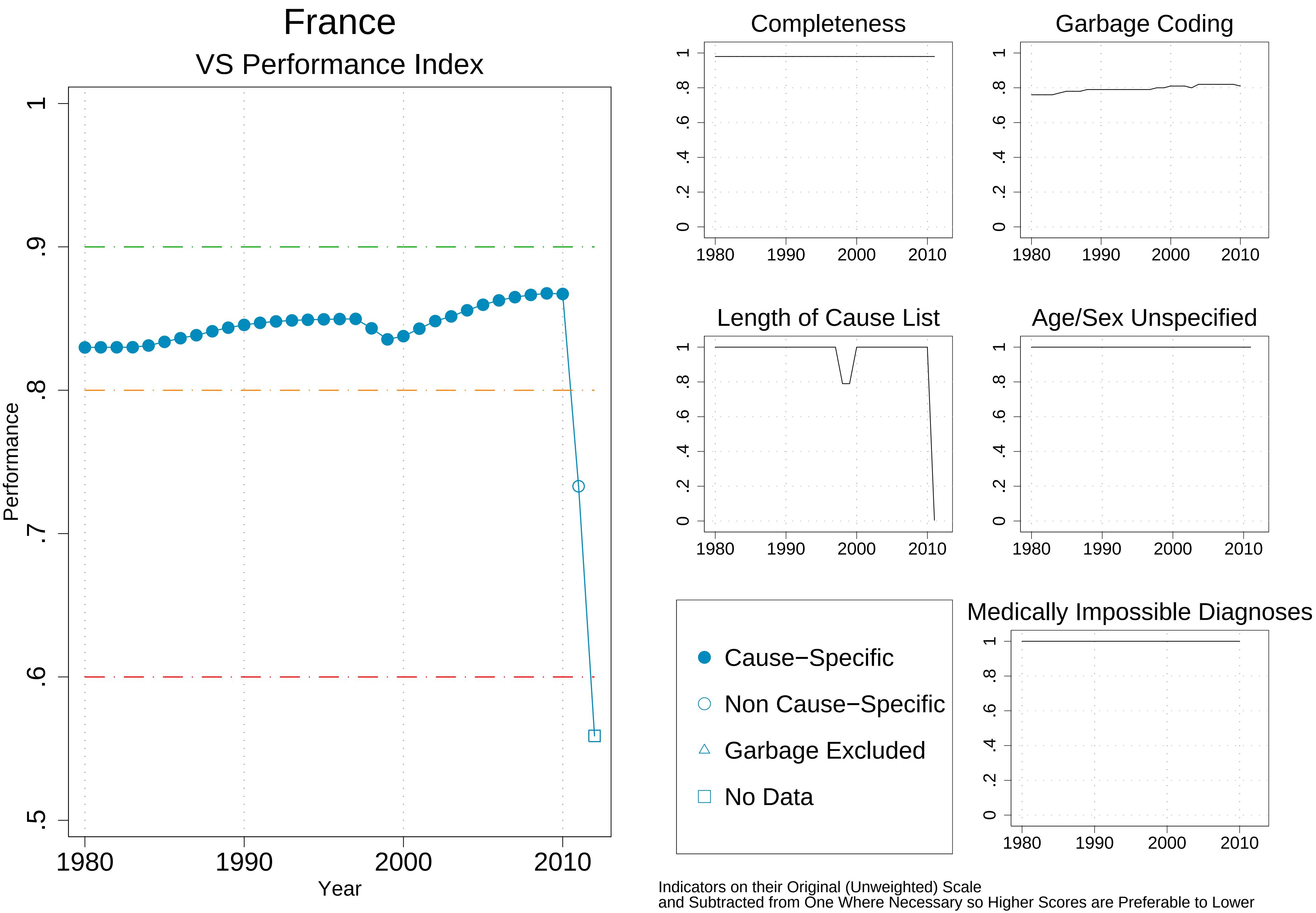

Indicators on their Original (Unweighted) Scale
and Subtracted from One Where Necessary so Higher Scores are Preferable to Lower

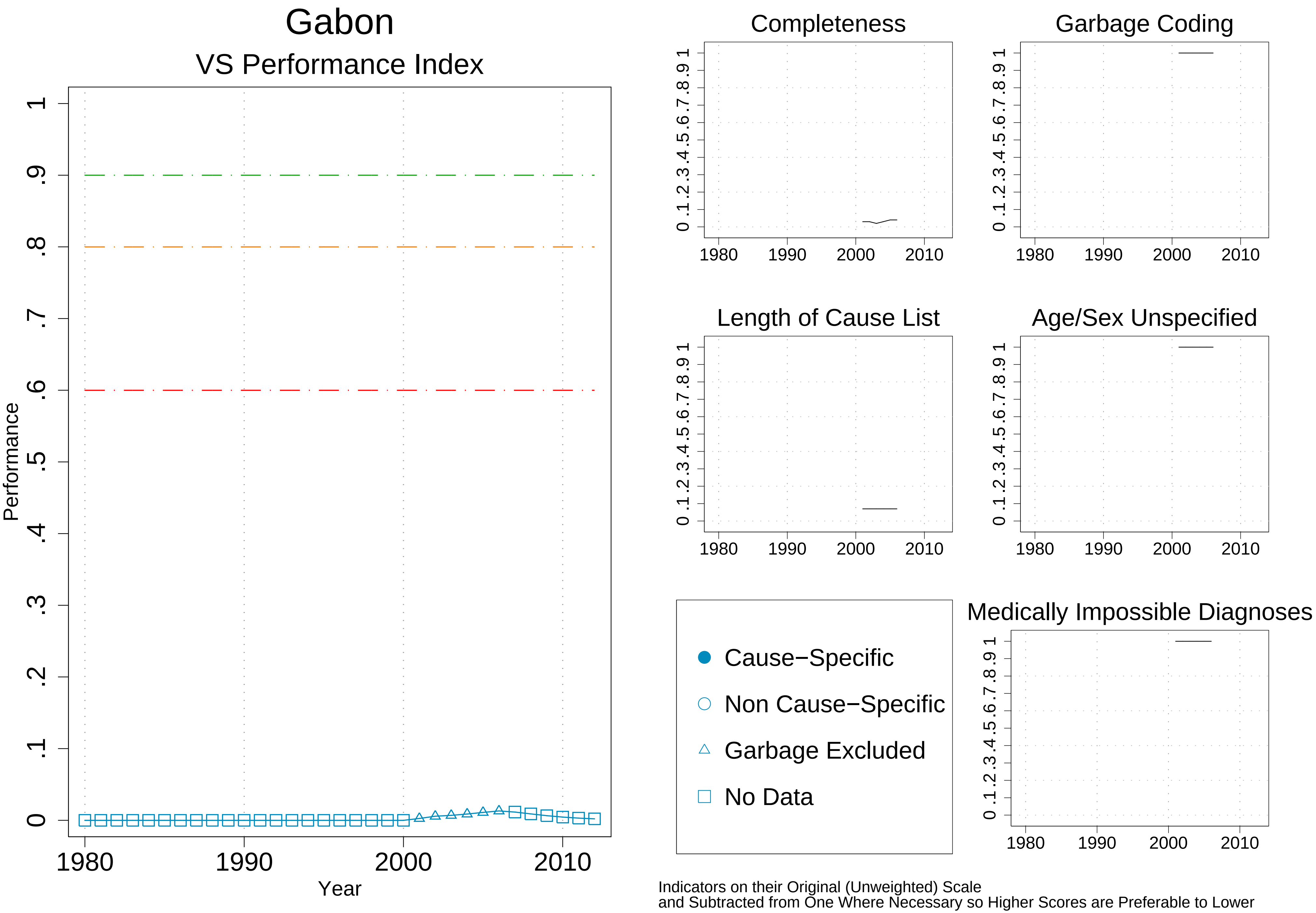

Indicators on their Original (Unweighted) Scale
and Subtracted from One Where Necessary so Higher Scores are Preferable to Lower

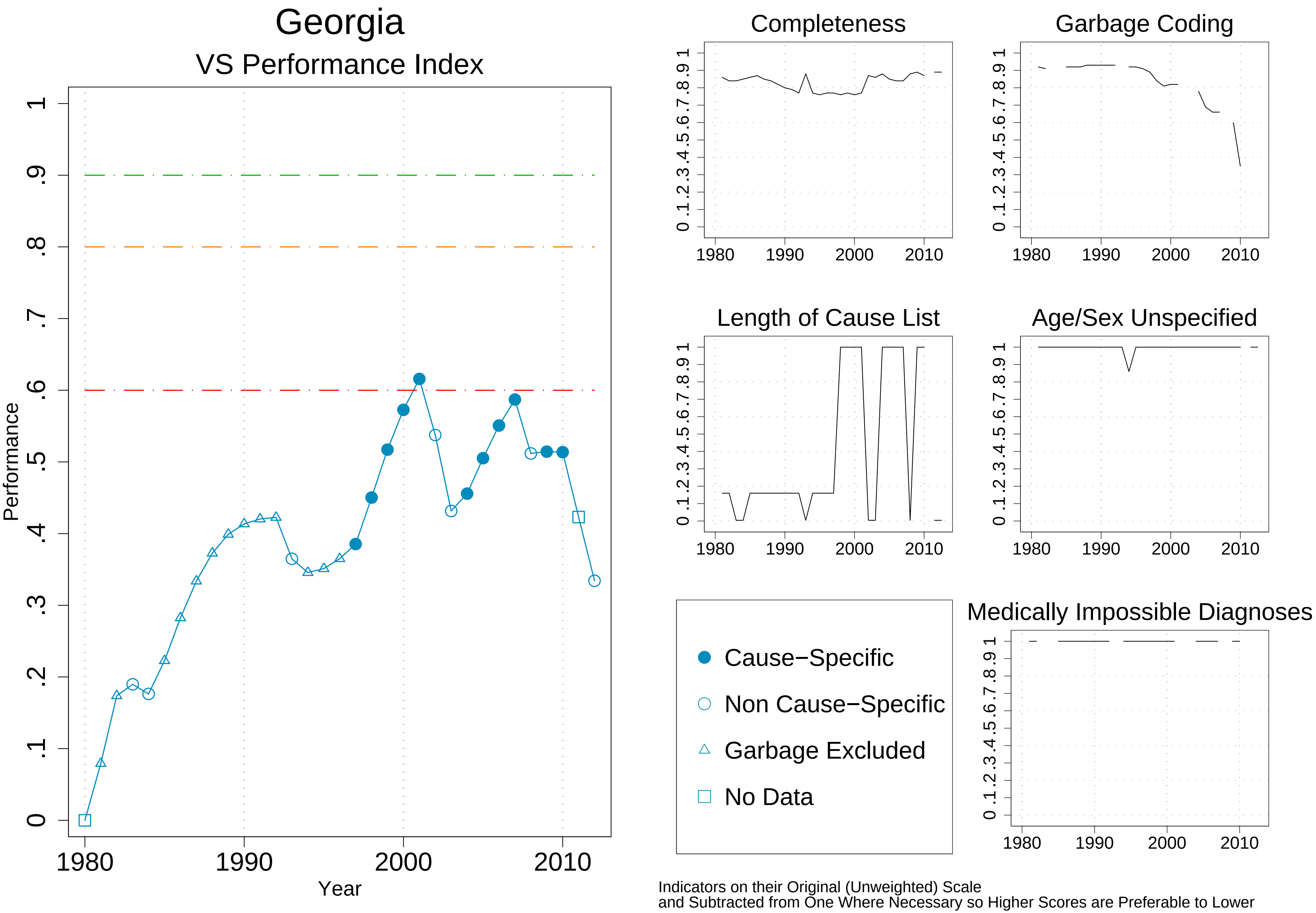

Indicators on their Original (Unweighted) Scale
and Subtracted from One Where Necessary so Higher Scores are Preferable to Lower

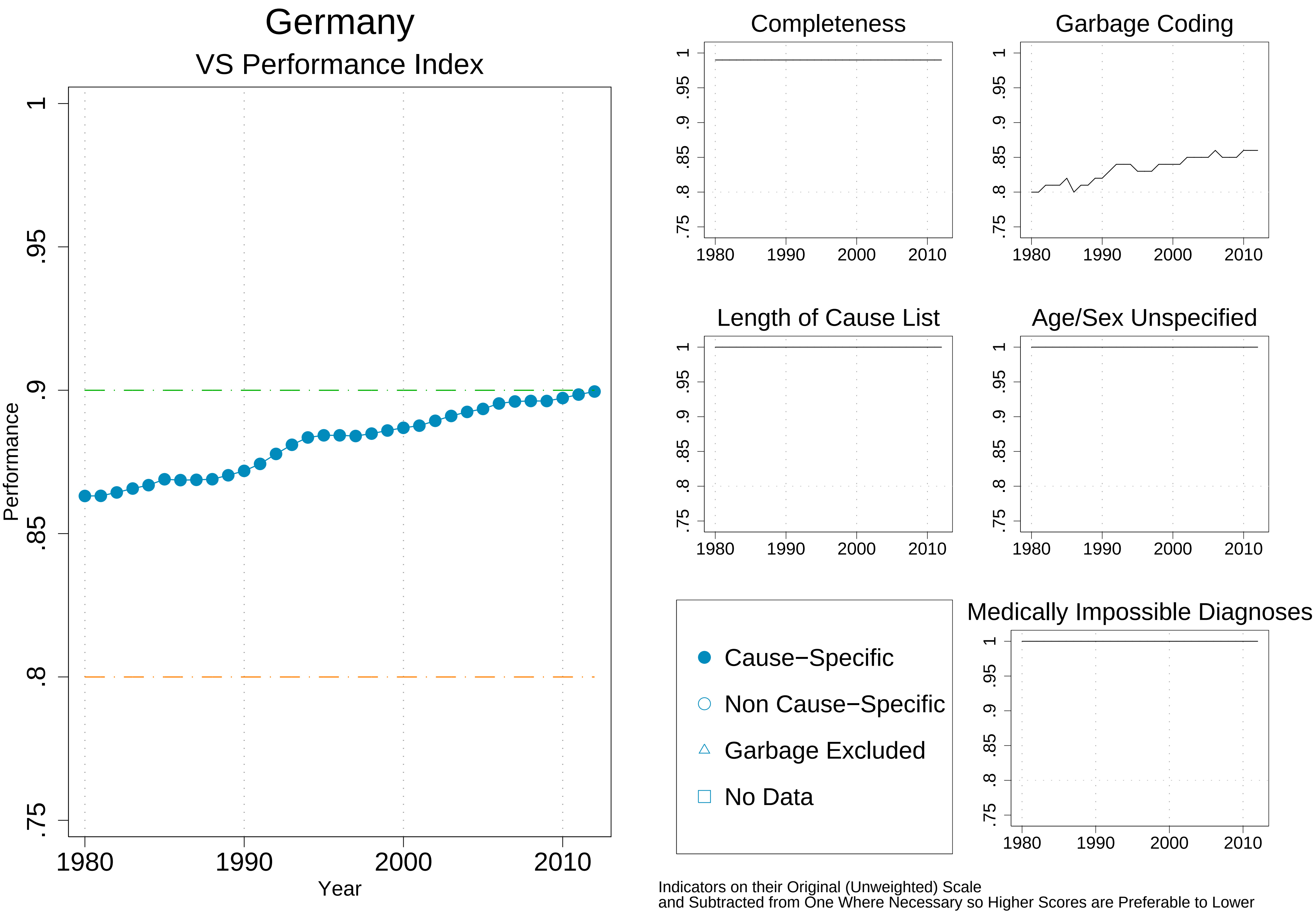

Indicators on their Original (Unweighted) Scale
and Subtracted from One Where Necessary so Higher Scores are Preferable to Lower

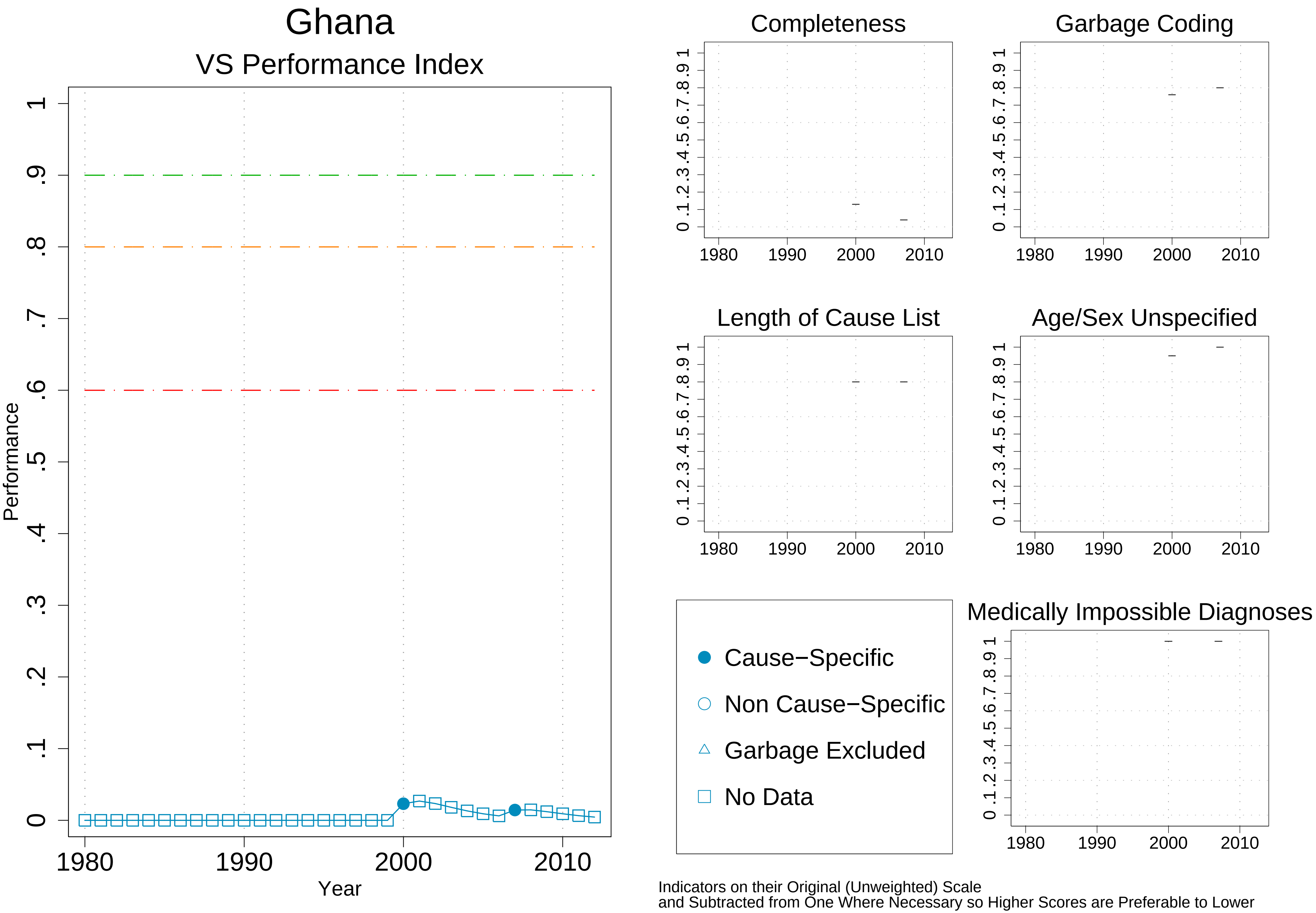
Ghana
VS Performance Index
Performance
Year
Completeness
Garbage Coding
Length of Cause List
Age/Sex Unspecified
Medically Impossible Diagnoses
Cause−Specific
Non Cause−Specific
Garbage Excluded
No Data
Indicators on their Original (Unweighted) Scale
and Subtracted from One Where Necessary so Higher Scores are Preferable to Lower

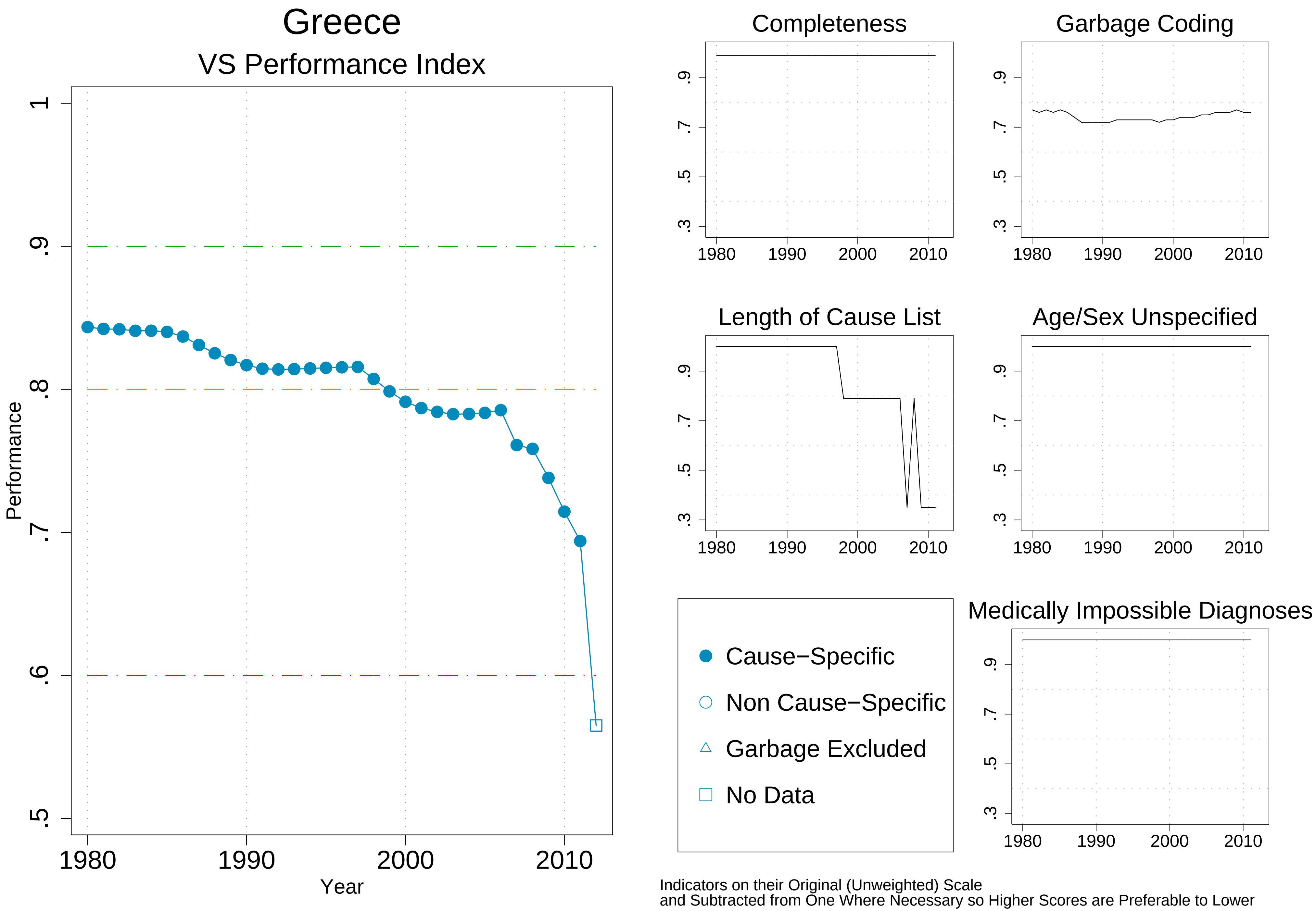

Greece
VS Performance Index
Performance
Year
Completeness
Garbage Coding
Length of Cause List
Age/Sex Unspecified
Medically Impossible Diagnoses
Cause−Specific
Non Cause−Specific
Garbage Excluded
No Data
Indicators on their Original (Unweighted) Scale
and Subtracted from One Where Necessary so Higher Scores are Preferable to Lower

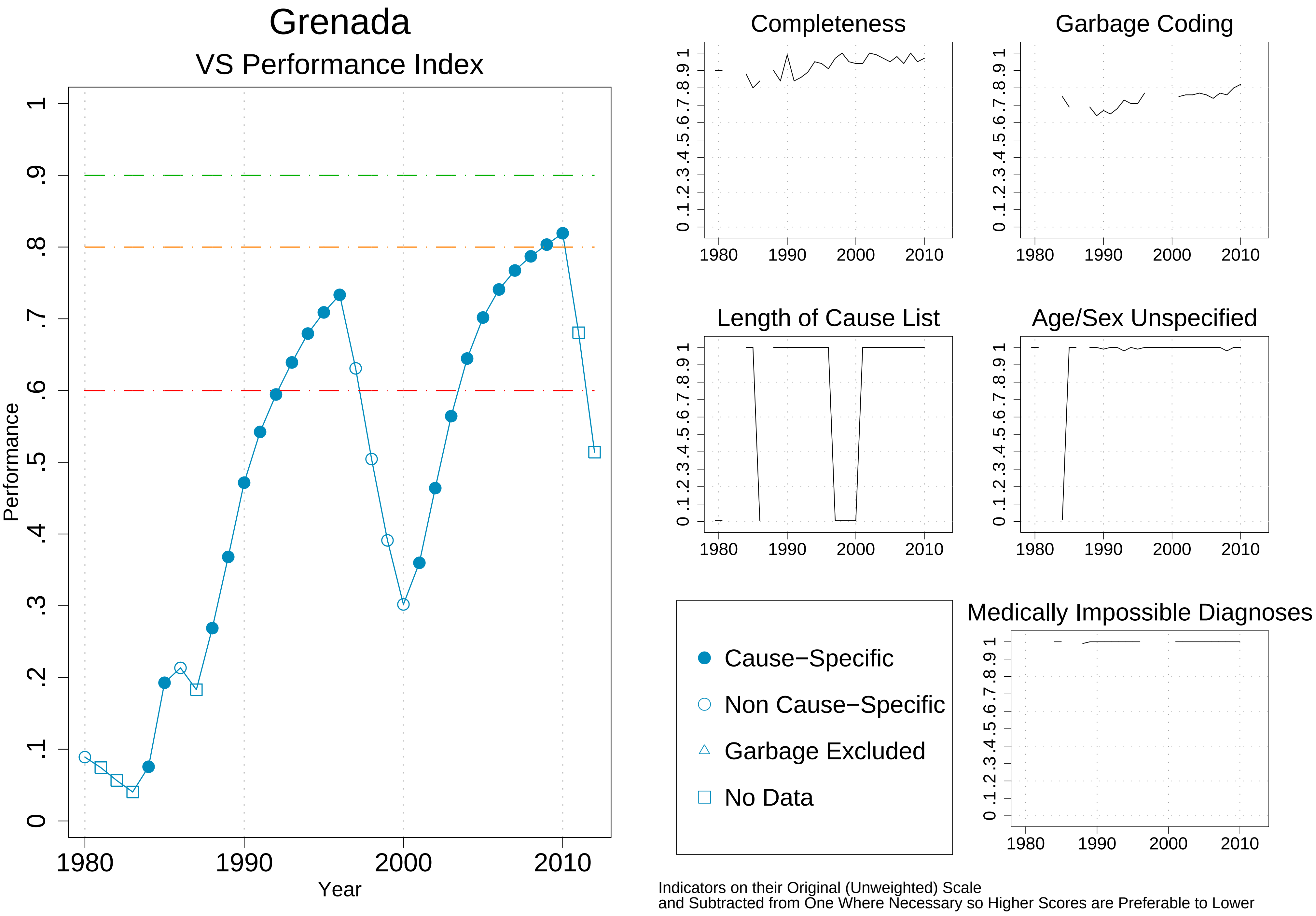
Grenada
VS Performance Index
Performance
Year
Completeness
Garbage Coding
Length of Cause List
Age/Sex Unspecified
Medically Impossible Diagnoses
Cause−Specific
Non Cause−Specific
Garbage Excluded
No Data
Indicators on their Original (Unweighted) Scale
and Subtracted from One Where Necessary so Higher Scores are Preferable to Lower

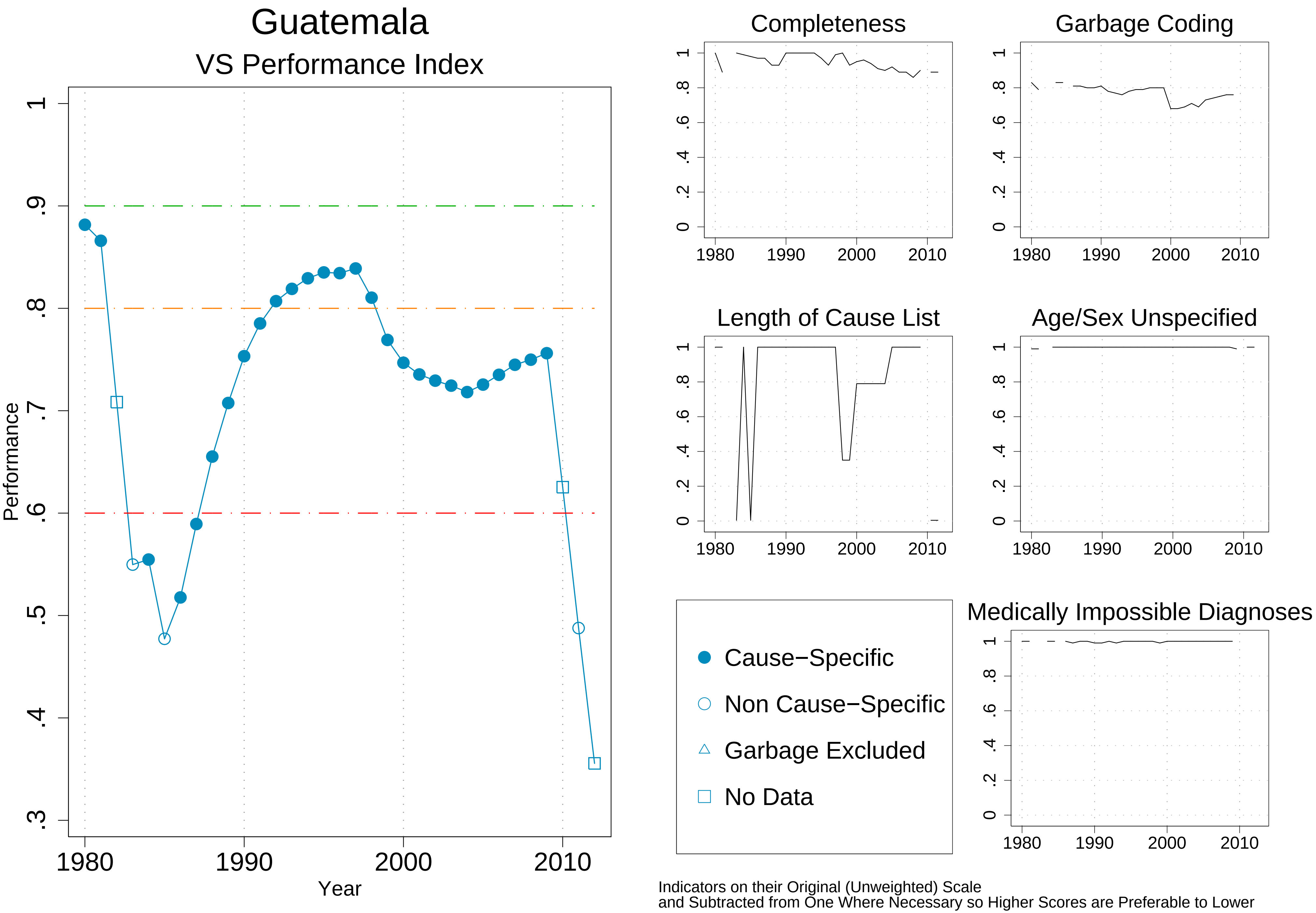
Guatemala
VS Performance Index
Performance
Year
Completeness
Garbage Coding
Length of Cause List
Age/Sex Unspecified
Medically Impossible Diagnoses
Cause−Specific
Non Cause−Specific
Garbage Excluded
No Data
Indicators on their Original (Unweighted) Scale
and Subtracted from One Where Necessary so Higher Scores are Preferable to Lower

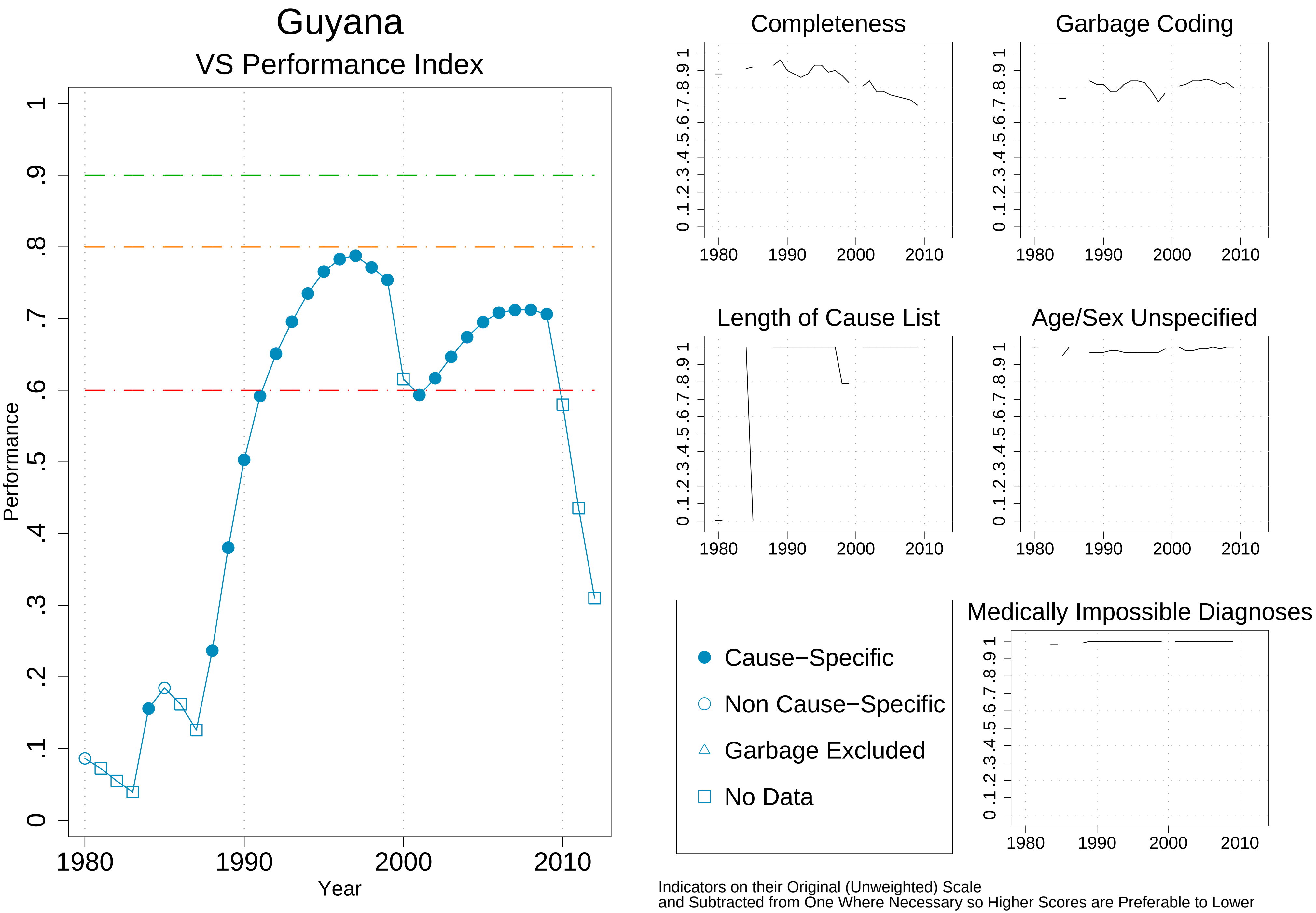

Indicators on their Original (Unweighted) Scale
and Subtracted from One Where Necessary so Higher Scores are Preferable to Lower

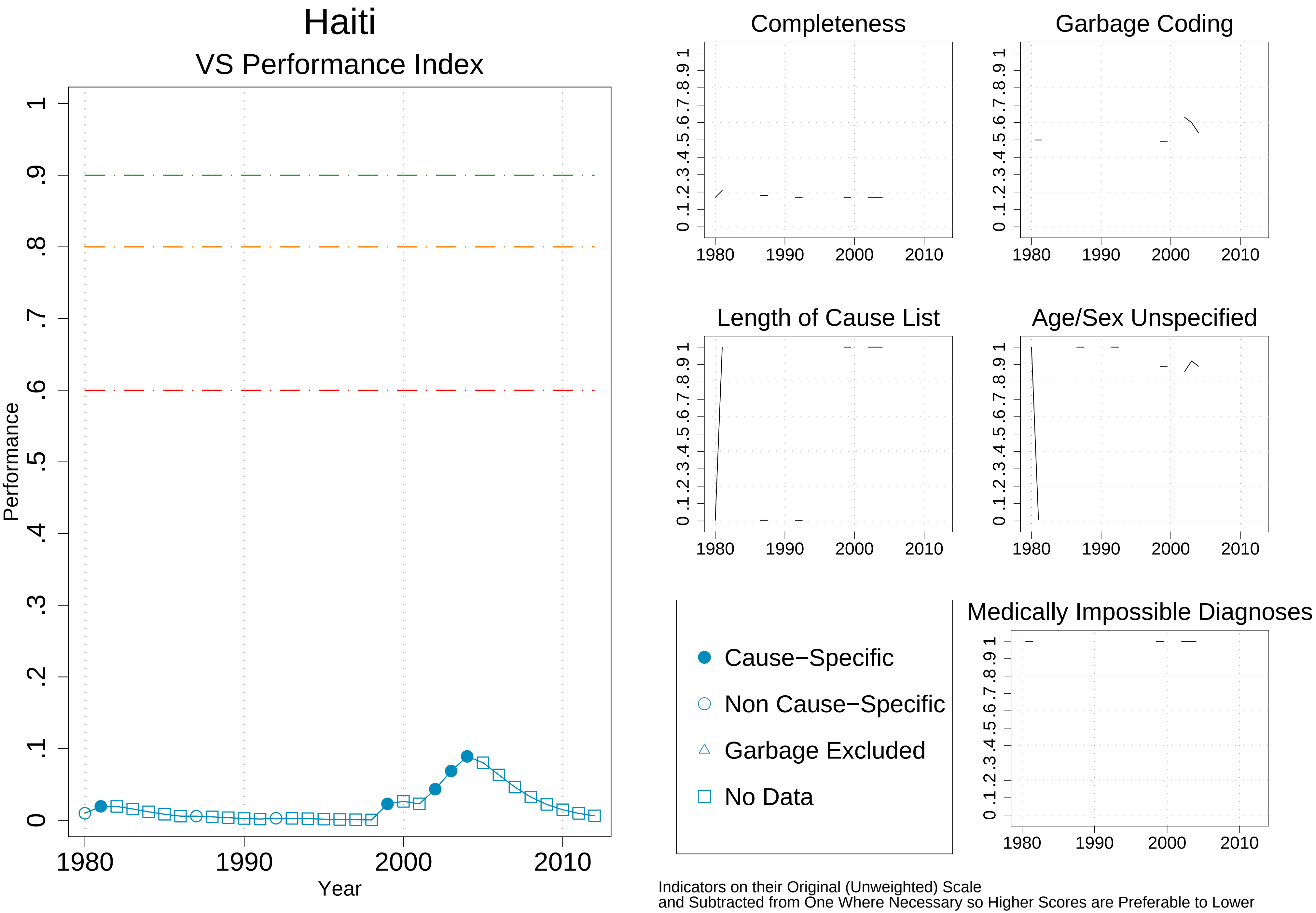

Haiti
VS Performance Index
Performance
Year
0
.1
.2
.3
.4
.5
.6
.7
.8
.9
1
1980
1990
2000
2010
Completeness
Garbage Coding
Length of Cause List
Age/Sex Unspecified
Medically Impossible Diagnoses
0 .1 .2 .3 .4 .5 .6 .7 .8 .9 1
Cause−Specific
Non Cause−Specific
Garbage Excluded
No Data
Indicators on their Original (Unweighted) Scale
and Subtracted from One Where Necessary so Higher Scores are Preferable to Lower

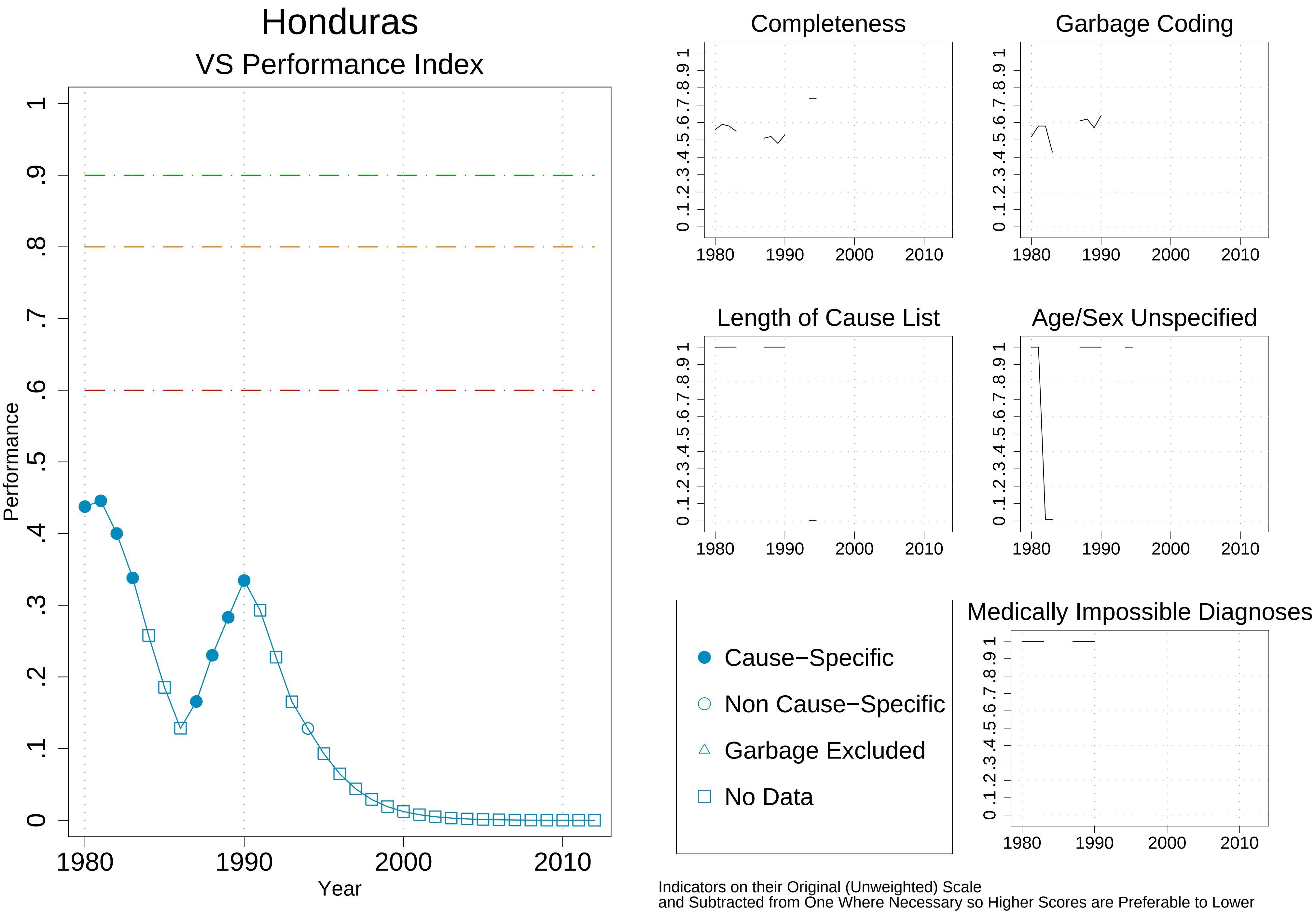

Indicators on their Original (Unweighted) Scale
and Subtracted from One Where Necessary so Higher Scores are Preferable to Lower

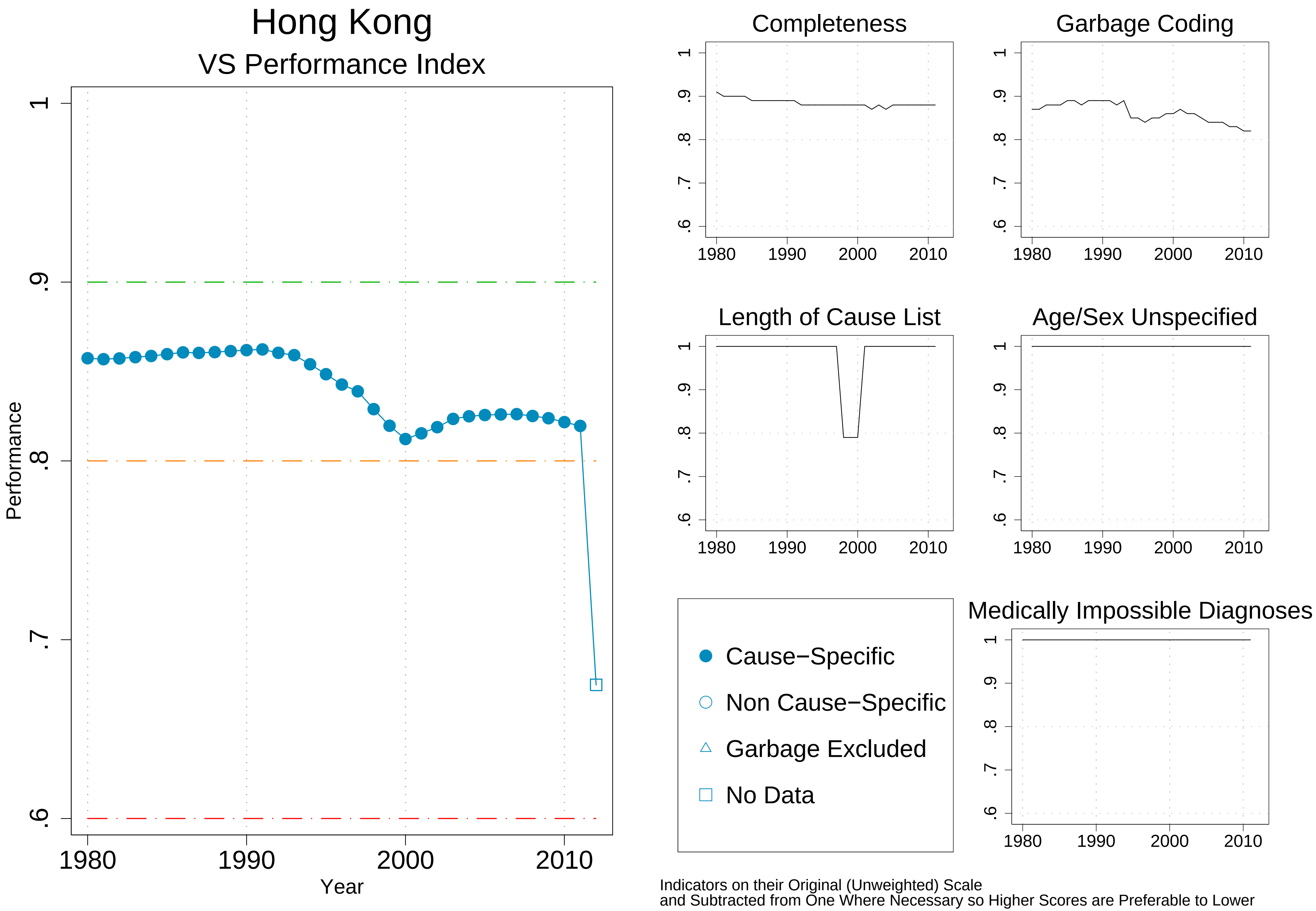

Indicators on their Original (Unweighted) Scale
and Subtracted from One Where Necessary so Higher Scores are Preferable to Lower

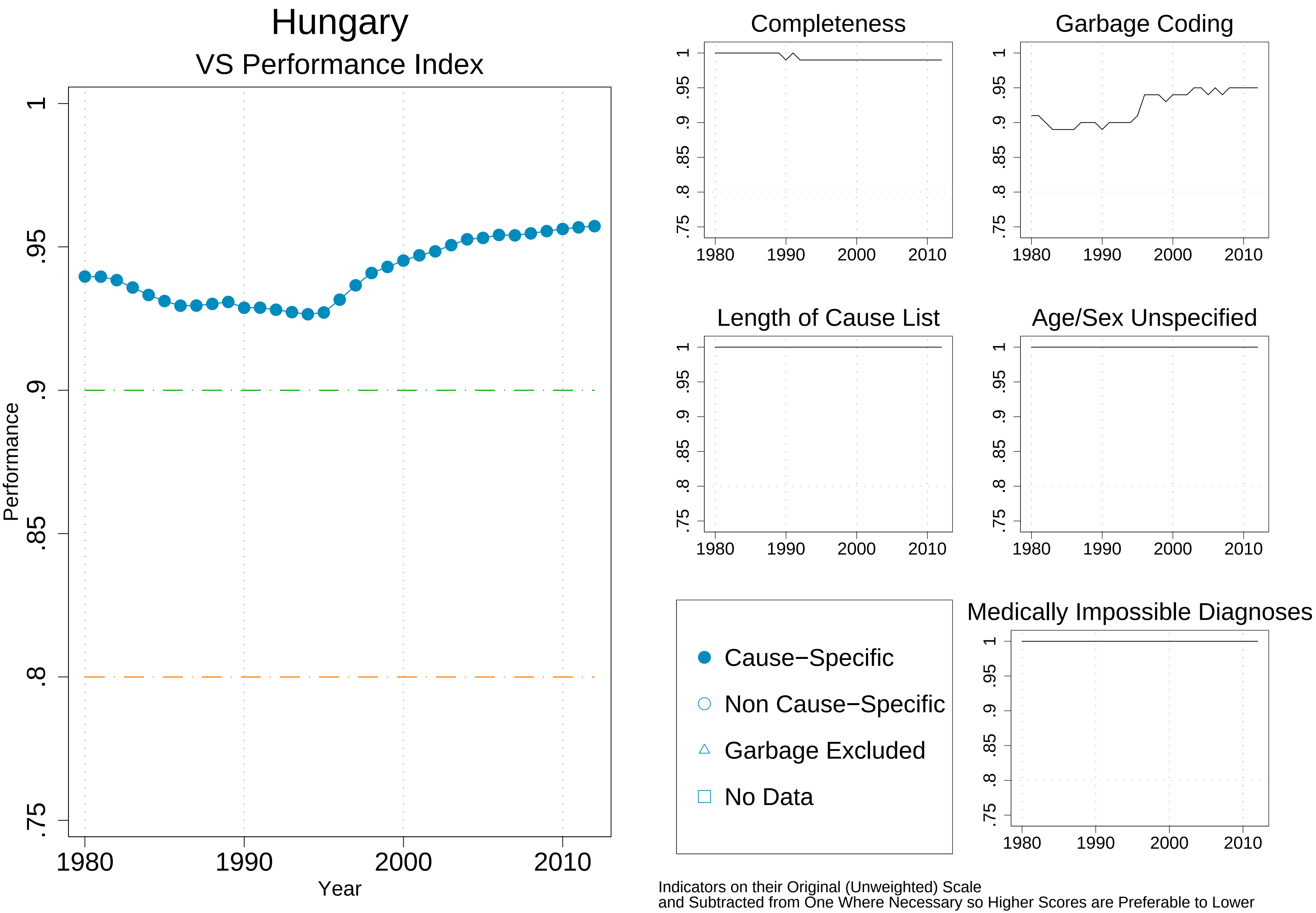

Hungary
VS Performance Index
Performance
Year
1980
1990
2000
2010
.75
.8
.85
.9
.95
1
Completeness
Garbage Coding
Length of Cause List
Age/Sex Unspecified
Medically Impossible Diagnoses
Cause−Specific
Non Cause−Specific
Garbage Excluded
No Data
Indicators on their Original (Unweighted) Scale
and Subtracted from One Where Necessary so Higher Scores are Preferable to Lower

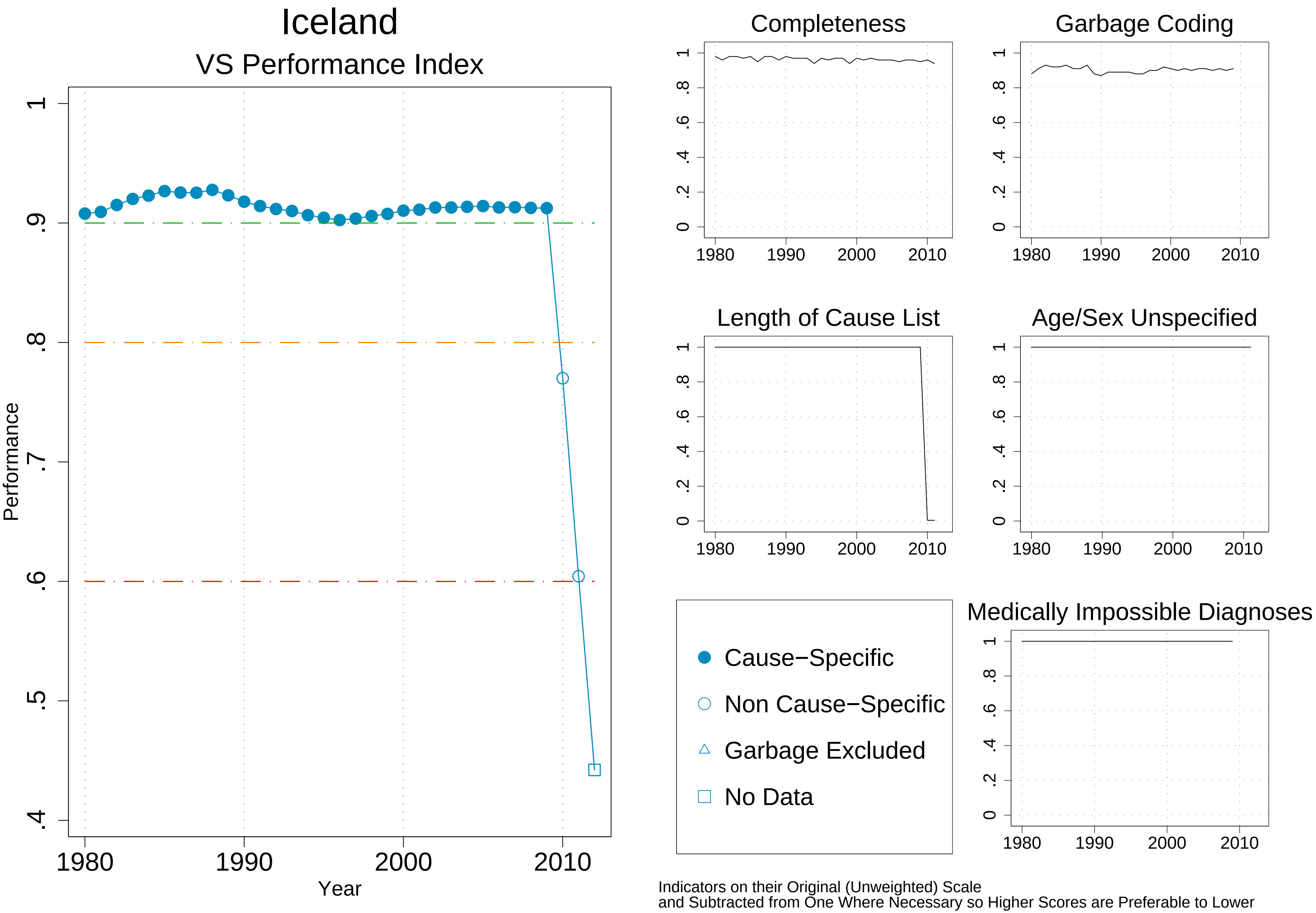

Indicators on their Original (Unweighted) Scale
and Subtracted from One Where Necessary so Higher Scores are Preferable to Lower

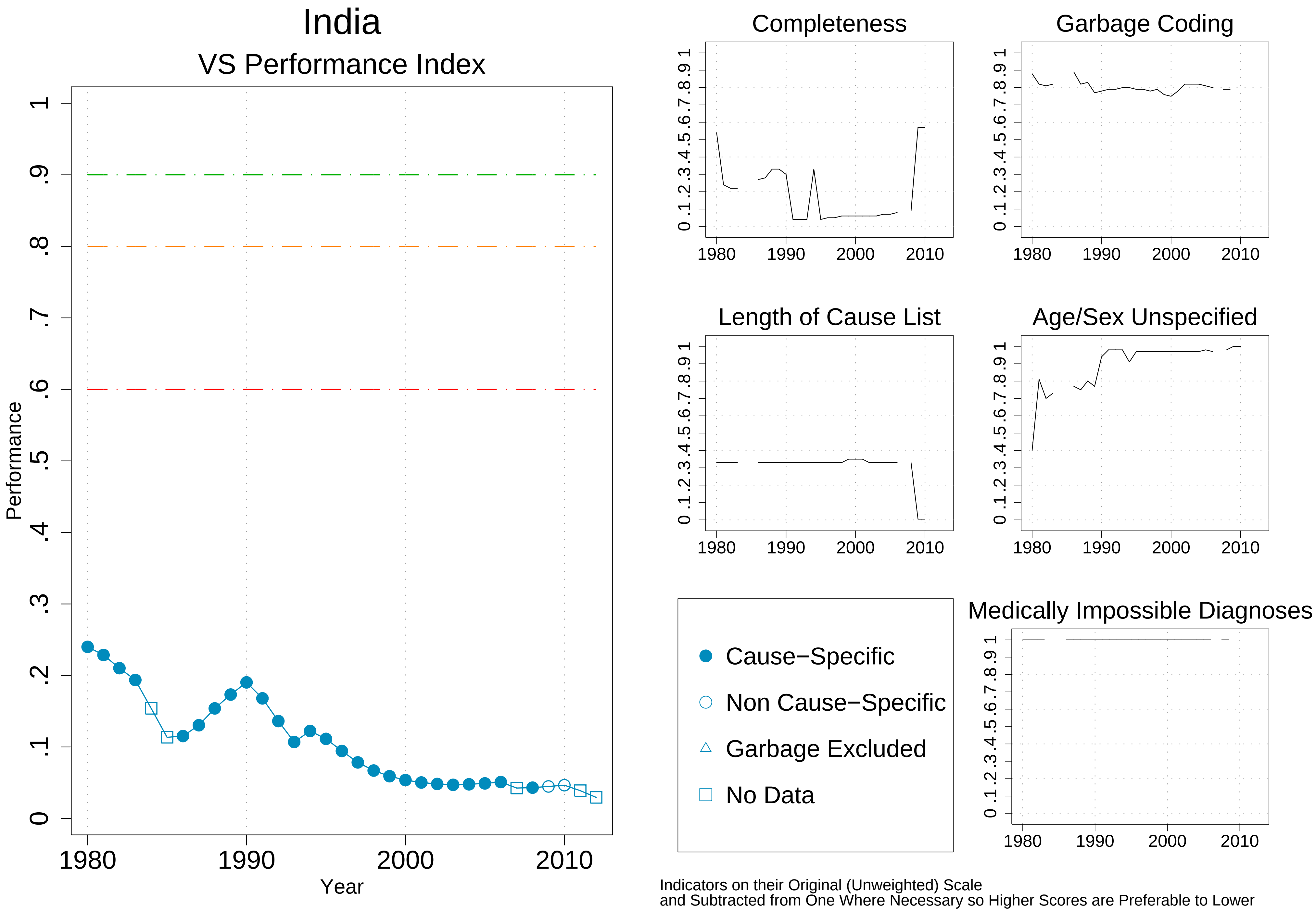

Indicators on their Original (Unweighted) Scale
and Subtracted from One Where Necessary so Higher Scores are Preferable to Lower

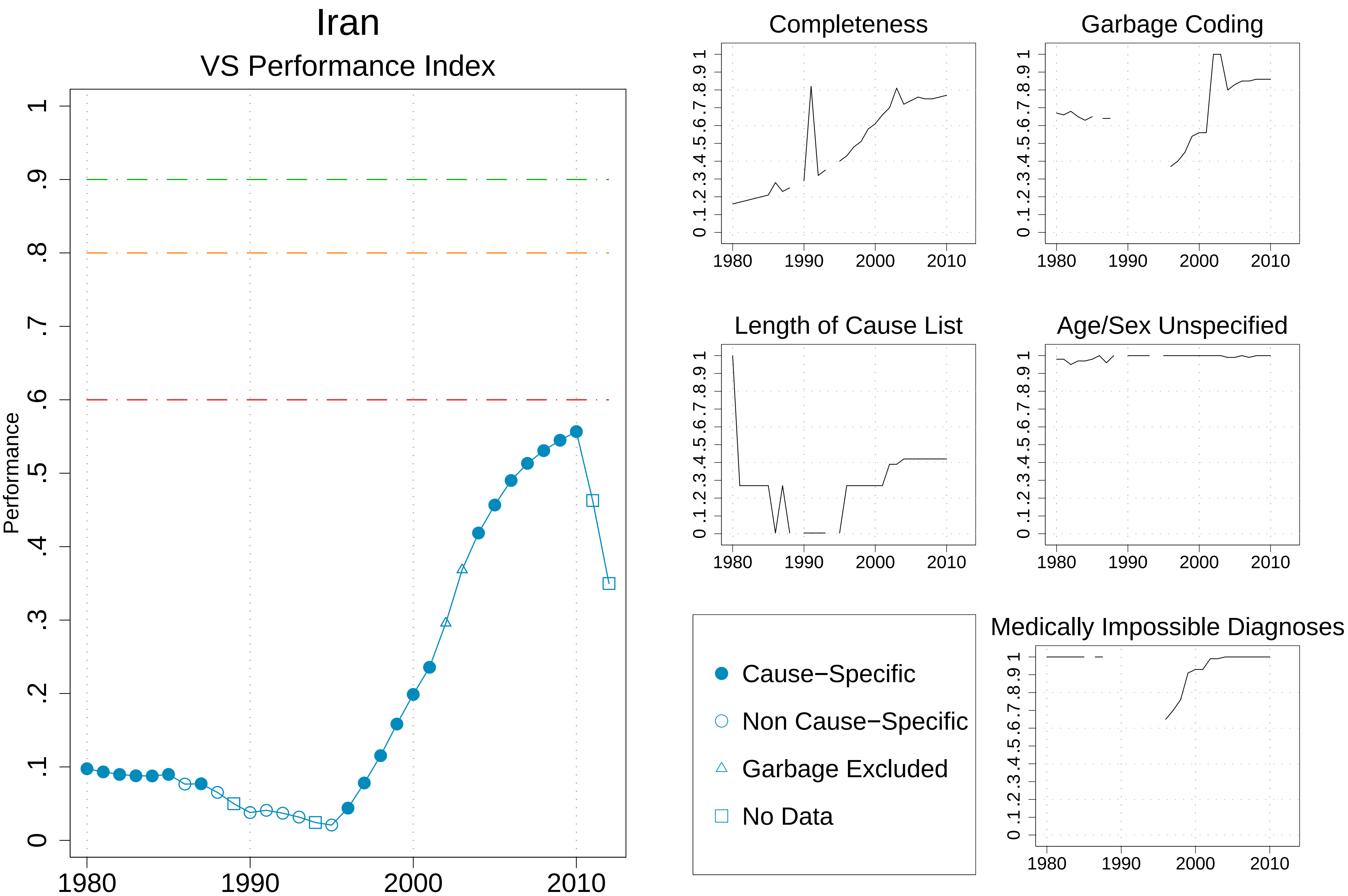

Indicators on their Original (Unweighted) Scale
and Subtracted from One Where Necessary so Higher Scores are Preferable to Lower

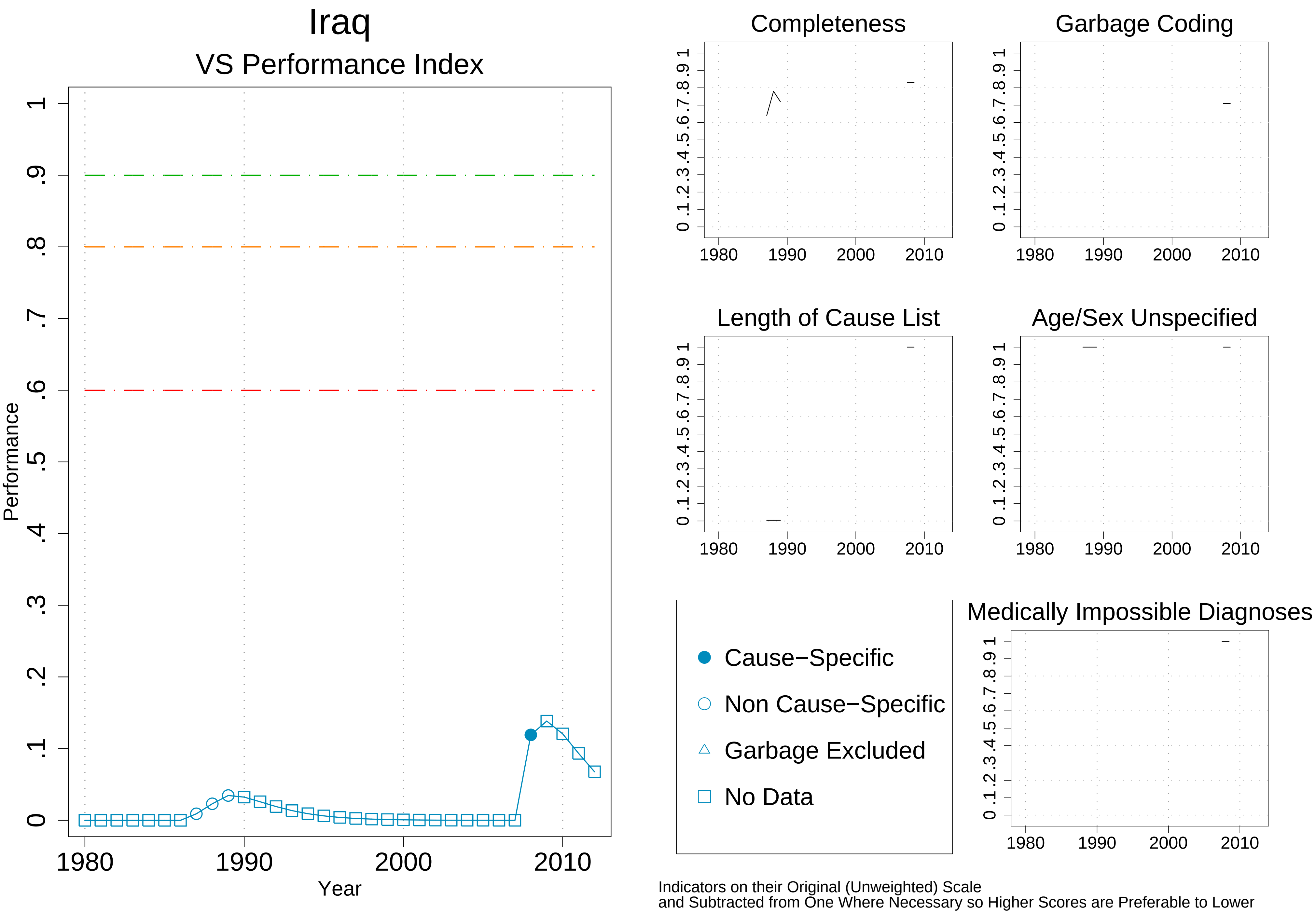

Indicators on their Original (Unweighted) Scale
and Subtracted from One Where Necessary so Higher Scores are Preferable to Lower

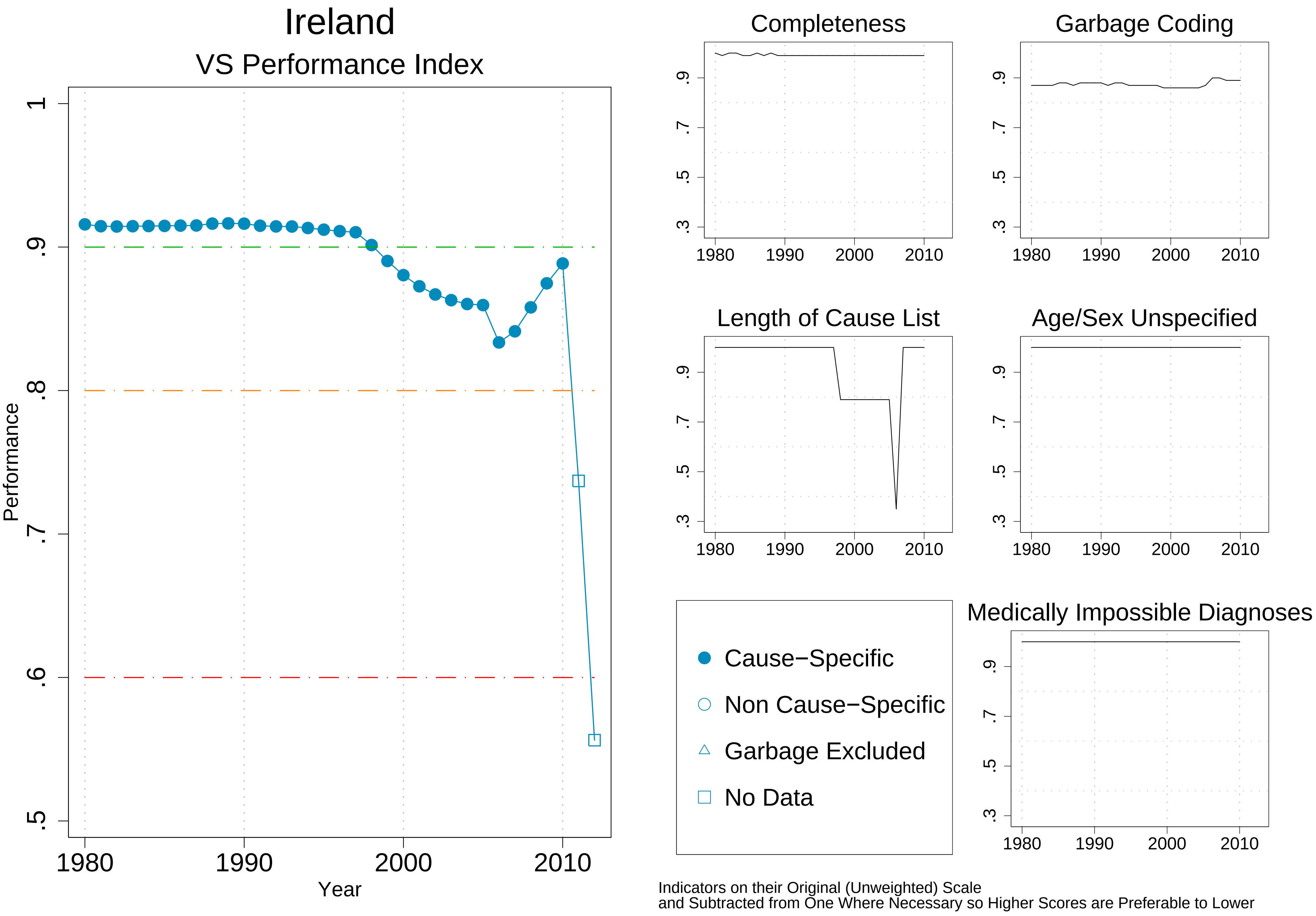

Ireland
VS Performance Index
Performance
Year
Completeness
Garbage Coding
Length of Cause List
Age/Sex Unspecified
Medically Impossible Diagnoses
Cause−Specific
Non Cause−Specific
Garbage Excluded
No Data
Indicators on their Original (Unweighted) Scale
and Subtracted from One Where Necessary so Higher Scores are Preferable to Lower

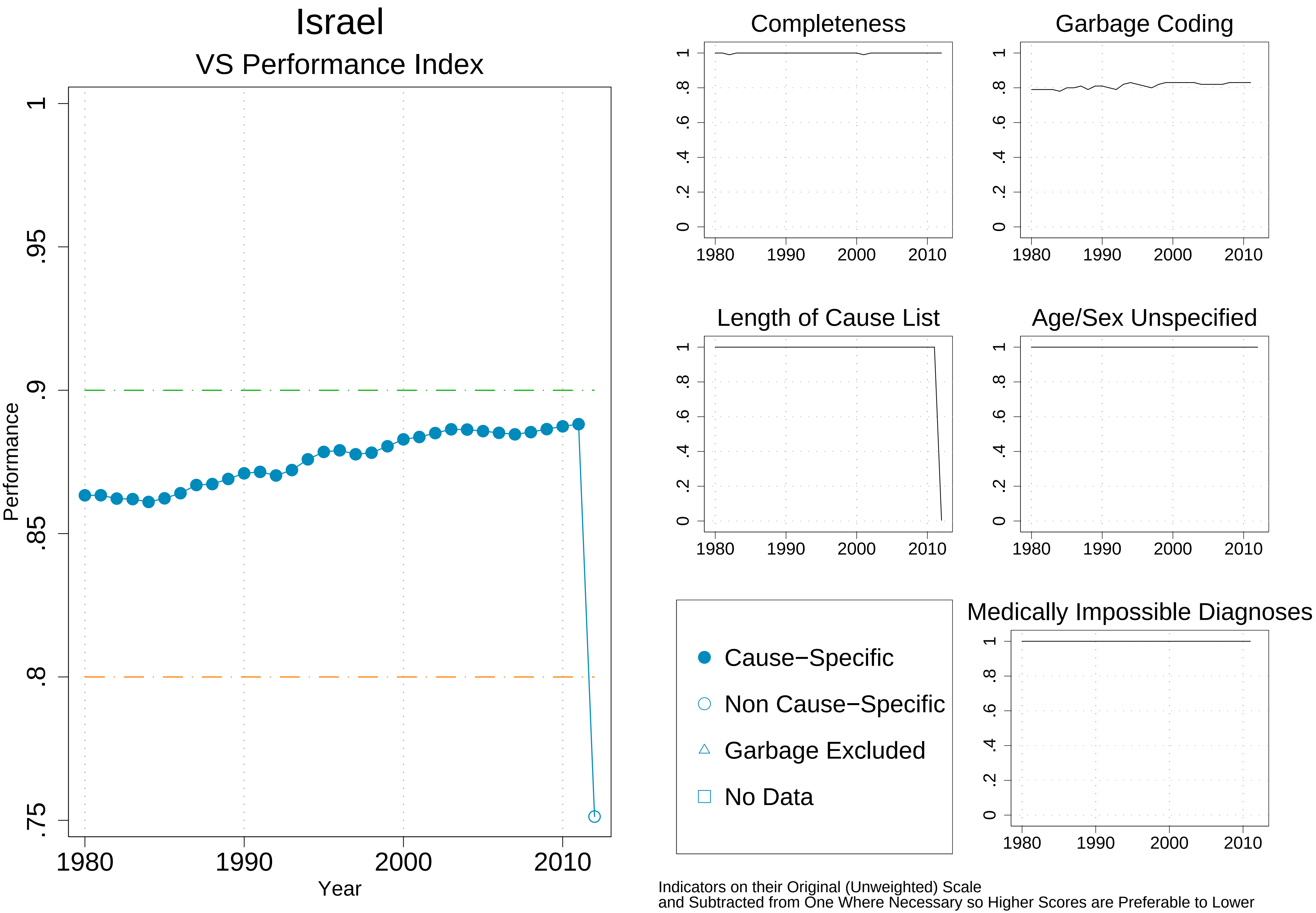

Indicators on their Original (Unweighted) Scale
and Subtracted from One Where Necessary so Higher Scores are Preferable to Lower

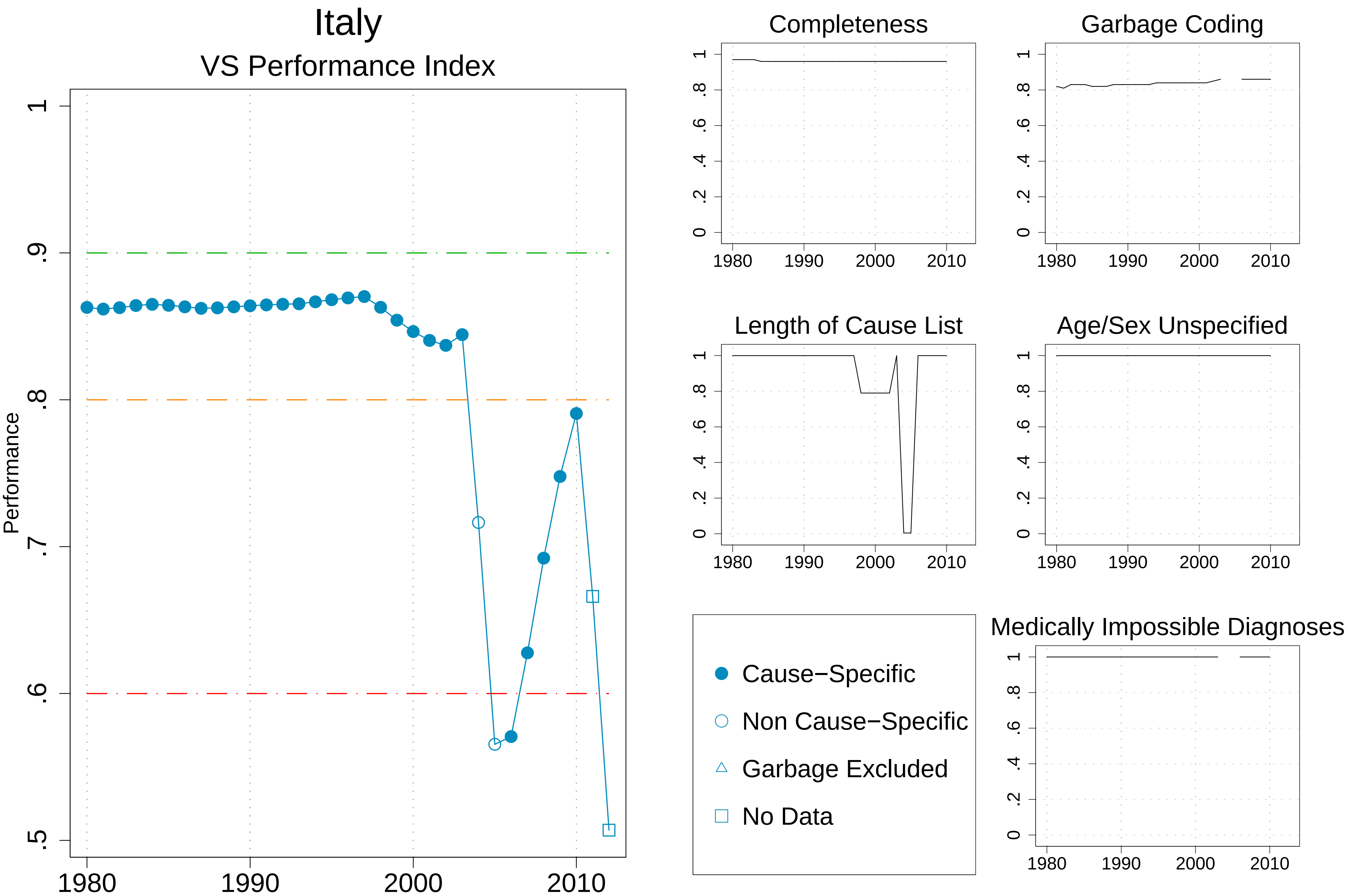

Indicators on their Original (Unweighted) Scale
and Subtracted from One Where Necessary so Higher Scores are Preferable to Lower

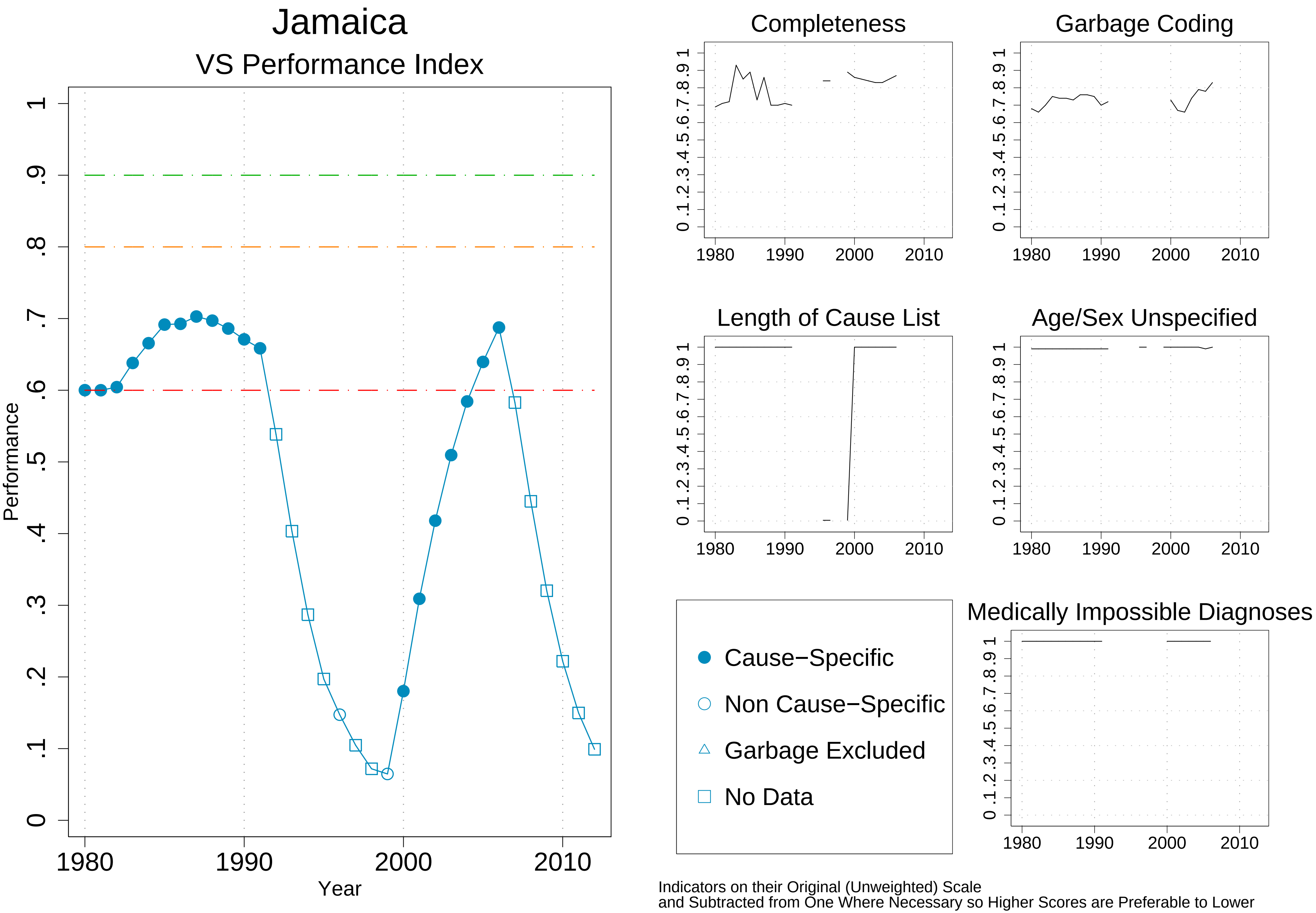

Indicators on their Original (Unweighted) Scale
and Subtracted from One Where Necessary so Higher Scores are Preferable to Lower

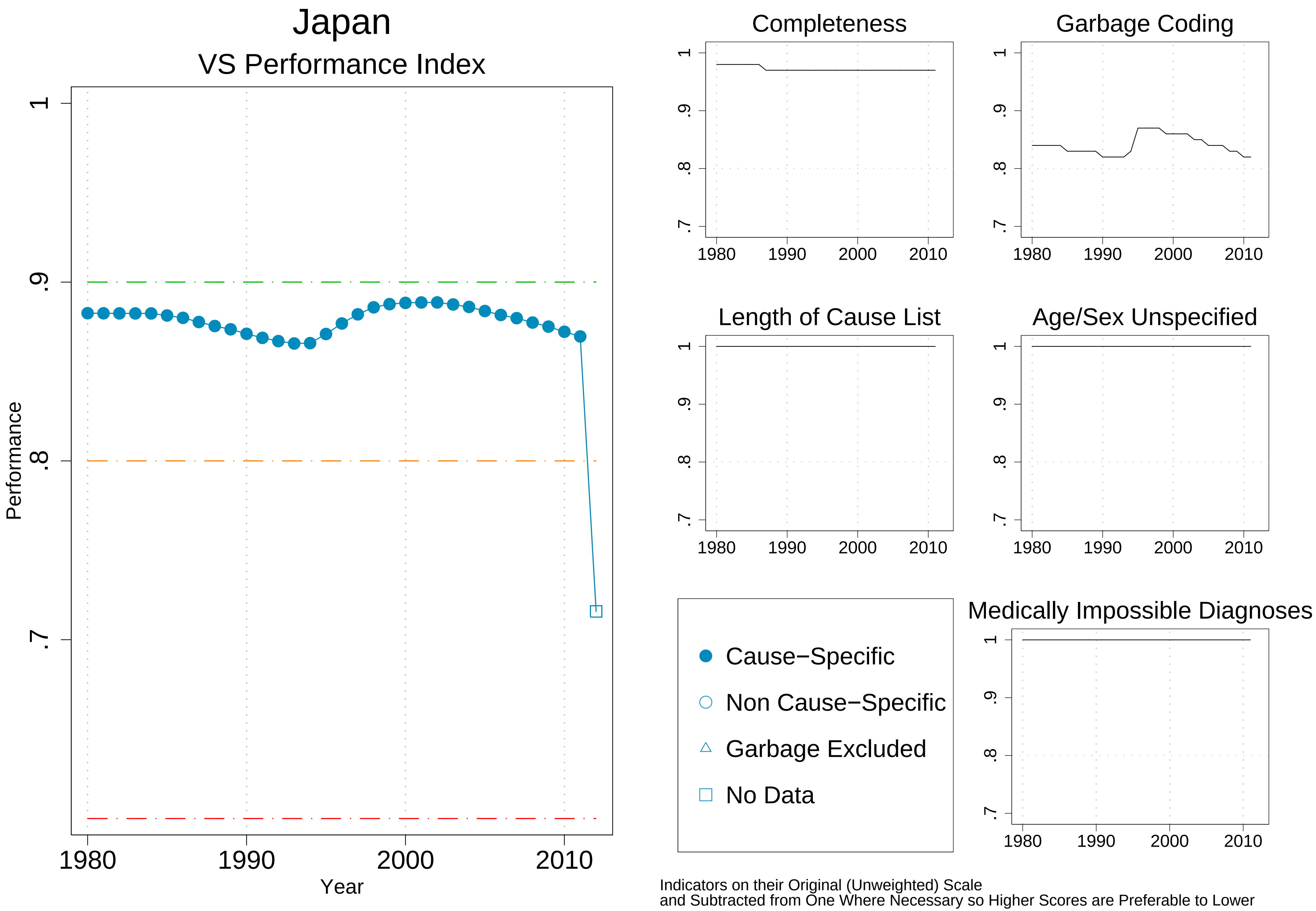
Japan
VS Performance Index
Performance
Year
Completeness
Garbage Coding
Length of Cause List
Age/Sex Unspecified
Medically Impossible Diagnoses
Cause−Specific
Non Cause−Specific
Garbage Excluded
No Data
Indicators on their Original (Unweighted) Scale
and Subtracted from One Where Necessary so Higher Scores are Preferable to Lower

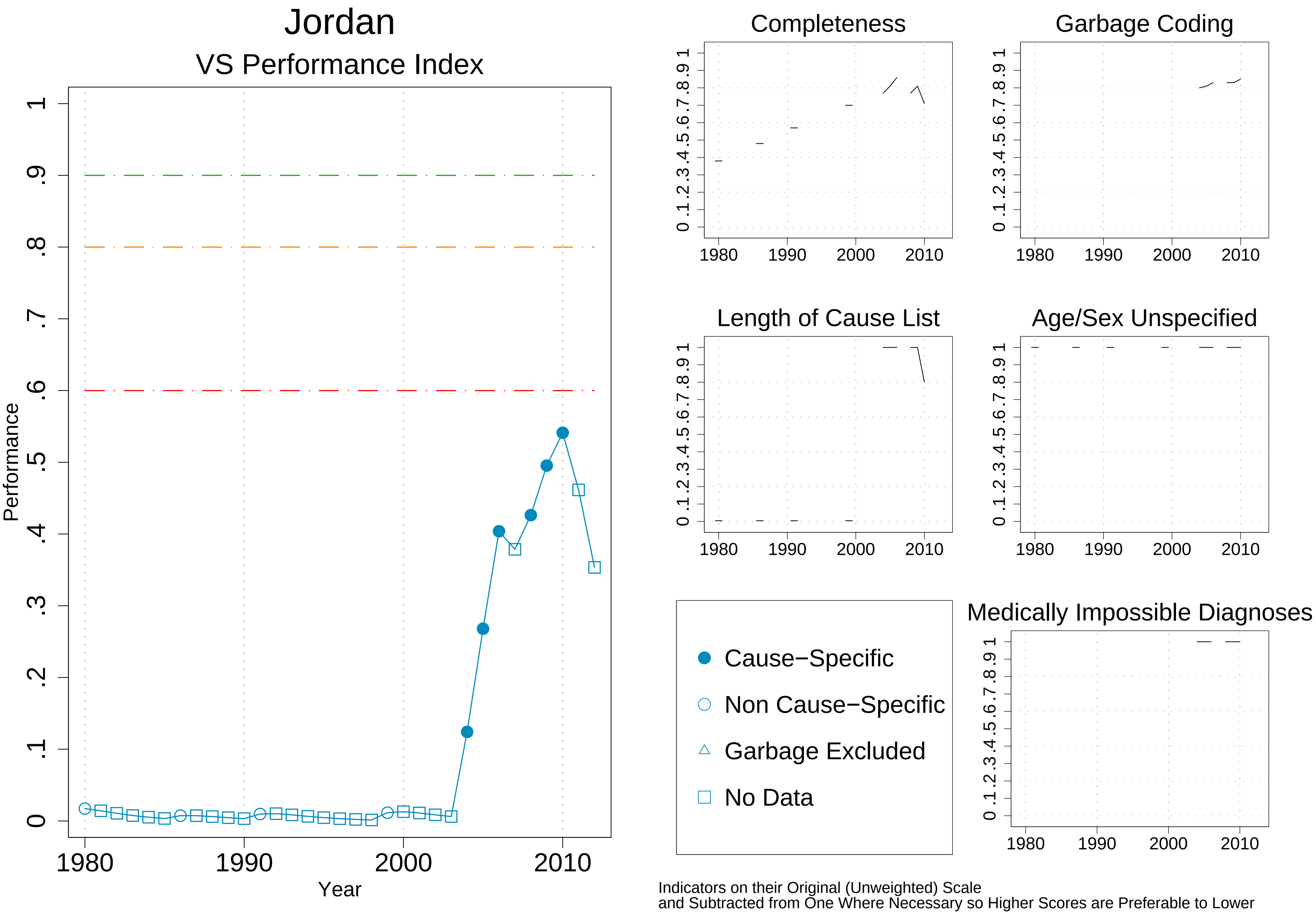

Jordan
VS Performance Index
Performance
Year
Completeness
Garbage Coding
Length of Cause List
Age/Sex Unspecified
Medically Impossible Diagnoses
Cause−Specific
Non Cause−Specific
Garbage Excluded
No Data
Indicators on their Original (Unweighted) Scale
and Subtracted from One Where Necessary so Higher Scores are Preferable to Lower

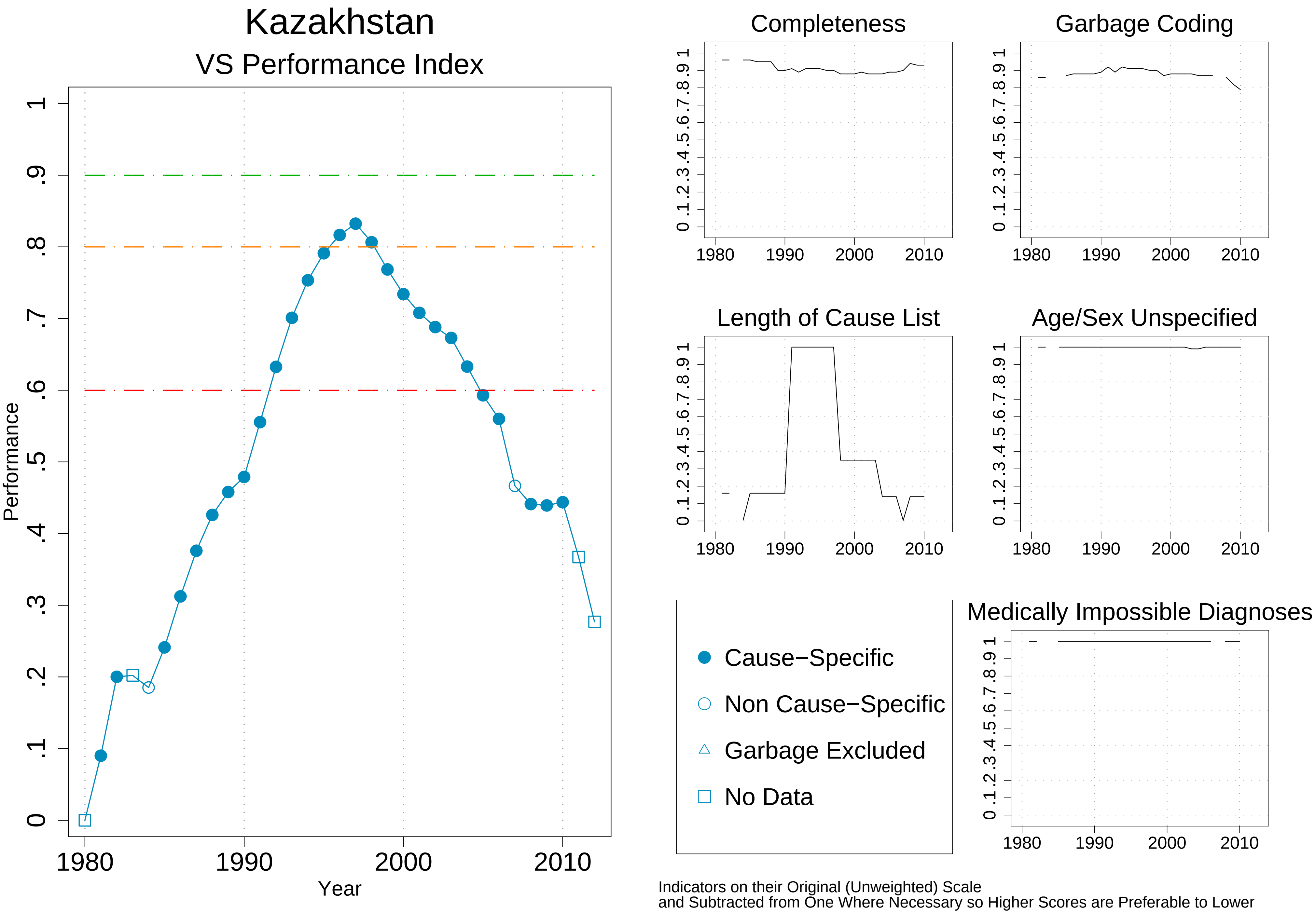

Indicators on their Original (Unweighted) Scale
and Subtracted from One Where Necessary so Higher Scores are Preferable to Lower

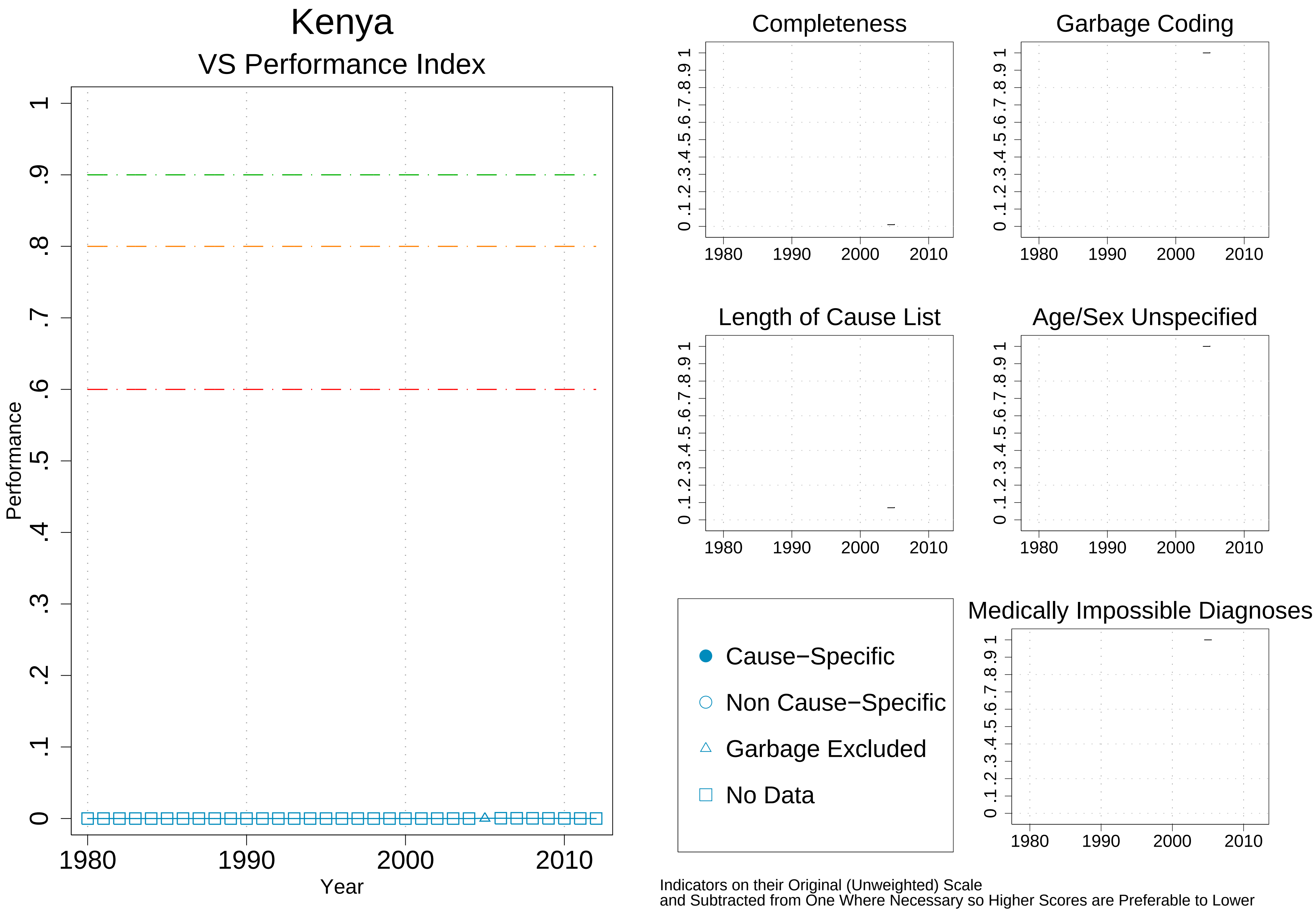

Indicators on their Original (Unweighted) Scale
and Subtracted from One Where Necessary so Higher Scores are Preferable to Lower

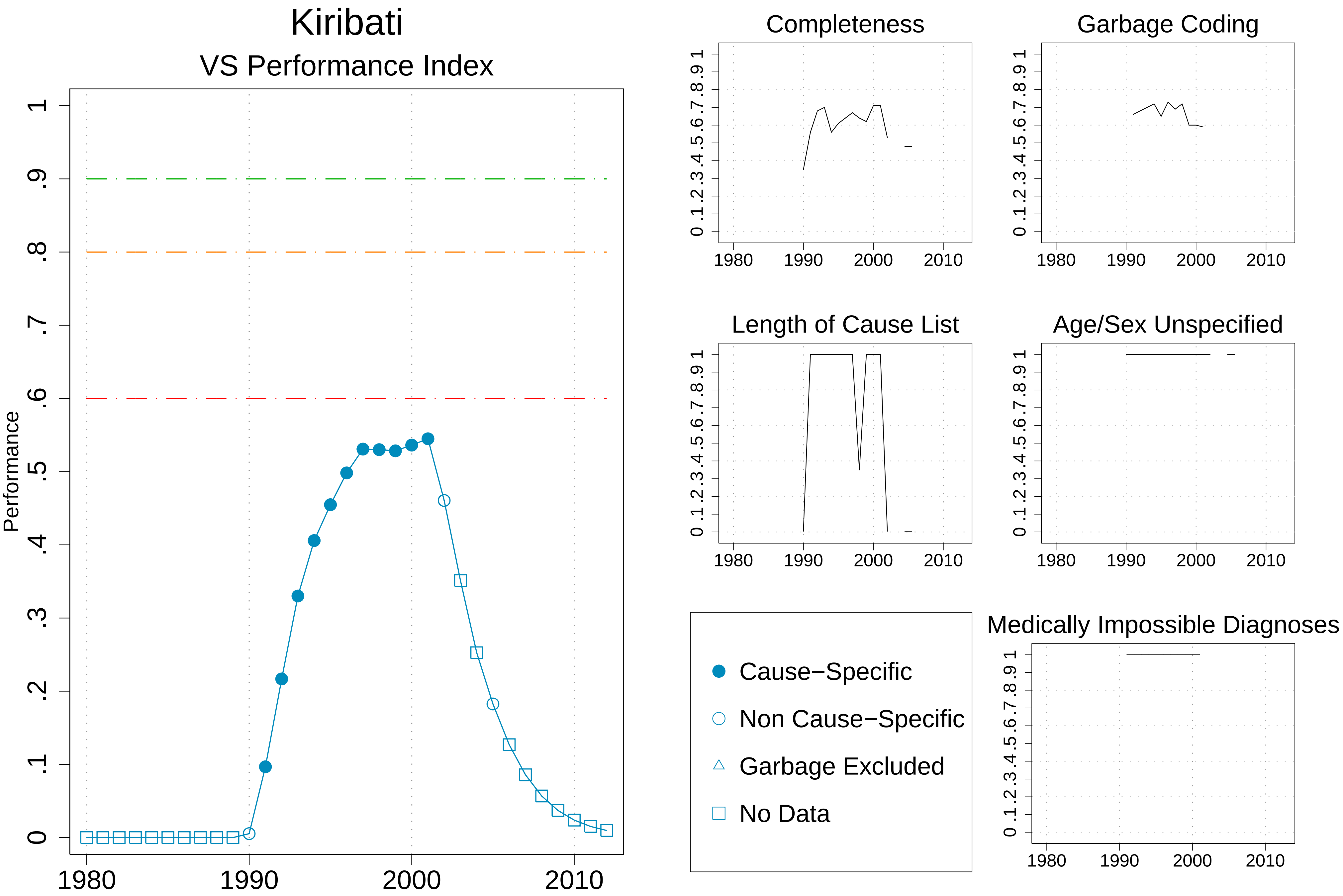

Indicators on their Original (Unweighted) Scale
and Subtracted from One Where Necessary so Higher Scores are Preferable to Lower

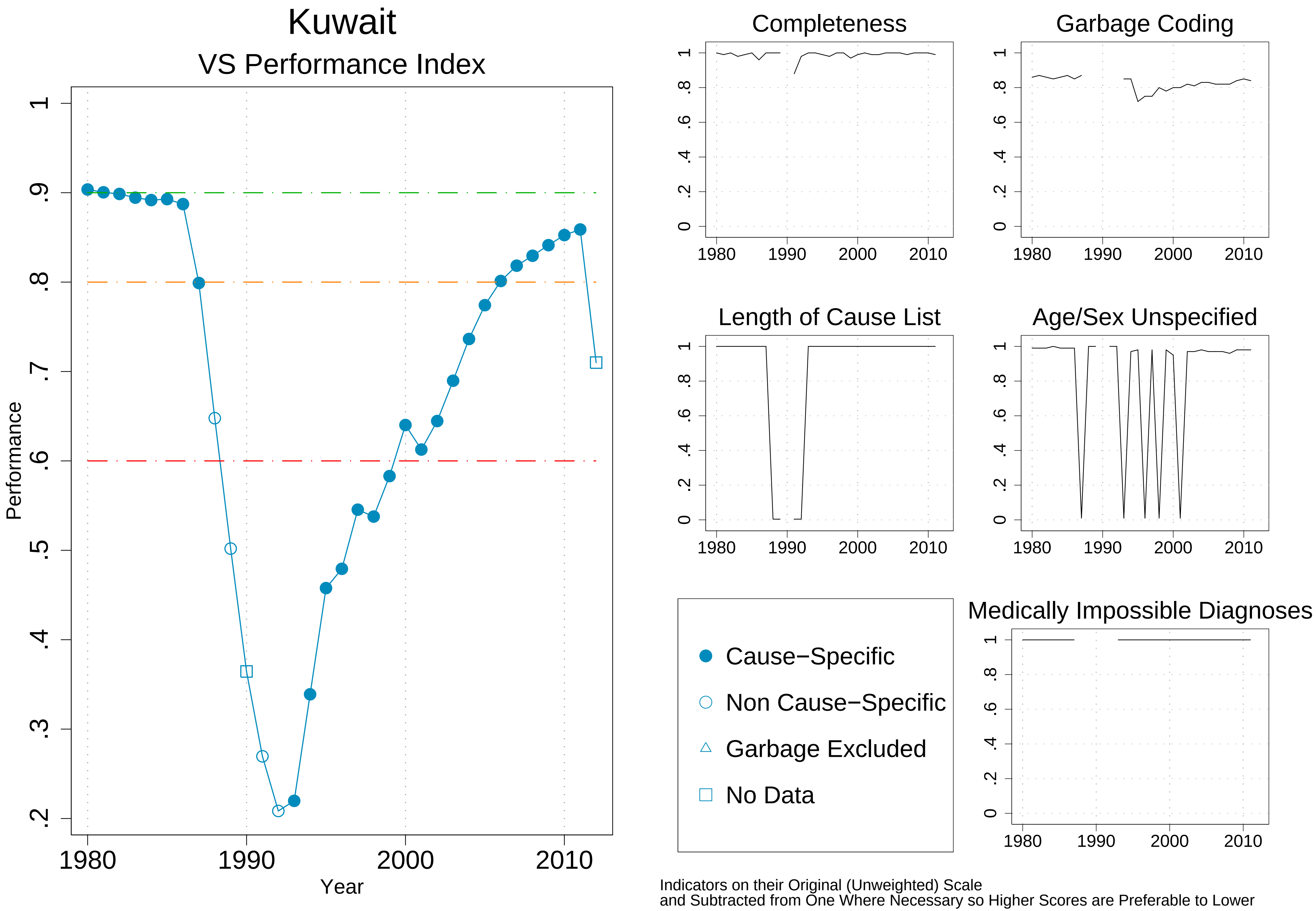

Indicators on their Original (Unweighted) Scale
and Subtracted from One Where Necessary so Higher Scores are Preferable to Lower

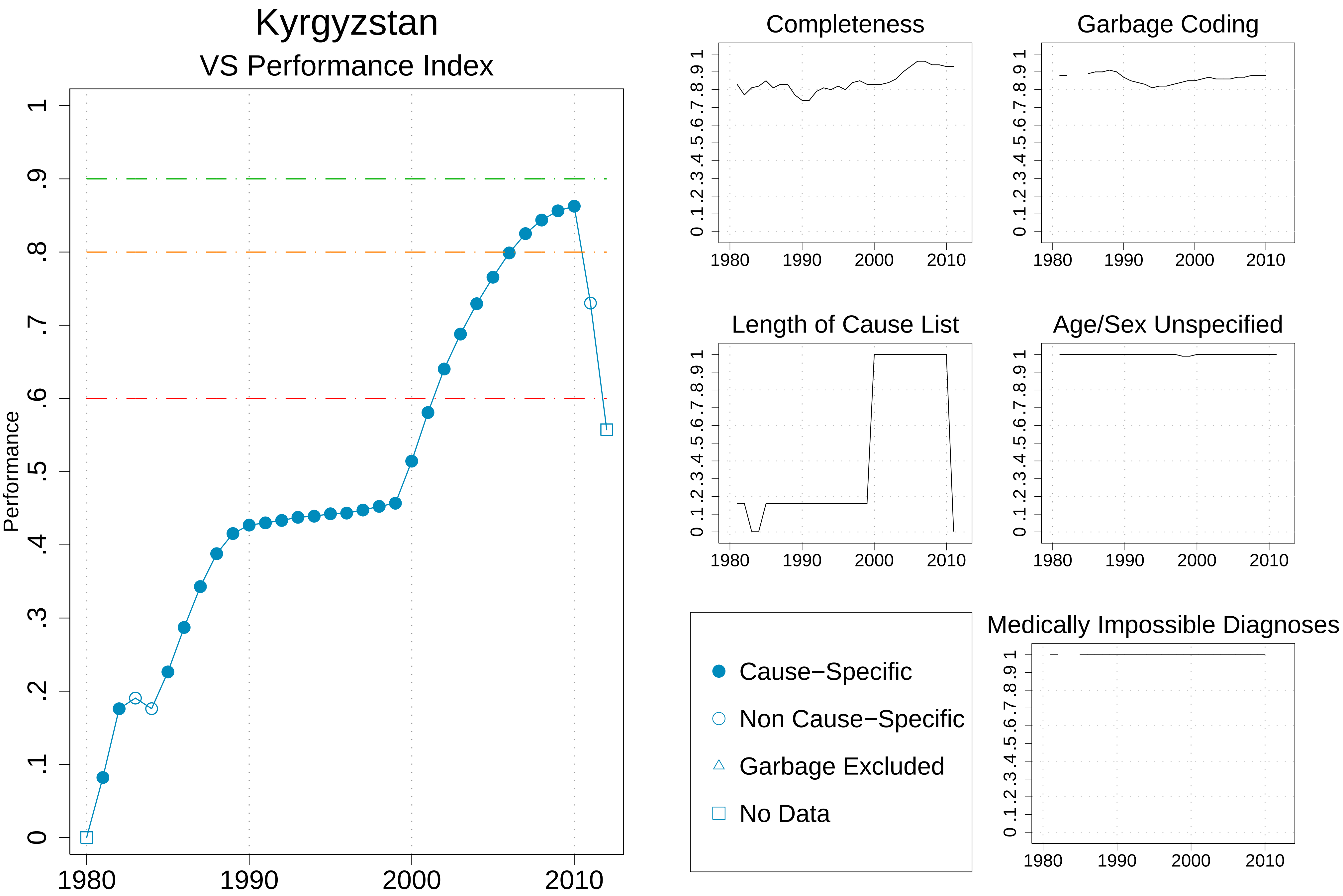

Indicators on their Original (Unweighted) Scale
and Subtracted from One Where Necessary so Higher Scores are Preferable to Lower

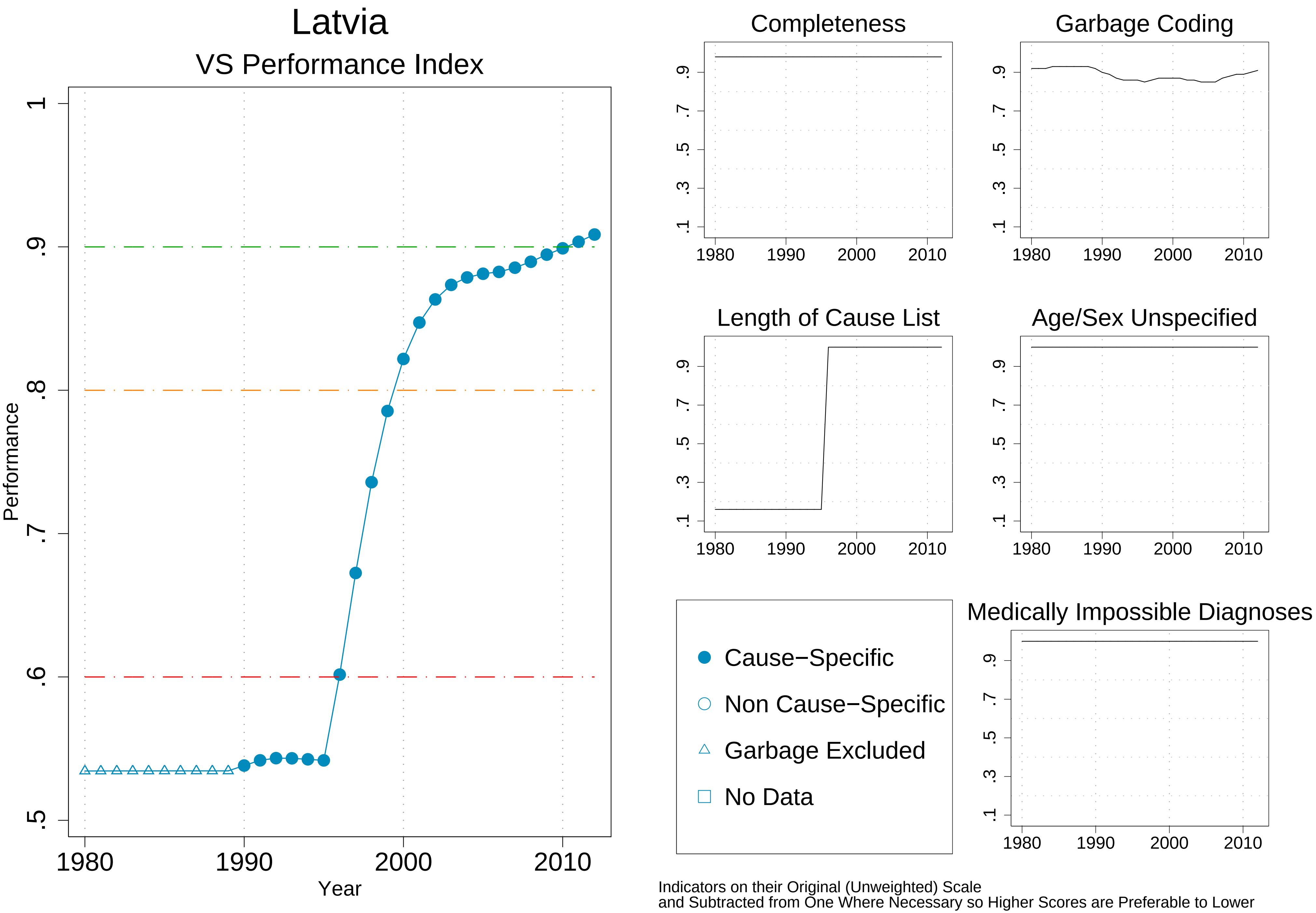

Indicators on their Original (Unweighted) Scale
and Subtracted from One Where Necessary so Higher Scores are Preferable to Lower

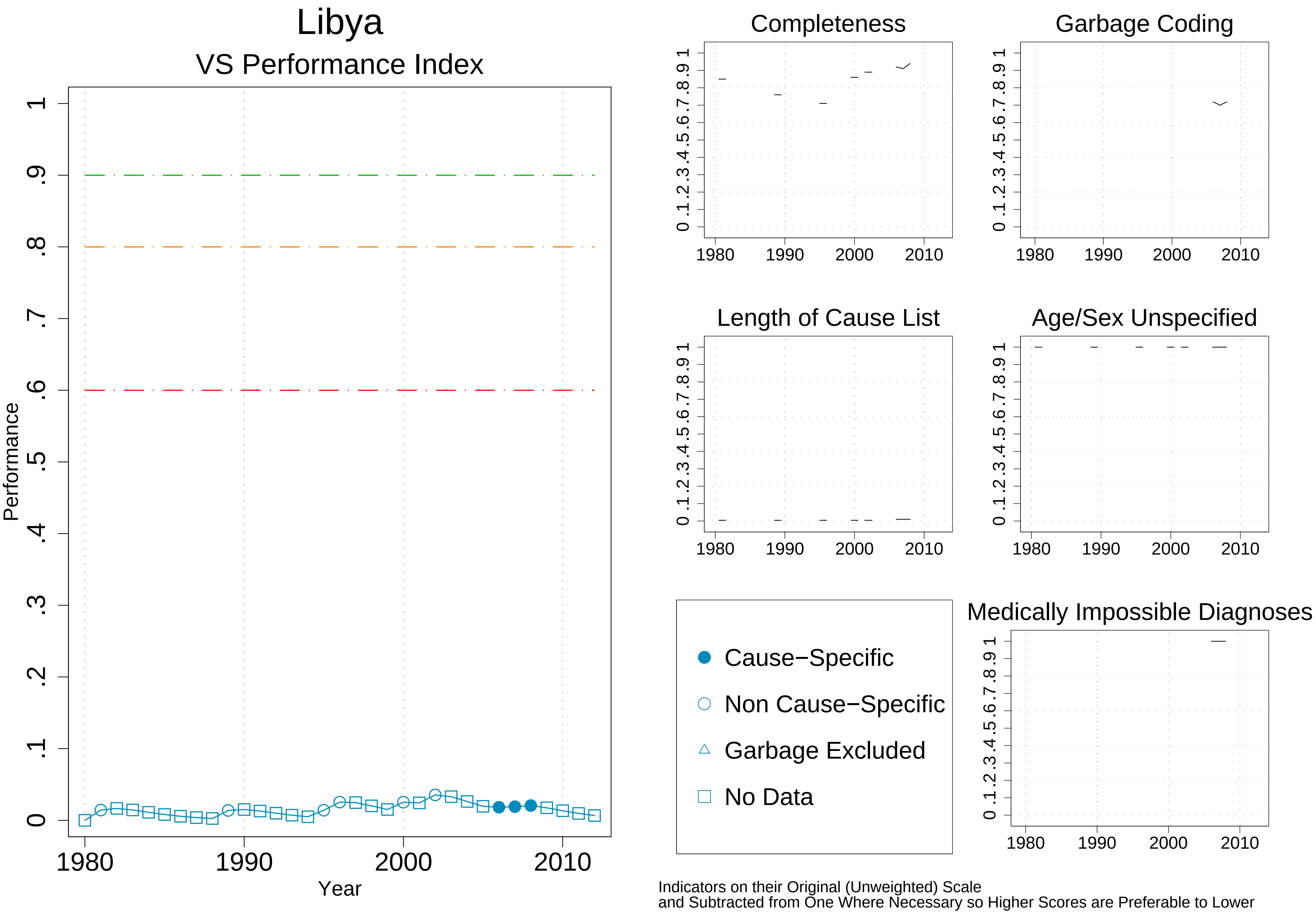

Indicators on their Original (Unweighted) Scale
and Subtracted from One Where Necessary so Higher Scores are Preferable to Lower

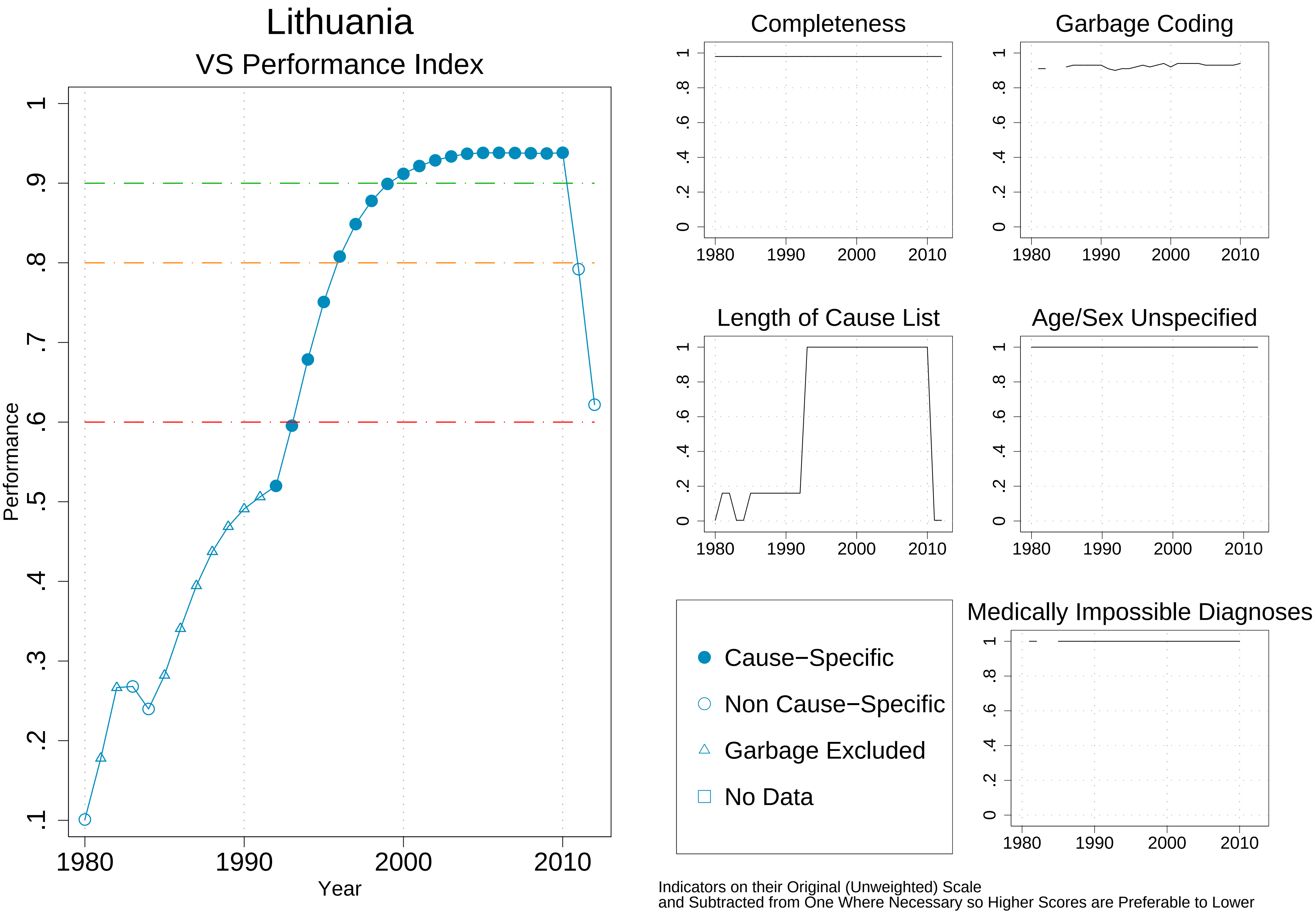

Indicators on their Original (Unweighted) Scale
and Subtracted from One Where Necessary so Higher Scores are Preferable to Lower

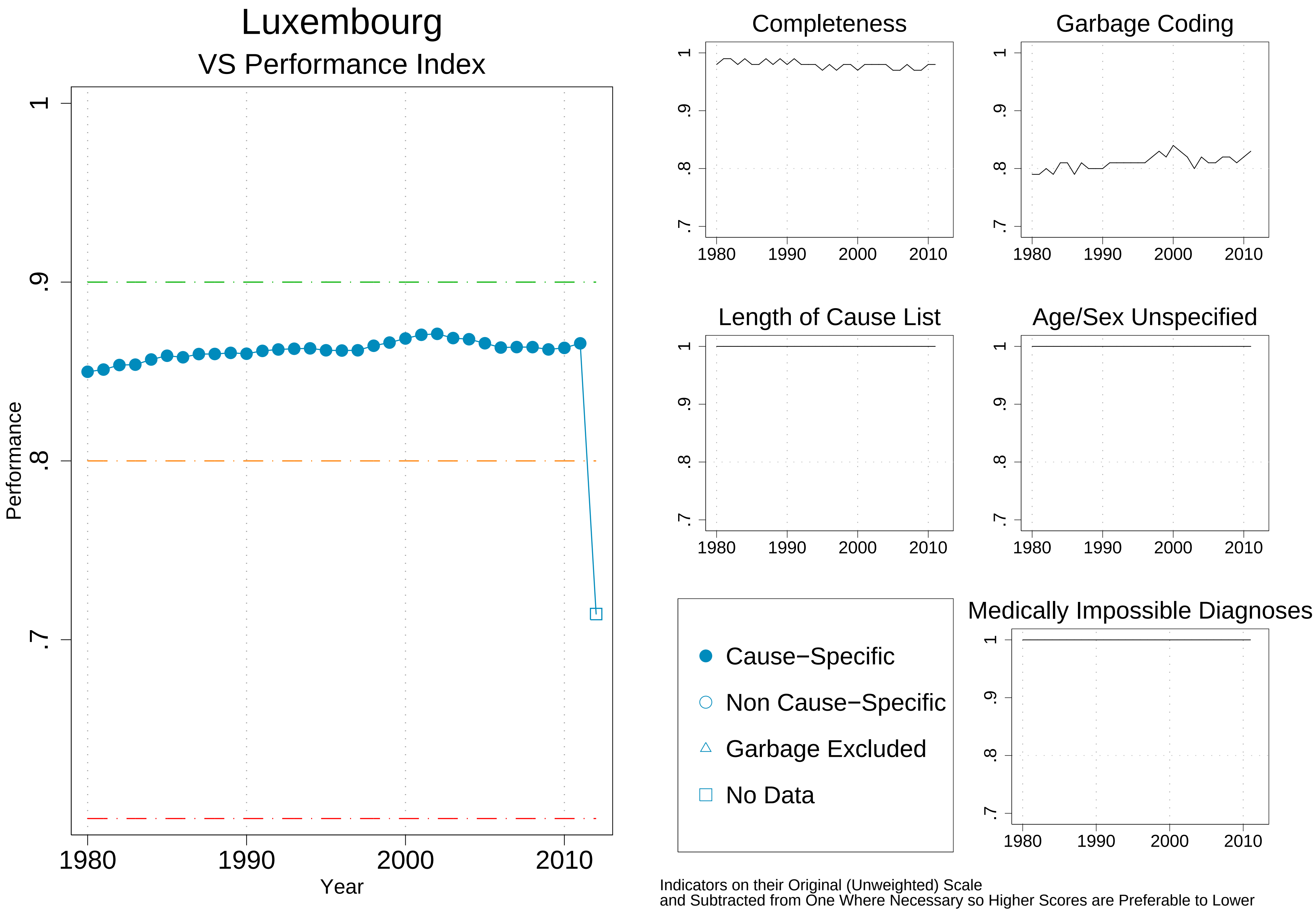

Indicators on their Original (Unweighted) Scale
and Subtracted from One Where Necessary so Higher Scores are Preferable to Lower

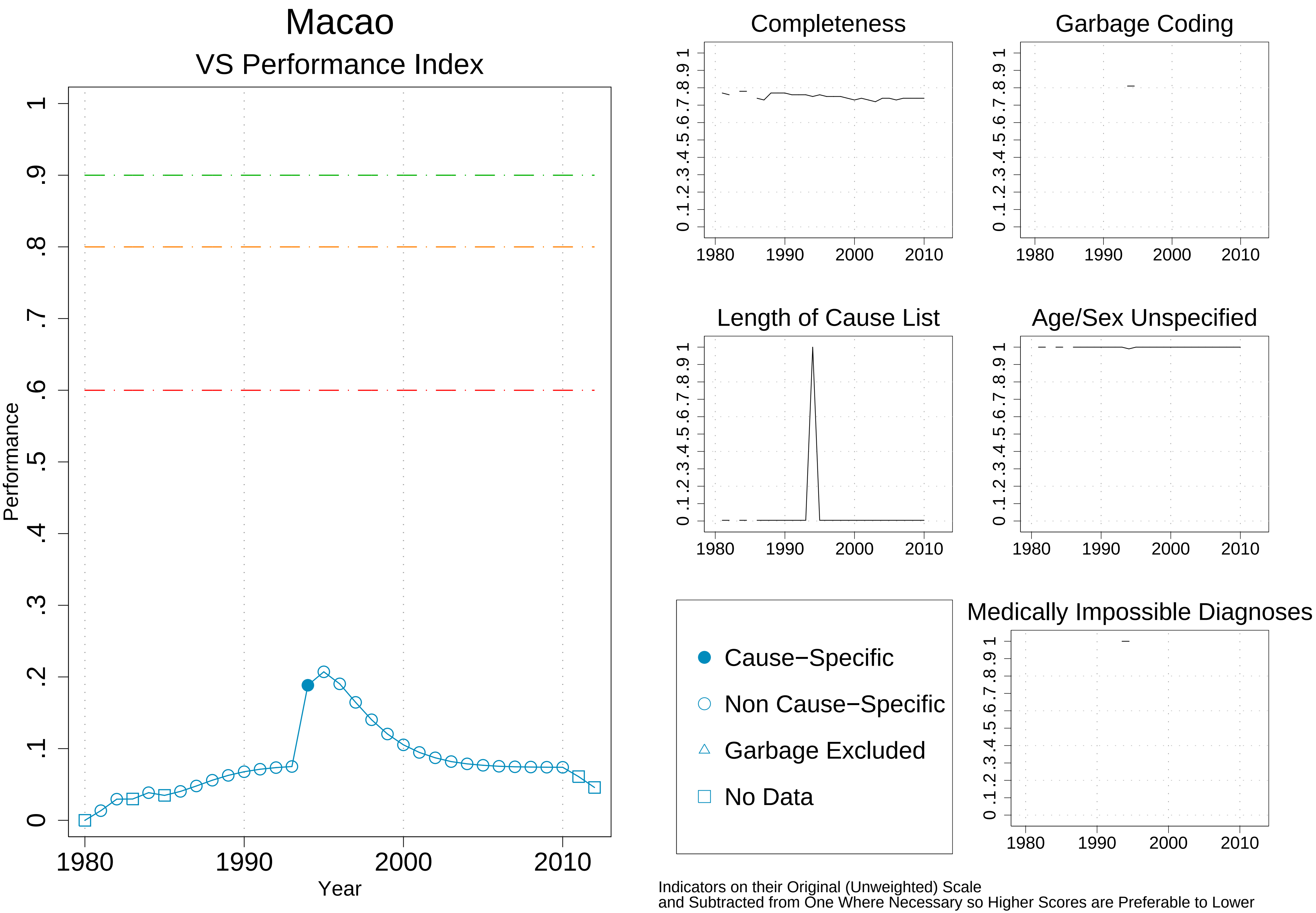

Indicators on their Original (Unweighted) Scale
and Subtracted from One Where Necessary so Higher Scores are Preferable to Lower

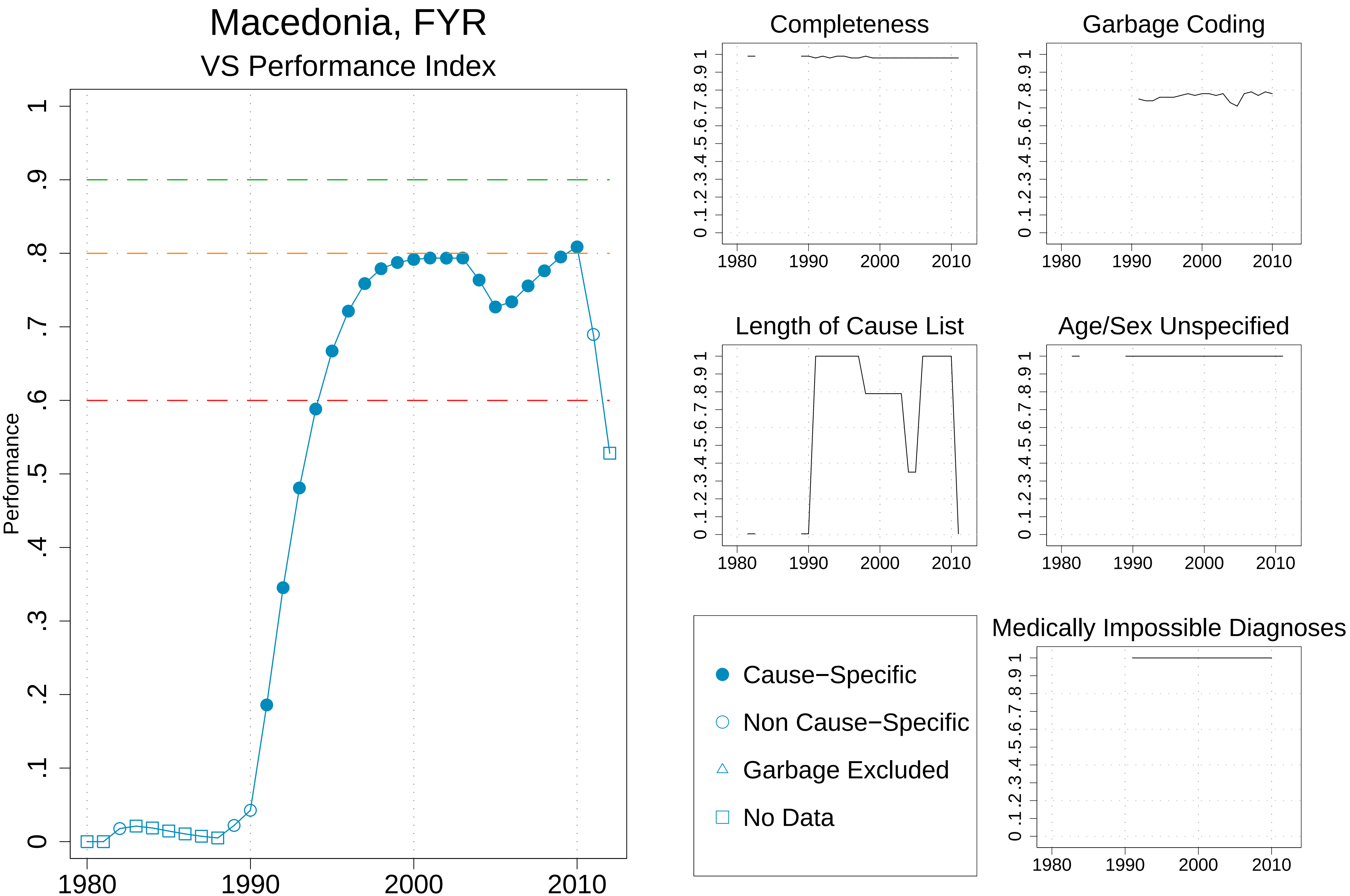

Indicators on their Original (Unweighted) Scale
and Subtracted from One Where Necessary so Higher Scores are Preferable to Lower

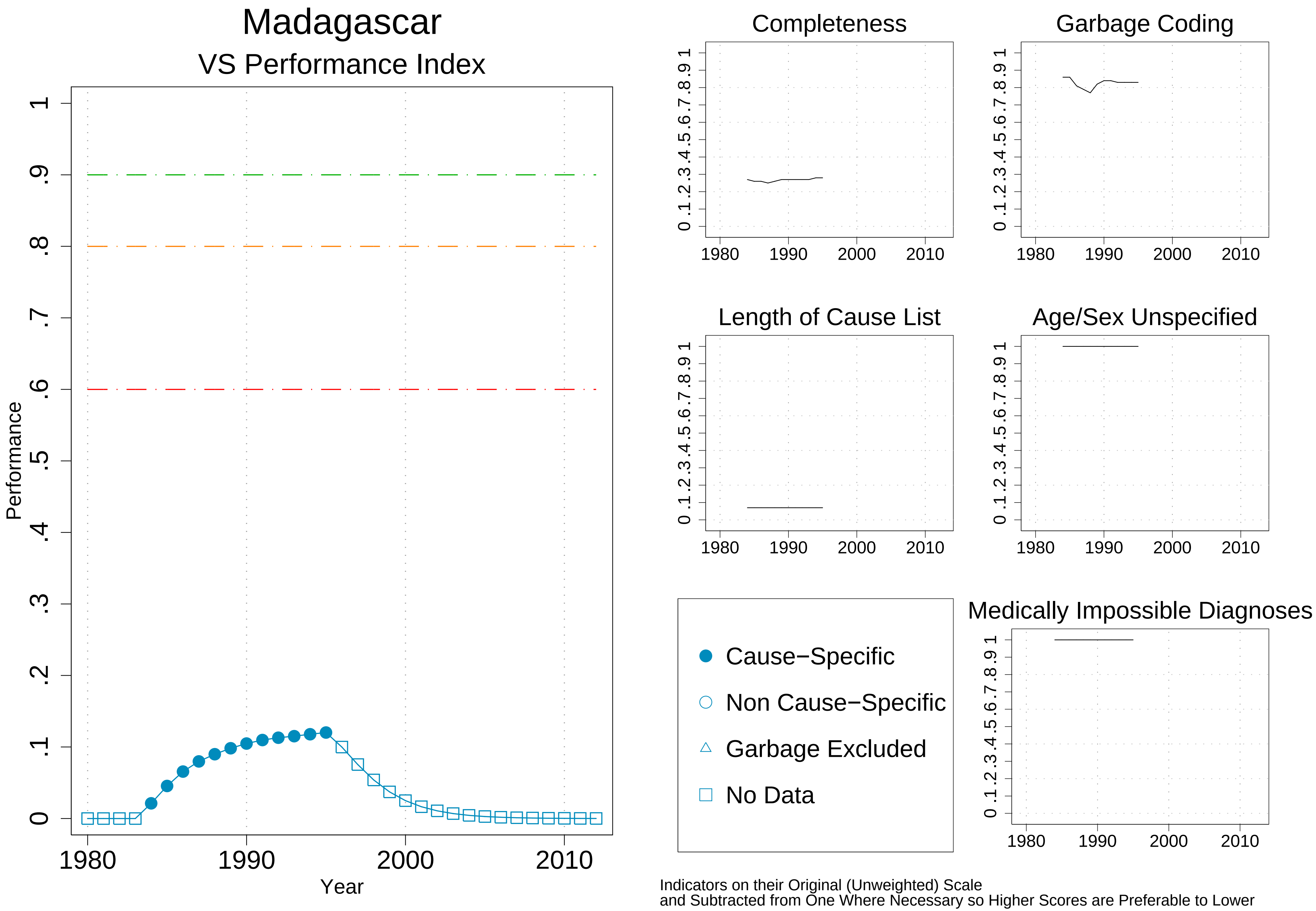

Indicators on their Original (Unweighted) Scale
and Subtracted from One Where Necessary so Higher Scores are Preferable to Lower

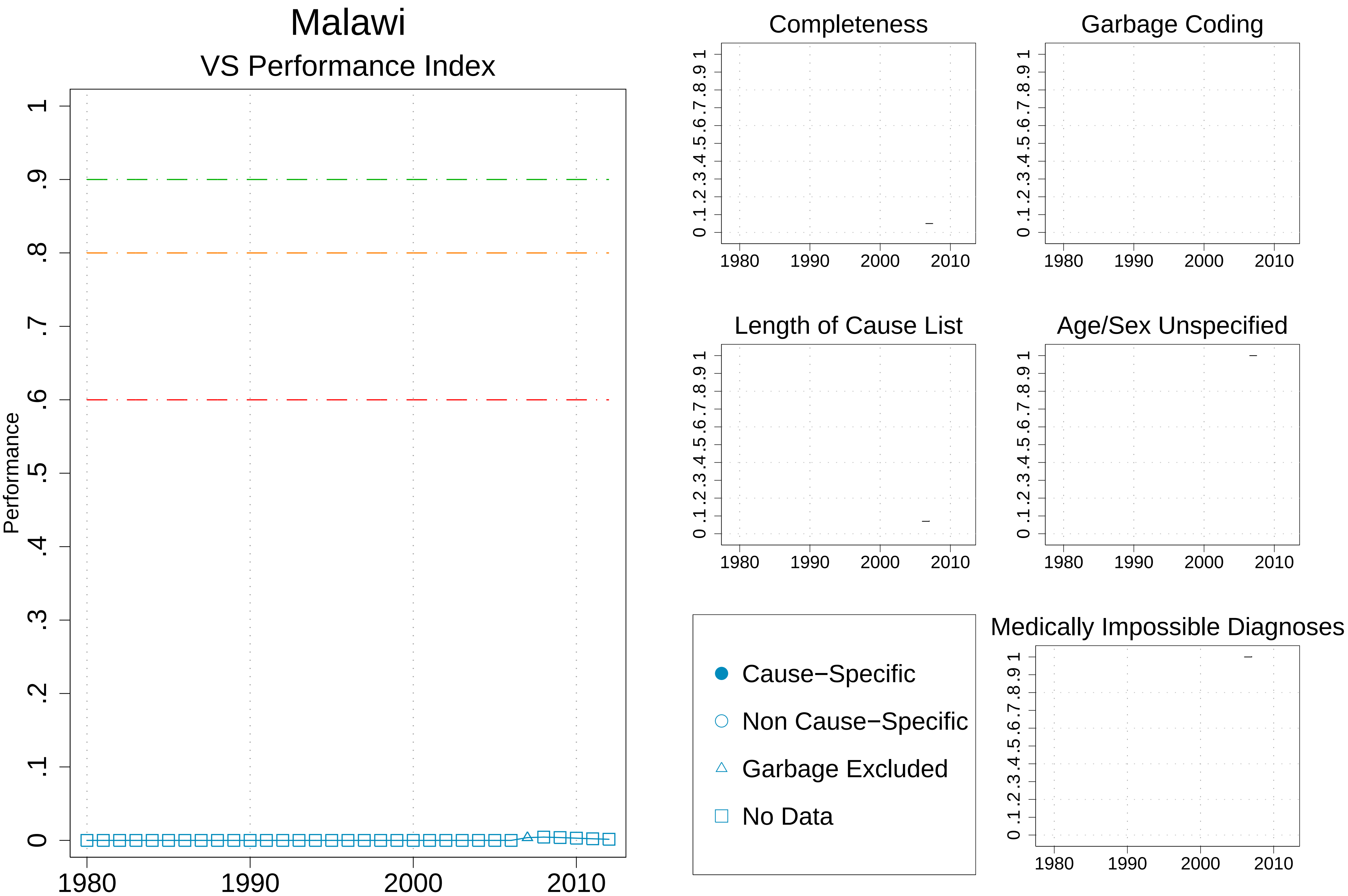

Indicators on their Original (Unweighted) Scale
and Subtracted from One Where Necessary so Higher Scores are Preferable to Lower

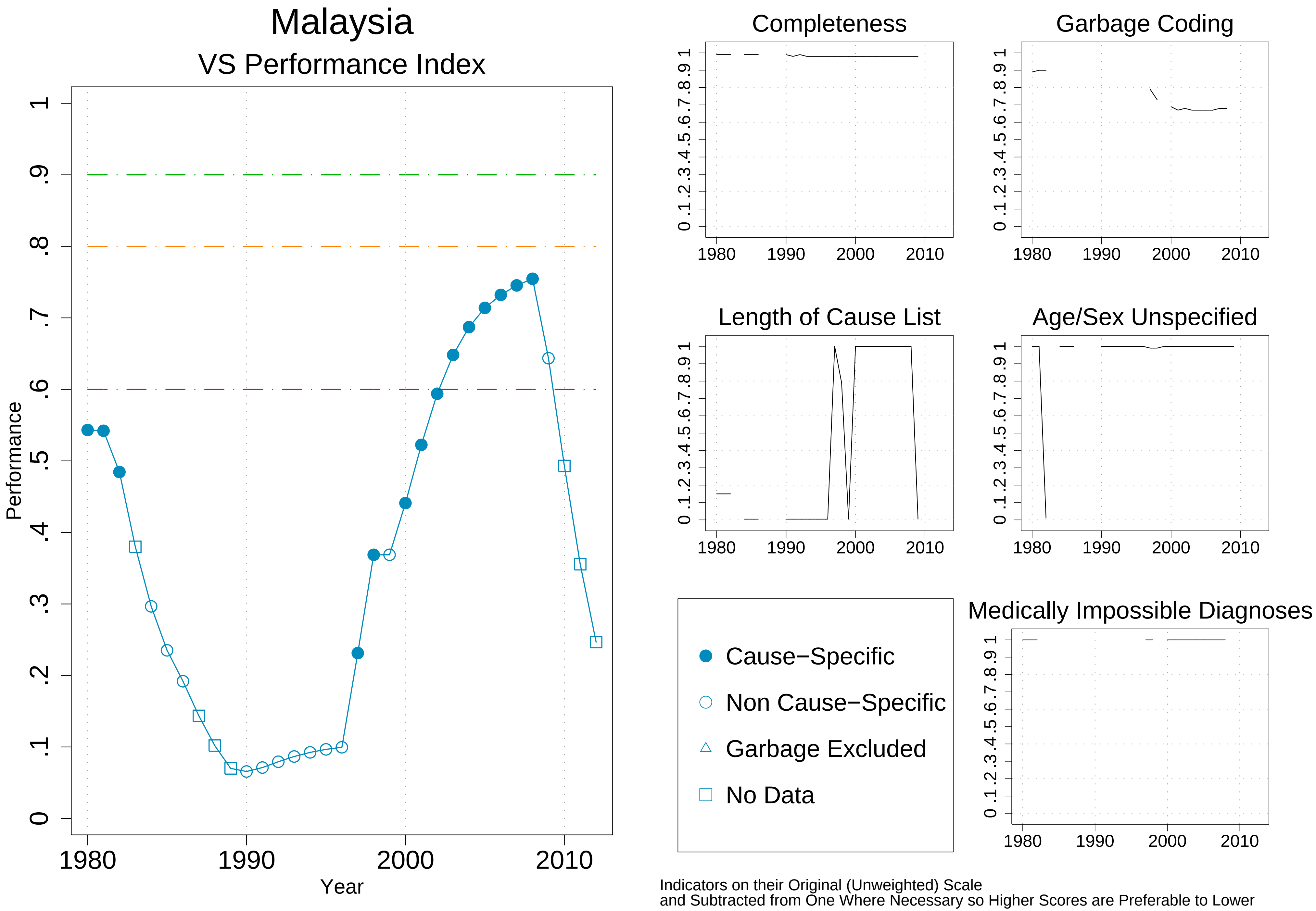

Indicators on their Original (Unweighted) Scale
and Subtracted from One Where Necessary so Higher Scores are Preferable to Lower

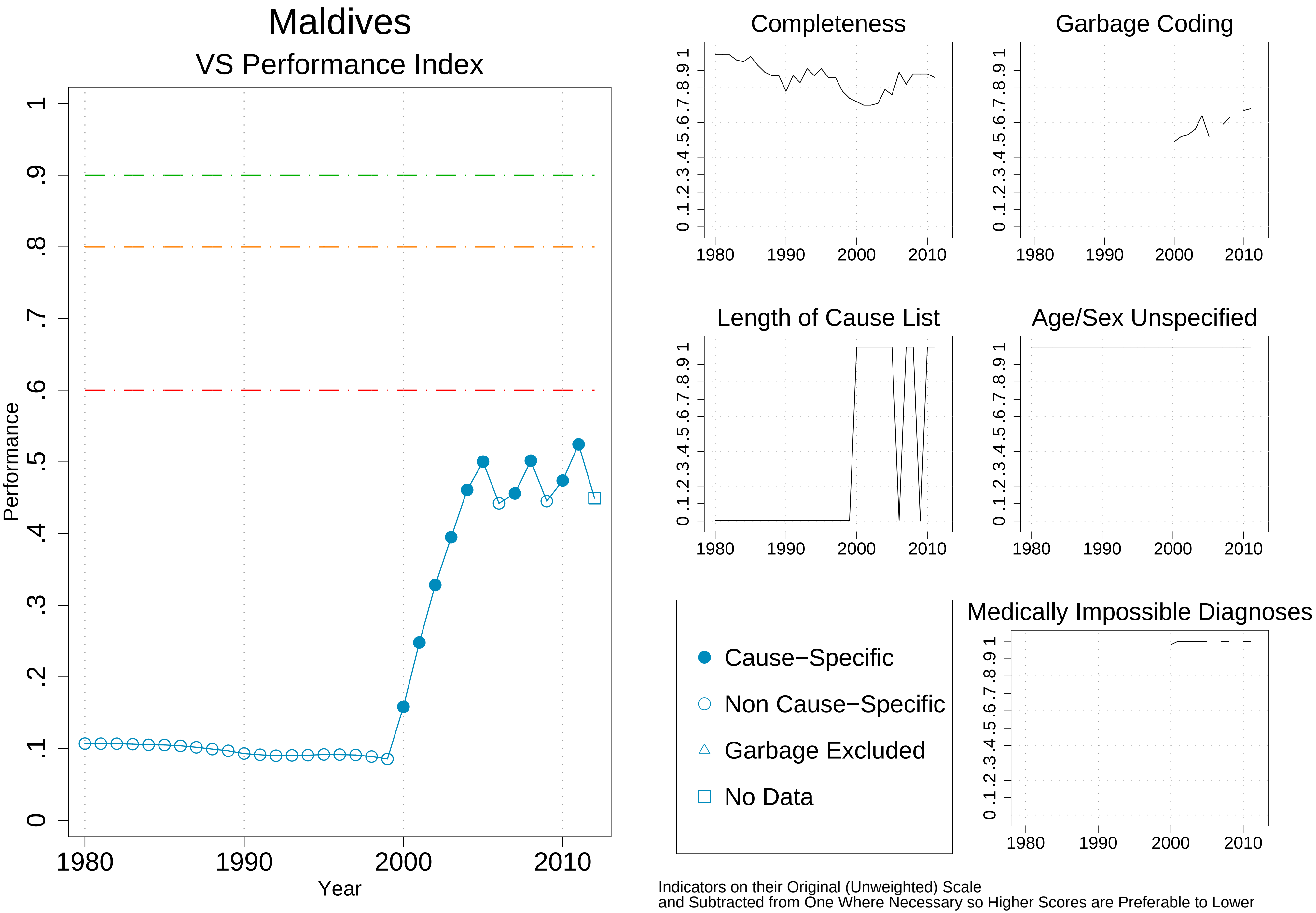

Indicators on their Original (Unweighted) Scale
and Subtracted from One Where Necessary so Higher Scores are Preferable to Lower

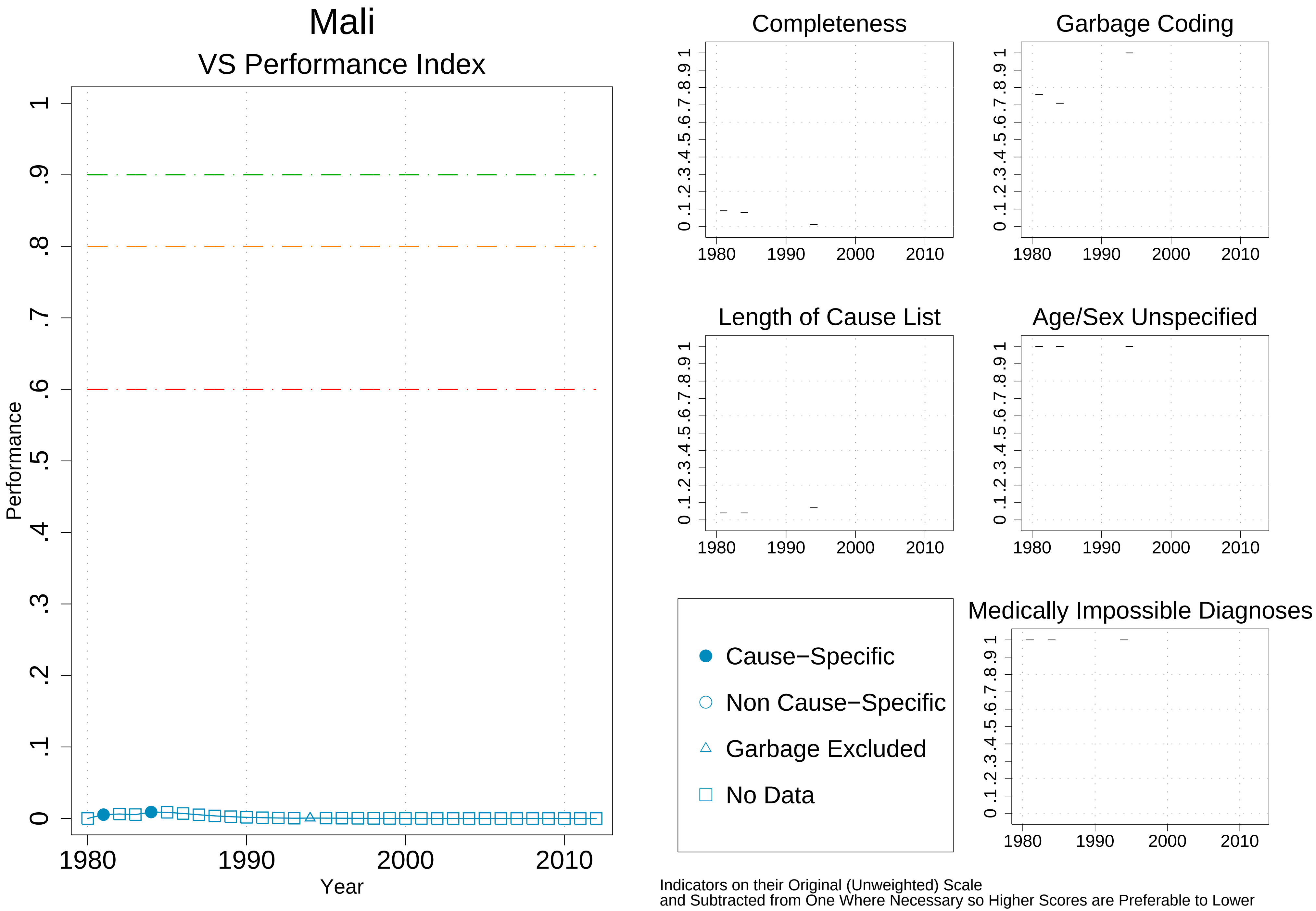

Indicators on their Original (Unweighted) Scale
and Subtracted from One Where Necessary so Higher Scores are Preferable to Lower

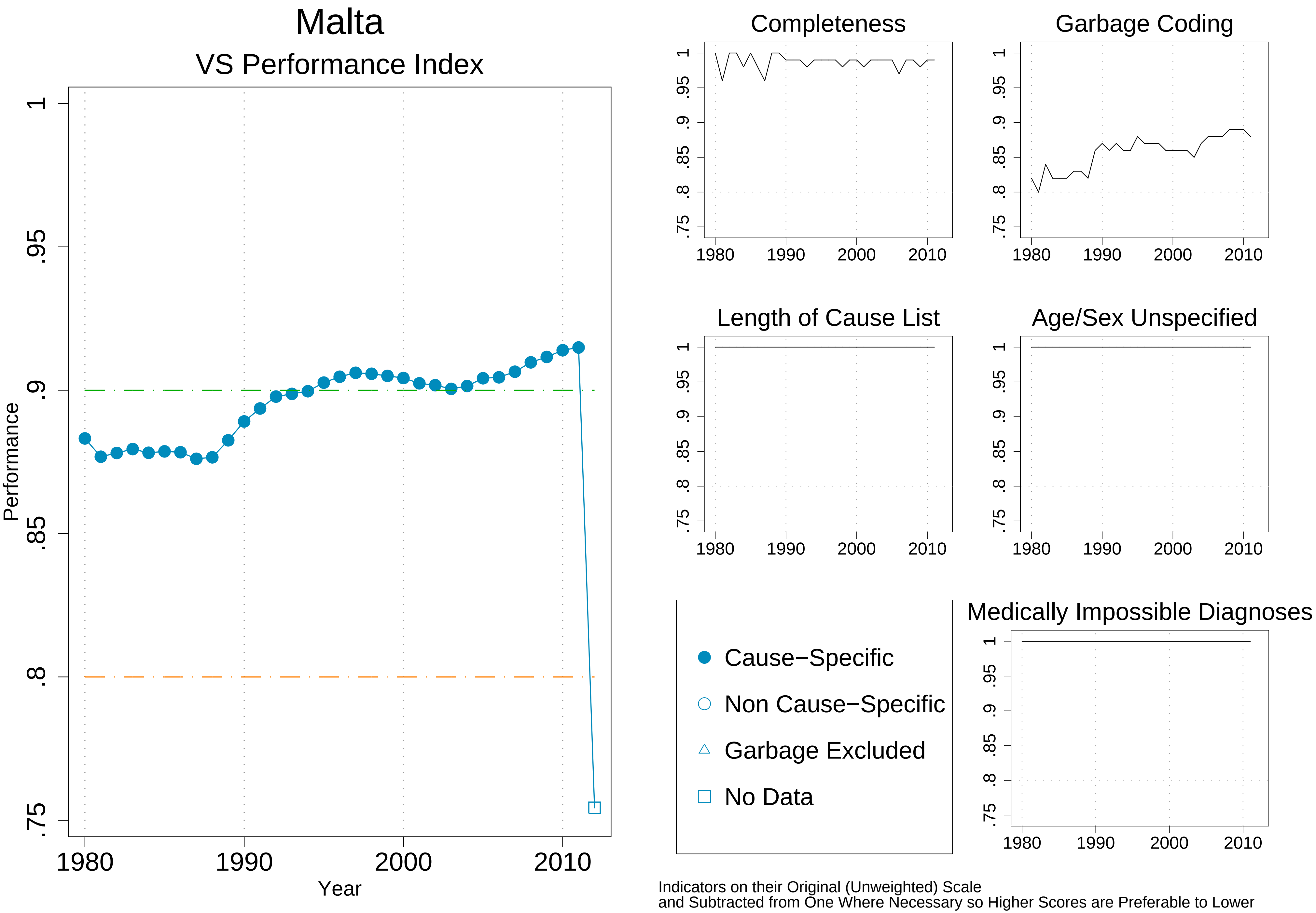

Indicators on their Original (Unweighted) Scale
and Subtracted from One Where Necessary so Higher Scores are Preferable to Lower

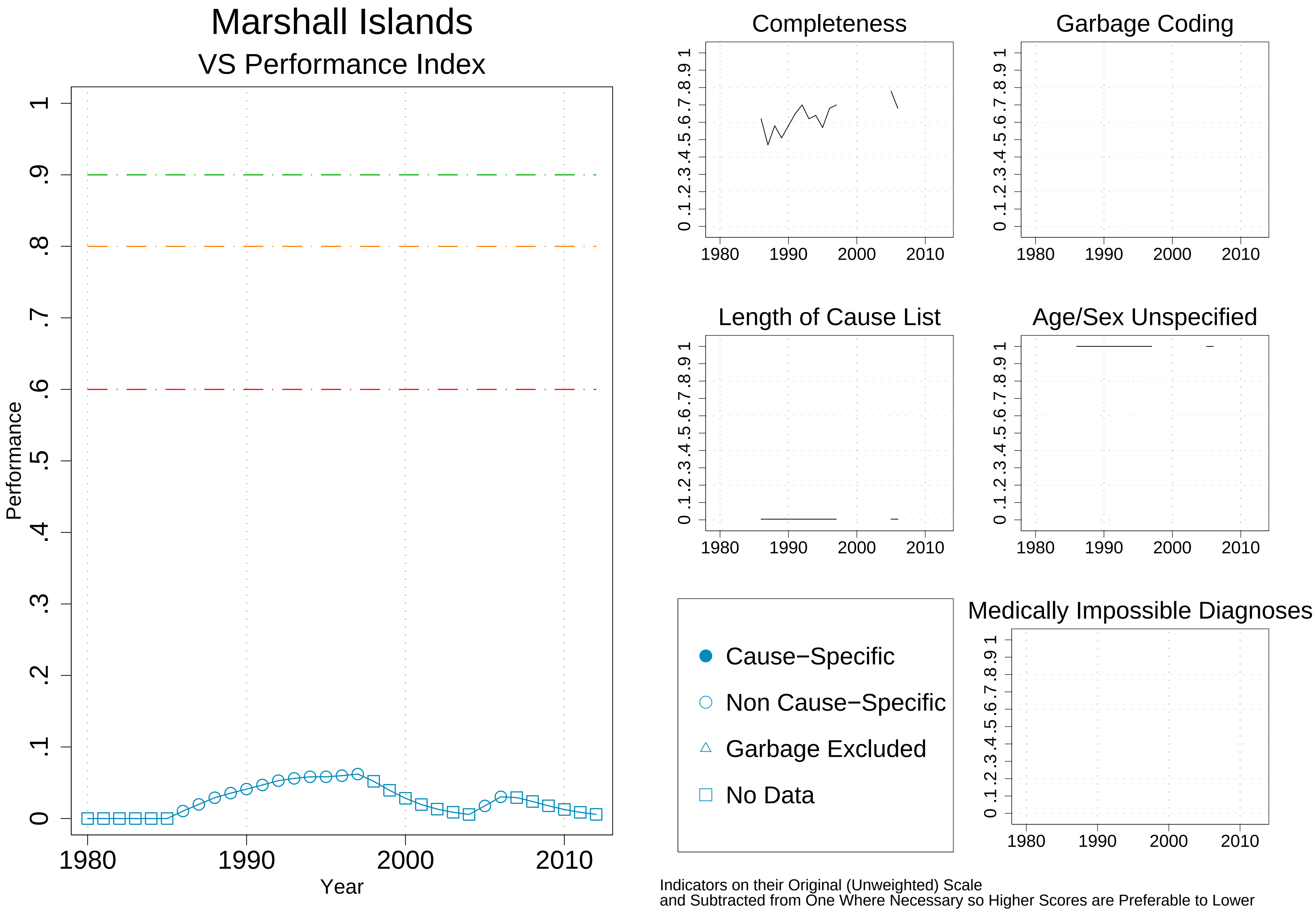

Indicators on their Original (Unweighted) Scale
and Subtracted from One Where Necessary so Higher Scores are Preferable to Lower

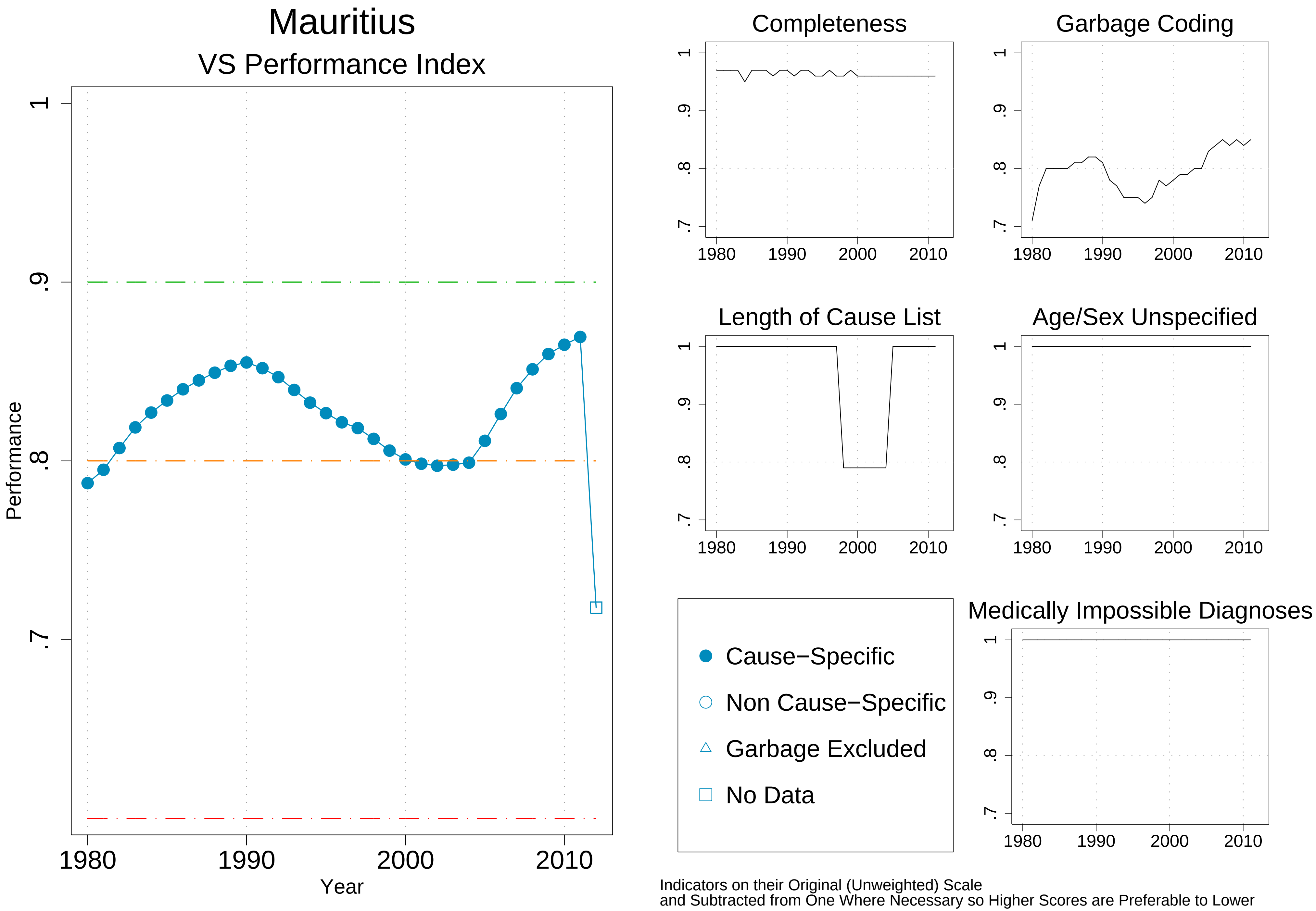

Indicators on their Original (Unweighted) Scale
and Subtracted from One Where Necessary so Higher Scores are Preferable to Lower

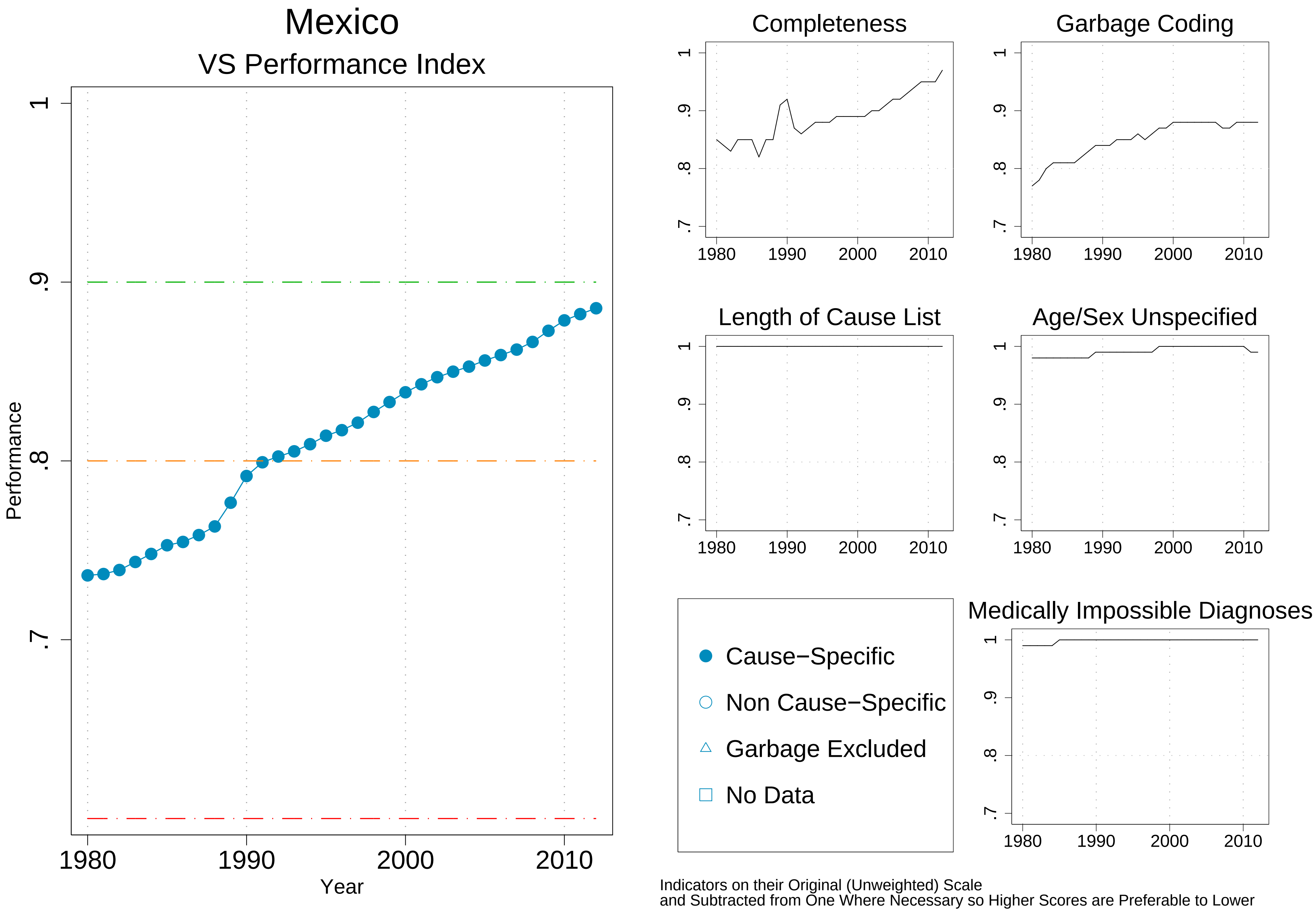

Indicators on their Original (Unweighted) Scale
and Subtracted from One Where Necessary so Higher Scores are Preferable to Lower

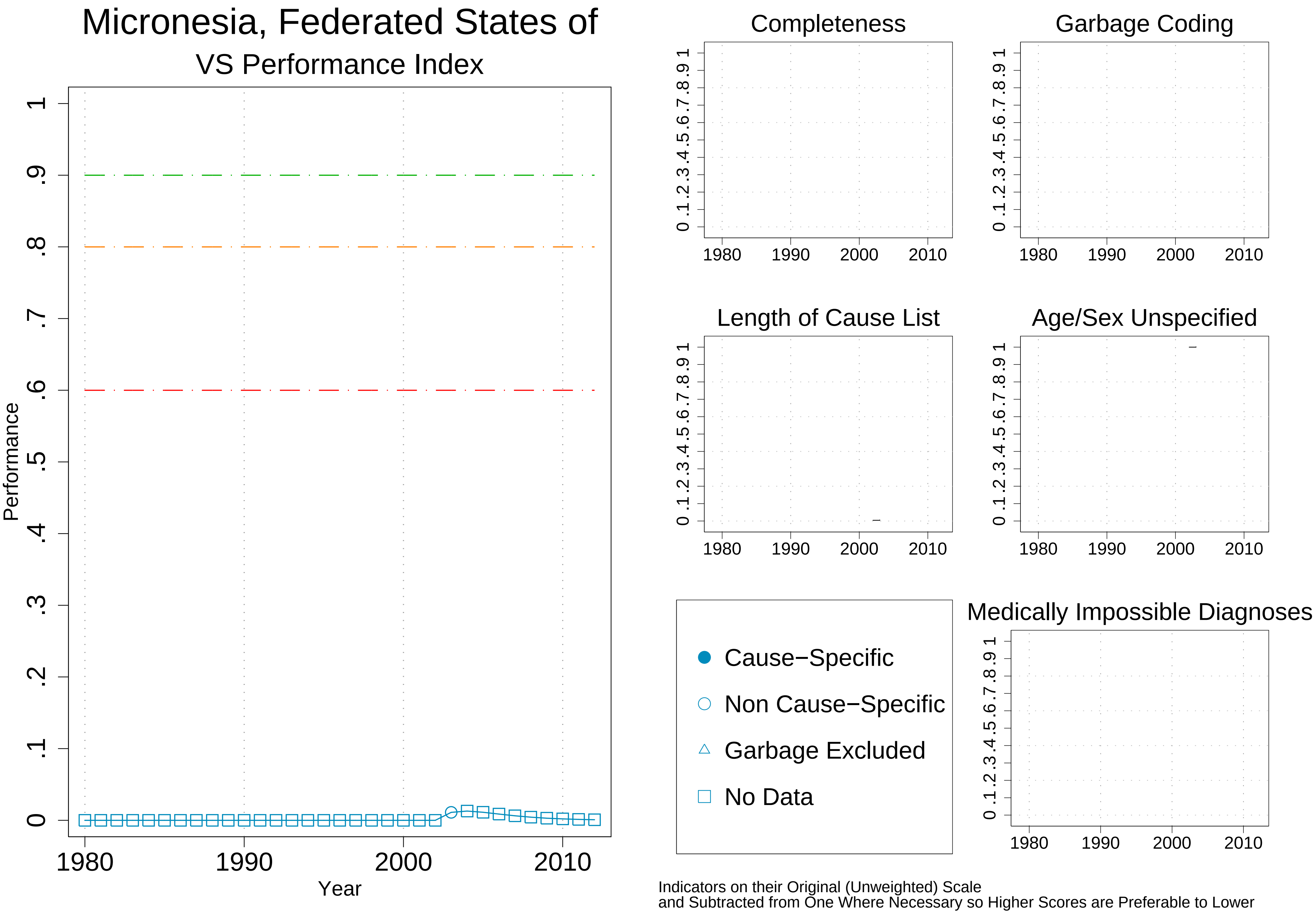

Indicators on their Original (Unweighted) Scale
and Subtracted from One Where Necessary so Higher Scores are Preferable to Lower

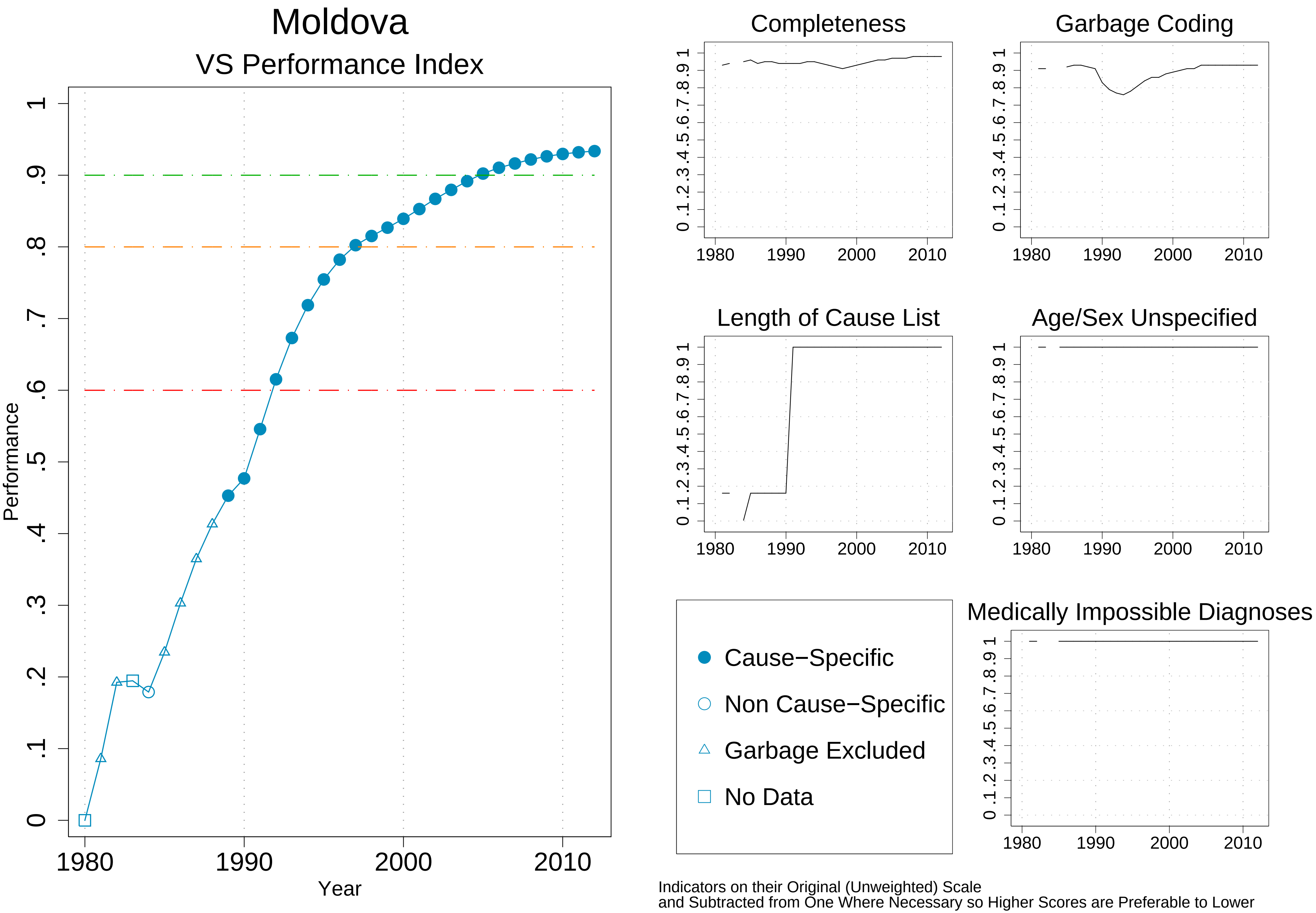

Indicators on their Original (Unweighted) Scale
and Subtracted from One Where Necessary so Higher Scores are Preferable to Lower

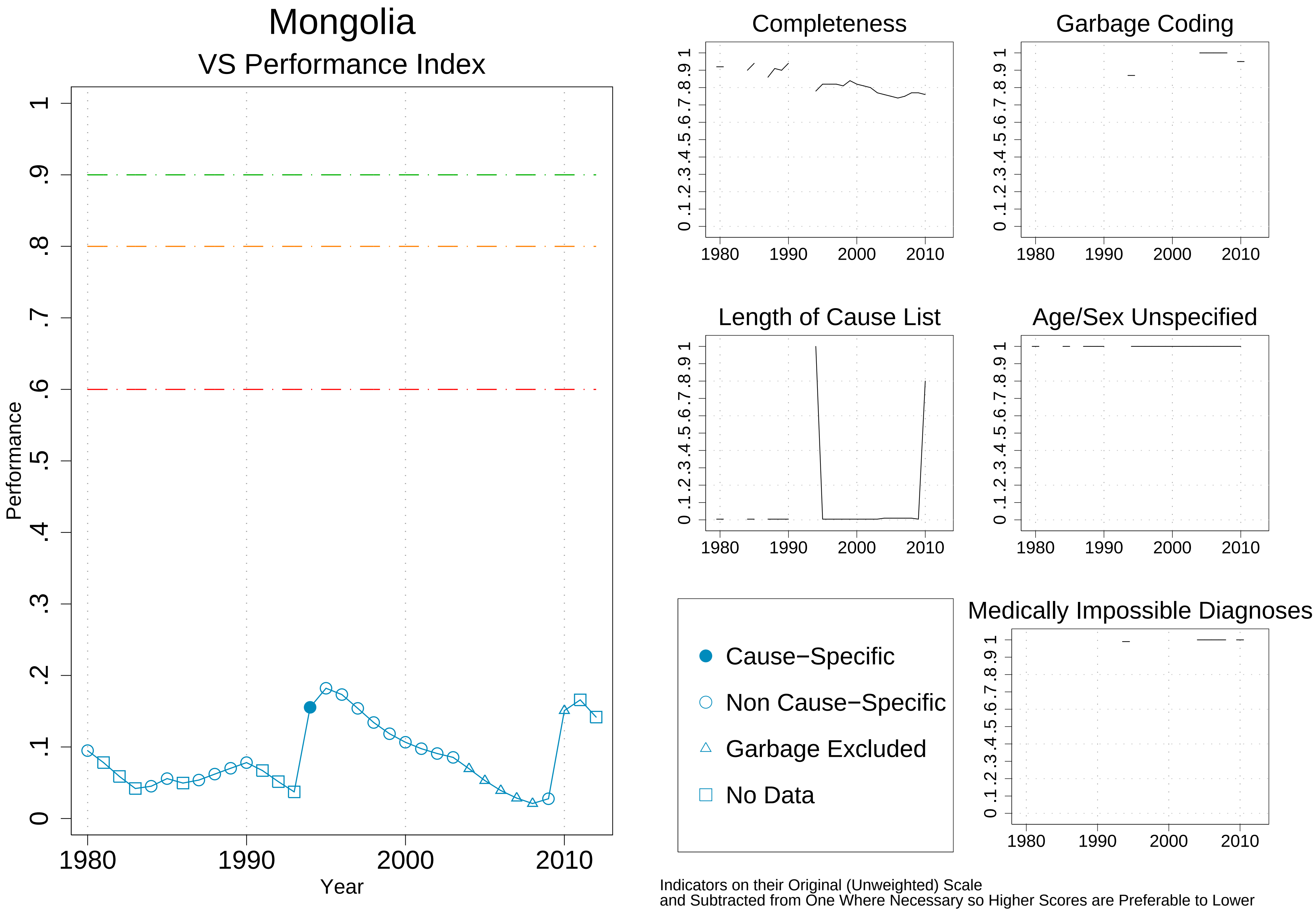

Indicators on their Original (Unweighted) Scale
and Subtracted from One Where Necessary so Higher Scores are Preferable to Lower

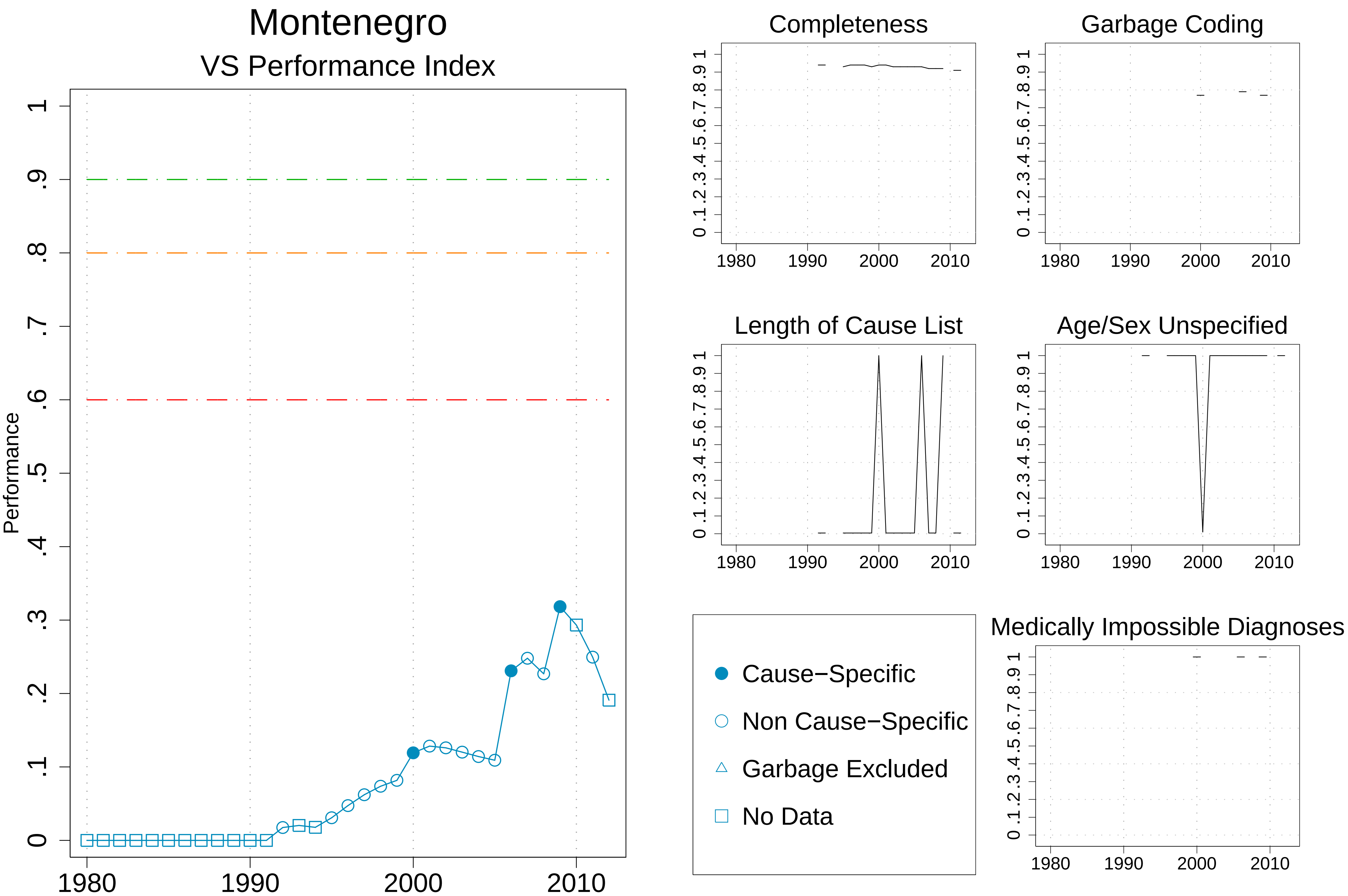

Indicators on their Original (Unweighted) Scale
and Subtracted from One Where Necessary so Higher Scores are Preferable to Lower

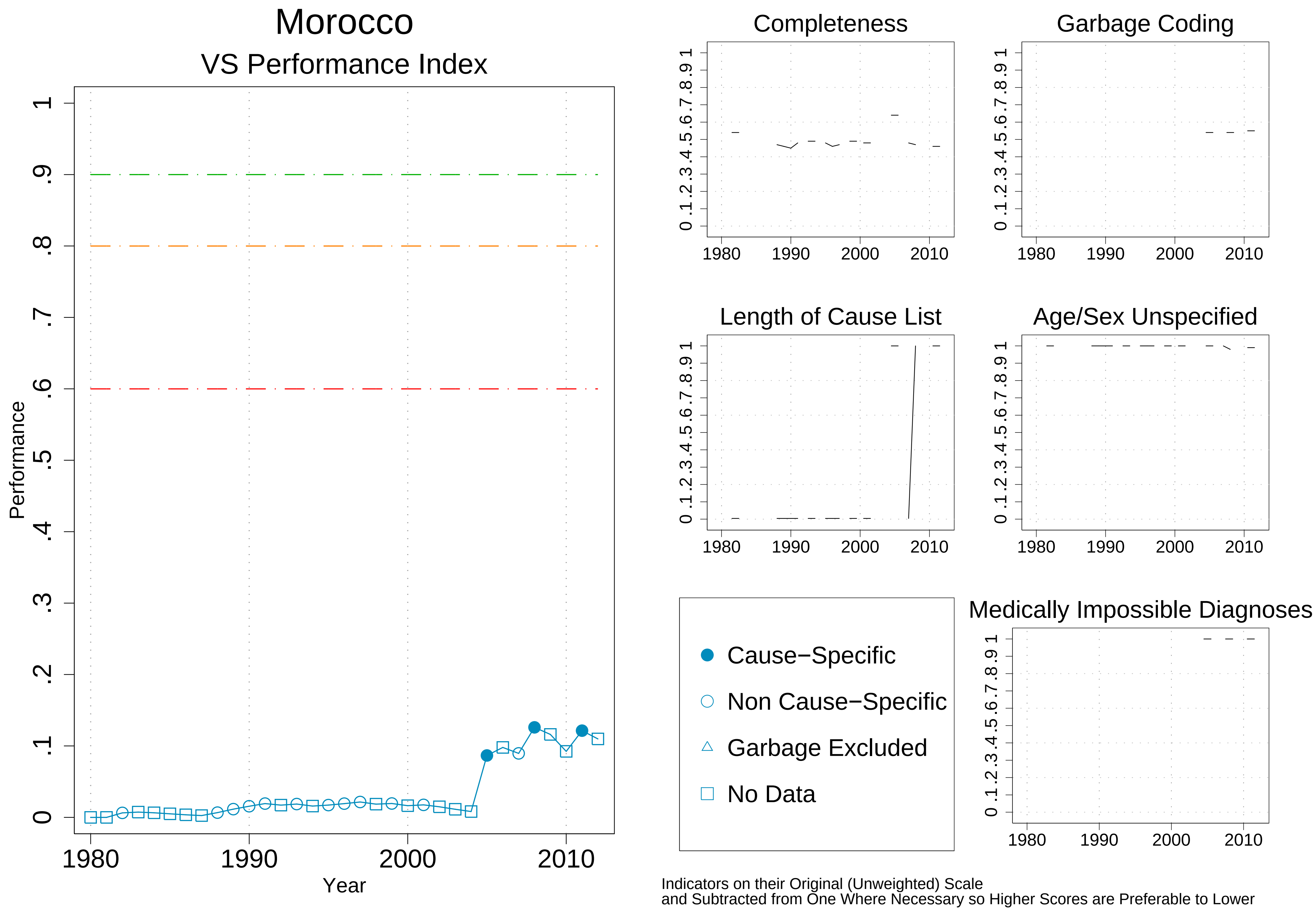
Morocco
VS Performance Index
Performance
Year
Completeness
Garbage Coding
Length of Cause List
Age/Sex Unspecified
Medically Impossible Diagnoses
Cause−Specific
Non Cause−Specific
Garbage Excluded
No Data
Indicators on their Original (Unweighted) Scale
and Subtracted from One Where Necessary so Higher Scores are Preferable to Lower

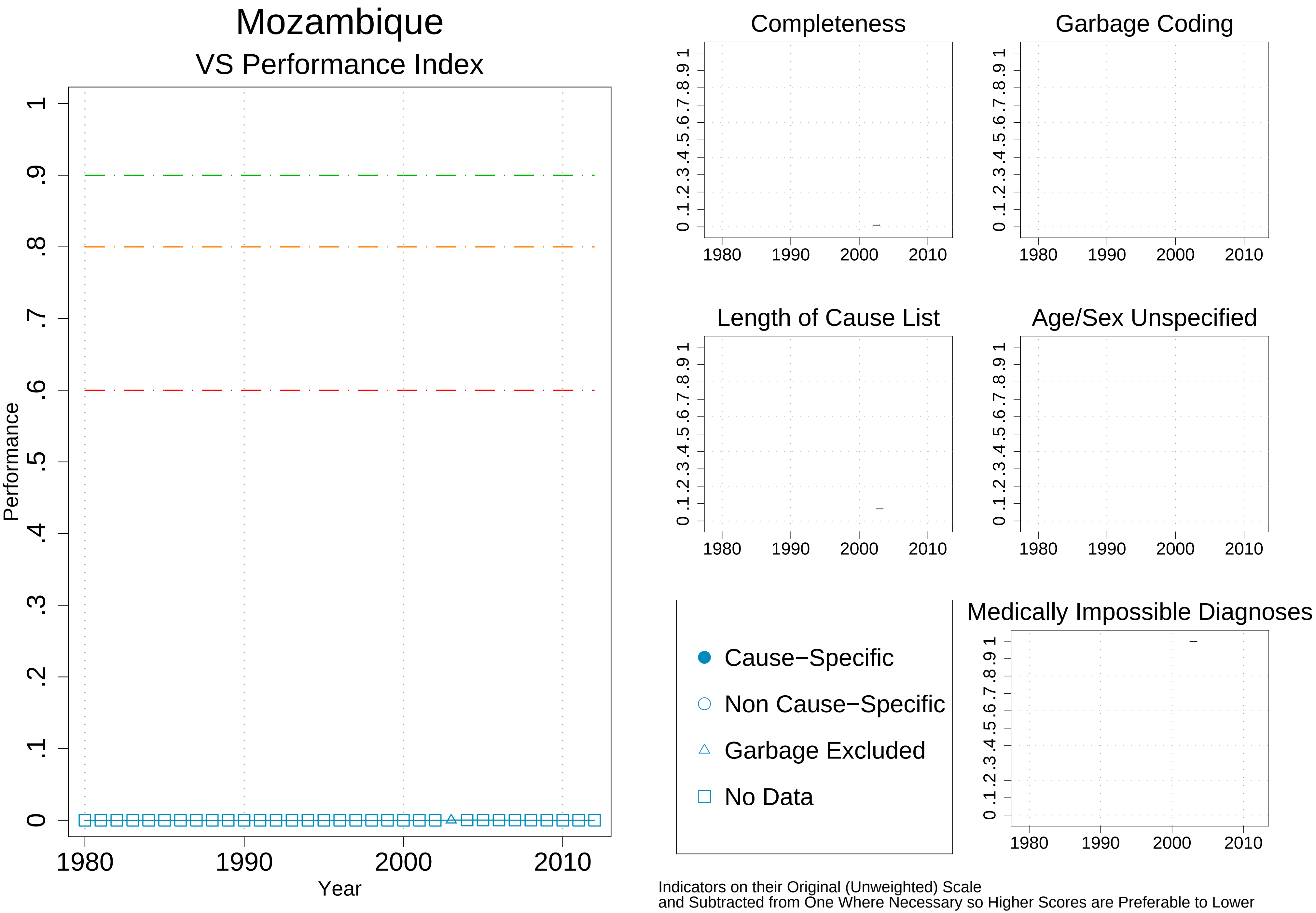

Indicators on their Original (Unweighted) Scale
and Subtracted from One Where Necessary so Higher Scores are Preferable to Lower

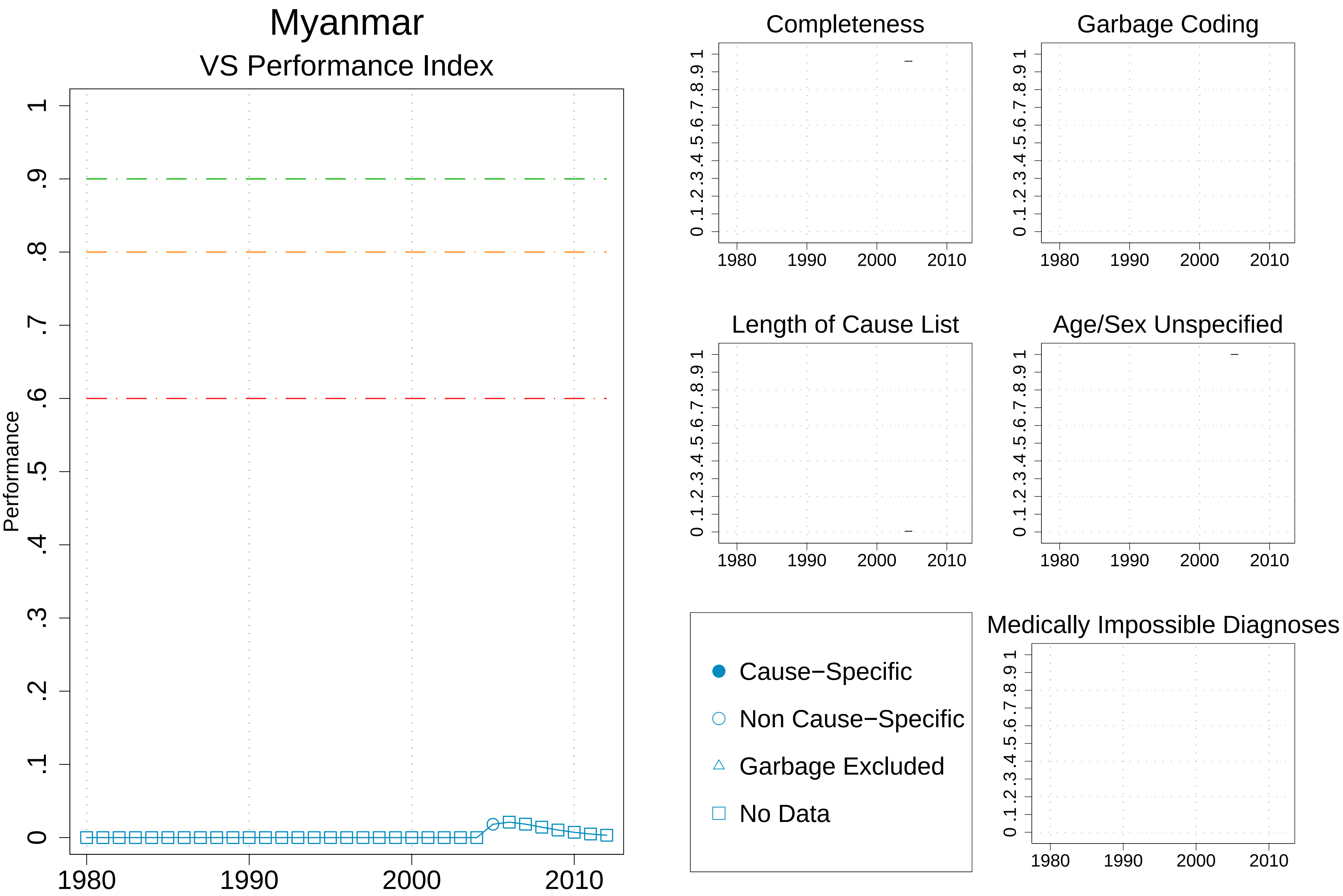

Indicators on their Original (Unweighted) Scale
and Subtracted from One Where Necessary so Higher Scores are Preferable to Lower

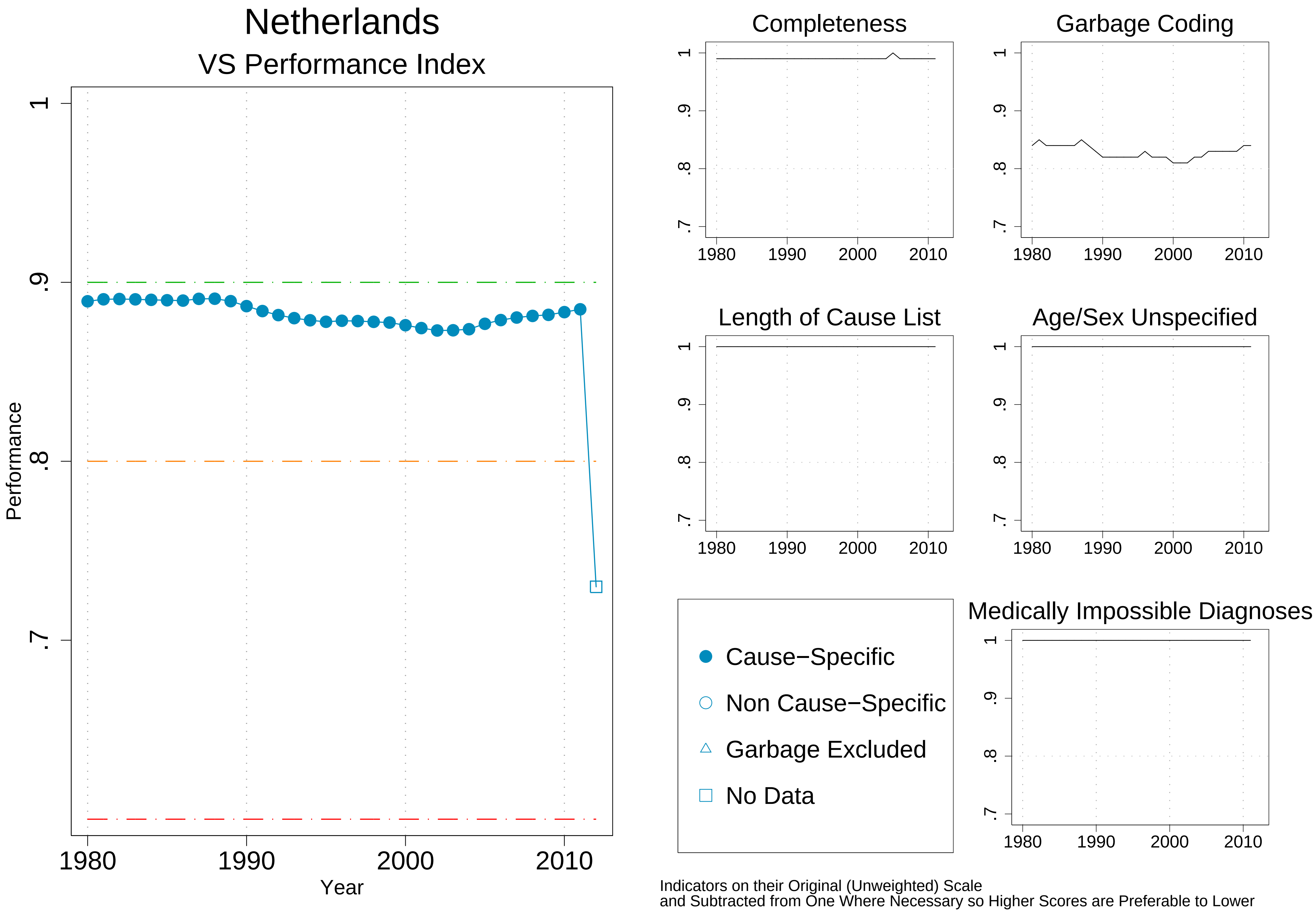
Netherlands
VS Performance Index
Performance
Year
1980
1990
2000
2010
1
.9
.8
.7
Completeness
Garbage Coding
Length of Cause List
Age/Sex Unspecified
Medically Impossible Diagnoses
Cause−Specific
Non Cause−Specific
Garbage Excluded
No Data
Indicators on their Original (Unweighted) Scale
and Subtracted from One Where Necessary so Higher Scores are Preferable to Lower

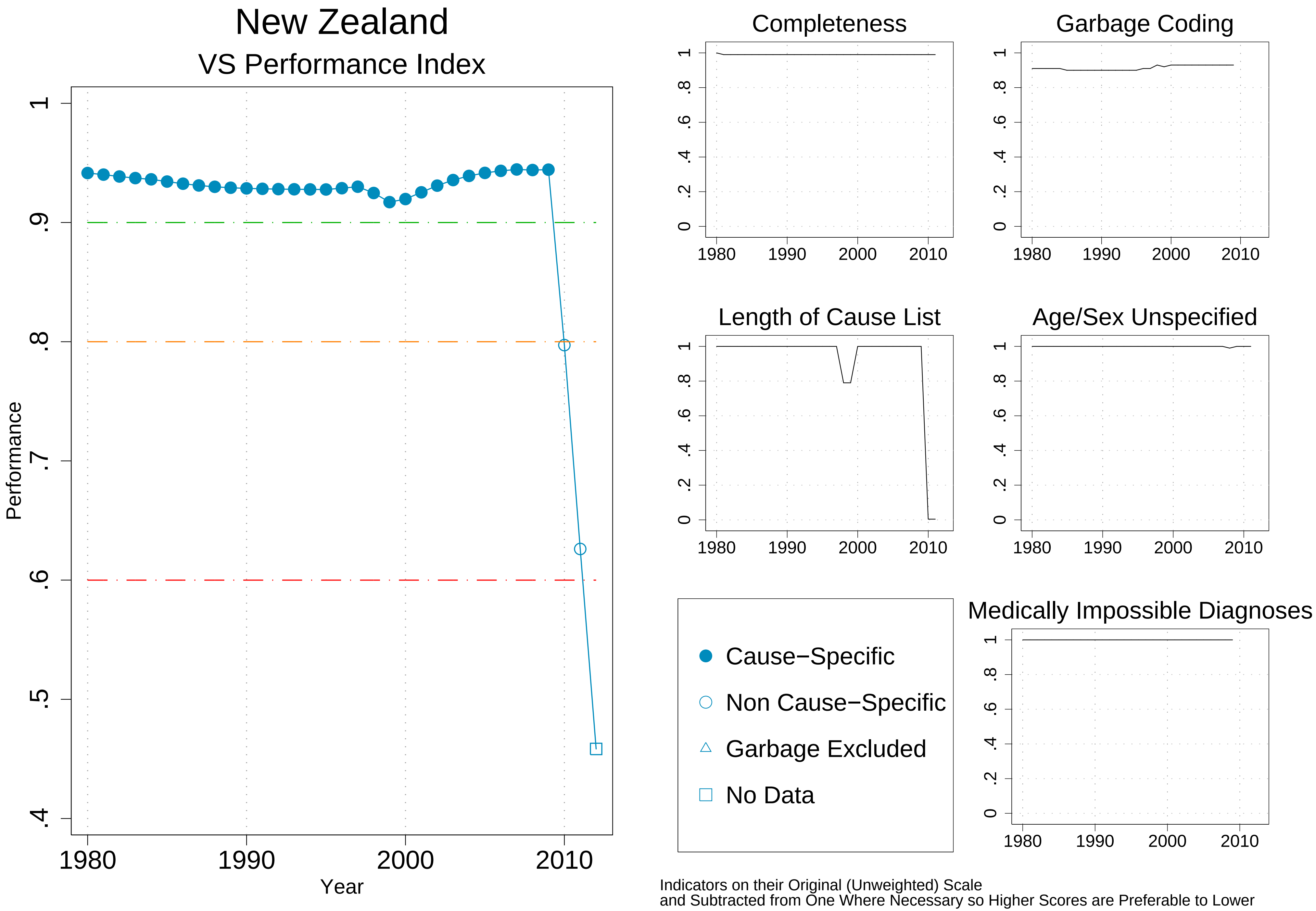

Indicators on their Original (Unweighted) Scale
and Subtracted from One Where Necessary so Higher Scores are Preferable to Lower

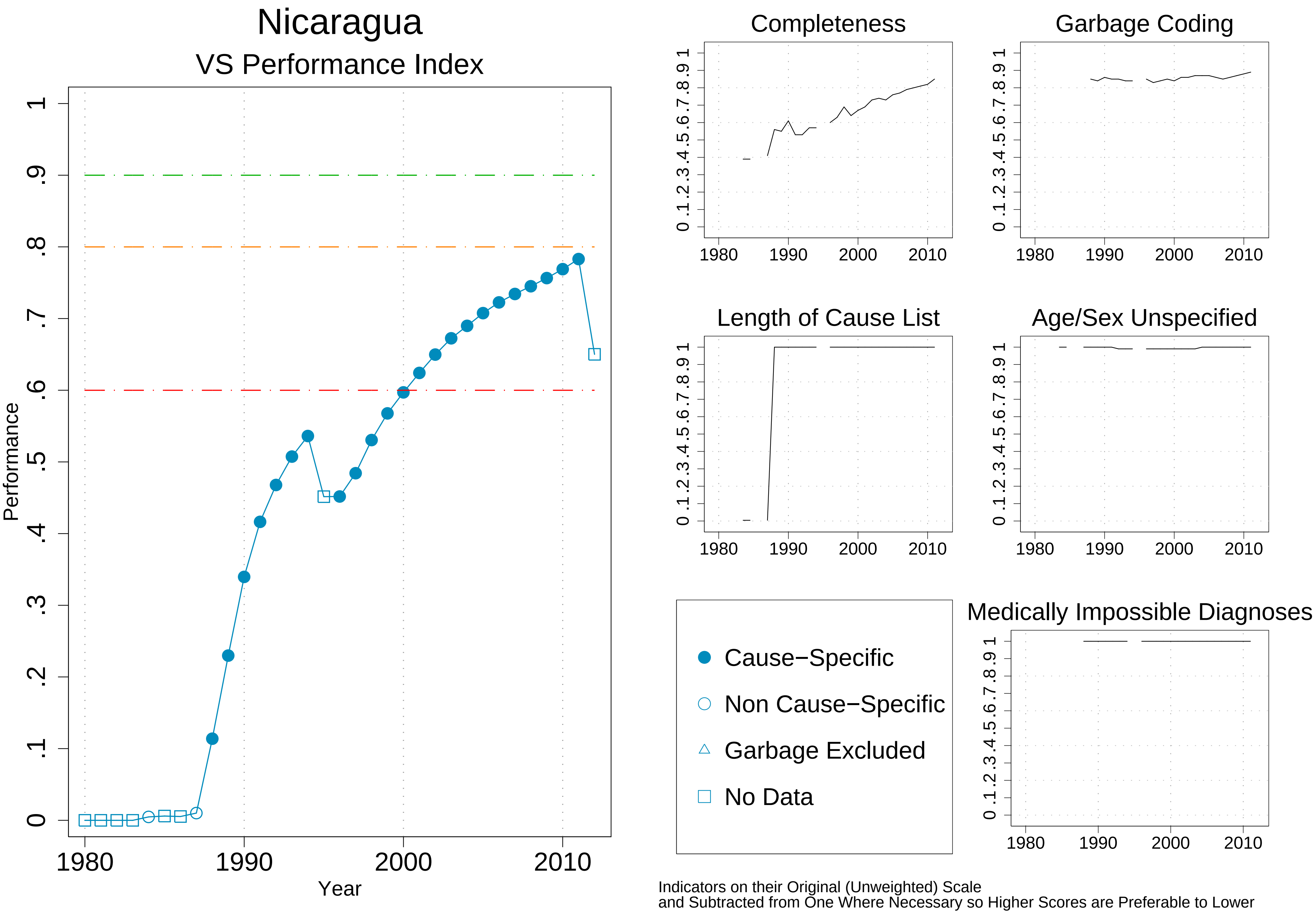

Indicators on their Original (Unweighted) Scale
and Subtracted from One Where Necessary so Higher Scores are Preferable to Lower

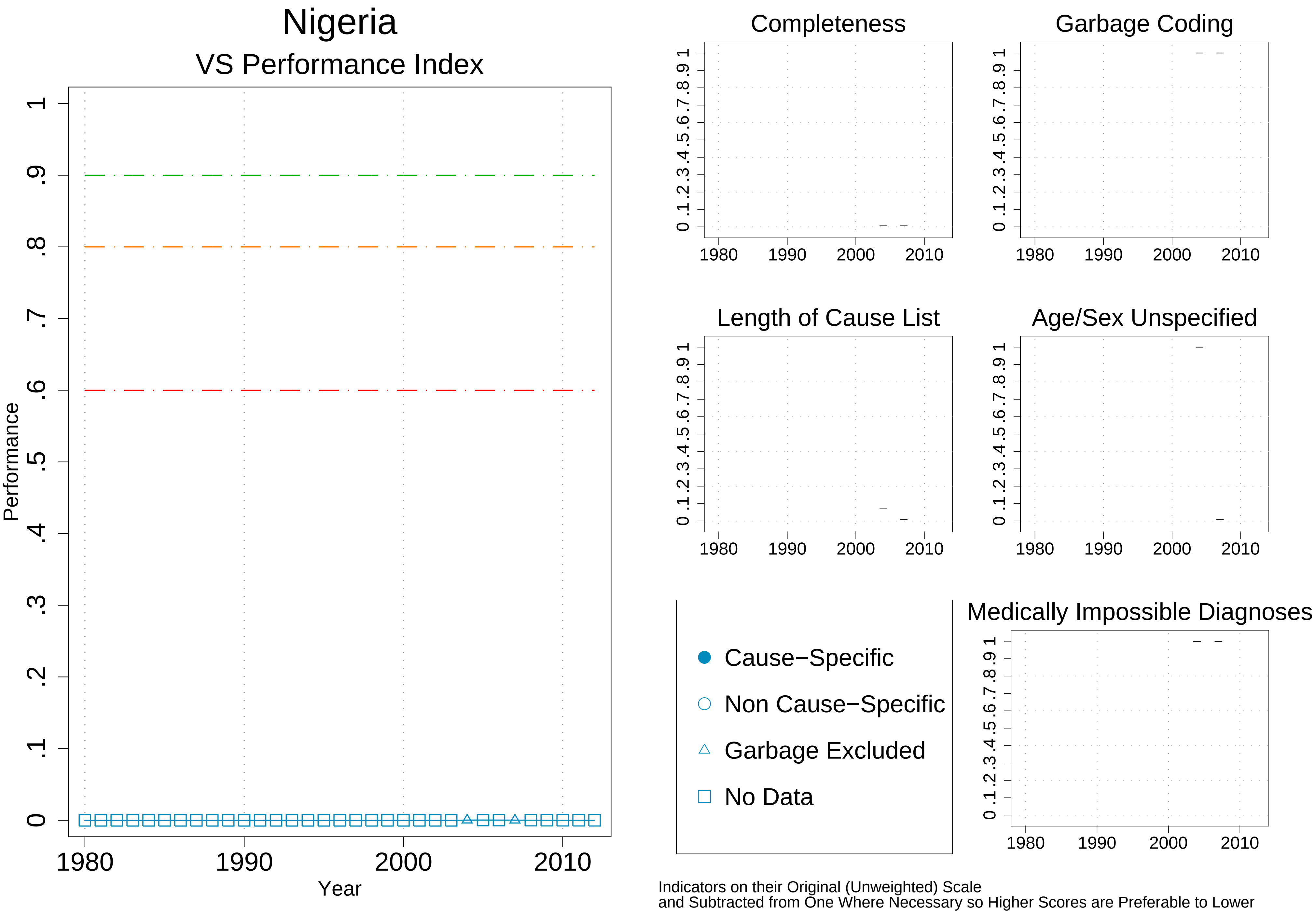

Indicators on their Original (Unweighted) Scale
and Subtracted from One Where Necessary so Higher Scores are Preferable to Lower

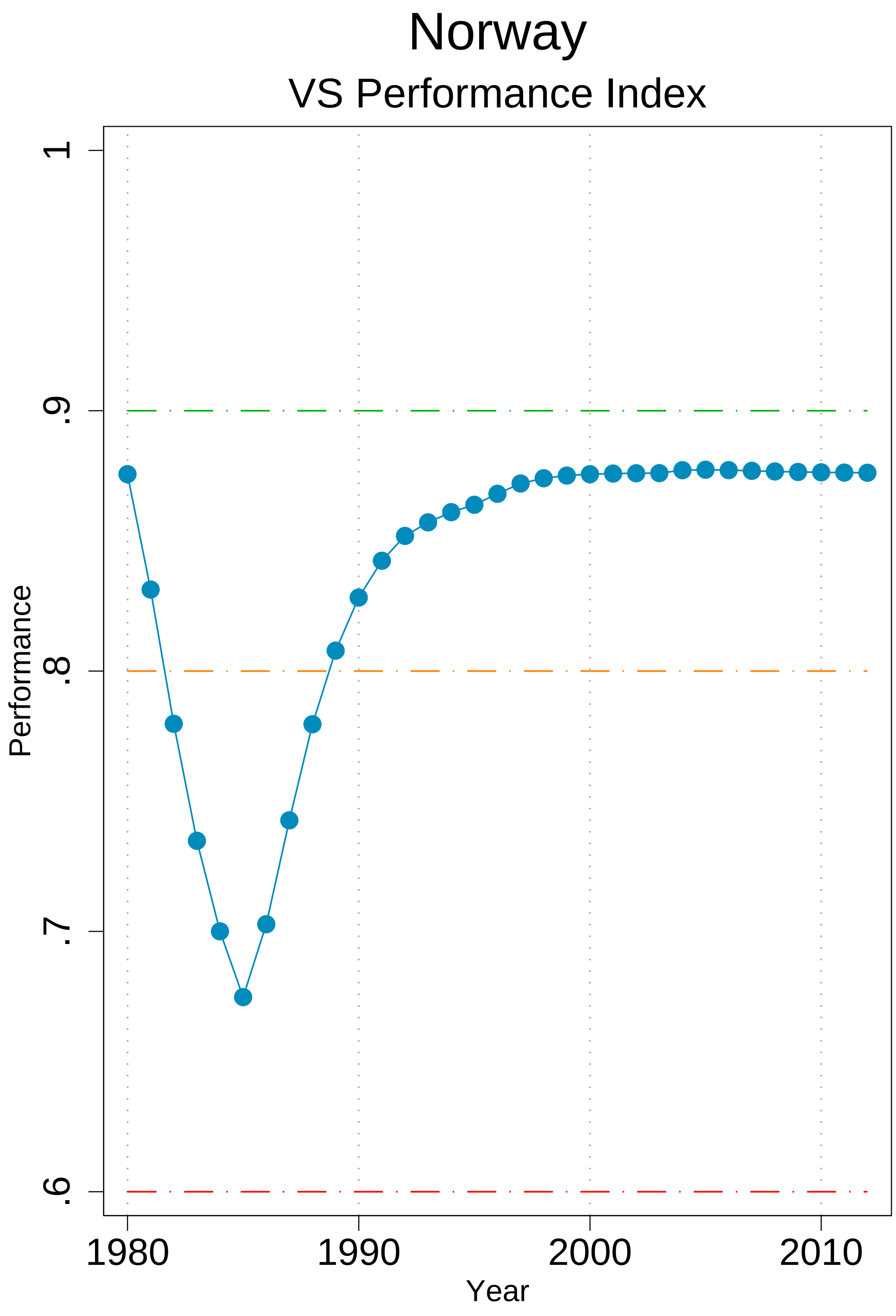

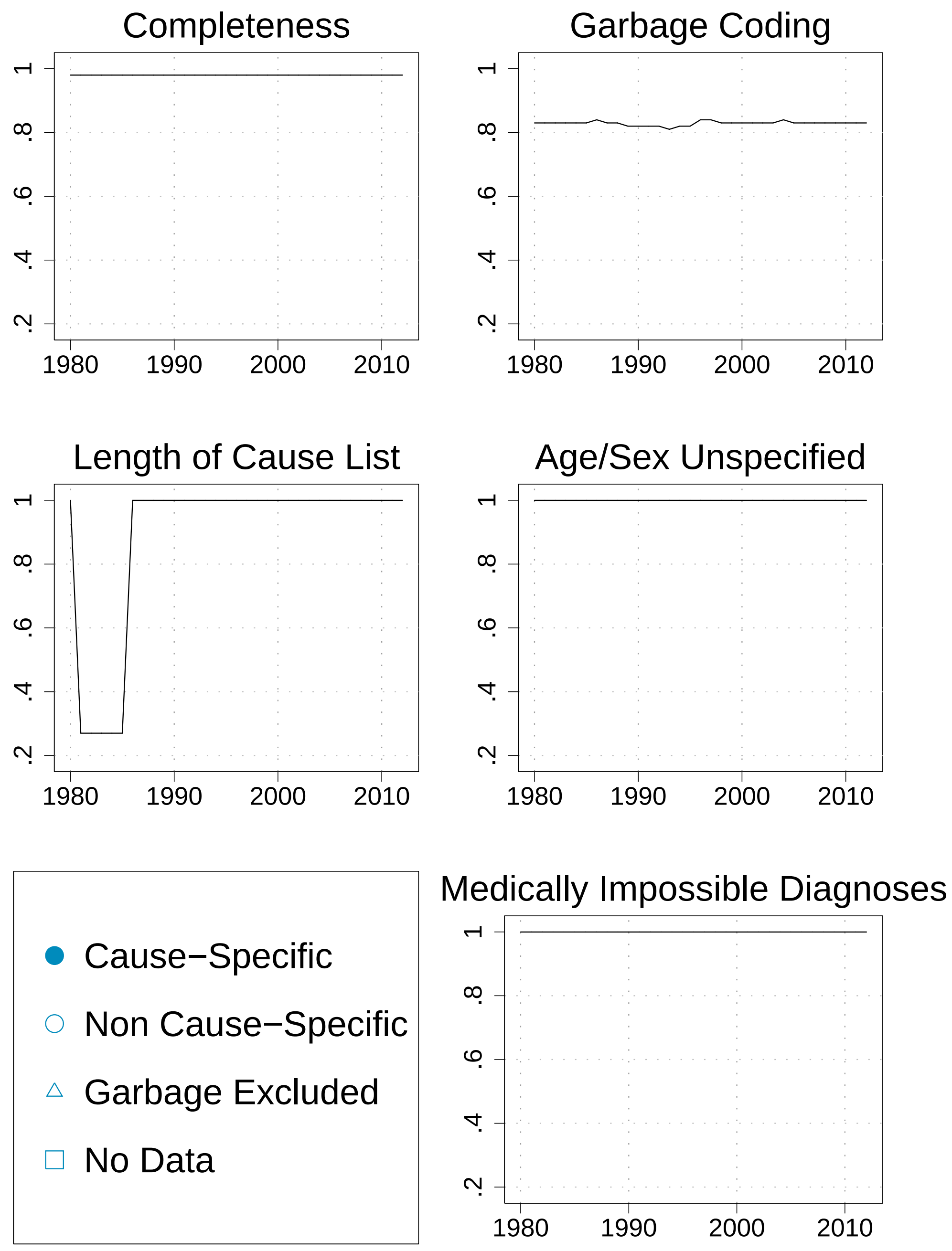

Indicators on their Original (Unweighted) Scale
and Subtracted from One Where Necessary so Higher Scores are Preferable to Lower

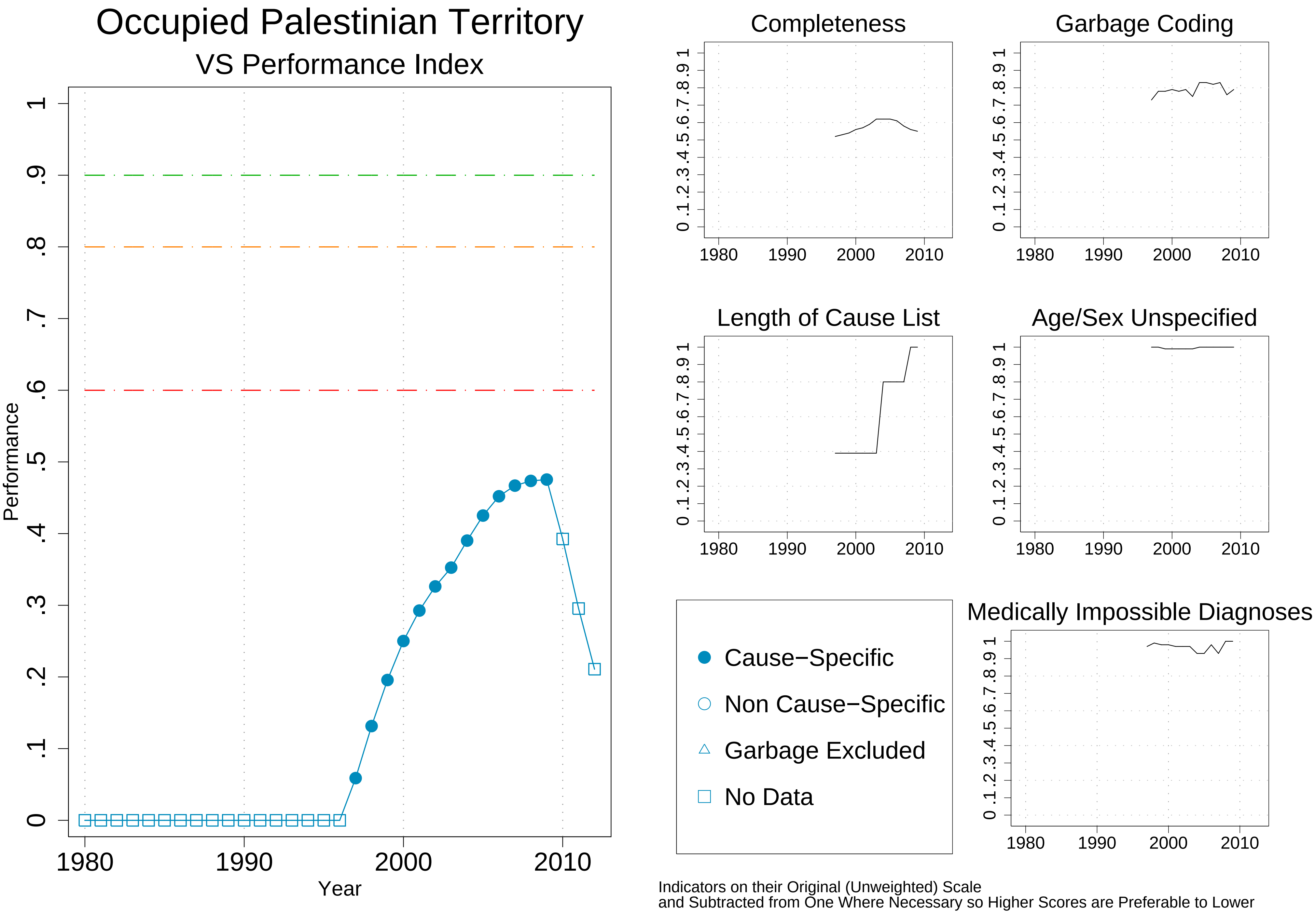
Occupied Palestinian Territory
VS Performance Index
Performance
Year
Completeness
Garbage Coding
Length of Cause List
Age/Sex Unspecified
Medically Impossible Diagnoses
Cause−Specific
Non Cause−Specific
Garbage Excluded
No Data
Indicators on their Original (Unweighted) Scale
and Subtracted from One Where Necessary so Higher Scores are Preferable to Lower

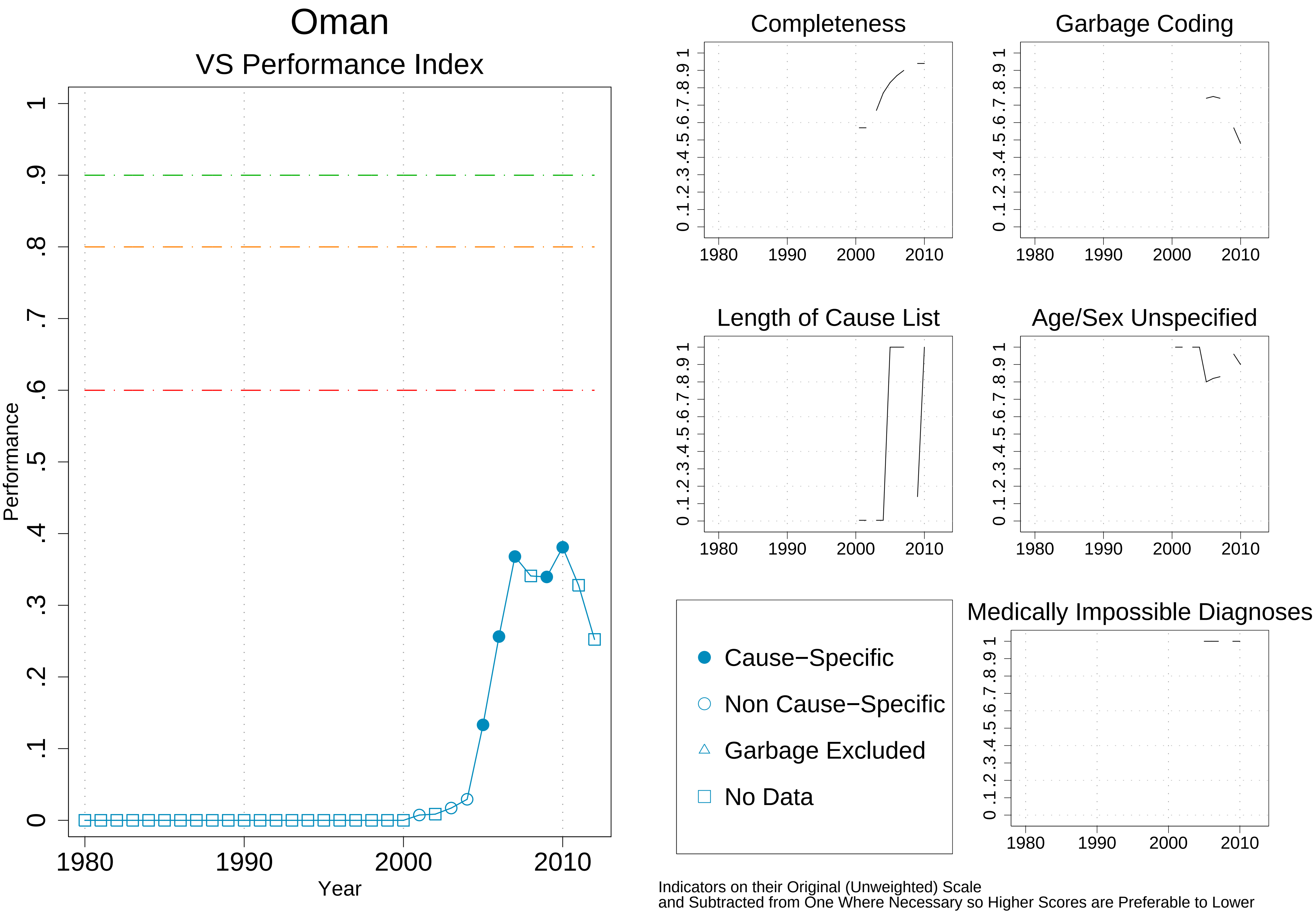

Indicators on their Original (Unweighted) Scale
and Subtracted from One Where Necessary so Higher Scores are Preferable to Lower

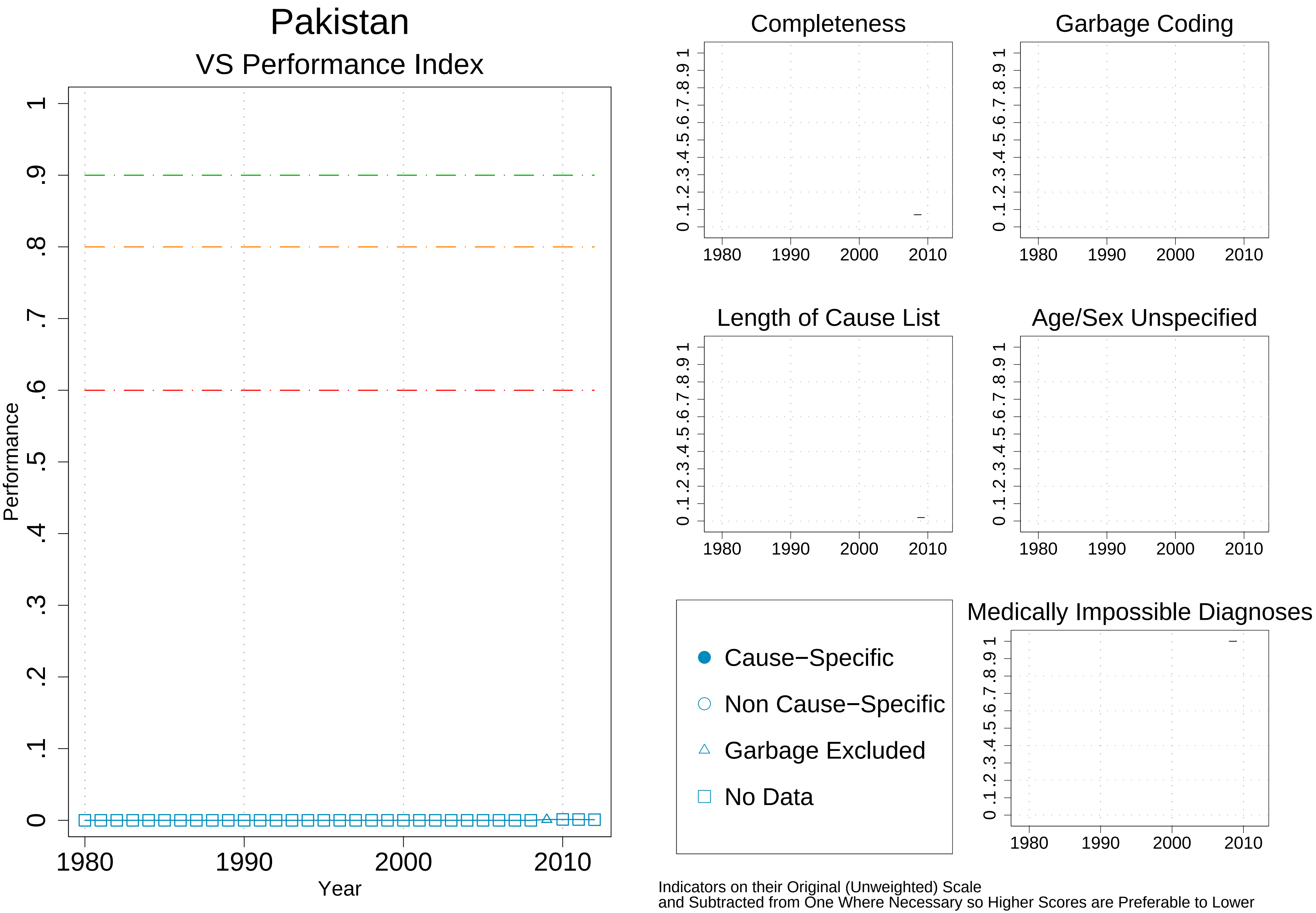

Indicators on their Original (Unweighted) Scale
and Subtracted from One Where Necessary so Higher Scores are Preferable to Lower

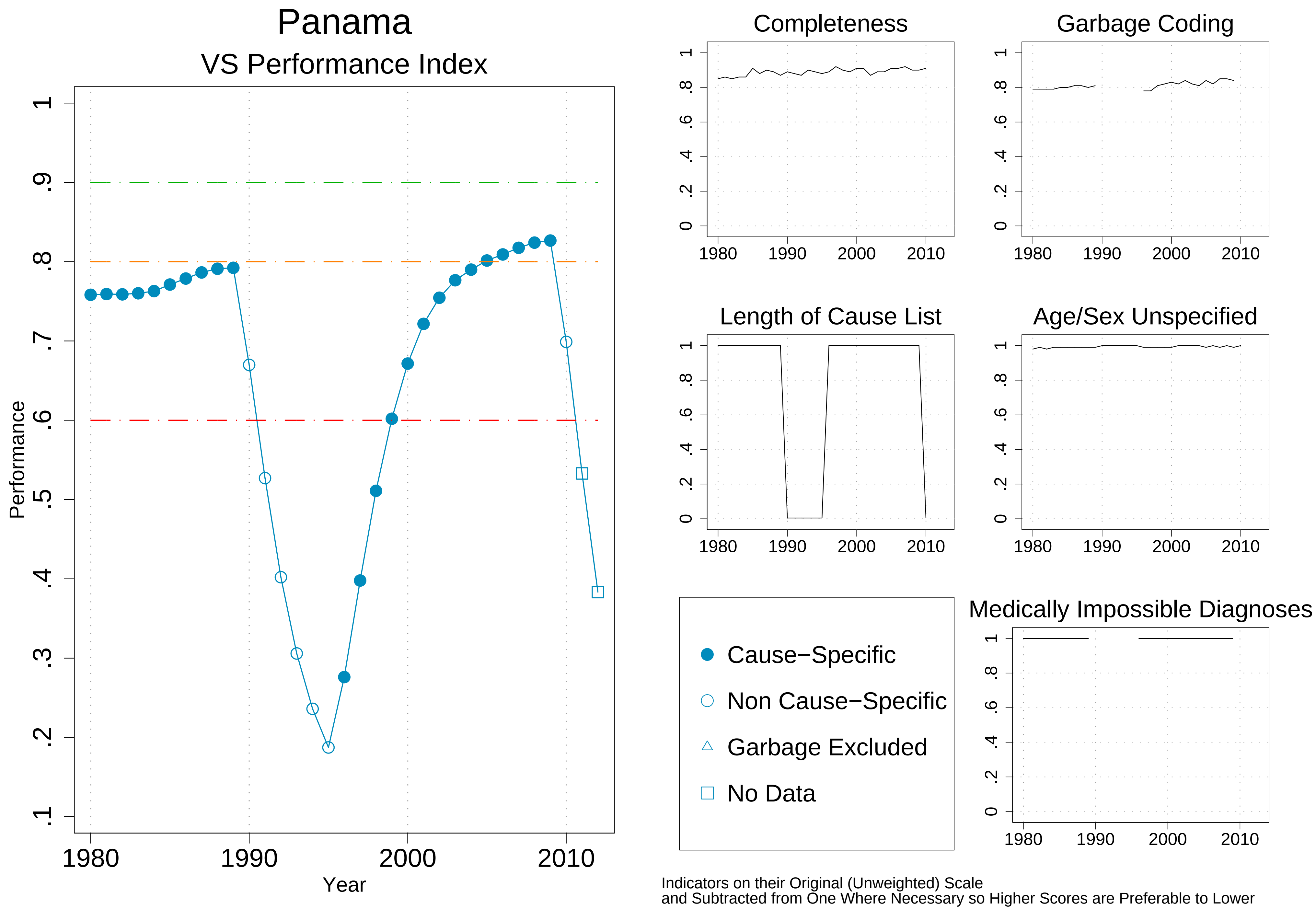
Panama
VS Performance Index
Performance
Year
1980
1990
2000
2010
.1
.2
.3
.4
.5
.6
.7
.8
.9
1
Completeness
Garbage Coding
Length of Cause List
Age/Sex Unspecified
Medically Impossible Diagnoses
0
.2
.4
.6
.8
1
Cause−Specific
Non Cause−Specific
Garbage Excluded
No Data
Indicators on their Original (Unweighted) Scale
and Subtracted from One Where Necessary so Higher Scores are Preferable to Lower

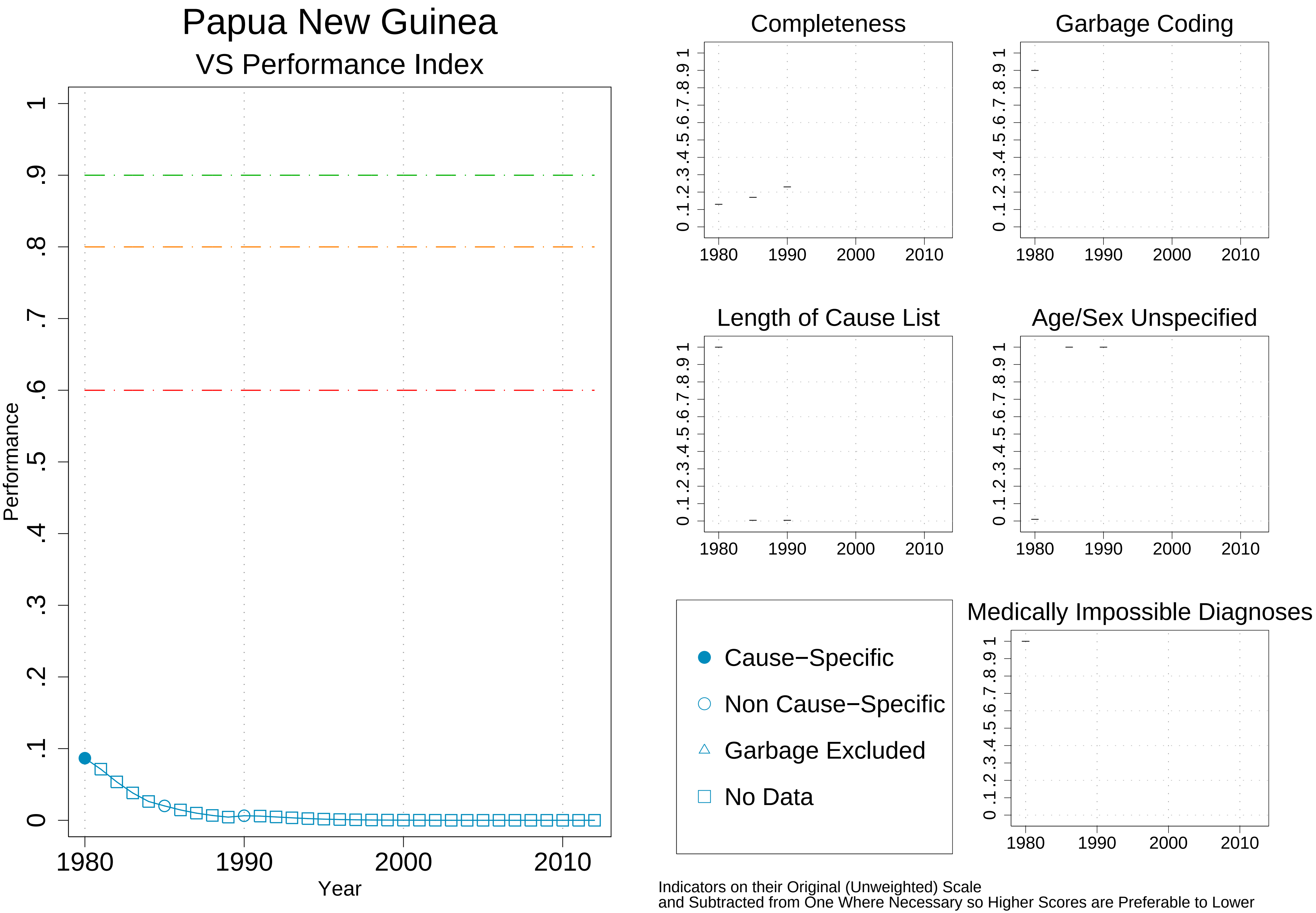

Indicators on their Original (Unweighted) Scale
and Subtracted from One Where Necessary so Higher Scores are Preferable to Lower

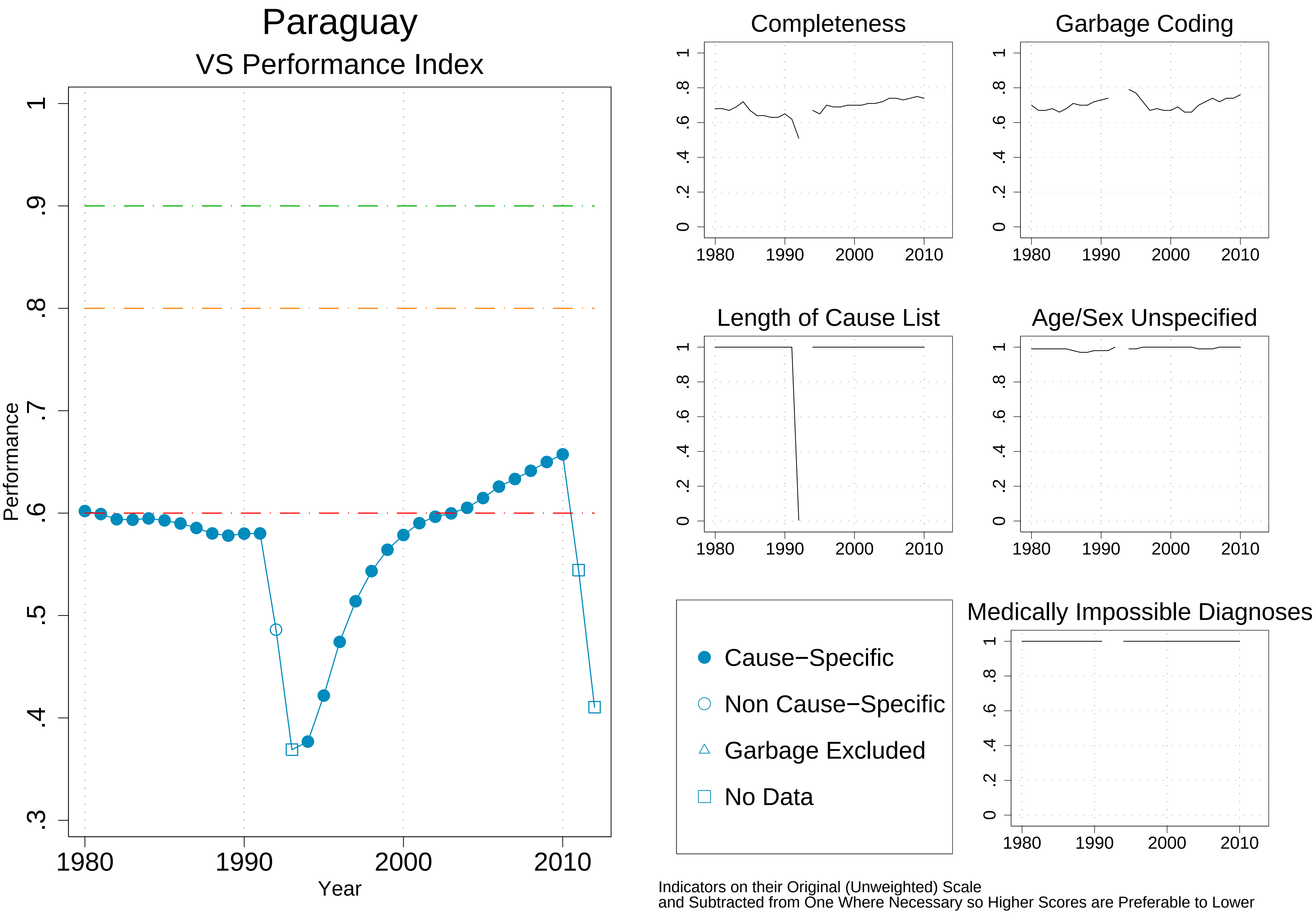

Indicators on their Original (Unweighted) Scale
and Subtracted from One Where Necessary so Higher Scores are Preferable to Lower

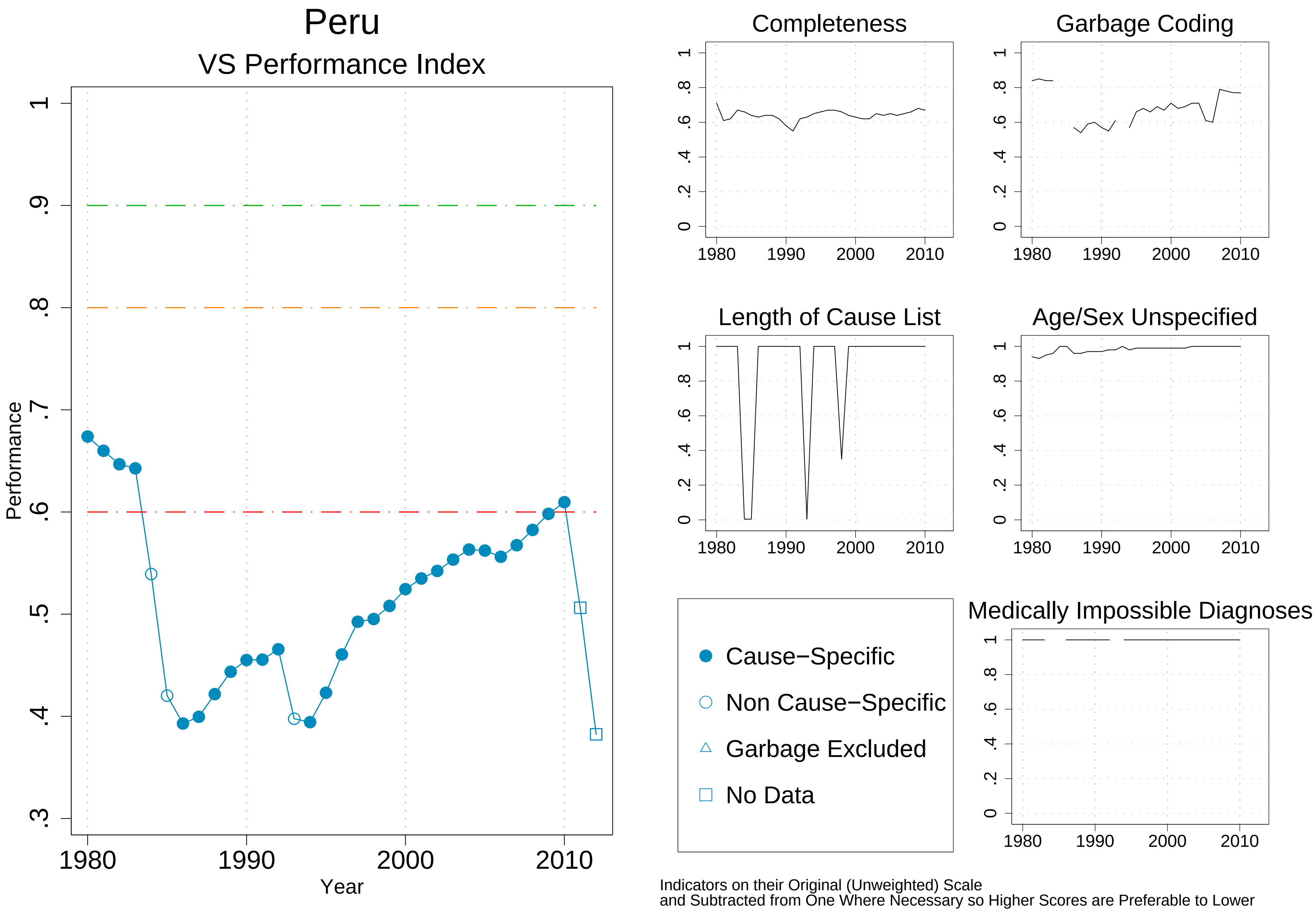

Indicators on their Original (Unweighted) Scale
and Subtracted from One Where Necessary so Higher Scores are Preferable to Lower

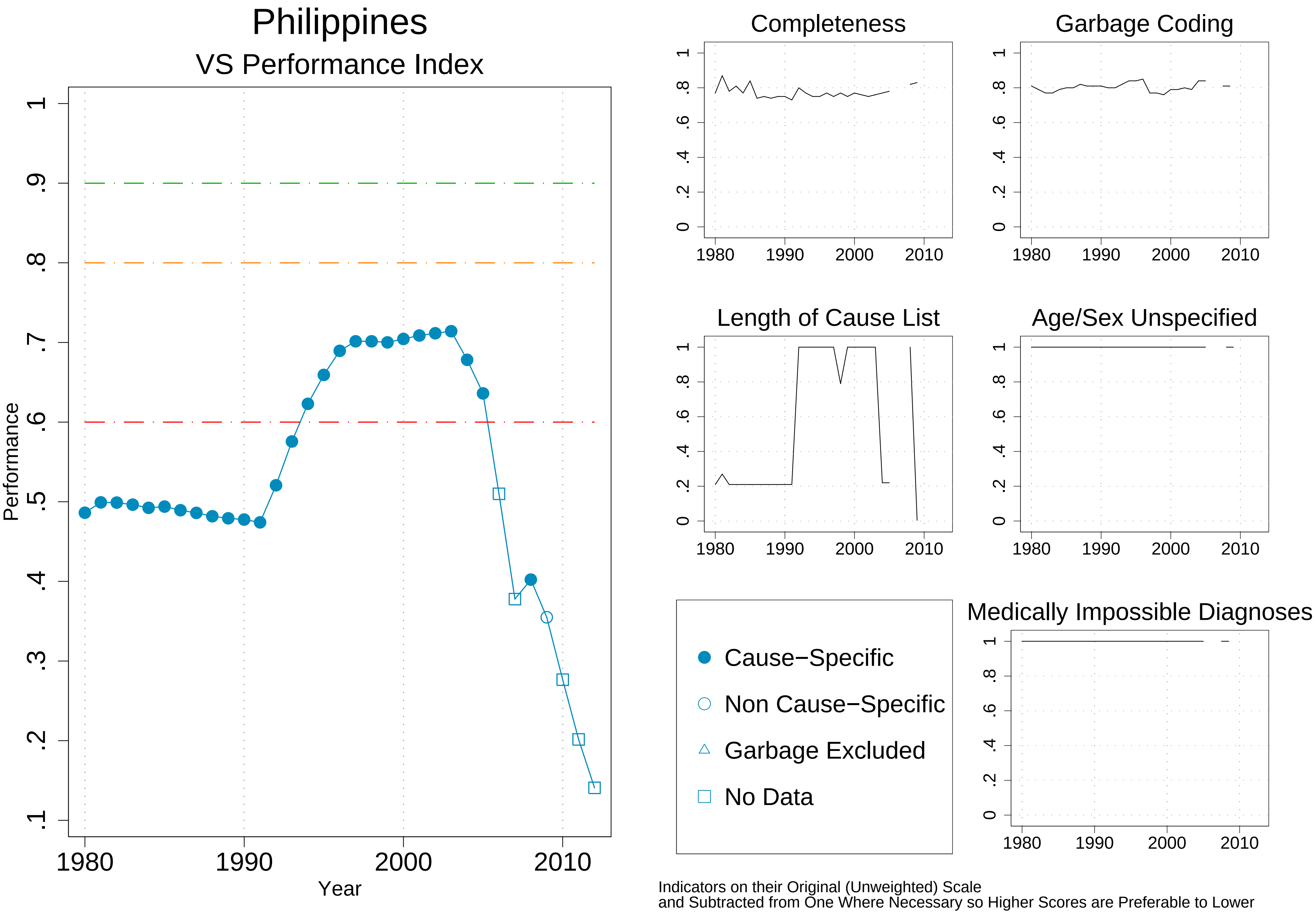

Indicators on their Original (Unweighted) Scale
and Subtracted from One Where Necessary so Higher Scores are Preferable to Lower

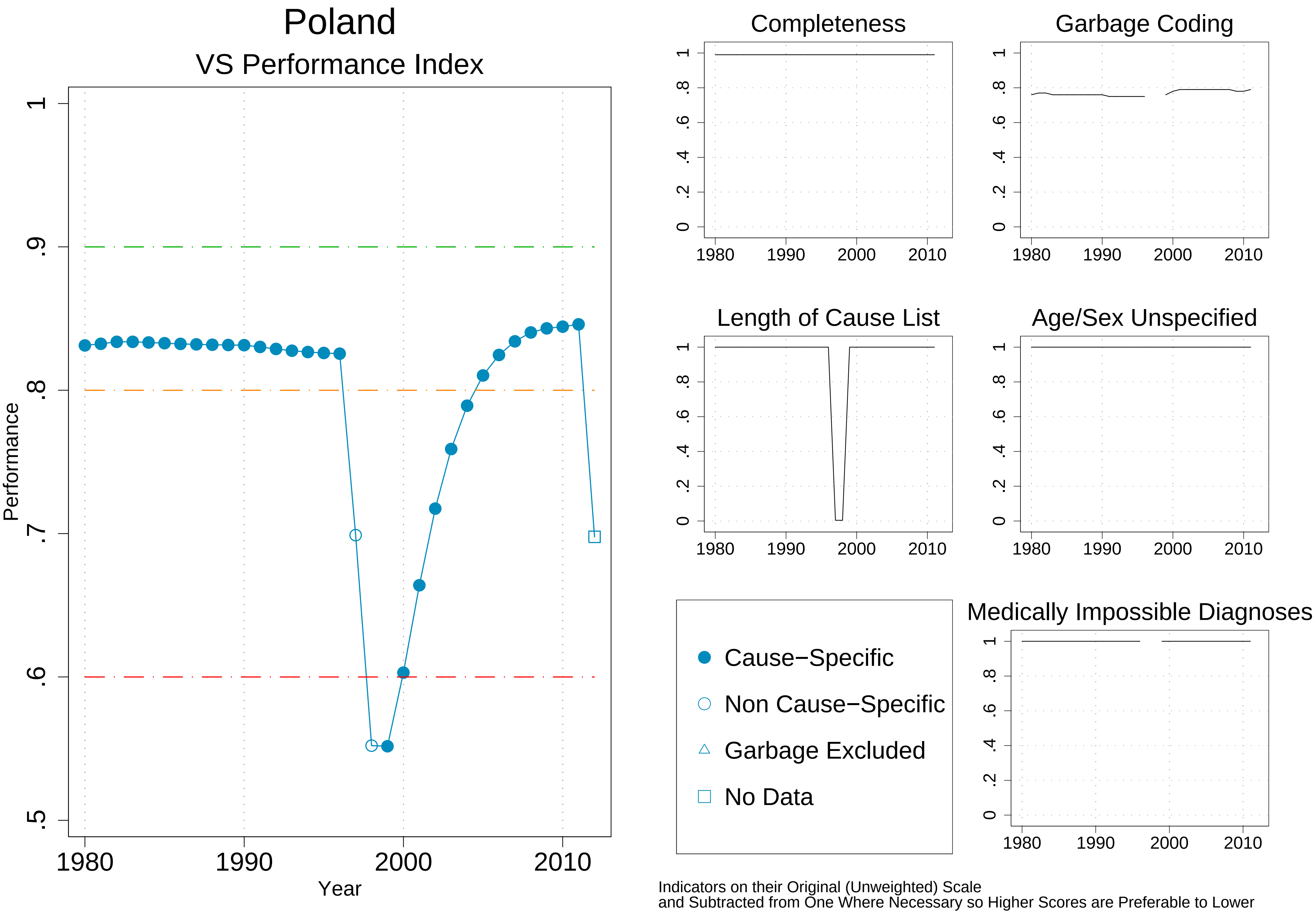

Indicators on their Original (Unweighted) Scale
and Subtracted from One Where Necessary so Higher Scores are Preferable to Lower

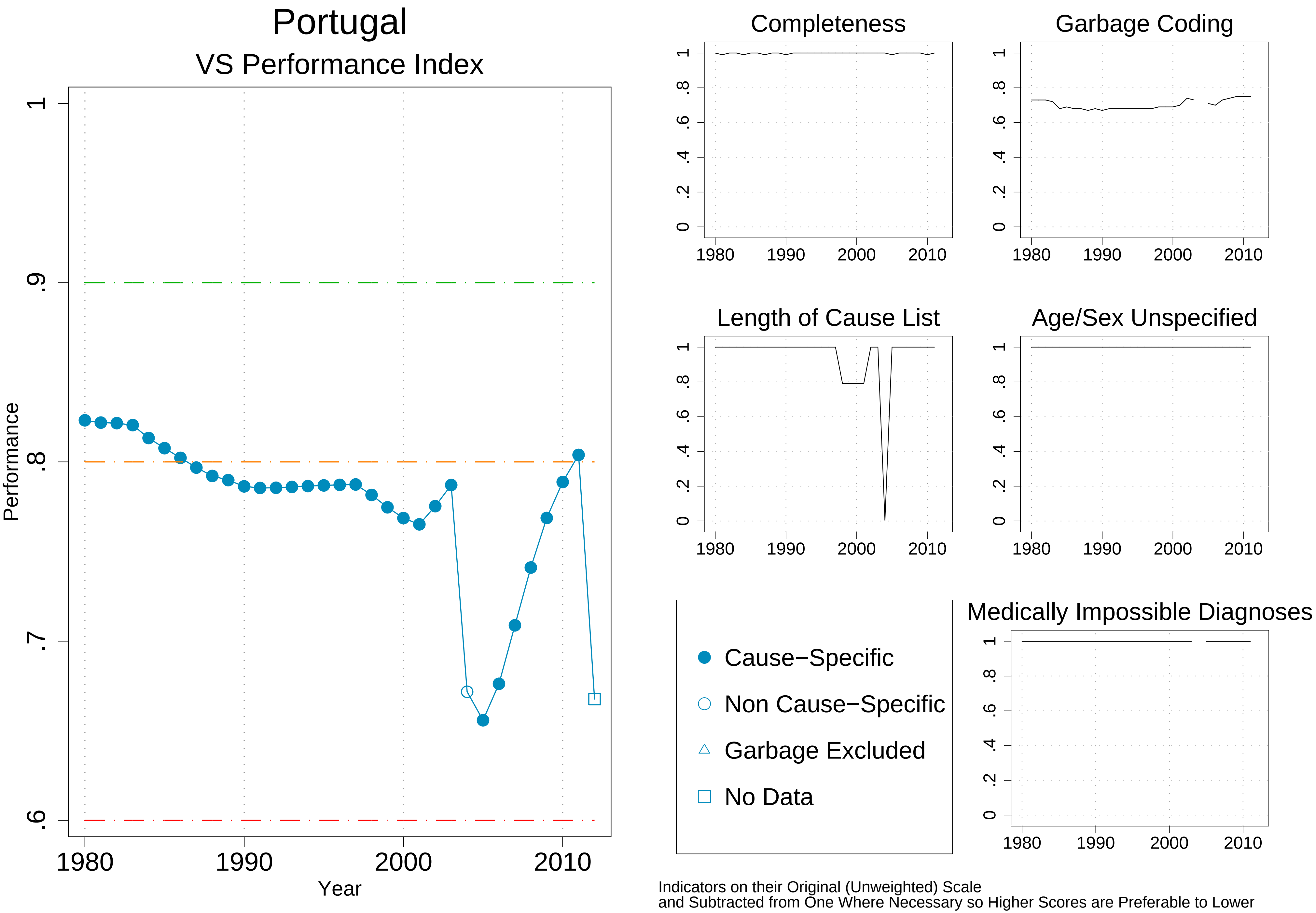

Indicators on their Original (Unweighted) Scale
and Subtracted from One Where Necessary so Higher Scores are Preferable to Lower

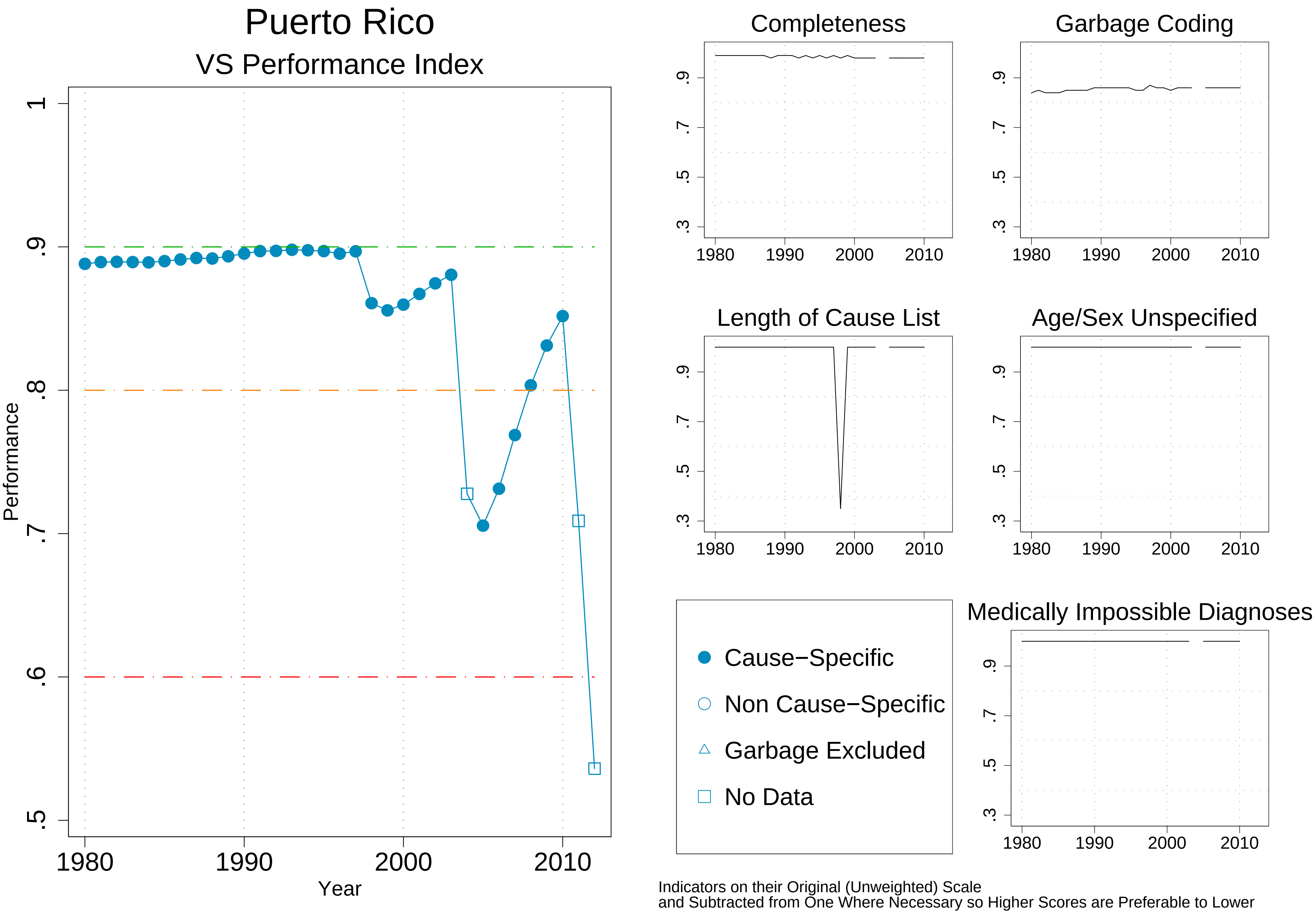

Indicators on their Original (Unweighted) Scale
and Subtracted from One Where Necessary so Higher Scores are Preferable to Lower

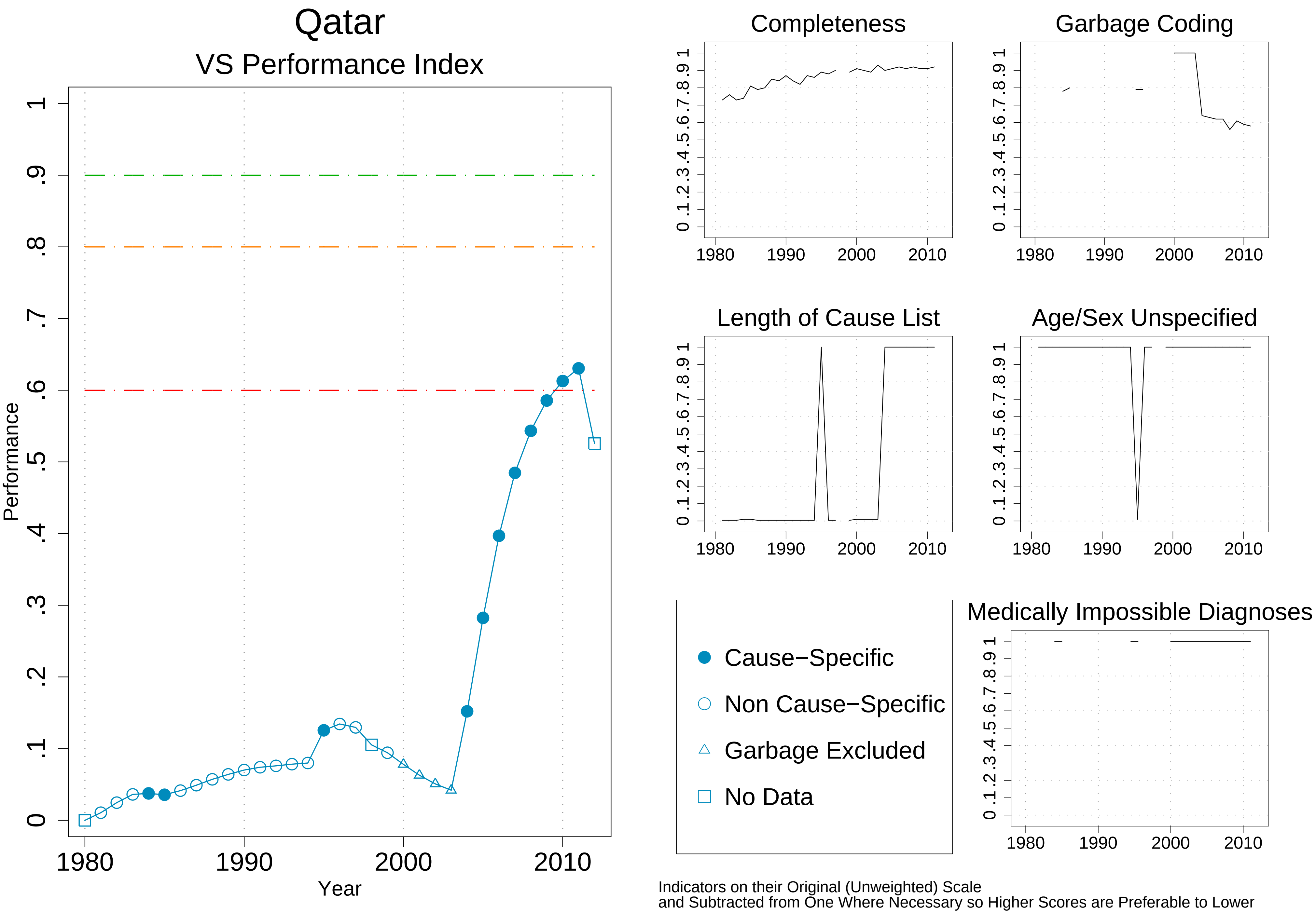

Qatar
VS Performance Index
Performance
Year
Completeness
Garbage Coding
Length of Cause List
Age/Sex Unspecified
Medically Impossible Diagnoses
Cause−Specific
Non Cause−Specific
Garbage Excluded
No Data
Indicators on their Original (Unweighted) Scale
and Subtracted from One Where Necessary so Higher Scores are Preferable to Lower

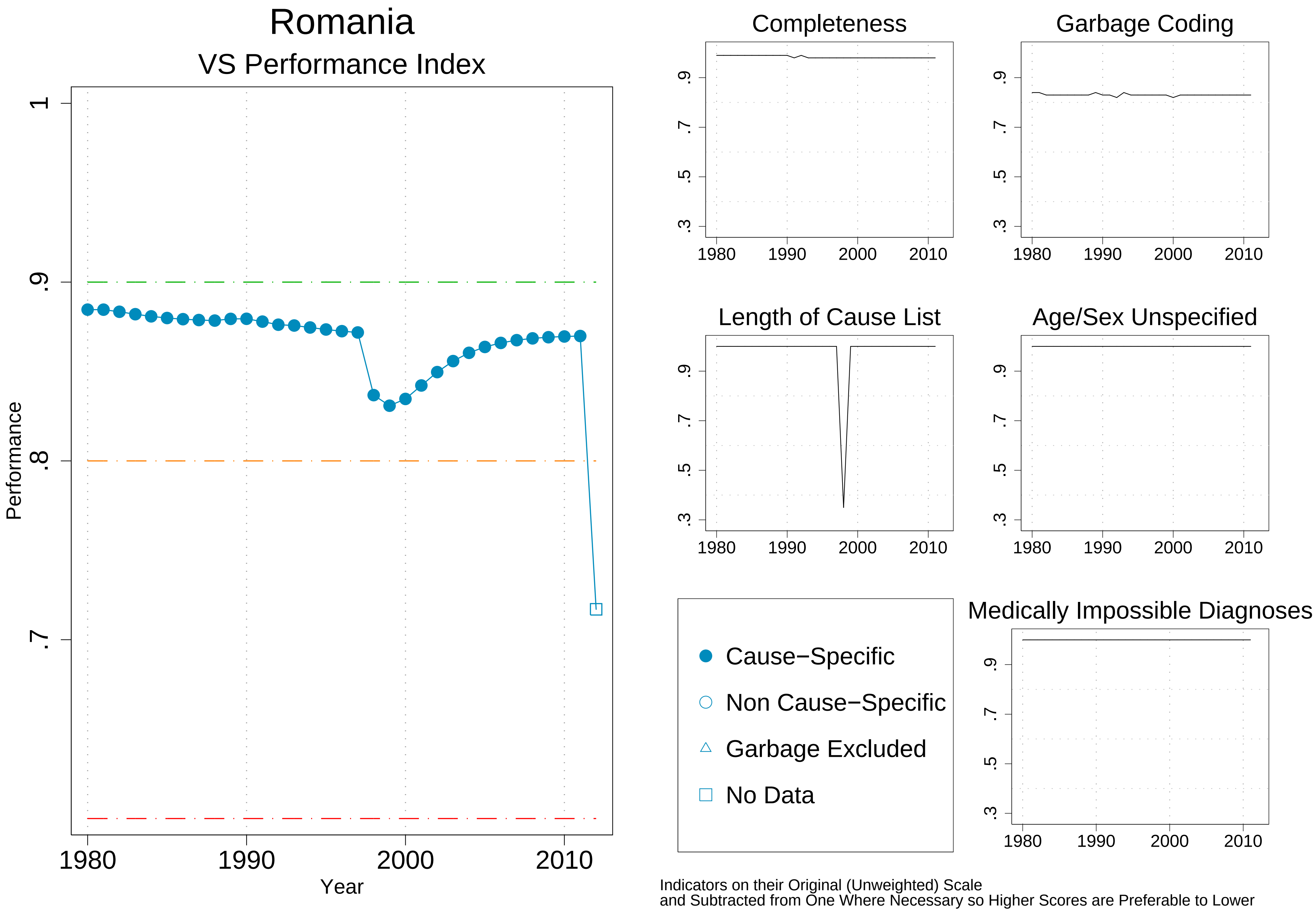

Indicators on their Original (Unweighted) Scale
and Subtracted from One Where Necessary so Higher Scores are Preferable to Lower

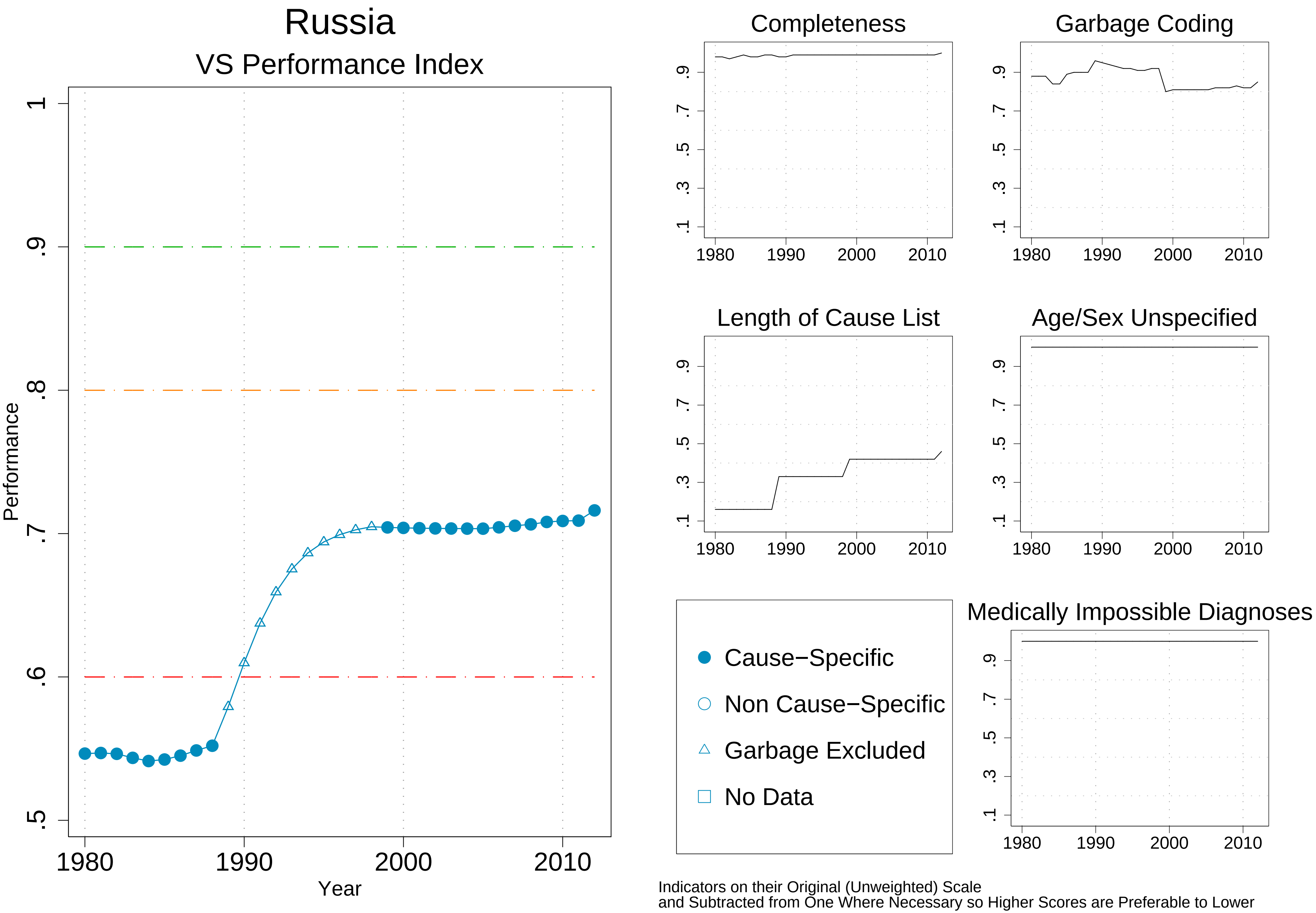

Indicators on their Original (Unweighted) Scale
and Subtracted from One Where Necessary so Higher Scores are Preferable to Lower

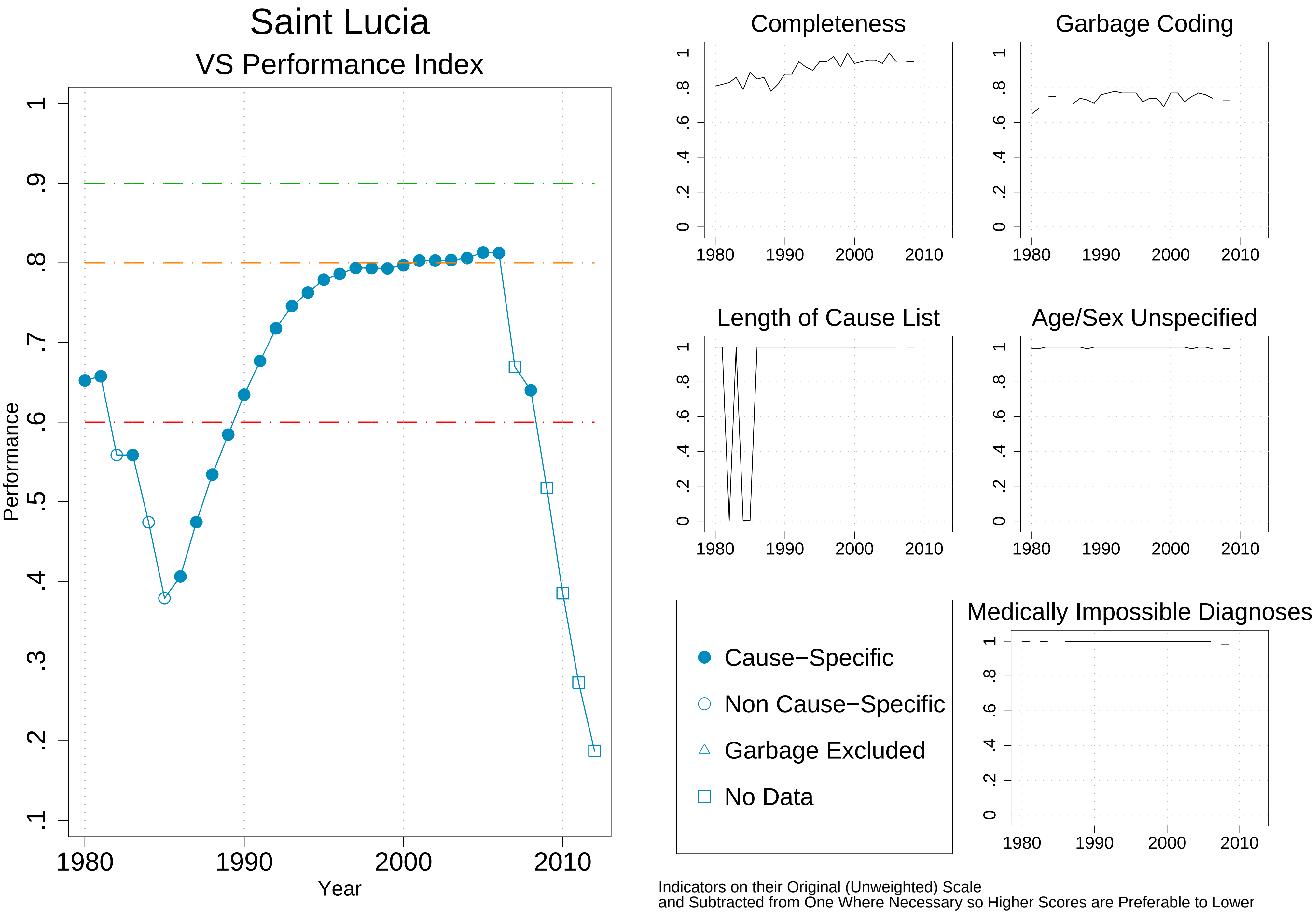

Indicators on their Original (Unweighted) Scale
and Subtracted from One Where Necessary so Higher Scores are Preferable to Lower

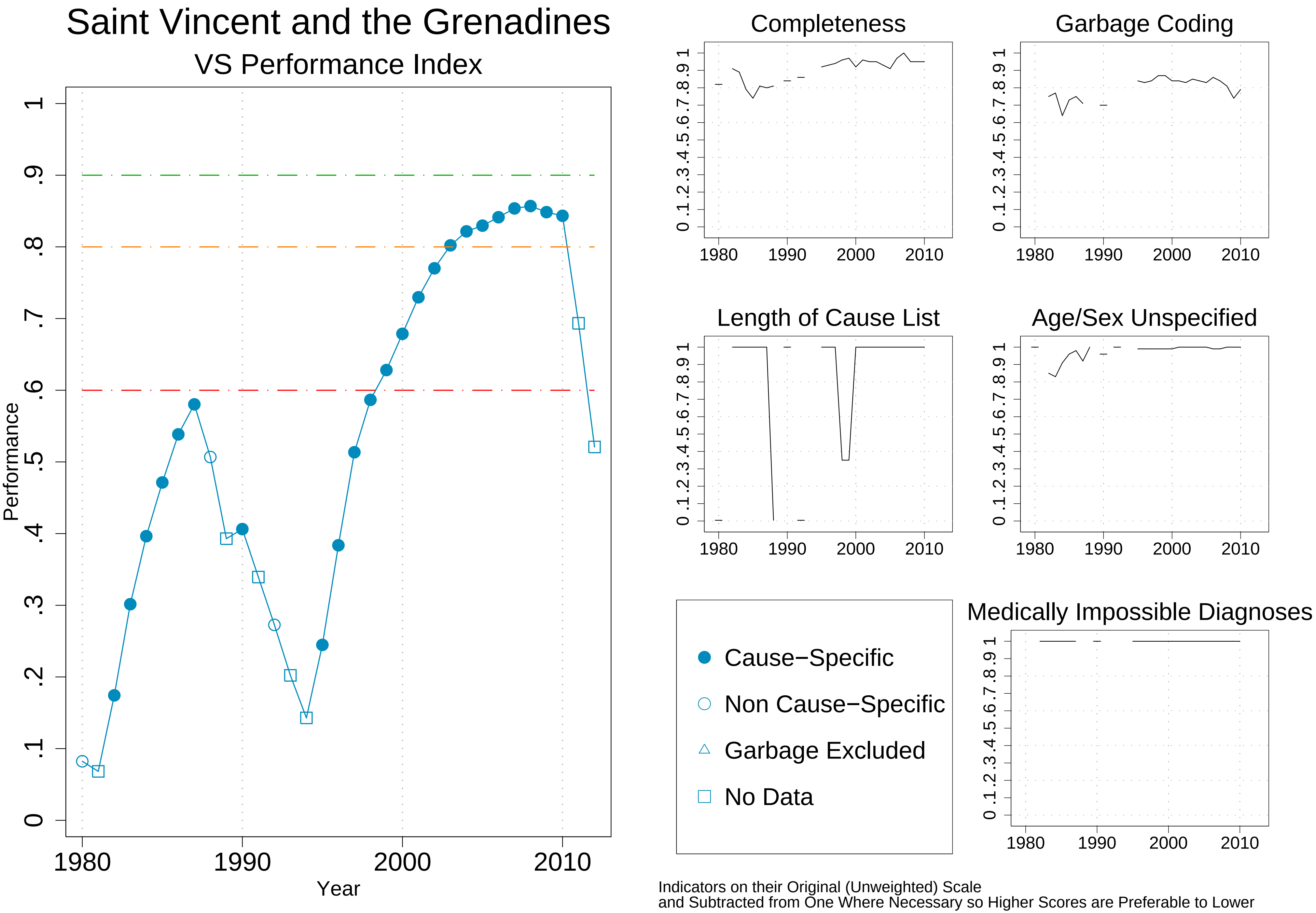

Indicators on their Original (Unweighted) Scale
and Subtracted from One Where Necessary so Higher Scores are Preferable to Lower

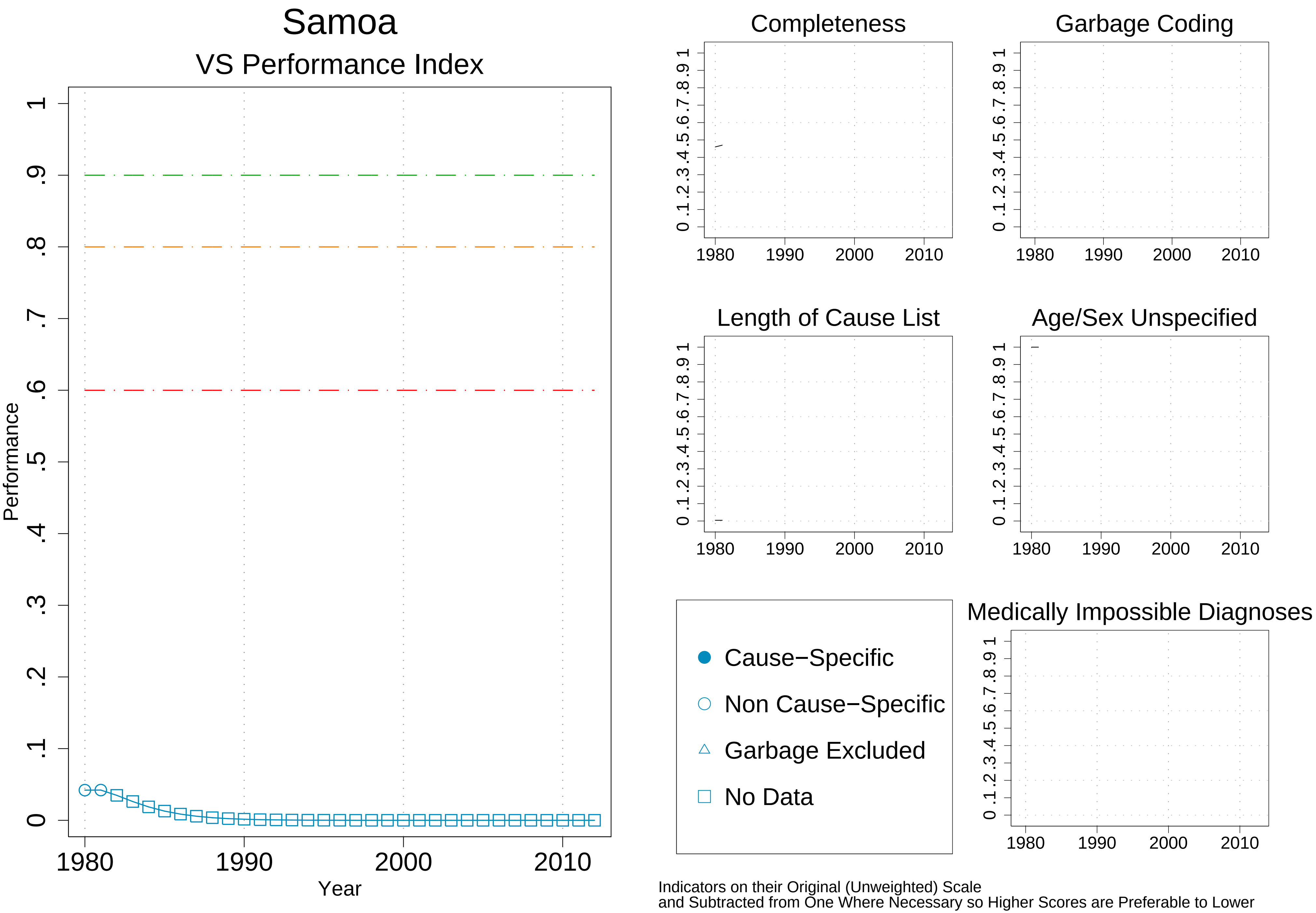

Indicators on their Original (Unweighted) Scale
and Subtracted from One Where Necessary so Higher Scores are Preferable to Lower

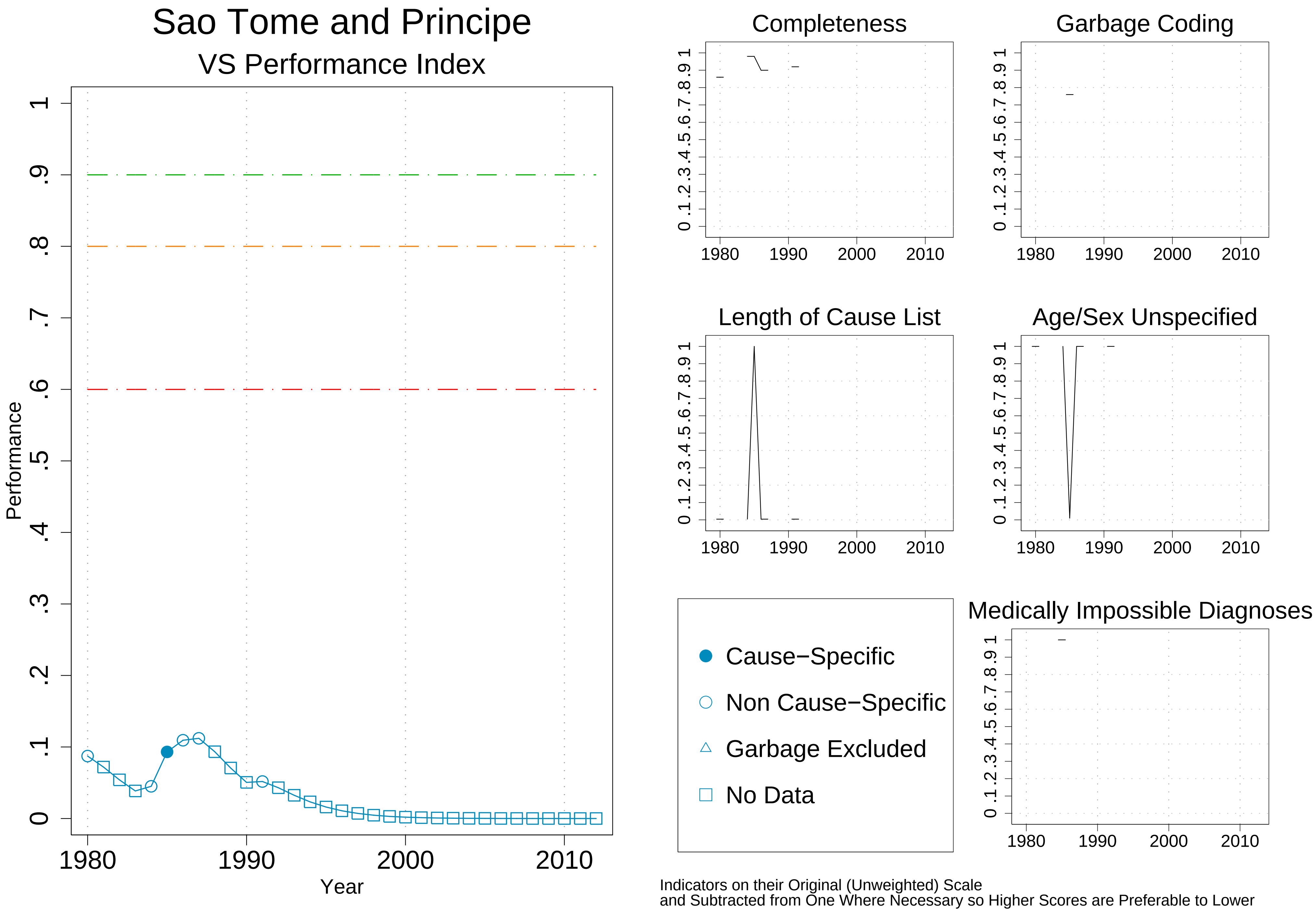

Indicators on their Original (Unweighted) Scale
and Subtracted from One Where Necessary so Higher Scores are Preferable to Lower

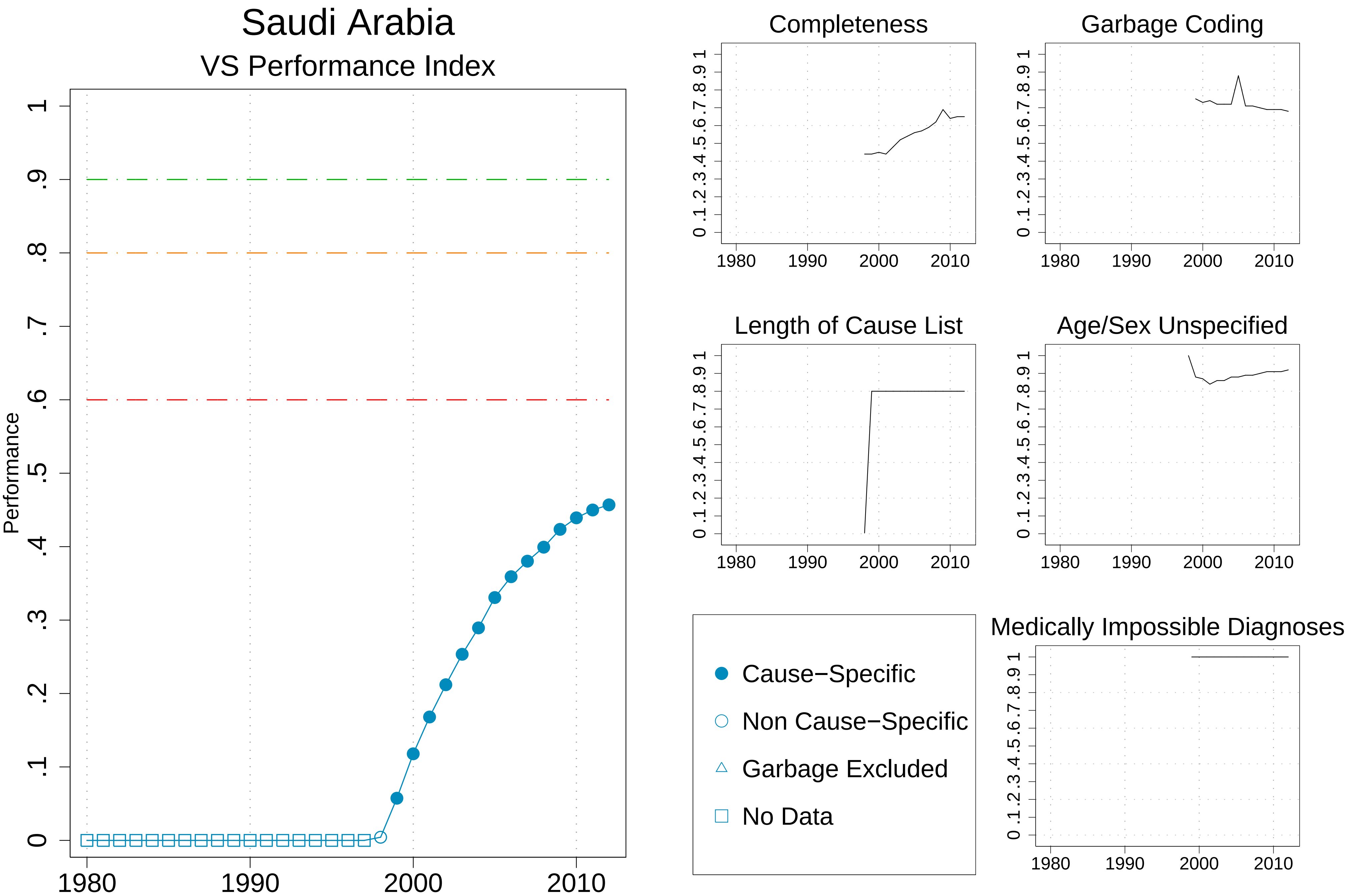

Indicators on their Original (Unweighted) Scale
and Subtracted from One Where Necessary so Higher Scores are Preferable to Lower

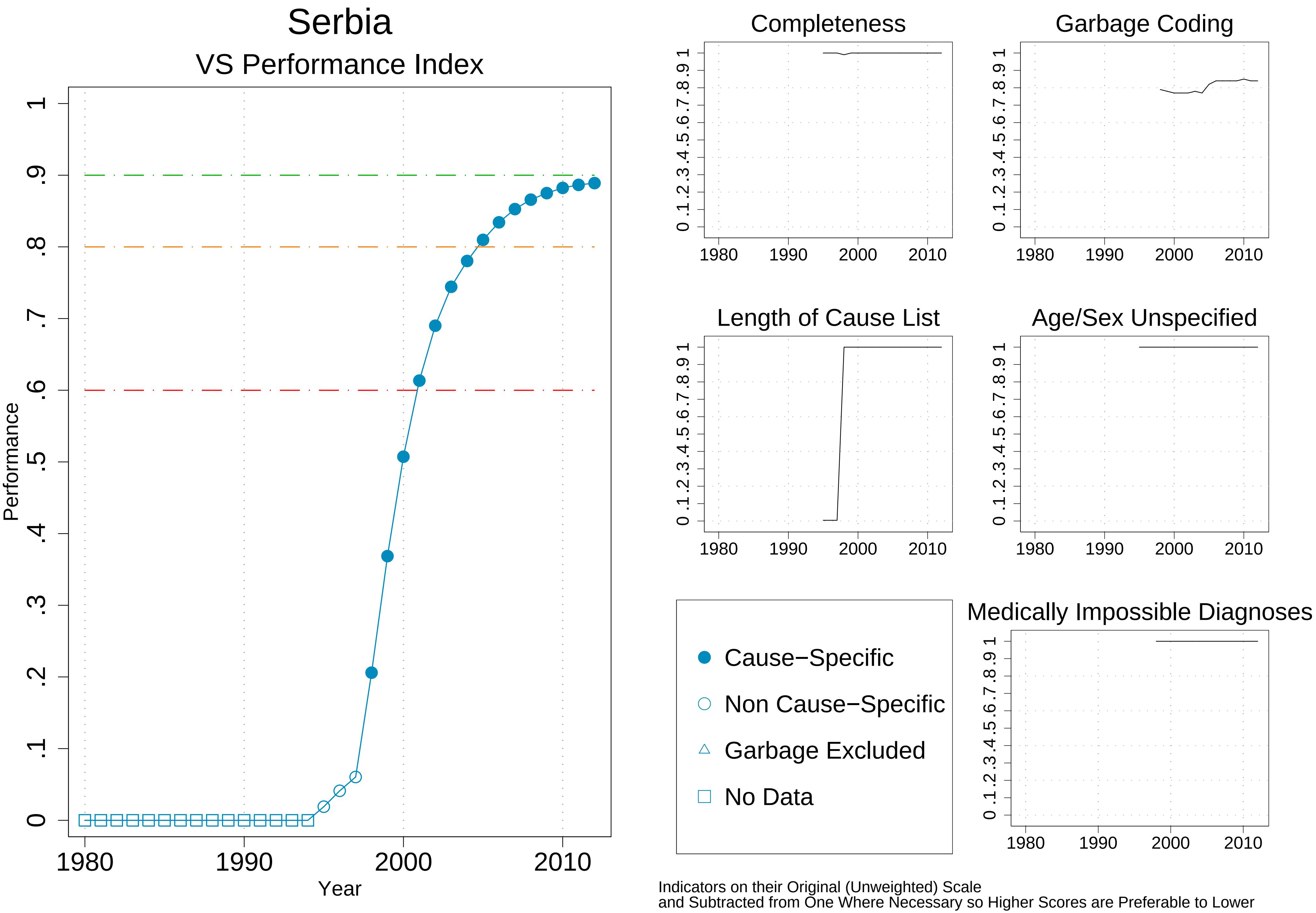

Indicators on their Original (Unweighted) Scale
and Subtracted from One Where Necessary so Higher Scores are Preferable to Lower

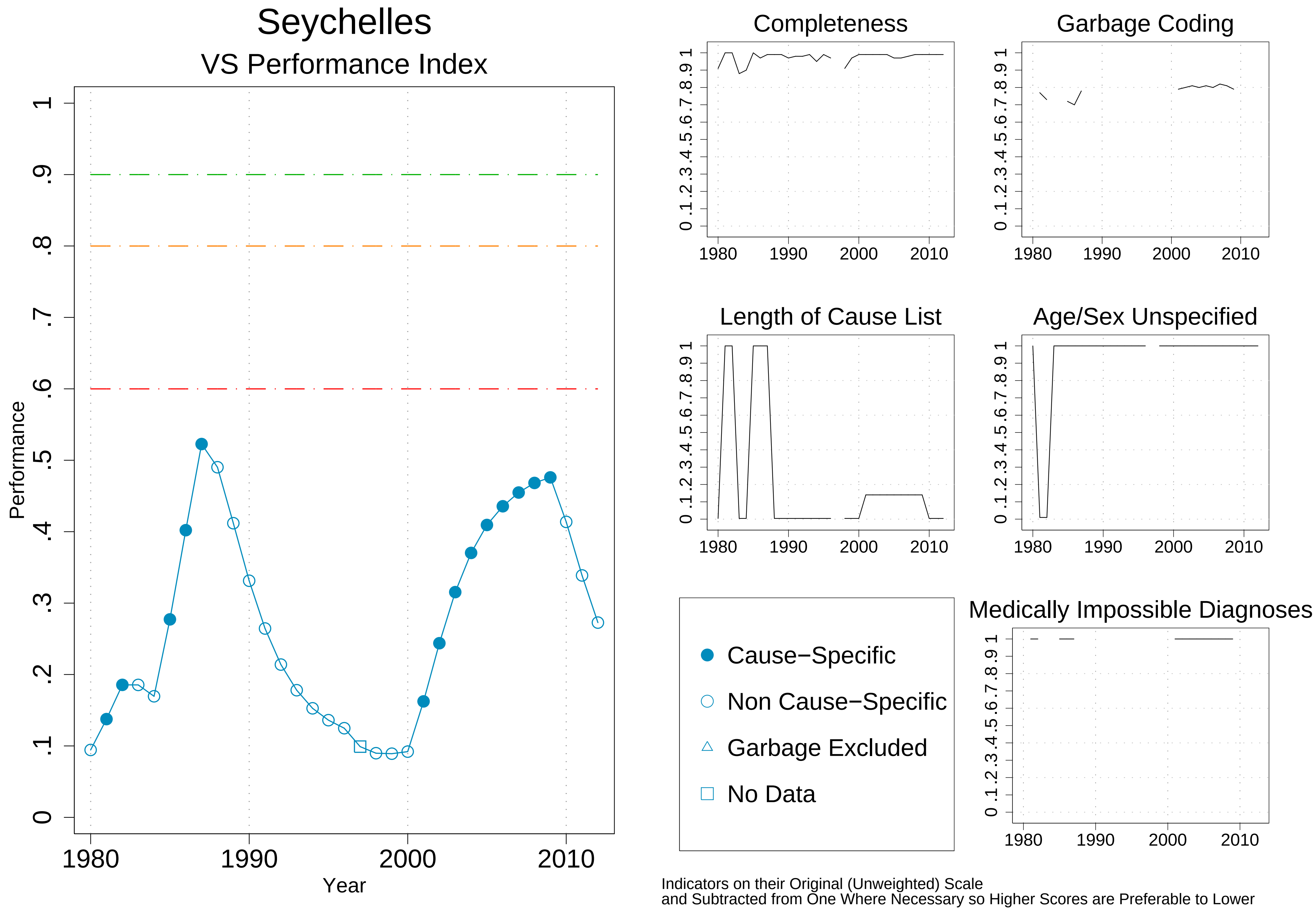

Seychelles
VS Performance Index
Performance
Year
Completeness
Garbage Coding
Length of Cause List
Age/Sex Unspecified
Medically Impossible Diagnoses
Cause−Specific
Non Cause−Specific
Garbage Excluded
No Data
Indicators on their Original (Unweighted) Scale
and Subtracted from One Where Necessary so Higher Scores are Preferable to Lower

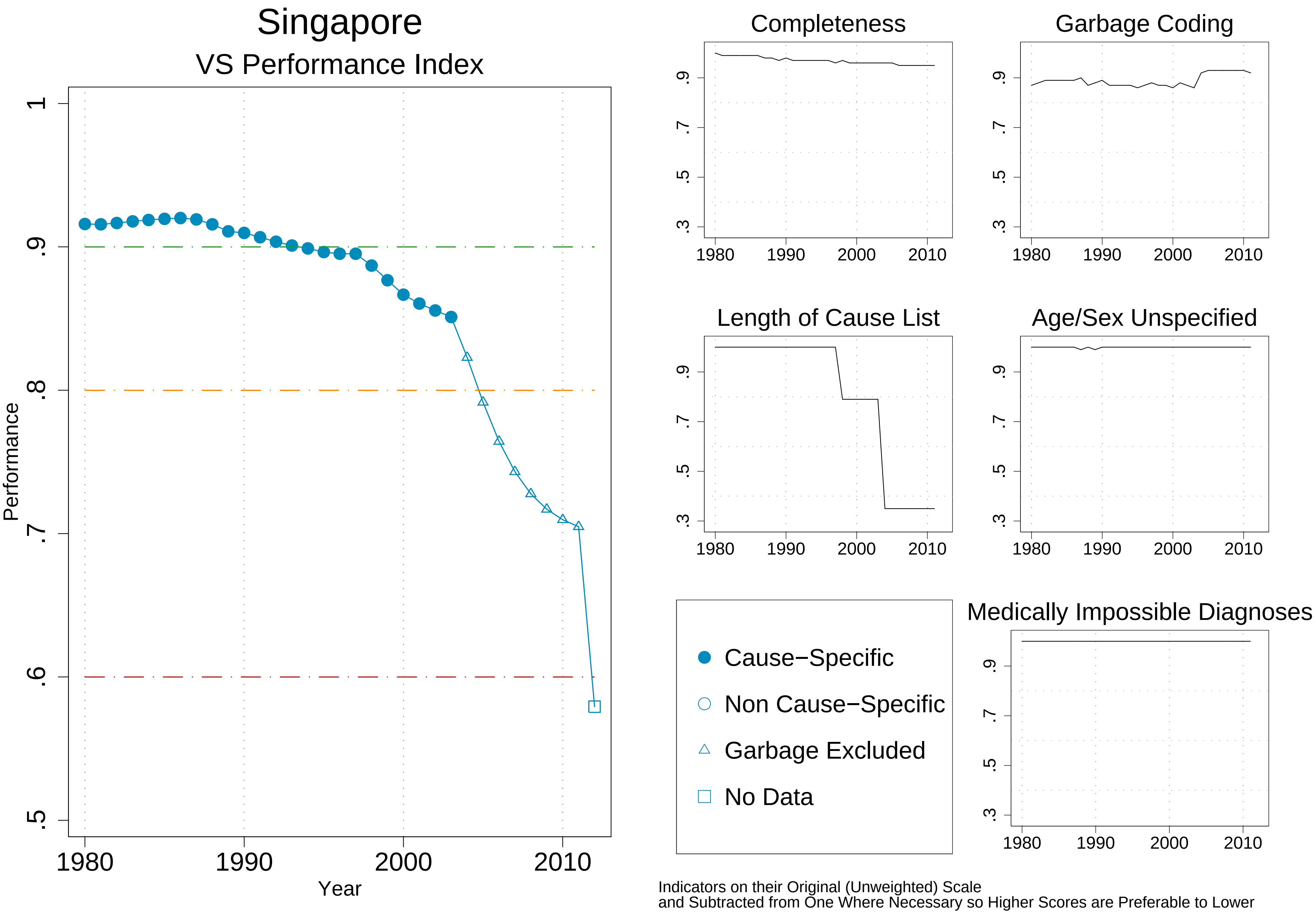

Indicators on their Original (Unweighted) Scale
and Subtracted from One Where Necessary so Higher Scores are Preferable to Lower

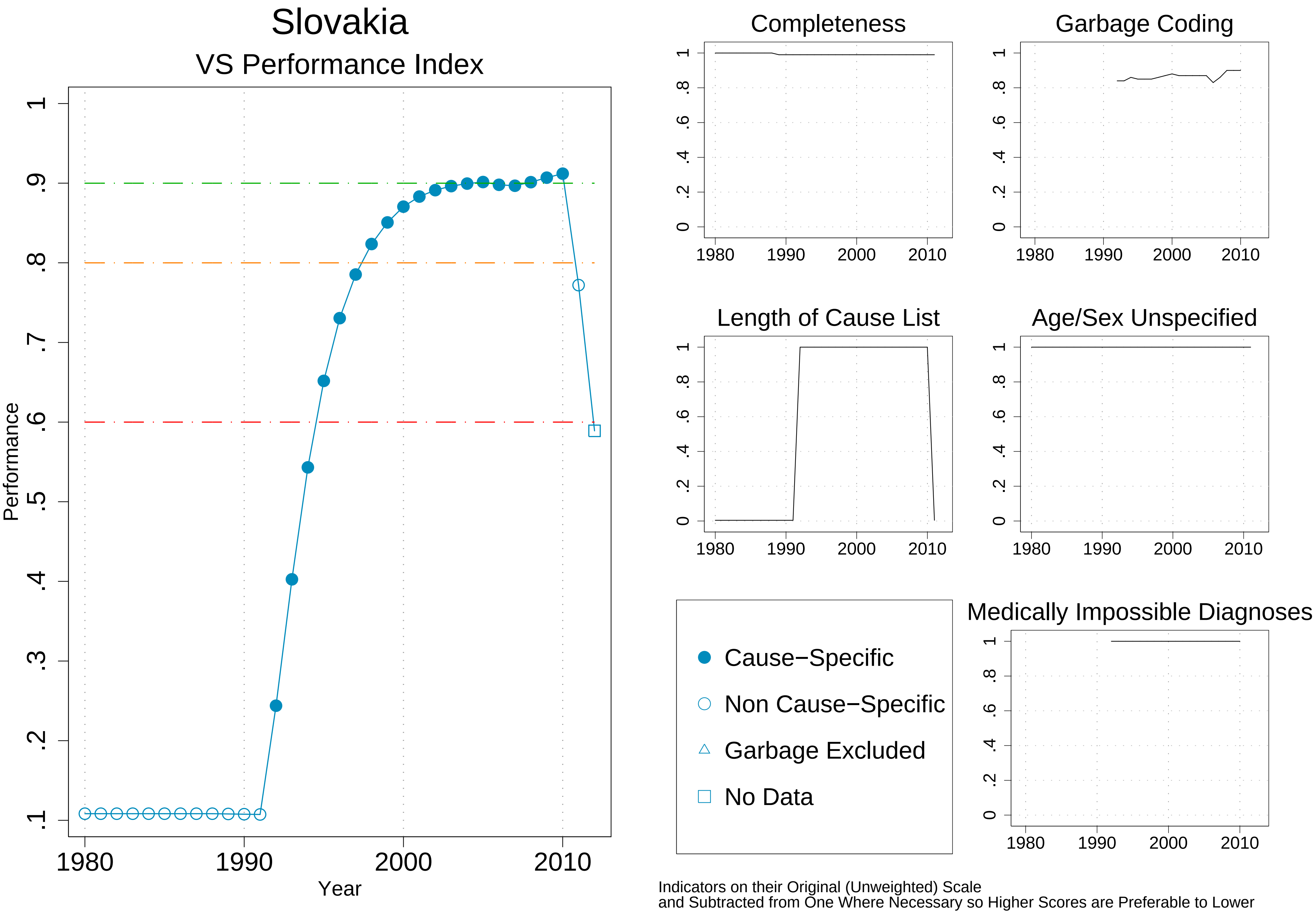

Indicators on their Original (Unweighted) Scale
and Subtracted from One Where Necessary so Higher Scores are Preferable to Lower

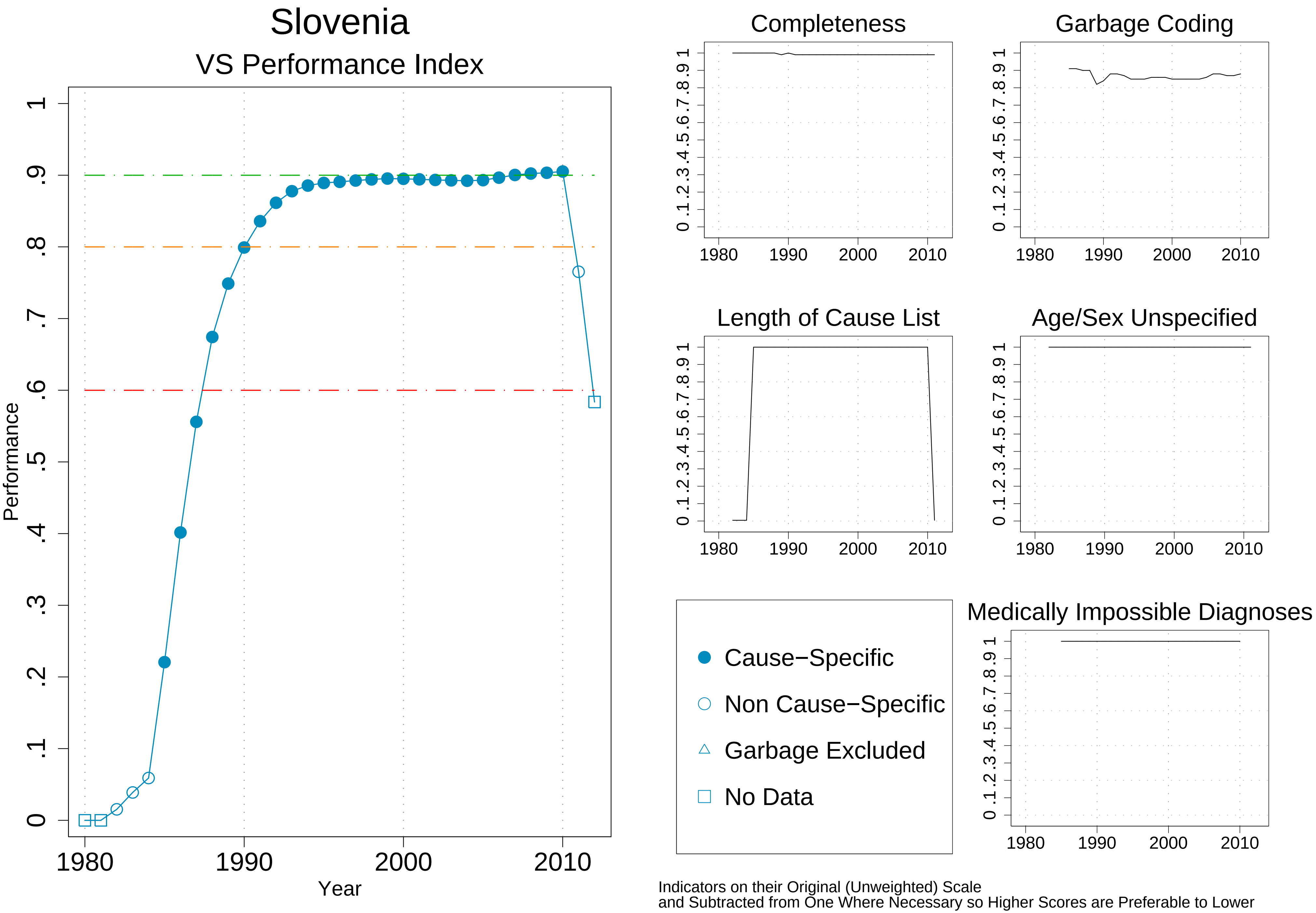

Indicators on their Original (Unweighted) Scale
and Subtracted from One Where Necessary so Higher Scores are Preferable to Lower

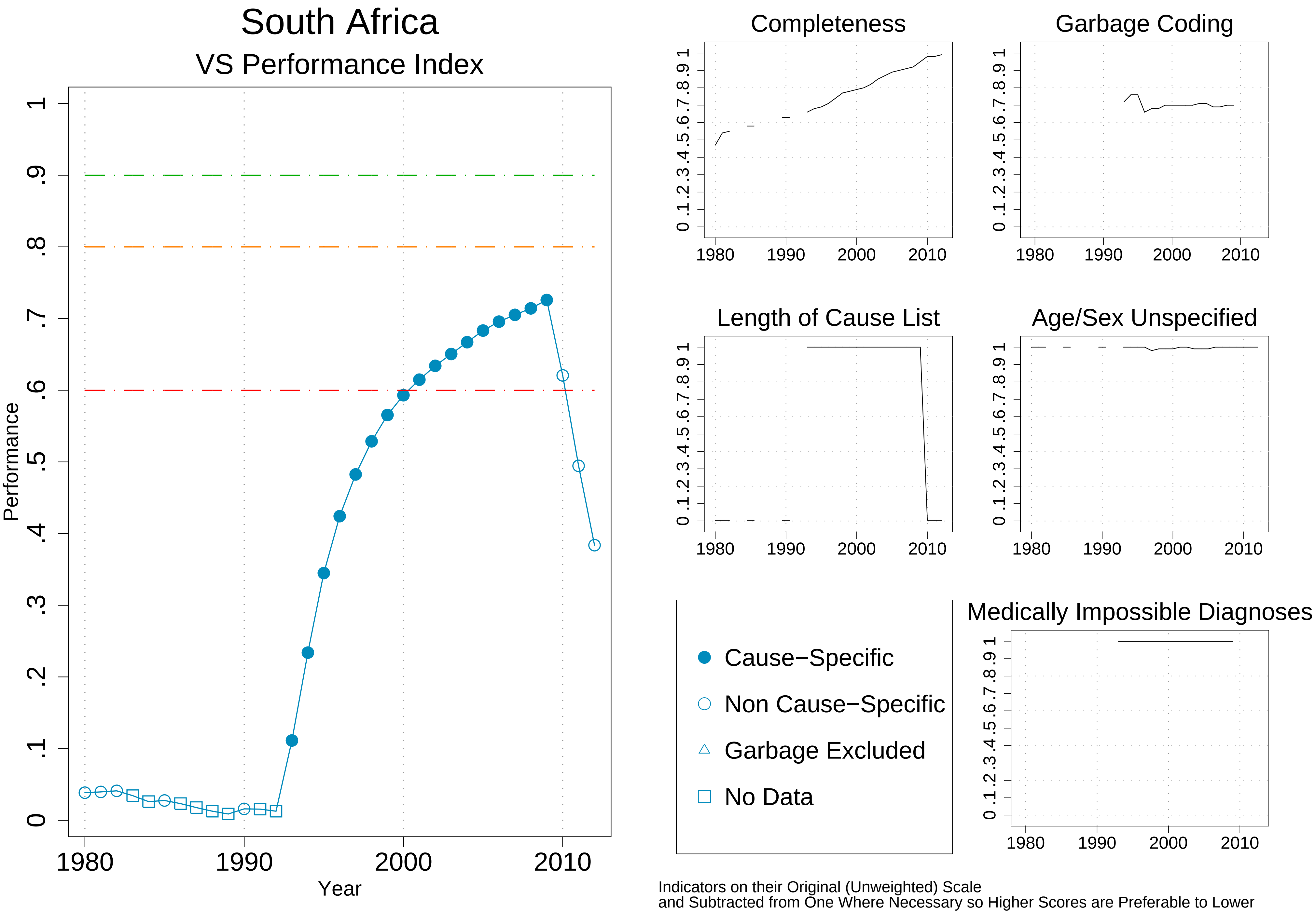

Indicators on their Original (Unweighted) Scale
and Subtracted from One Where Necessary so Higher Scores are Preferable to Lower

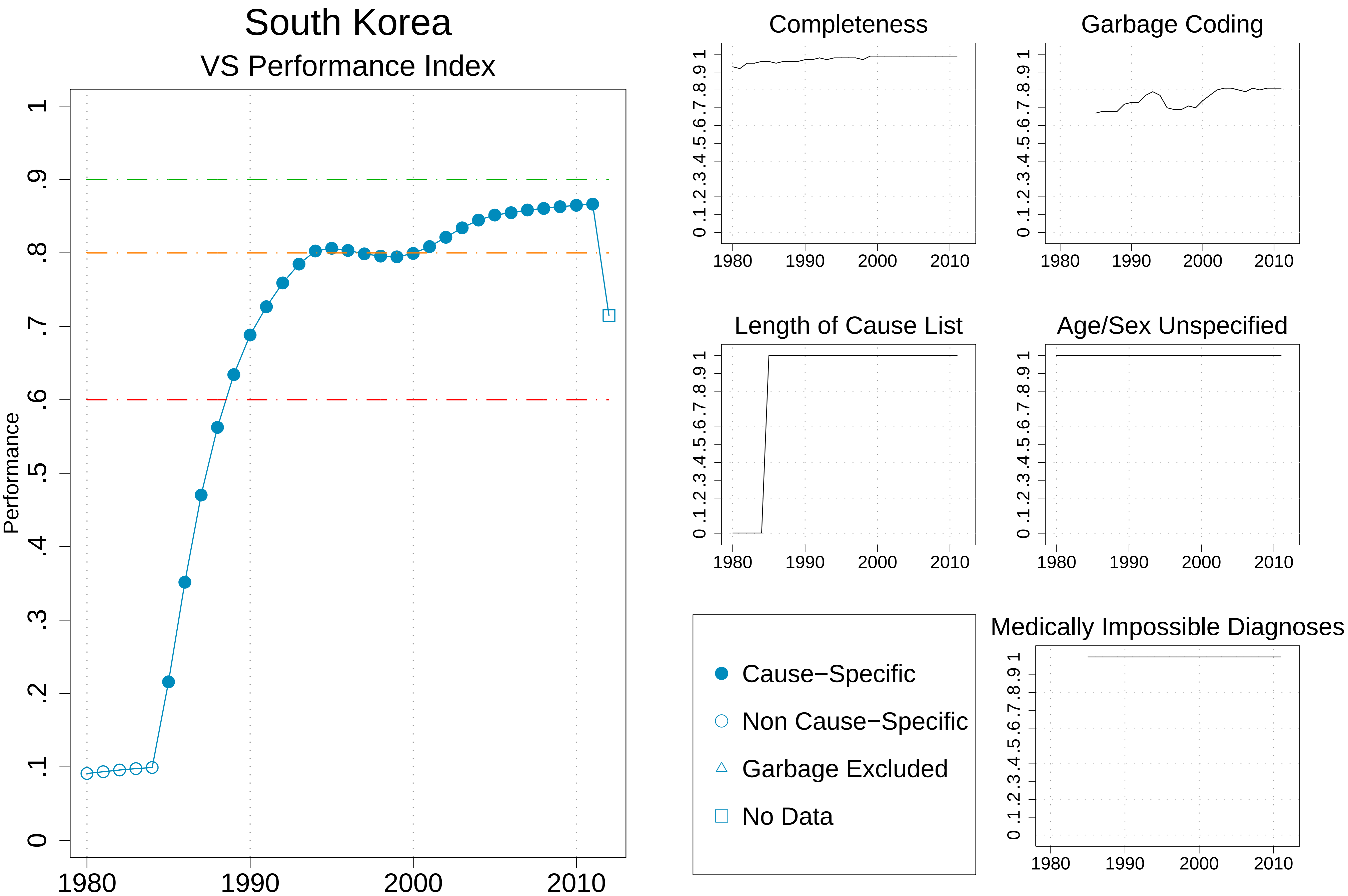

Indicators on their Original (Unweighted) Scale
and Subtracted from One Where Necessary so Higher Scores are Preferable to Lower

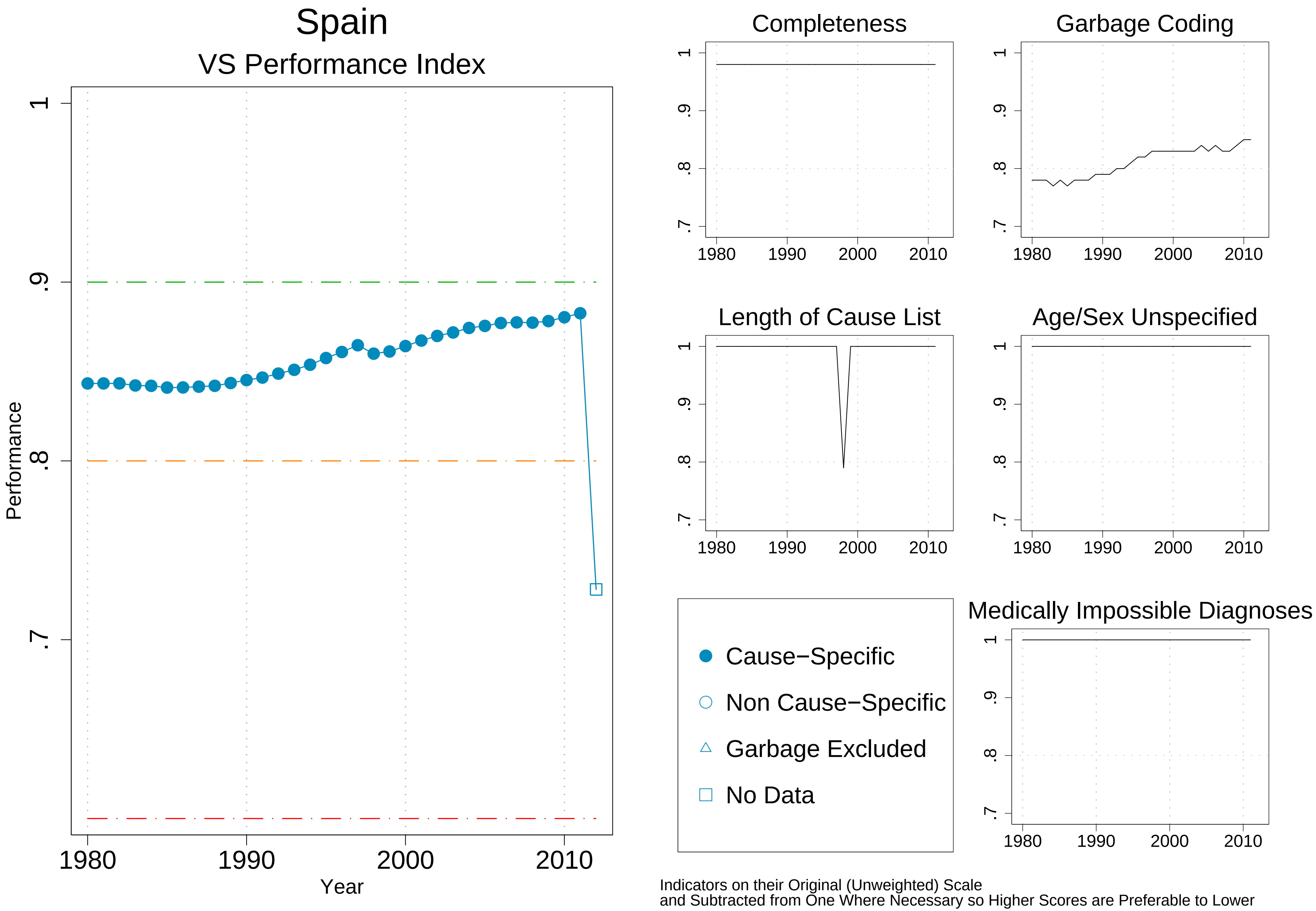

Indicators on their Original (Unweighted) Scale
and Subtracted from One Where Necessary so Higher Scores are Preferable to Lower

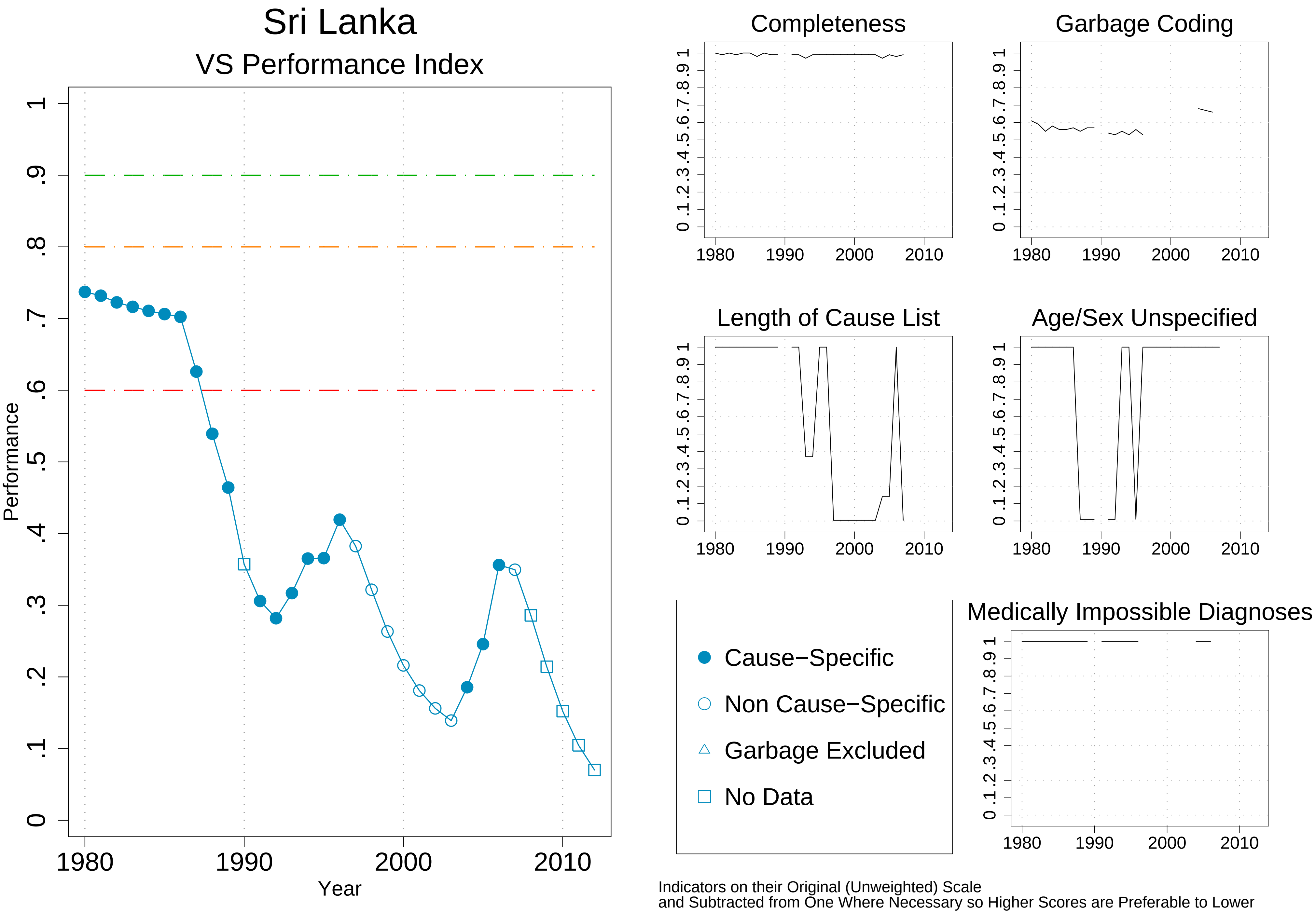

Indicators on their Original (Unweighted) Scale
and Subtracted from One Where Necessary so Higher Scores are Preferable to Lower

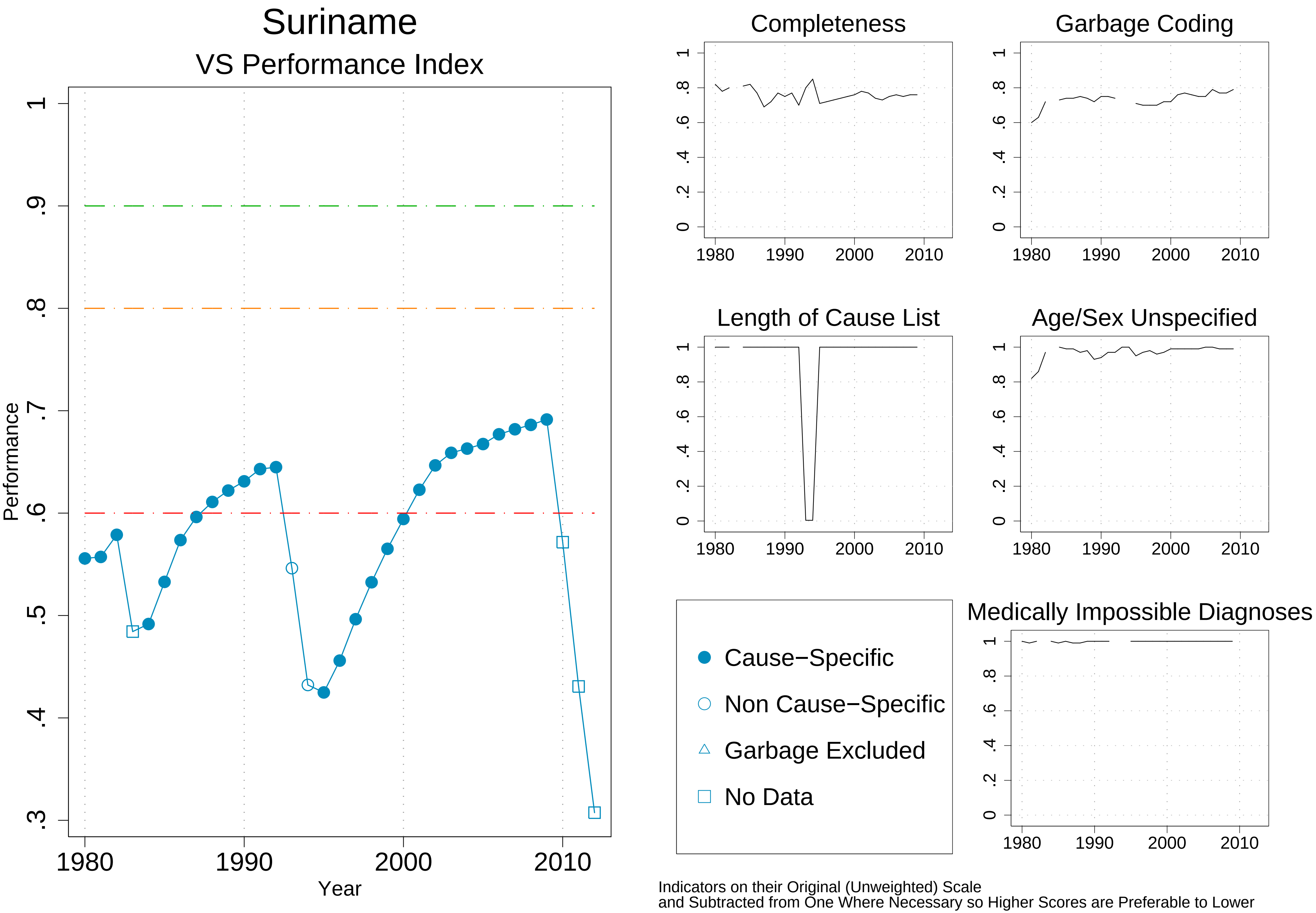

Indicators on their Original (Unweighted) Scale
and Subtracted from One Where Necessary so Higher Scores are Preferable to Lower

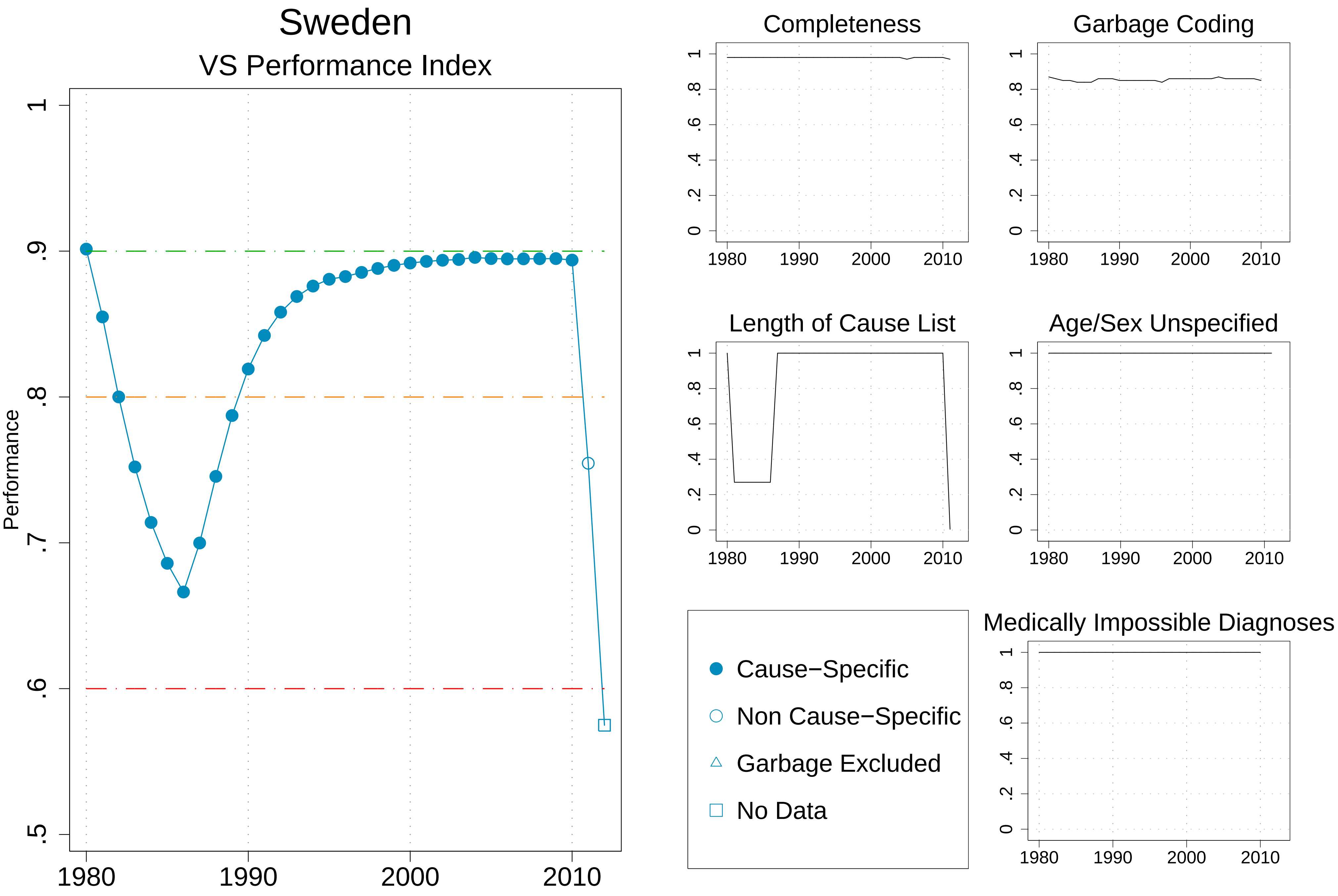

Indicators on their Original (Unweighted) Scale
and Subtracted from One Where Necessary so Higher Scores are Preferable to Lower

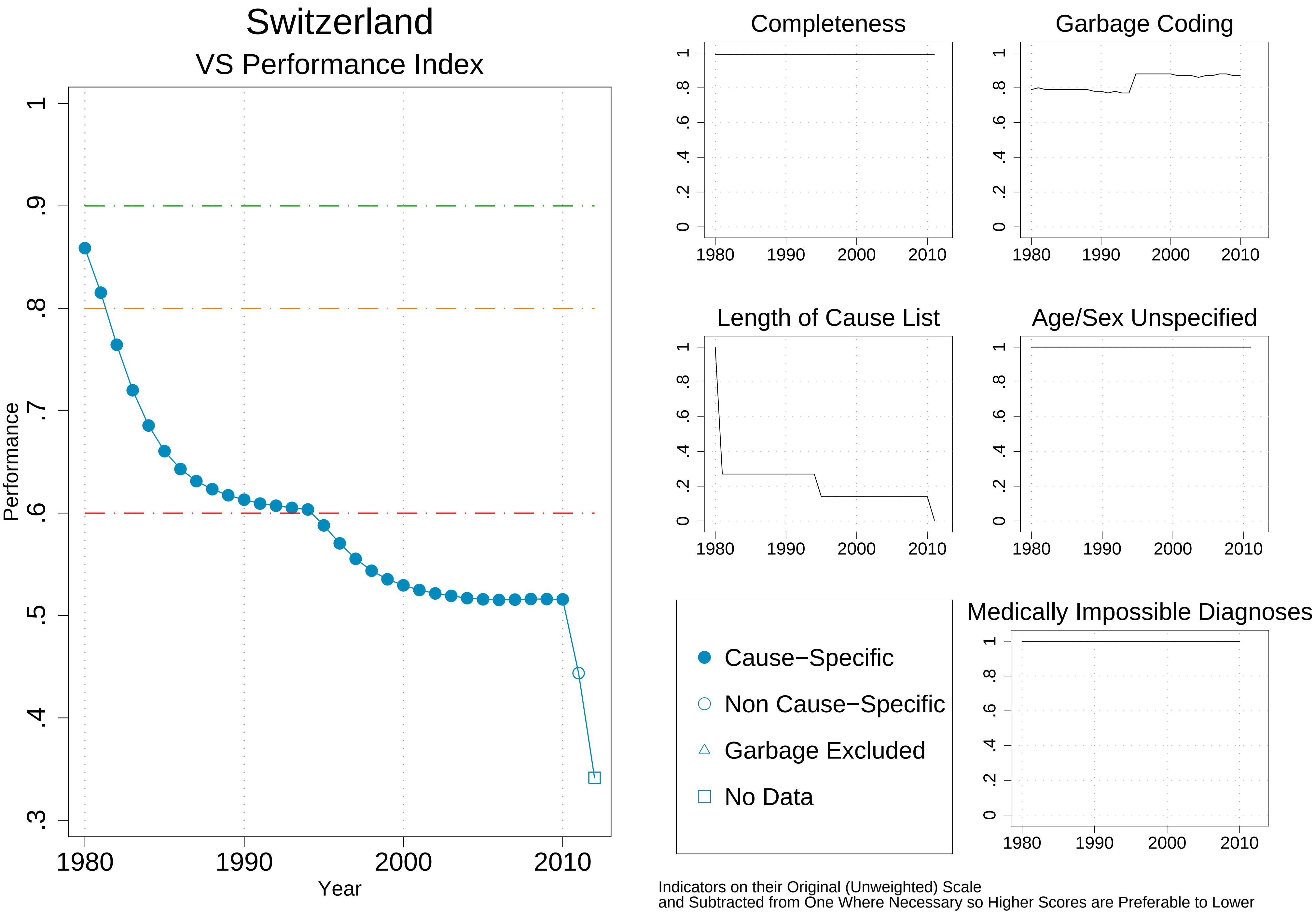

Indicators on their Original (Unweighted) Scale
and Subtracted from One Where Necessary so Higher Scores are Preferable to Lower

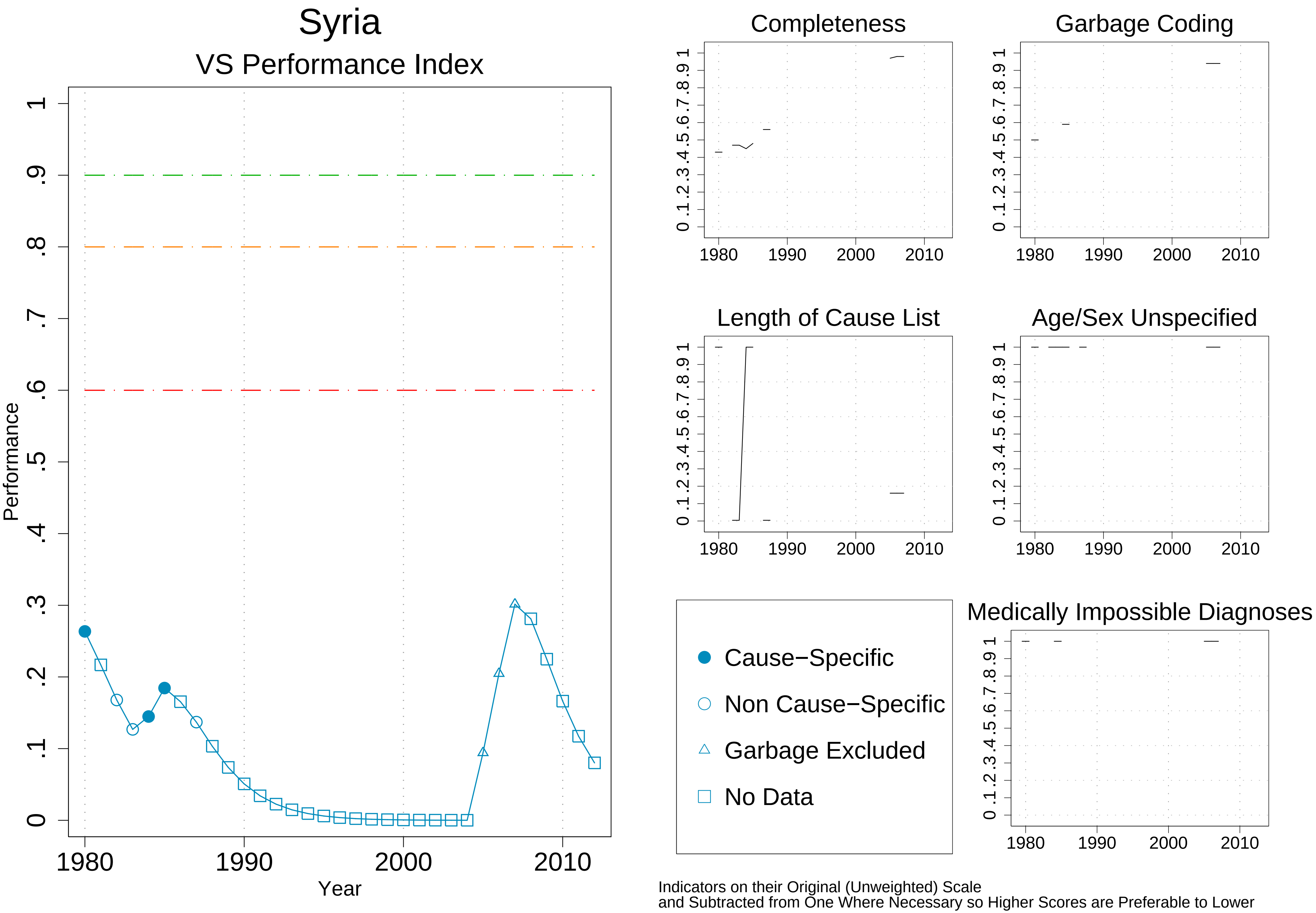

Indicators on their Original (Unweighted) Scale
and Subtracted from One Where Necessary so Higher Scores are Preferable to Lower

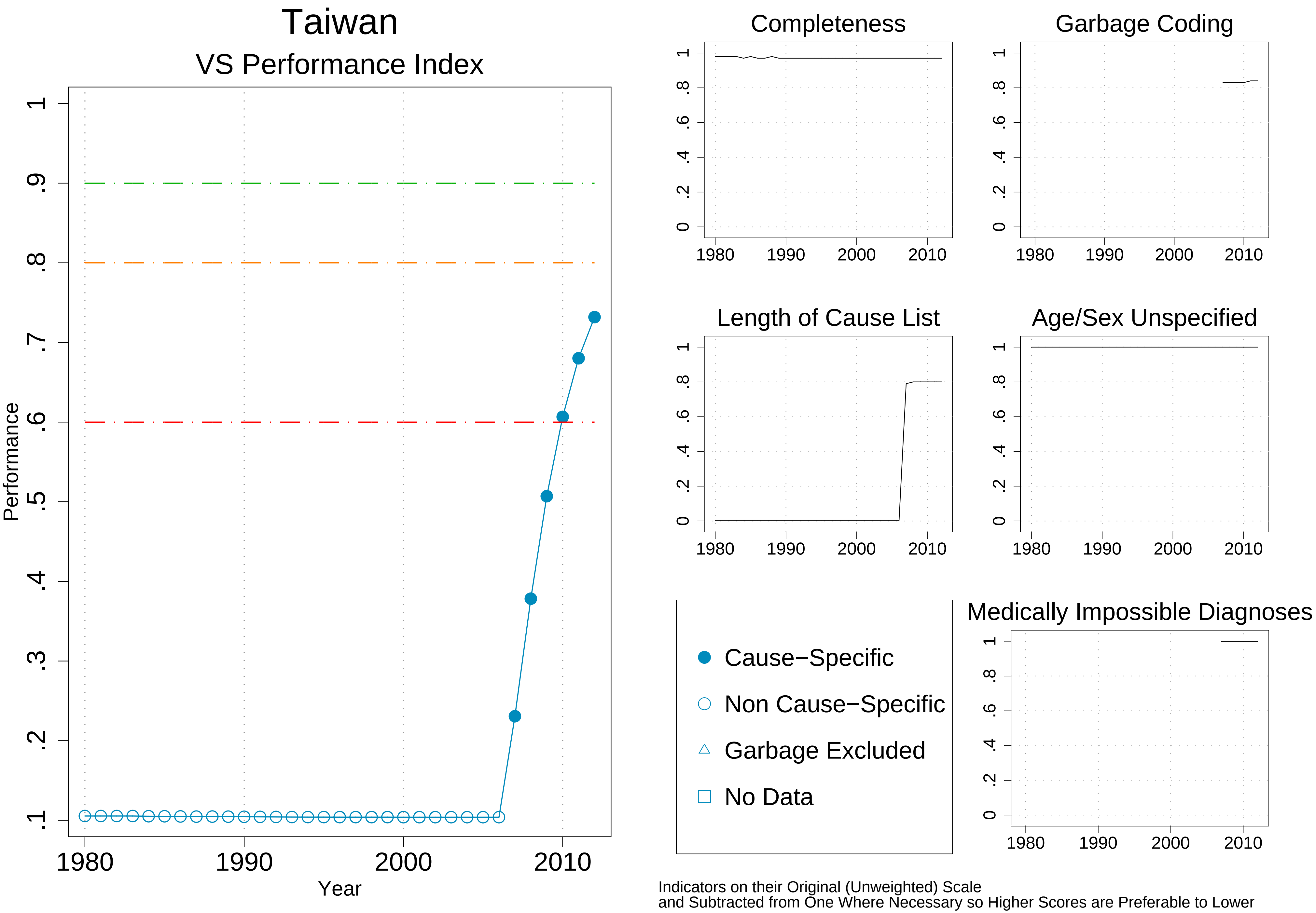

Indicators on their Original (Unweighted) Scale
and Subtracted from One Where Necessary so Higher Scores are Preferable to Lower

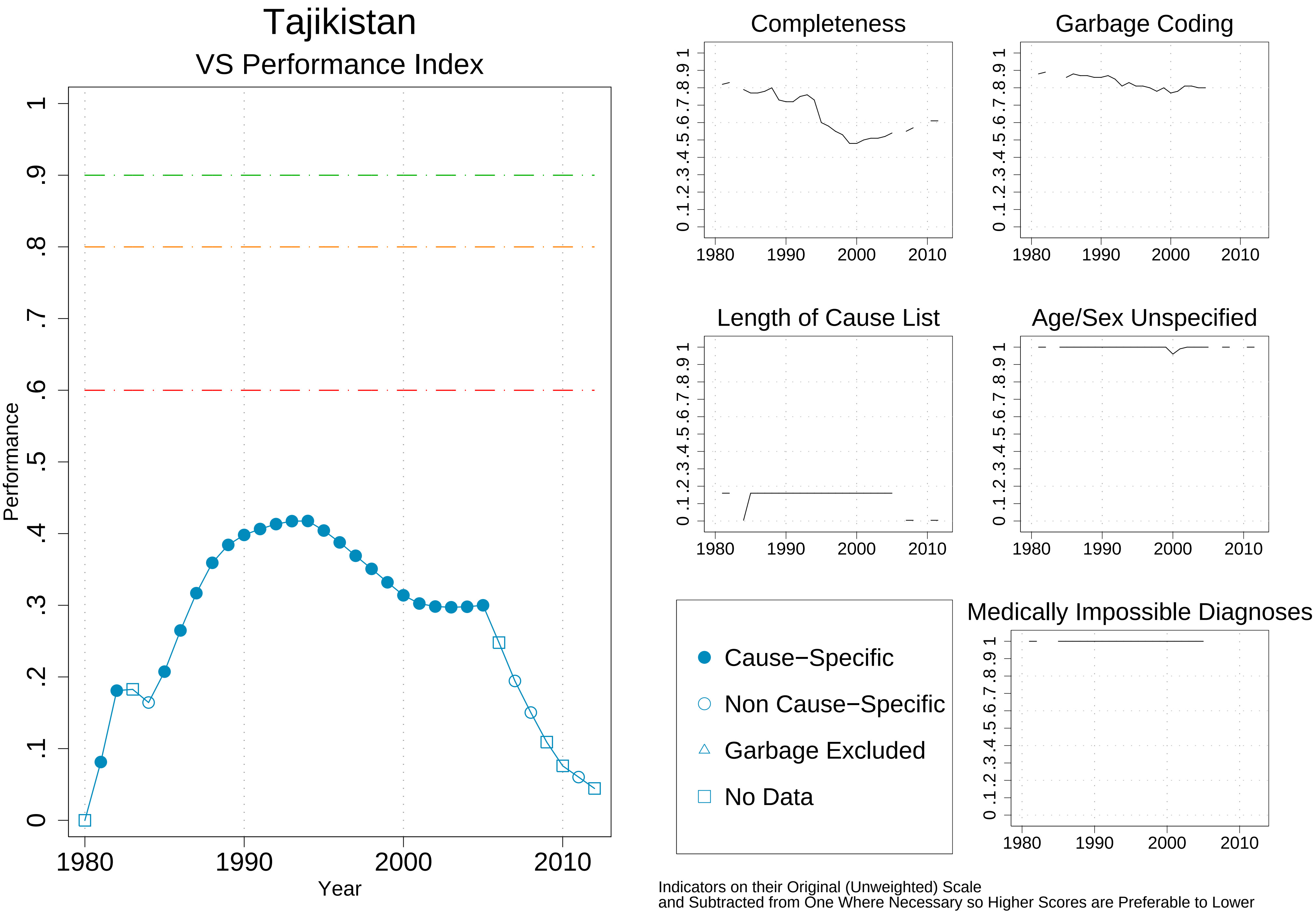

Indicators on their Original (Unweighted) Scale
and Subtracted from One Where Necessary so Higher Scores are Preferable to Lower

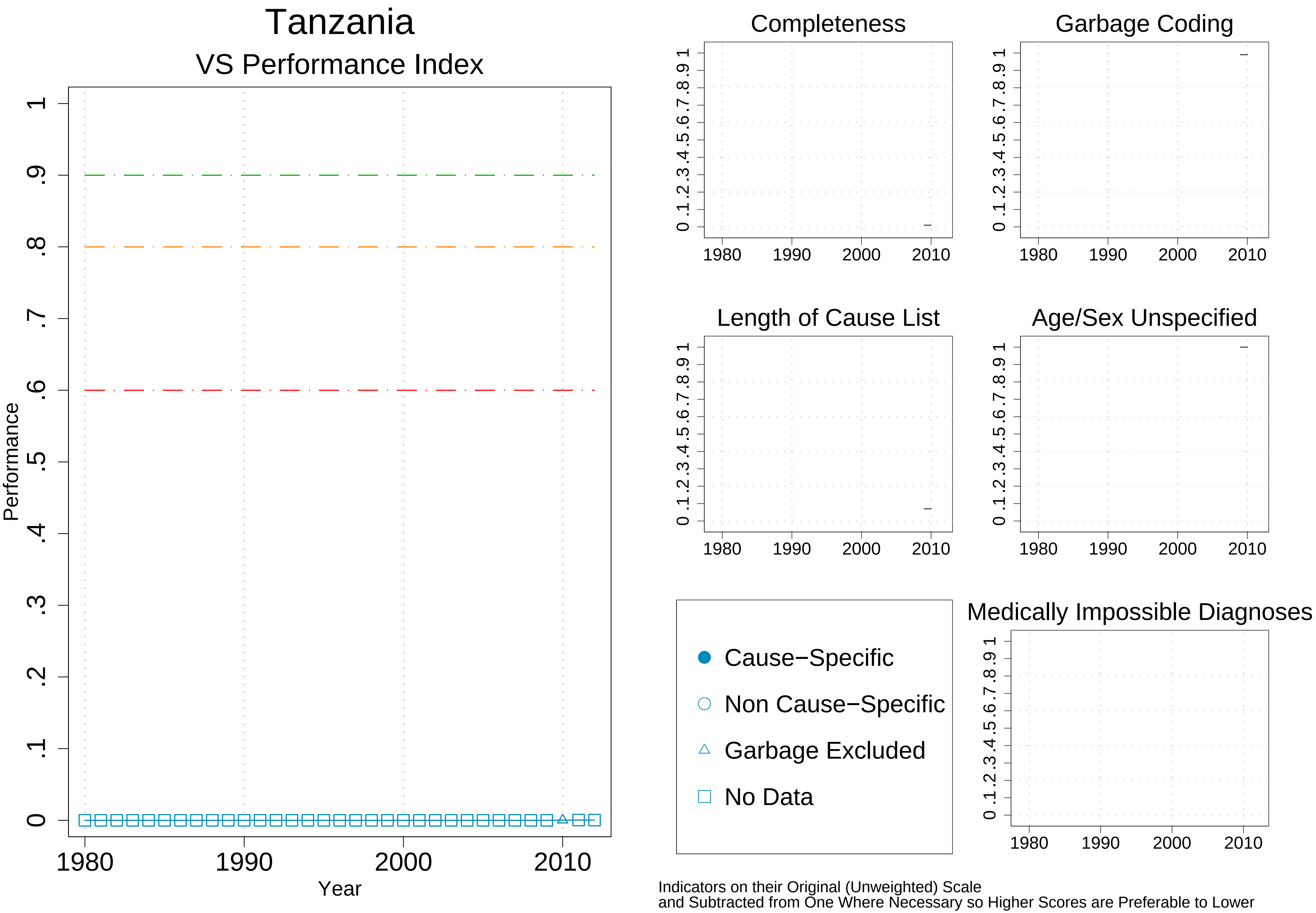

Indicators on their Original (Unweighted) Scale
and Subtracted from One Where Necessary so Higher Scores are Preferable to Lower

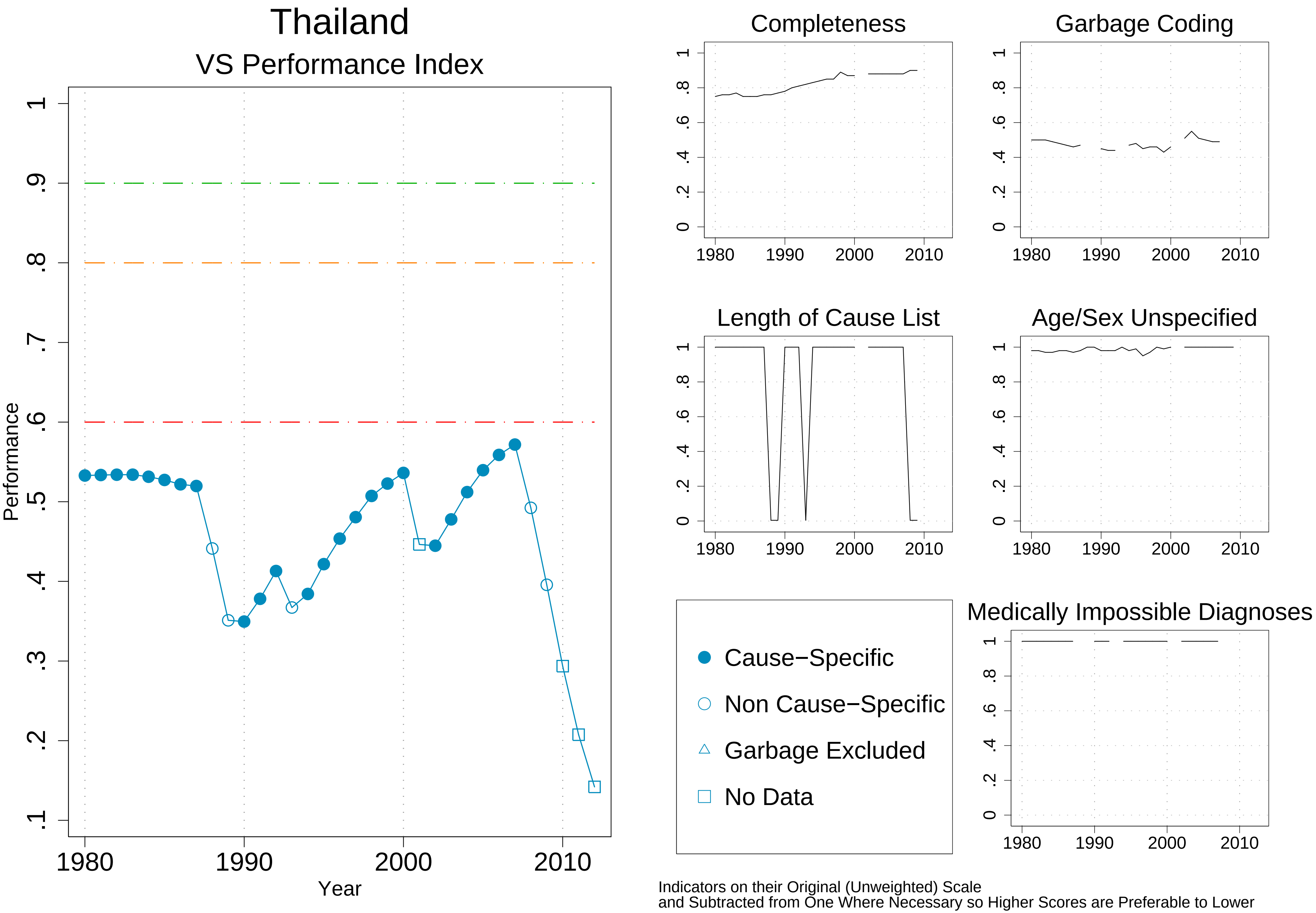

Indicators on their Original (Unweighted) Scale
and Subtracted from One Where Necessary so Higher Scores are Preferable to Lower

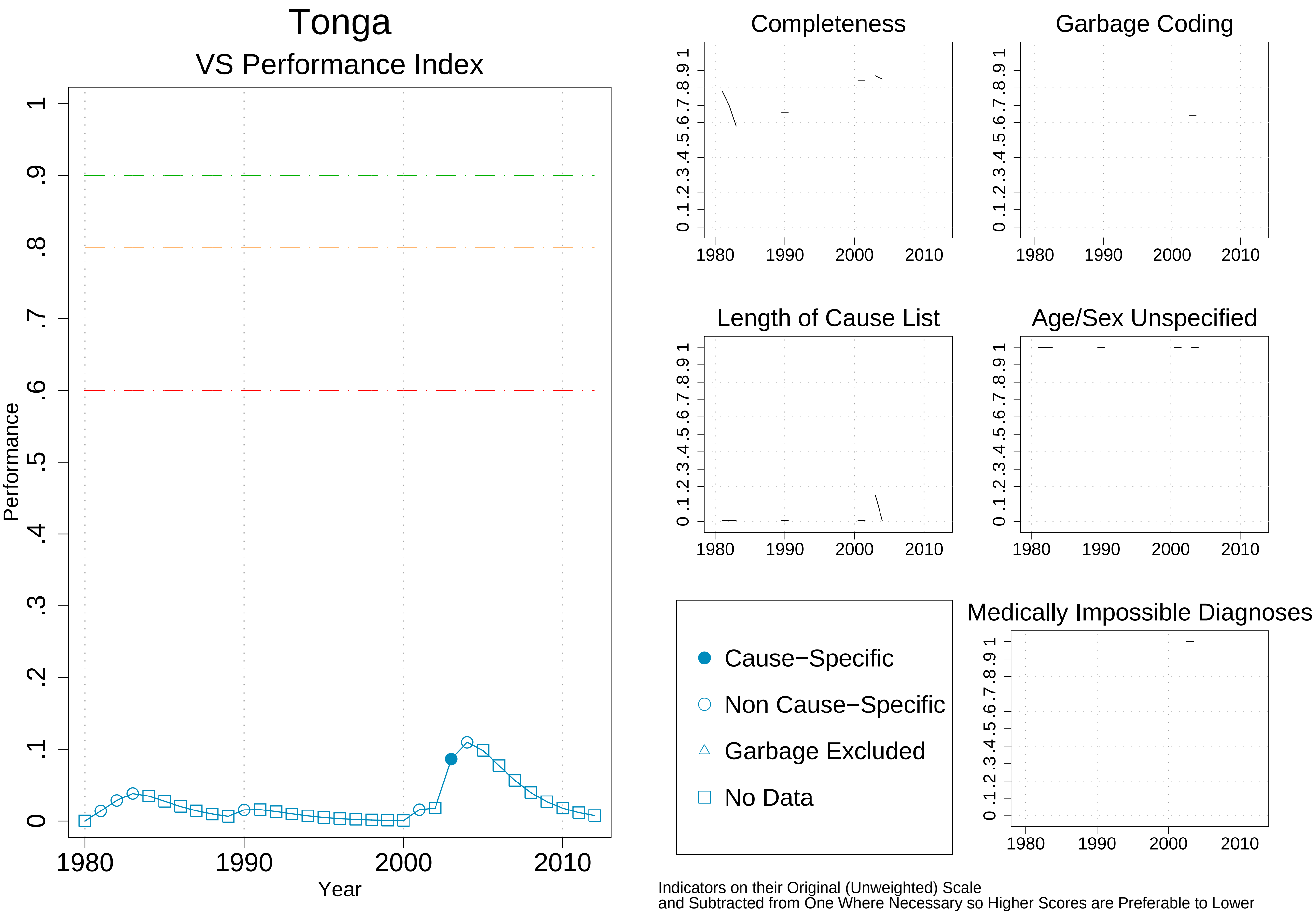
Tonga
VS Performance Index
Performance
Year
Completeness
Garbage Coding
Length of Cause List
Age/Sex Unspecified
Medically Impossible Diagnoses
Cause−Specific
Non Cause−Specific
Garbage Excluded
No Data
Indicators on their Original (Unweighted) Scale
and Subtracted from One Where Necessary so Higher Scores are Preferable to Lower

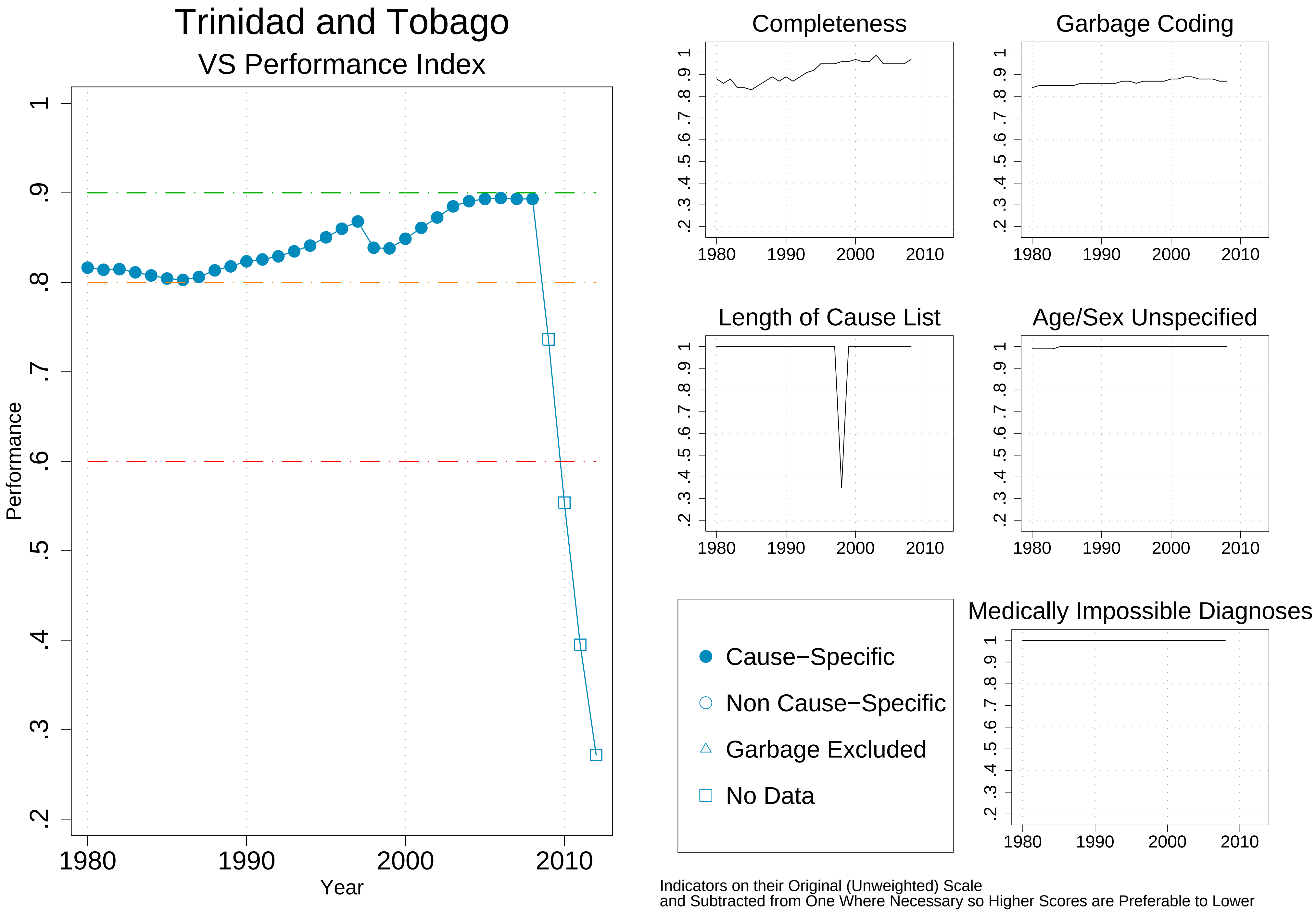
Trinidad and Tobago
VS Performance Index
Performance
Year
Completeness
Garbage Coding
Length of Cause List
Age/Sex Unspecified
Medically Impossible Diagnoses
Cause−Specific
Non Cause−Specific
Garbage Excluded
No Data
Indicators on their Original (Unweighted) Scale
and Subtracted from One Where Necessary so Higher Scores are Preferable to Lower

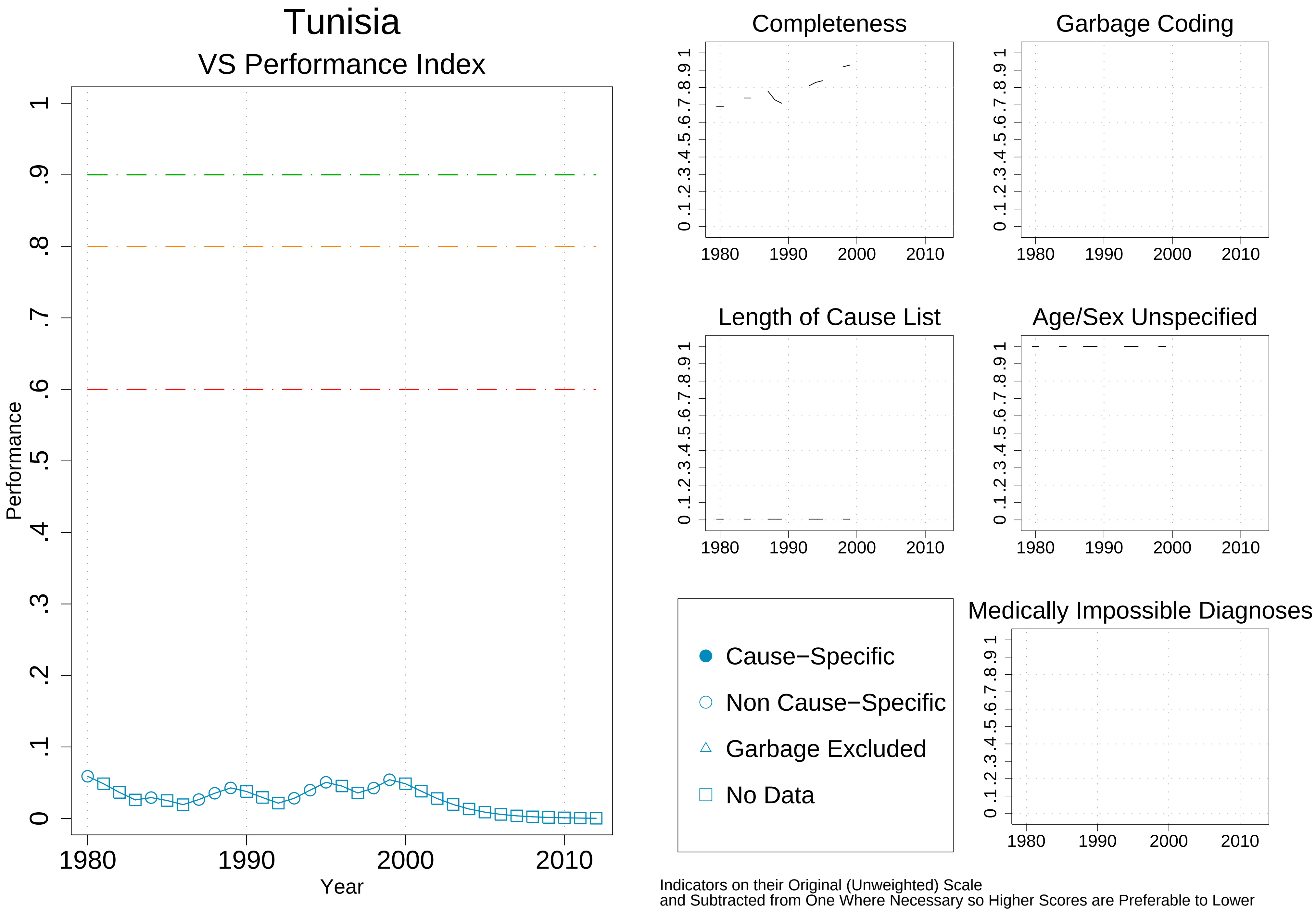

Indicators on their Original (Unweighted) Scale
and Subtracted from One Where Necessary so Higher Scores are Preferable to Lower

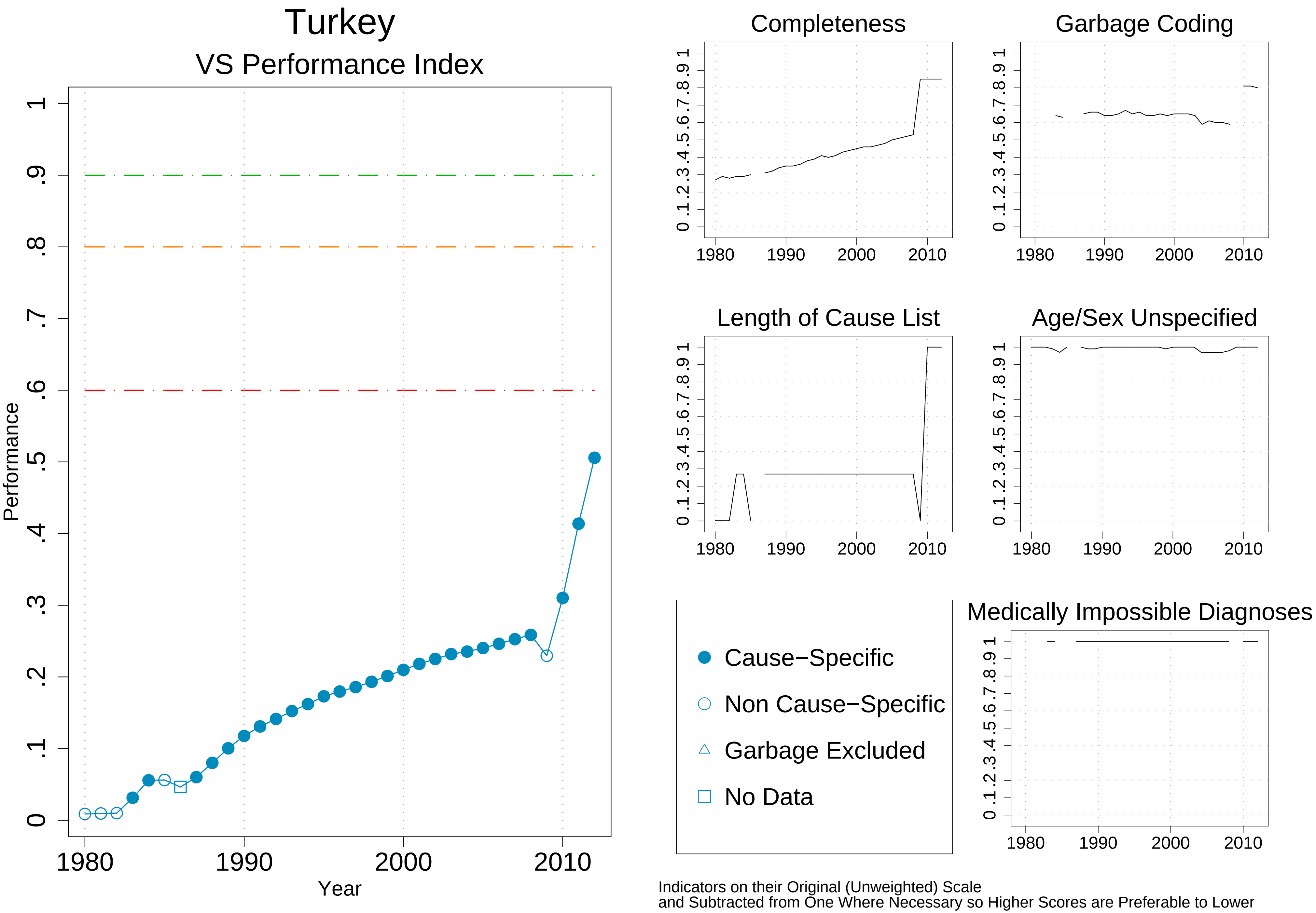

Indicators on their Original (Unweighted) Scale
and Subtracted from One Where Necessary so Higher Scores are Preferable to Lower

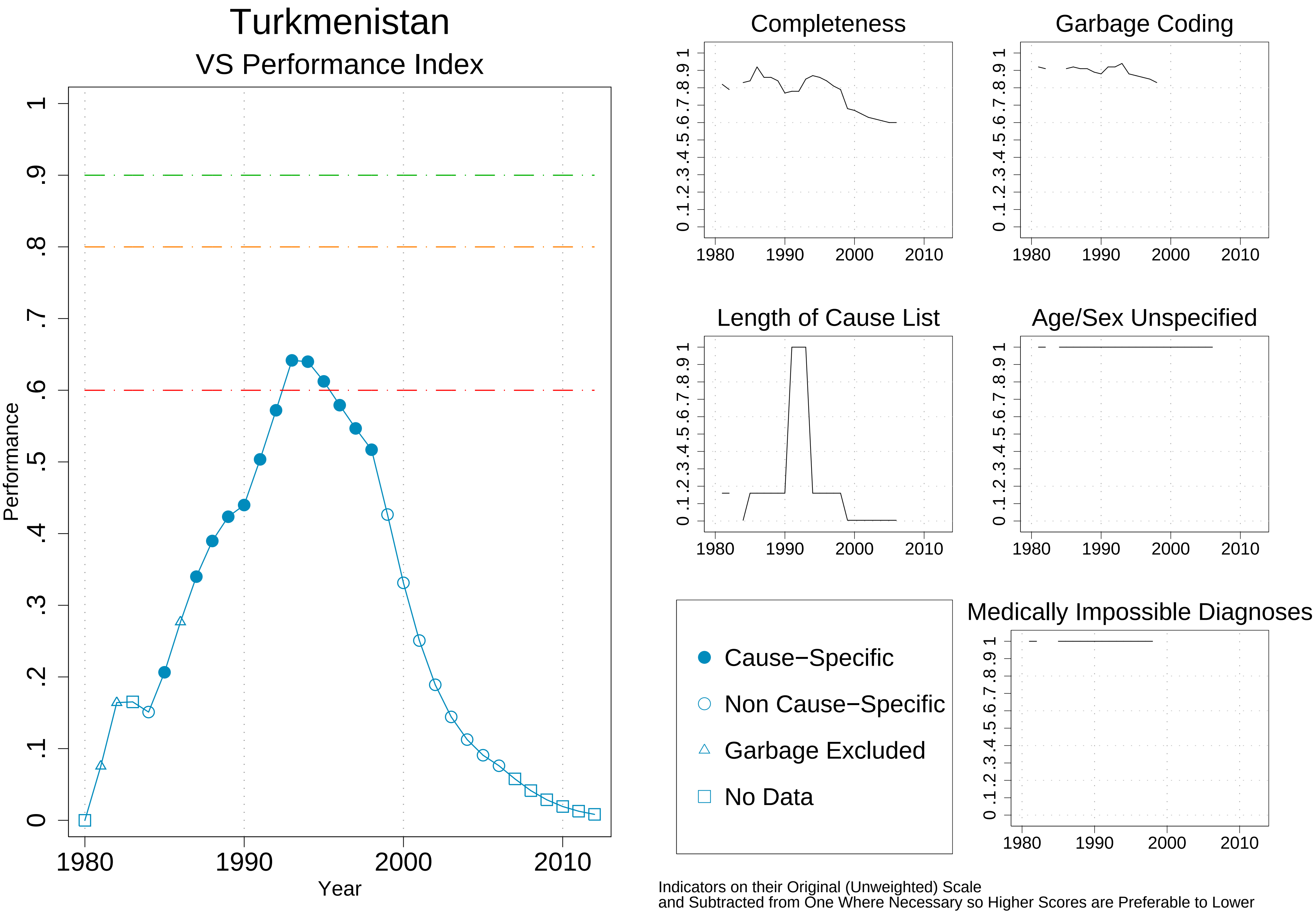

Indicators on their Original (Unweighted) Scale
and Subtracted from One Where Necessary so Higher Scores are Preferable to Lower

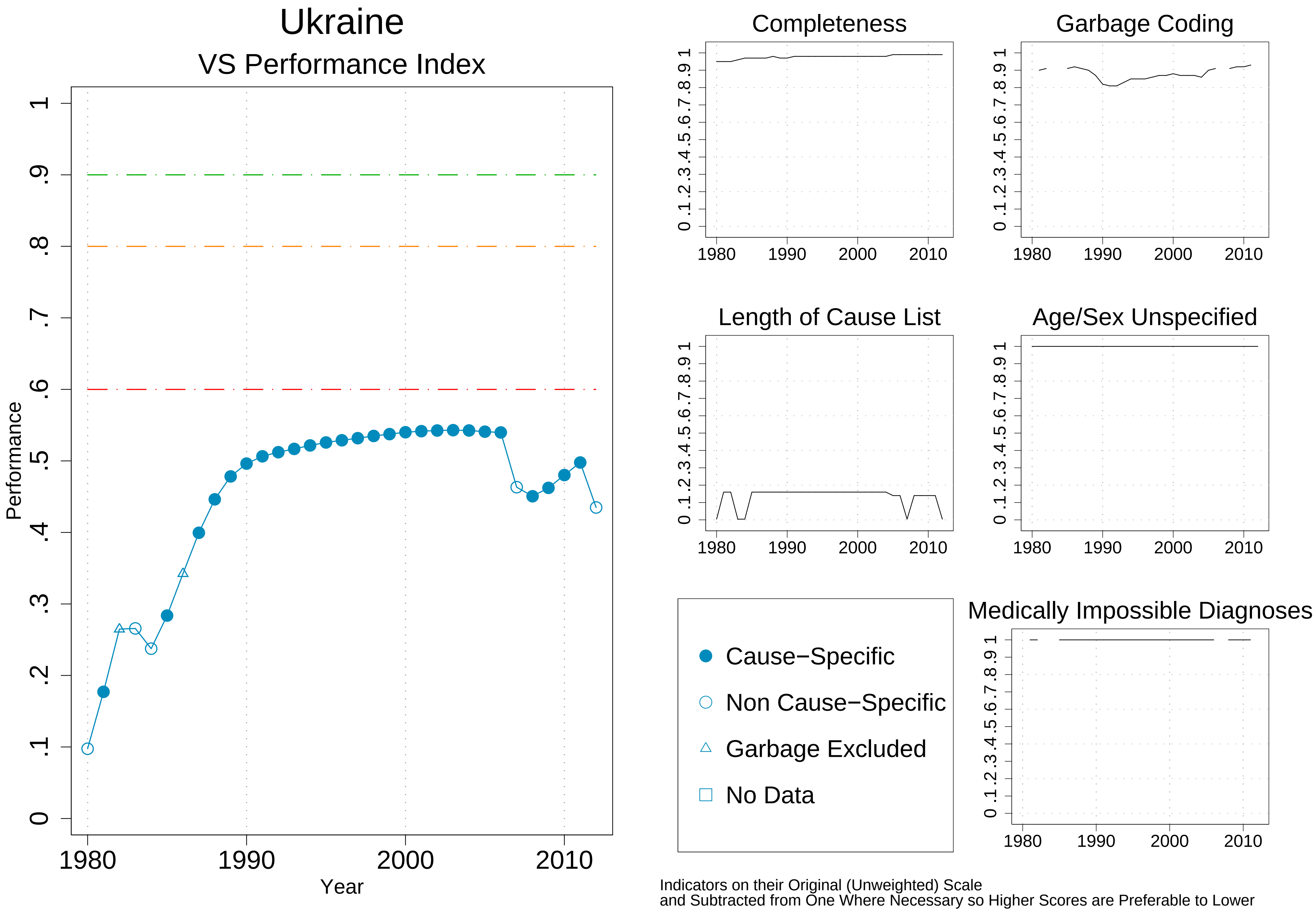

Indicators on their Original (Unweighted) Scale
and Subtracted from One Where Necessary so Higher Scores are Preferable to Lower

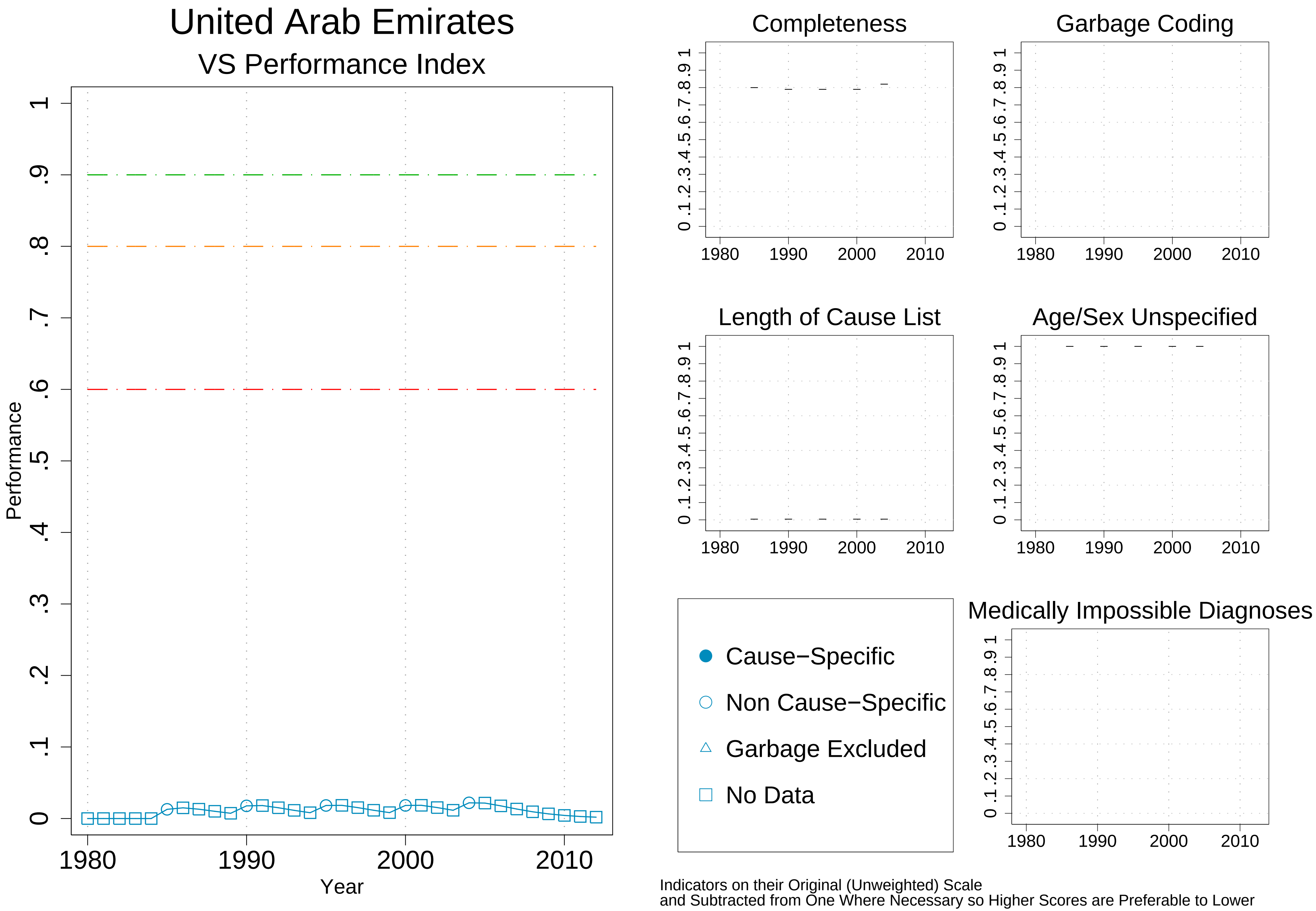

Indicators on their Original (Unweighted) Scale
and Subtracted from One Where Necessary so Higher Scores are Preferable to Lower

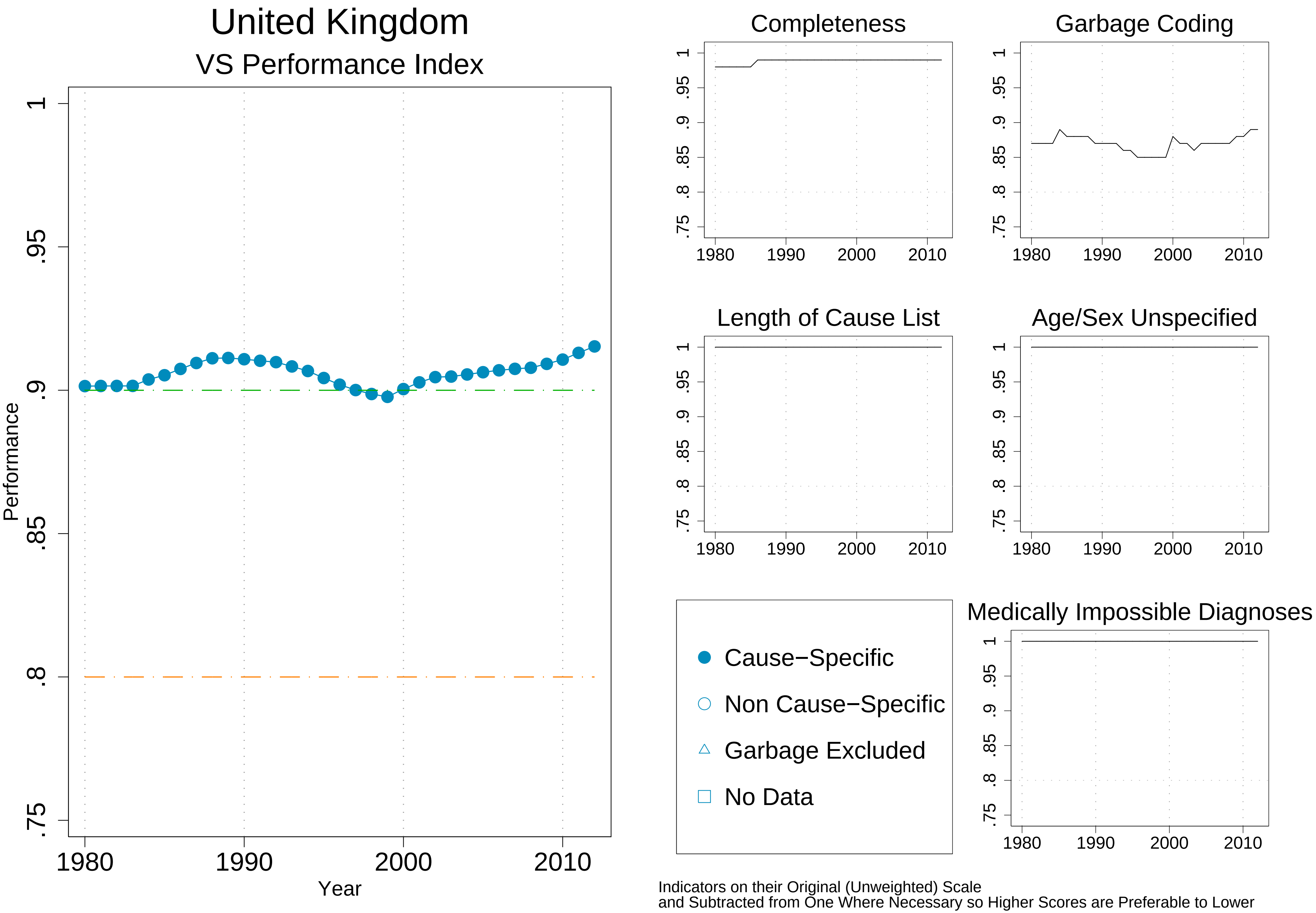

Indicators on their Original (Unweighted) Scale
and Subtracted from One Where Necessary so Higher Scores are Preferable to Lower

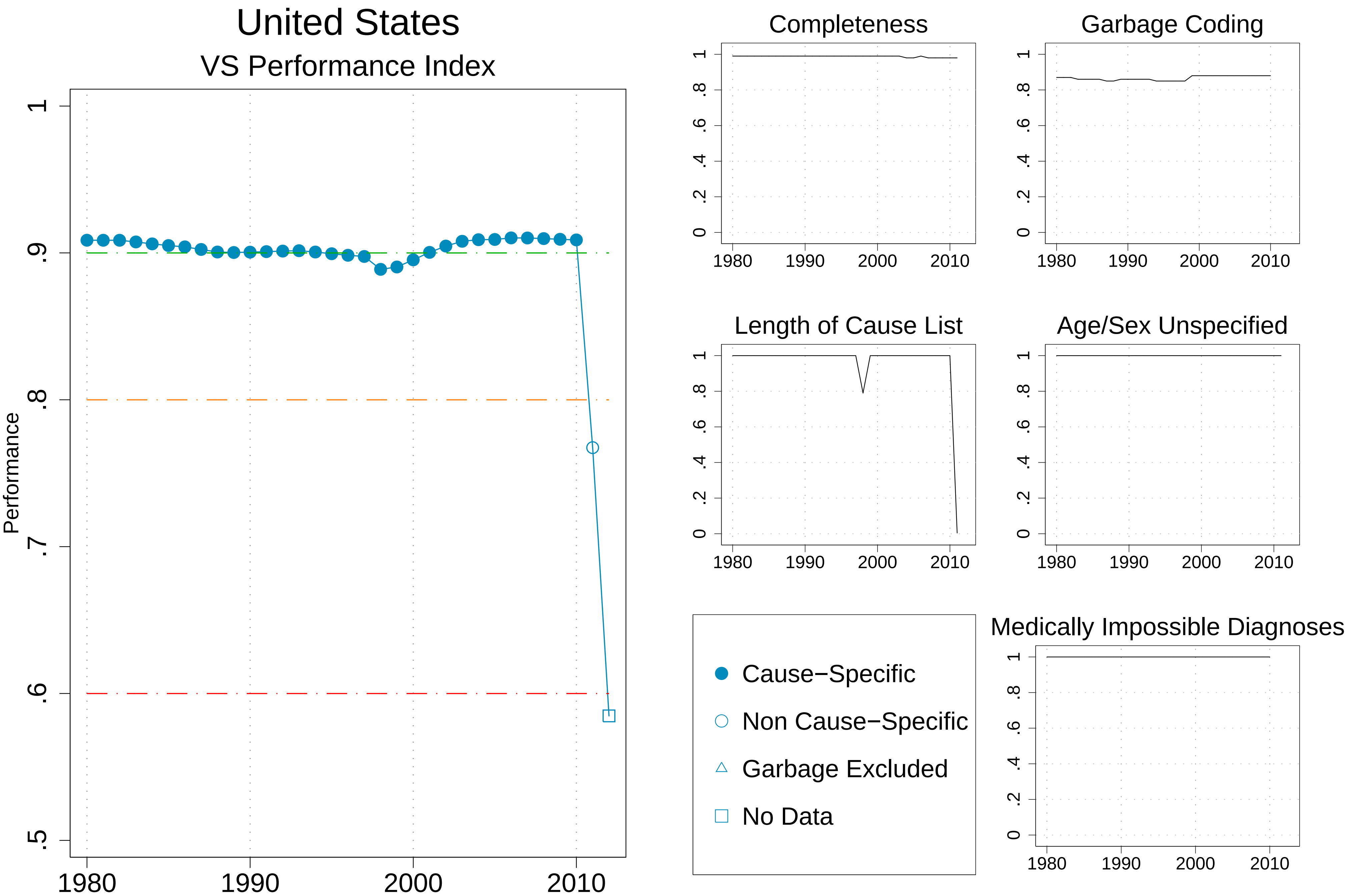

Indicators on their Original (Unweighted) Scale
and Subtracted from One Where Necessary so Higher Scores are Preferable to Lower

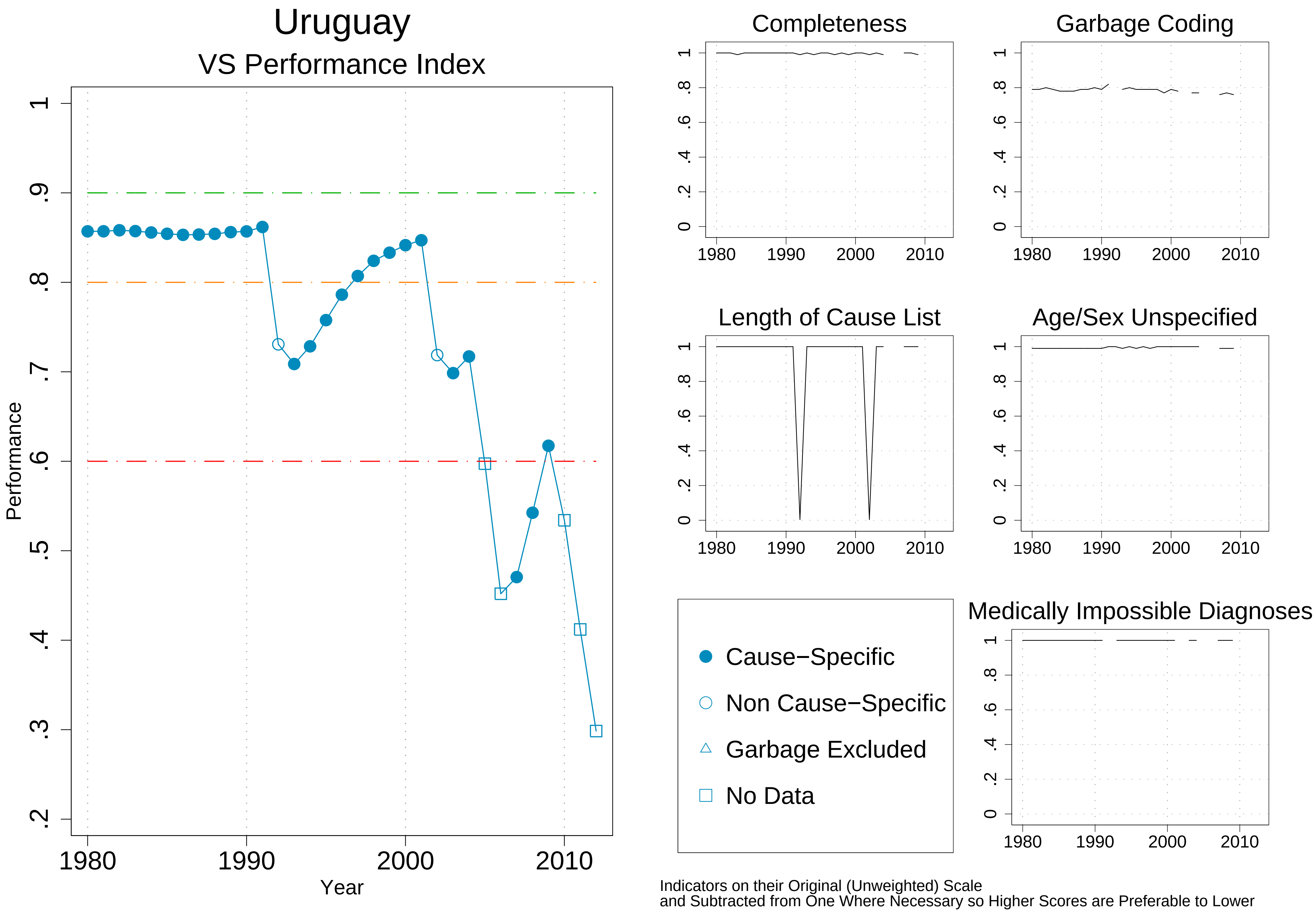

Uruguay
VS Performance Index
Performance
Year
1980
1990
2000
2010
.2
.3
.4
.5
.6
.7
.8
.9
1
Completeness
Garbage Coding
Length of Cause List
Age/Sex Unspecified
Medically Impossible Diagnoses
0
.2
.4
.6
.8
1
Cause−Specific
Non Cause−Specific
Garbage Excluded
No Data
Indicators on their Original (Unweighted) Scale
and Subtracted from One Where Necessary so Higher Scores are Preferable to Lower

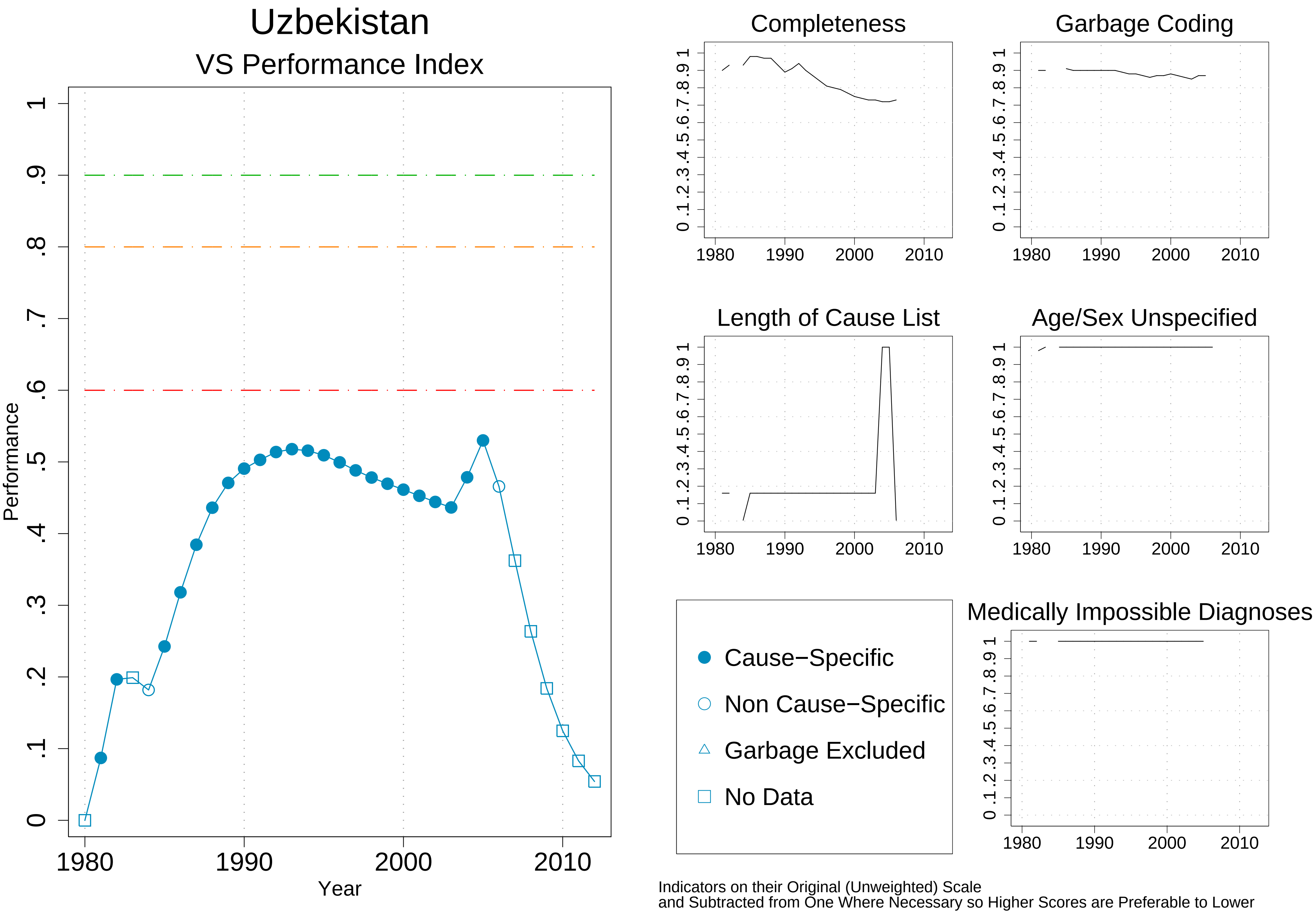

Indicators on their Original (Unweighted) Scale
and Subtracted from One Where Necessary so Higher Scores are Preferable to Lower

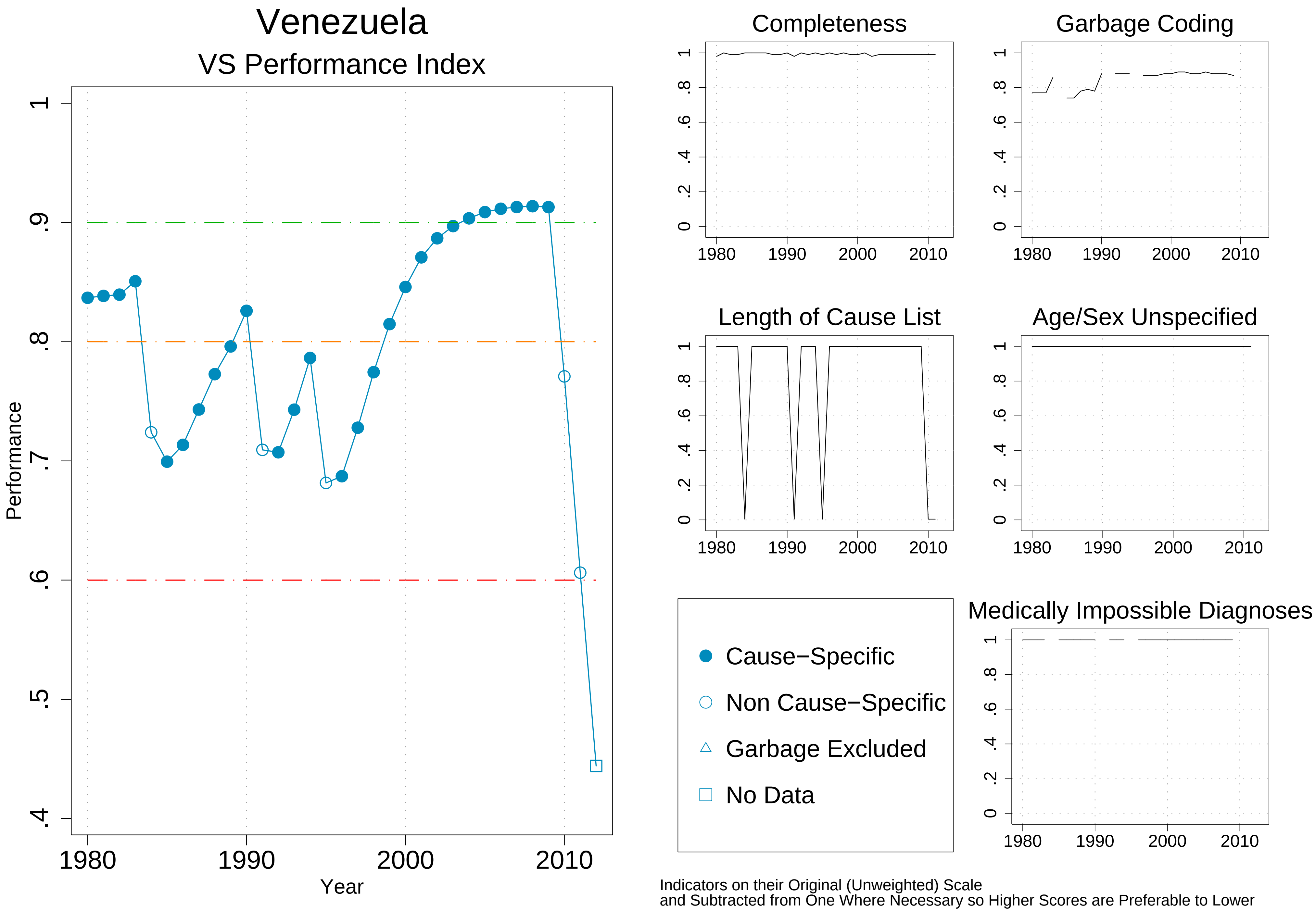

Indicators on their Original (Unweighted) Scale
and Subtracted from One Where Necessary so Higher Scores are Preferable to Lower

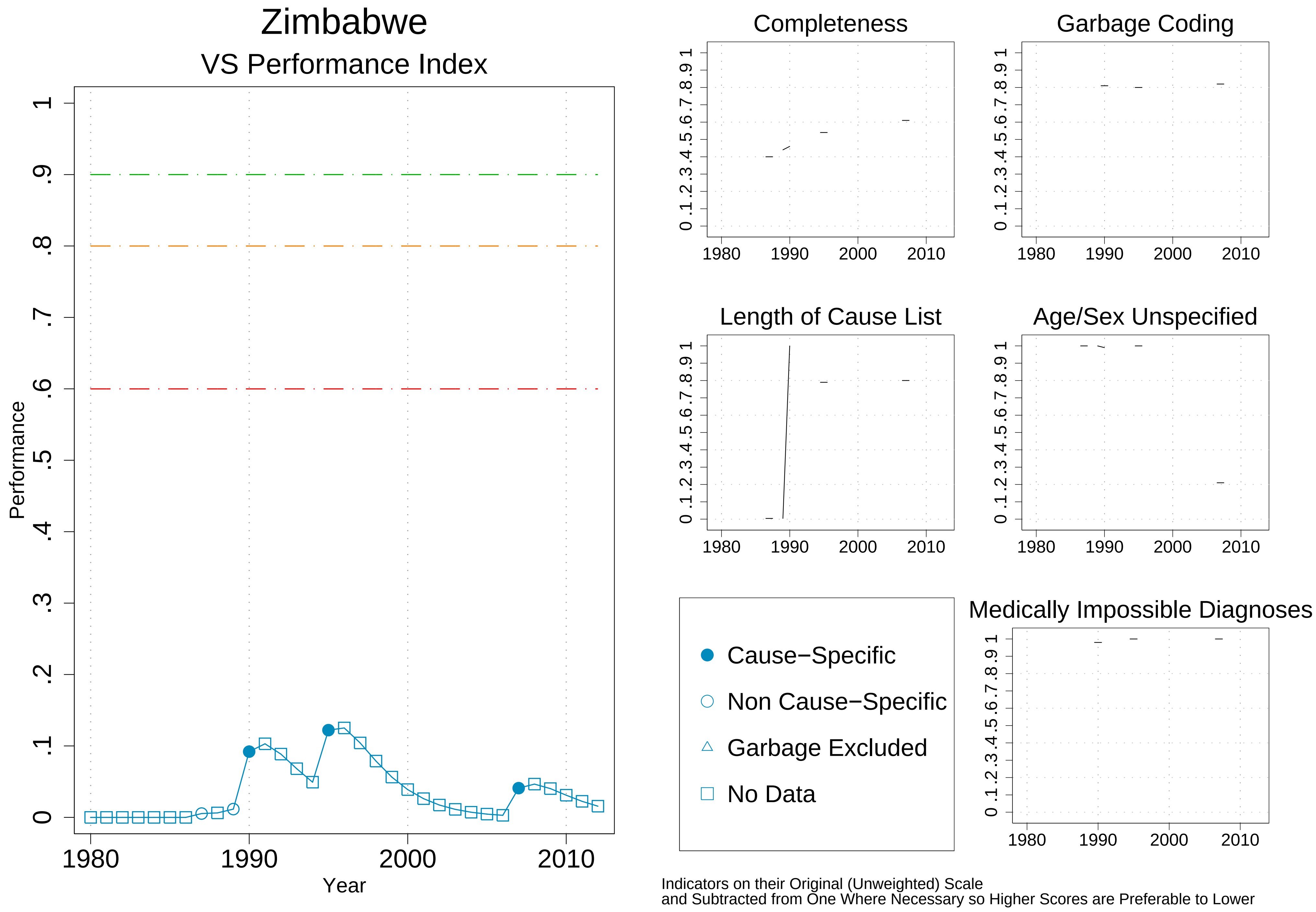

Zimbabwe
VS Performance Index
Performance
Year
Completeness
Garbage Coding
Length of Cause List
Age/Sex Unspecified
Medically Impossible Diagnoses
Cause−Specific
Non Cause−Specific
Garbage Excluded
No Data
Indicators on their Original (Unweighted) Scale
and Subtracted from One Where Necessary so Higher Scores are Preferable to Lower

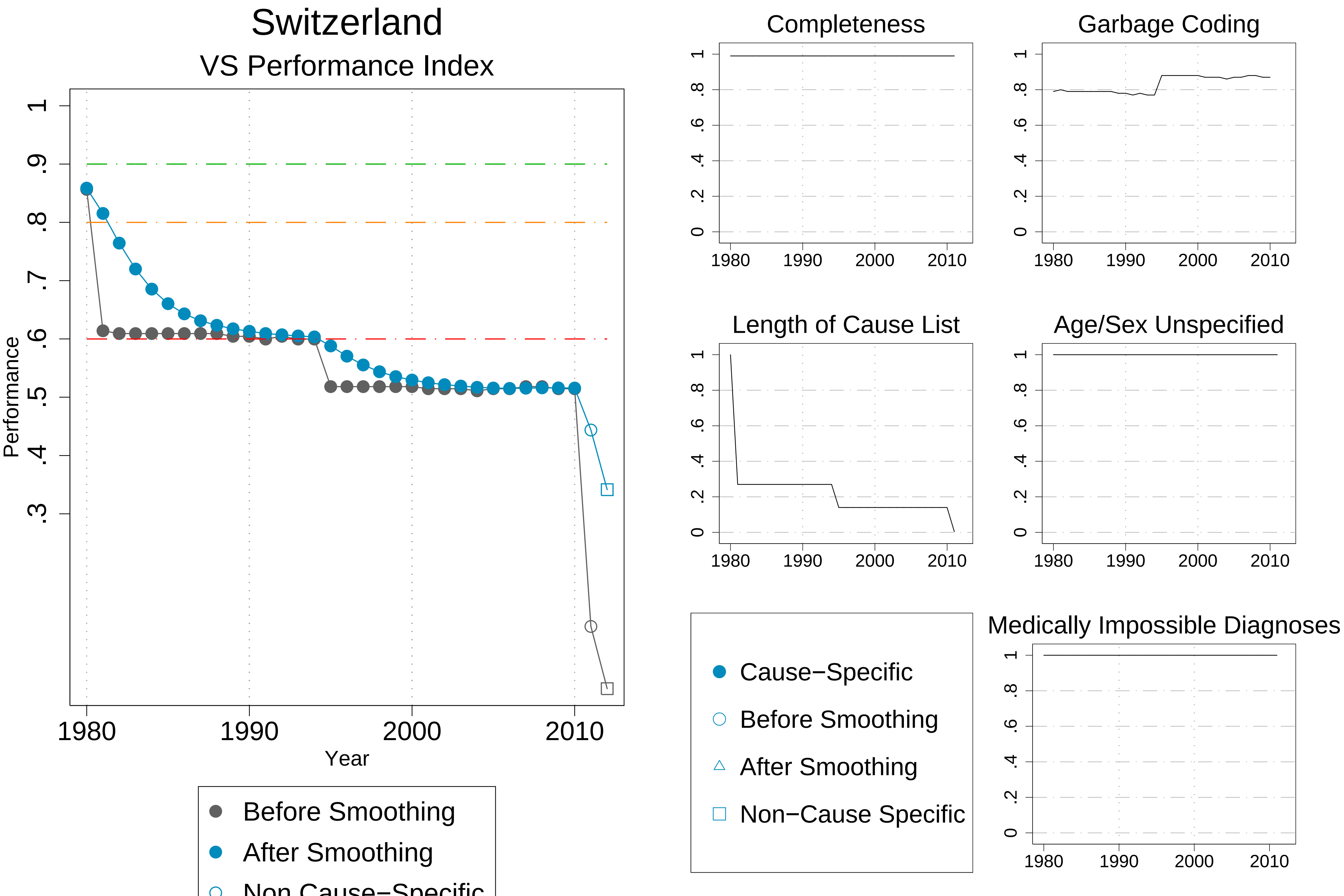

Indicators on their Original (Unweighted) Scale
and Subtracted from One Where Necessary so Higher Scores are Preferable to Lower
