# Supplementary material for: A composite metric for assessing data on mortality and causes of death: the vital statistics performance index
Source: Popul Health Metr. 2014 May 14;12:14. doi: 10.1186/1478-7954-12-14 (PMC4060759; doi:10.1186/1478-7954-12-14)
Supplement: Additional file 2: Text 1 — Methods – Simulation. Text 2. Methods – Smoothing. Text 3. Discussion – Timeliness and Availability. Text 4. Discussion – Potential Methodological Limitations. [file 1478-7954-12-14-S2.docx]

**Additional file 2**

**Contents**

Additional Text 1. Methods – Simulation

Additional Text 2. Methods – Smoothing

Additional Text 3. Discussion – Timeliness and Availability

Additional Text 4. Discussion –Potential Methodological Limitations

**Methods - Simulation**

*The following is additional description of the simulation procedure as it pertains to each of the six components.*

The simulation described in the text relies on a cause-specific probability (such as the proportion of all deaths with age or sex unspecified) to inform the procedure. As stated in the text, this probability was directly estimated for the indicators of age or sex unspecified and medically impossible diagnoses. Using all ICD-10-coded country-years, the number of deaths with either unspecified age or sex or the number of deaths with a medically impossible diagnosis were counted and divided by the total number of deaths by cause. This fraction was then applied to the simulation data in order to mimic a dataset with a realistic cause of death composition for a certain level of one of the indicators.

For garbage coding, cause-specific proportions of deaths coded to garbage cannot be directly computed from VS data. Following the principles outlined by Naghavi and colleagues, we redistributed all garbage codes according to garbage-code-specific assigned proportions onto garbage-code-specific target codes.(1) Once completed, we computed the proportion by which each CSMF increased as a result of garbage code redistribution, and used these proportions as the set of probabilities to selectively drop deaths at incrementally higher levels of simulated garbage, according to the same procedure described previously.

Similar additional steps needed to be taken for simulating VS completeness. There is no way to directly estimate the proportion of each CSMF which would be lost if completeness were to decrease. To approximate this proportion, we built on previous work to estimate the probability of death in hospital for each cause of death, using the same methodology as published by Murray and colleagues.(2) An assumption thus had to be made that the probability of a death occurring in a hospital is a reasonable proxy for the probability of a death being observed in a less-than-complete VS system (see Discussion). These probabilities were then used in an identical fashion to the proportions computed for the previous indicators to simulate decreasing levels of completeness.

One additional step was carried out for the case of completeness that was deemed unnecessary for other indicators. Theoretically, a VS system with zero completeness has zero utility, whereas it can be argued that this is not the case for all other indicators (besides availability data). As a consequence, we rescaled the resulting CSMF accuracy values post-simulation to have an intercept of zero, while still preserving the overall trend. This was accomplished by subtracting the minimum CSMF accuracy value from each CSMF accuracy estimate, then dividing by the range of CSMF accuracy.

To simulate the indicator of length of cause list, it was necessary to take advantage of the hierarchical structure of the GBD 2010 cause list.(3) Instead of defining cause-specific probabilities (as was done for the other indicators), if a given country-year had lower cause-specific detail than the GBD 2010 list of causes of death, available causes were mapped to aggregate categories. For example, a given VS dataset may have reported all injuries together, but other diseases in high detail. In order to simulate the CSMF accuracy associated with such a cause list, all specific injury codes were treated as though zero deaths were assigned, and the category “all injuries” was assigned all injury deaths. Then, CSMF accuracy was computed with a fully-hierarchical cause list, instead of the standard list of mutually-exclusive causes. Additionally, although all other indicators were simulated at all possible levels, the vast number of possible ways one might aggregate the cause list we used made simulating all possible cause lists intractable. Instead, we only performed the simulation for the cause lists which were actually observed in the database, and then fit a log-linear curve to the resultant simulated CSMF accuracy values.

**Methods – Exponential smoothing**

*The following is additional description of the exponential smoothing algorithm.*

As noted in the main text, we incorporated timeliness and availability of data as the sixth indicator of VS performance. This was accomplished by applying a standard technique called double exponential smoothing to carry forward in time the effects of previous VSPI values. (4) Briefly, this model expands on general exponential smoothing by allowing for local trends in a time series, making it suited for smoothing more complicated series, and is often expressed in the form:

$$\hat{S}_{t}=\alpha S_{t}+\left( 1-\alpha\right)\hat{S}_{t-1}$$

Where $S_{t}$ in this case represents the VSPI, $\hat{S}$ is its smoothed value and $\alpha$ is user-defined smoothing constant (4). This model places more weight on more recent time periods, and weights exponentially decay with time. The rate of decay, and thus the extent to which the smoothed function is allowed to fluctuate with the data, is controlled by $\alpha$, which can range from 1 (a model which perfectly follows the data) to 0 (a model which smooths all fluctuations in the series). In this study, $\alpha$ was determined through a similar simulation to that used with the other indicators. The aforementioned set of reference CSMFs was used to estimate the accuracy of approximating a time series of CSMFs with progressively fewer years of data. The $\alpha$ parameter which minimized the in-sample squared error (when smoothing over the simulation results) was chosen, and used as the smoothing parameter across all observed VSPIs. This parameter was approximately 0.419.

**Discussion – Timeliness and Availability**

*The following is an elaboration on the discussion regarding timeliness and availability*

The combined result of the five simulated indicators was smoothed over time in order to add an element of data availability and timeliness. It may be useful to elaborate on the motivation for this with two examples. First, we use an example of a country which has a single year with an anomalously low VSPI in the midst of an otherwise well-performing series of VS data. In this case, it would be unrealistic to describe this country’s VS system as suddenly beginning to perform very poorly, yet it would be realistic to state that the available data describe the *current* epidemiological situation in that country-year less well than before. This is particularly relevant in 2012 and 2011, as many countries exhibit a reporting delay which does not signify a collapse of a VS system (as the VSPI without smoothing might imply), yet deserve to be highlighted. Alternatively, consider a country which has no VS data with the exception of an anomalous observation with a moderate VSPI. In this situation, it would likewise be unrealistic to describe this country’s VS system as briefly performing very well at describing the epidemiology if its country, because (as these authors argue) a time-series of observations is more epidemiologically informative than a single point. Yet, a single year of VS data does yield some important information that should reflect positively on the country’s VS system performance. In both scenarios, it may be argued that the actual performance of a VS system in a given year is therefore best described by taking into account prior years of VS data, rather than simply the present year in isolation.

**Discussion - Potential Methodological Limitations**

*The following is an elaboration on potential methodological limitations*

*Garbage Coding*

The proportion of deaths assigned to a garbage code is naturally dependent on the degree of aggregation in the cause list. By including a separate component for cause-specific detail in the VSPI, we are taking the position that a more comprehensive and granular cause list is more useful for health policy than a shorter list of causes. Although a high degree of garbage coding may not be problematic with an aggregated cause list, we believe that a more dis-aggregated cause list is useful, and therefore garbage coding is critical to VS performance.

The indicator of garbage coding was adjusted to allow for differences between somewhat useful garbage codes and entirely uninformative garbage codes. We were unable to locate any literature to guide the choice of how various categories of garbage codes are likely to be useful for public policy. Given this lack of guiding evidence, we assigned a 50% weight to the somewhat useful garbage codes. This was entirely arbitrary and could have had implications for our index. Limited sensitivity analyses (not displayed) suggest that although the value of VSPI changes depending on the weight given to somewhat useful garbage codes, the relative order of countries remains stable.

Importantly, some degree of garbage coding is to be expected in even the most high-performing VS systems because some cases simply do not have the background information required to make an accurate diagnosis of the underlying cause of death. In such cases however, even a mildly informative diagnosis would yield more epidemiologic information than one which is left completely ambiguous. Adjusting for the composition of garbage codes reflects the likely policy value of even mildly informative epidemiological information, and avoids over-penalizing VS systems for the uncertain circumstances under which they are certifying deaths.

*CSMF Accuracy*

It may be argued that CSMF accuracy is not the sole descriptor of concordance between the observed data and the true epidemiologic profile of a country. Other summary concordance metrics could conceivably be used. Furthermore it may be that evaluating a demographic indicator like age and sex quality on the basis of a metric which is inherently cause-specific measures this indicator’s relationship with epidemiologic accuracy on a scale which is only partially relevant. A solution is not readily available to this problem. It could be advocated that different metrics of accuracy be used for each indicator (such as age-specific or sex-specific mortality fraction accuracy), or that all metrics of accuracy be used for all indicators and subsequently combined together, but both options may have their own sets of methodological issues including the loss of comparability when using a different metric of accuracy for each component, and the additional assumptions required in combining them. For the sake of parsimony and acknowledging that VS data on mortality derive their greatest utility for public policy because of the information they convey about causes of death, we believe that CSMF accuracy is the most appropriate measure against which to assess VS performance.

*Imputation of Completeness Simulation Proportions*

In the completeness simulation processes, it was discovered that a small subset of causes did not have available proportions for simulation. An ad-hoc imputation procedure was put in place to fill in these missing values, which again took advantage of the hierarchical organization of the cause list. Causes at a lower or upper level of the hierarchy were used in place of missing values when necessary. This process, although necessary, could have resulted in inappropriate simulation proportions for certain causes of death. Overall, this was required to fill in the values for 60 out of the 192 causes of death.

*Representativeness of Simulation Proportions*

An inherent assumption made in this analysis is that the garbage, impossible, unspecified age/sex, and completeness proportions (such as those in Table 2 and Additional file 1: Table S3), which were derived from all ICD10 countries, are representative of the proportions that would have been found in any data generating process. The problem inherent to this assumption is that developing countries are underrepresented in the ICD10 dataset, while developed countries are fully represented (to generalize). The choice was made to compute these proportions based off of a single cause list in order avoid any complications surrounding mapping between versions of ICD, let alone special tabulations thereof. To provide an example, some of the tabulations of ICD9 and ICD8 do not include every GBD-level cause of death. As a result, the garbage, impossible or unspecified age/sex proportion associated with these causes would be indeterminate, while others would be calculable. This would result in compositional bias between ICD revisions by cause, altering the final proportions in potentially unfavorable ways. Instead, we simply used the single cause list with the most observations as an approximation of the true proportions, which is ICD10.

Another inherent assumption made in the simulation formula is that the simulation proportions are space and time invariant. This means that the proportions used to selectively drop deaths during simulation were not allowed to change from one year to the next, nor were they allowed to change by region (nor country). This assumption was made for two reasons. First, the geographic distribution of deaths used to compute these proportions was not comprehensive. This fact alone requires some kind of restrictive assumption, as proportions were required for all regions. Second, the authors could find no evidence in the appropriate literature documenting differential patterns of garbage coding, impossible coding, unspecified age/sex reporting, or hospital mortality probability over time for a given cause of death, relative to all other causes. Given this lack of temporal evidence and lack of specially-distributed data, the assumption was made that these proportions are space-time invariant.

*Noise Generation Process*

A similar assumption pertains to the process developed to replicate noise among the GBD 2010 CSMFs used as the foundation for the simulation process. As described earlier, a cause-age-sex-specific RMSE was used in generating random values to be added or subtracted from CSMFs. The inherent assumption is that cause-age-sex-specific noise is again space and time invariant. This assumption may be invalid as the magnitude of stochastic variation may change over time or from one location to another. This could potentially have resulted in simulation data which was either too noisy or too smooth, which consequently would have placed too much or too little weight (respectively) some dimensions.

*Forcing Y-Intercept to Be Zero for Completeness*

As mentioned previously, a special exception was made for the completeness indicator. Because this VS data theoretically have zero utility when this indicator is zero, a constraint was placed on the resulting CSMF accuracy values that they must intersect the y-axis at zero. Technically, this has the effect that it alters the metric of accuracy in completeness simulation from CSMF accuracy to *rescaled* CSMF accuracy, making it arguably incompatible with the metrics of accuracy associated with the other indicators. The possible consequence of this effect is an excess of weight given to completeness, as compared to other indicators. While this problem is acknowledged, no suitable solution could be found.

*Smooth Fit to Cause List*

The final operational decision made in the simulation procedure was to fit a smooth curve to the erratic CSMFs associated with different cause lists, as described previously. The choice of a log-linear curve to describe this relationship was based on convention rather than theory, and one of many other functional forms may have also suitably fit the observations. Furthermore, readers may question the need to fit a smooth curve at all, as CSMF accuracy values were obtained for all observed cause lists. This decision was made in order to aid interpretability of the results, as a monotonically increasing function between cause list length and CSMF accuracy would avoid the confusing circumstance where one cause list takes on a higher CSMF accuracy value than a more detailed alternative cause list. A circumstance such as this could have potentially implied that shorter cause lists are sometimes preferable, an interpretation that is not the intention of this analysis.

1. Naghavi M, Makela S, Foreman K, O’Brien J, Pourmalek F, Lozano R. Algorithms for enhancing public health utility of national causes-of-death data. Popul Health Metr. 2010;8(9).

2. Murray CJL, Lopez AD, Barofsky JT, Bryson-Cahn C, Lozano R. Estimating Population Cause-Specific Mortality Fractions from in-Hospital Mortality: Validation of a New Method. PLoS Med. 2007 Nov 20;4(11):e326.

3. Lozano R, Naghavi M, Foreman K, Lim S, Shibuya K, Aboyans V, et al. Global and regional mortality from 235 causes of death for 20 age groups in 1990 and 2010: a systematic analysis for the Global Burden of Disease Study 2010. The Lancet. 2012 Dec 15;380(9859):2095–128.

4. Brown RG. Smoothing, Forecasting and Prediction of Discrete Time Series. Englewood Cliffs, USA: Prentice-Hall; 1963.
